# Structurally Diverse Diterpenes from the South China Sea Soft Coral *Sarcophyton trocheliophorum*

**DOI:** 10.3390/md21020069

**Published:** 2023-01-20

**Authors:** Yu-Ting Song, Dan-Dan Yu, Ming-Zhi Su, Hui Luo, Jian-Guo Cao, Lin-Fu Liang, Fan Yang, Yue-Wei Guo

**Affiliations:** 1 State Key Laboratory of Drug Research, Shanghai Institute of Materia Medica, Chinese Academy of Sciences, 555 Zu Chong Zhi Road, Zhangjiang Hi-Tech Park, Shanghai 201203, China; 2 College of Life Sciences, Shanghai Normal University, 100 Guilin Road, Shanghai 200234, China; 3 College of Materials Science and Engineering, Central South University of Forestry and Technology, 498 South Shaoshan Road, Changsha 410004, China; 4 Shandong Laboratory of Yantai Drug Discovery, Bohai rim Advanced Research Institute for Drug Discovery, Yantai 264117, China; 5 Key Laboratory of Zhanjiang for Research and Development Marine Microbial Resources in the Beibu Guif Rim, Marine Biomedical Research Institute, Guangdong Medical University, Zhanjiang 524023, China

**Keywords:** soft coral, *Sarcophyton trocheliophorum*, capnosane, cembrane, absolute configuration, anti-tumor activity

## Abstract

The present investigation of the South China Sea soft coral *Sarcophyton trocheliophorum* resulted in the discovery of six new polyoxygenated diterpenes, namely sartrocheliols A–E (**1**, **3**, **5**–**8**) along with four known ones, **2**, **4**, **9**, and **10**. Based on extensive spectroscopic data analysis, sartrocheliol A (**1**) was identified as an uncommon capnosane diterpene, while sartrocheliols B–E (**3**, **5**–**8**) were established as cembrane diterpenes. They displayed diverse structural features not only at the distinctly different carbon frameworks but also at the various types of heterocycles, including the epoxide, γ-lactone, furan, and pyran rings. Moreover, their absolute configurations were determined by a combination of quantum mechanical-nuclear magnetic resonance (QM-NMR) approach, modified Mosher’s method, and X-ray diffraction analysis. In the anti-tumor bioassay, compound **4** exhibited moderate cytotoxic activities against A549, H1975, MDA-MB-231, and H1299 cells with the IC_50_ values ranging from 26.3 to 47.9 μM.

## 1. Introduction

A fairly large variety of bioactive secondary metabolites have been found in the soft corals of the genus *Sarcophyton* [1,2,3,4]. Over the past few decades, about 20 *Sarcophyton* species have been chemically investigated and more than 500 secondary metabolites have been identified. Based on their structural types, these metabolites could be classified into terpenes [2], steroids [3,5,6,7], quinones [8,9], prostaglandins [10,11], ceramides [12,13], and other miscellaneous compounds [8,14,15]. As reported in the literature, terpenes have dominated the chemical profile of the genus *Sarcophyton* [2,16]. Moreover, terpenes display rich structural diversities, which could be further categorized as sesquiterpenes [17,18,19], diterpenes [20,21,22,23,24], and biscembranoids [21,25,26,27,28]. Moreover, these metabolites exhibit a wide spectrum of biological activities, including anti-angiogenic [17], antimicrobial [27,29], cytotoxic [29,30], anti-inflammatory [27,31], immunomodulatory [25], antifouling [8,32], and neuroprotective [33,34] effects. The intriguing chemical and biological properties of terpenes have led to extensive attention from global researchers [35].

In the last decades, we systematically carried out chemical and biological studies on South China Sea marine fauna and flora [36]. As a result, numerous terpenes with complex structures, some of which possessed unprecedented carbon frameworks, were found from the genus *Sarcophyton* [20,37,38]. During our continuous research, we frequently encountered the soft coral *Sarcophyton trocheliophorum*, one productive species belonging to the above-mentioned genus. Previously, our group disclosed a vast array of diterpenes with four skeletons, and these metabolites showed a broad spectrum of pharmacological activities such as protein tyrosine phosphatase 1B (PTP1B) inhibitory, antitumor, antibacterial and neuroprotective activities [39]. It was interesting to notice that the chemical profiles of diterpenes from the title soft corals varied upon temporal variations and geographical distributions. The samples collected in Yalong Bay, Hainan Island, South China Sea in February 2006 yielded diterpenes with an unprecedented carbon framework [37] together with sarsolenane, capnosane, and cembrane skeletons [39]. However, the specimen collected in the same water but in May 2006 only yielded cembrane diterpenes [40]. Of more interest, the soft corals collected from another different region, Ximao Island, South China Sea, merely afforded the cembranoids [41]. These findings probably reflected the existence of different metabolic processes in different seasons and inhabiting environments, which needs to be further investigated.

**Figure 1:**
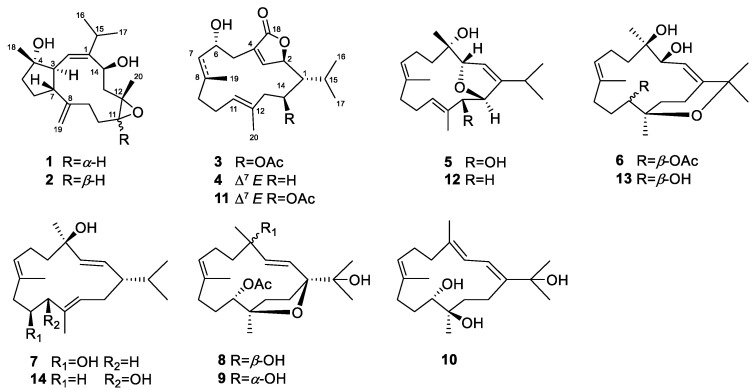
The chemical structures of compounds **1**–**14**.

In order to obtain more evidence to disclose that the diterpenoid profile of the title animals was influenced by temporal variations, we made a new collection of *S. trocheliophorum* from Ximao Island. In the current study, six new polyoxygenated diterpenes, namely sartrocheliols A–E (**1**, **3**, **5**–**8**), and four known related analogs **2**, **4**, **9**, and **10** were obtained (Figure 1). These diterpenes displayed two distinctly different carbon frameworks: capnosane and cembrane, the former of which has rarely been found in soft corals. Meanwhile, various types of heterocycles, including the epoxide, γ-lactone, furan, and pyran rings were incorporated in their macrocyclic skeletons. Herein, we report the detailed structural elucidation of these isolates from the title soft corals, especially the challenging stereochemistry determination of the new compounds, which was dissolved by a combination of quantum mechanical-nuclear magnetic resonance (QM-NMR) approach, modified Mosher’s method, and X-ray diffraction analysis. In addition, their biological evaluations including cytotoxicity against a panel of cancel cells and antibacterial against an array of bacteria are described.

## 2. Results and Discussion

**Figure 2:**
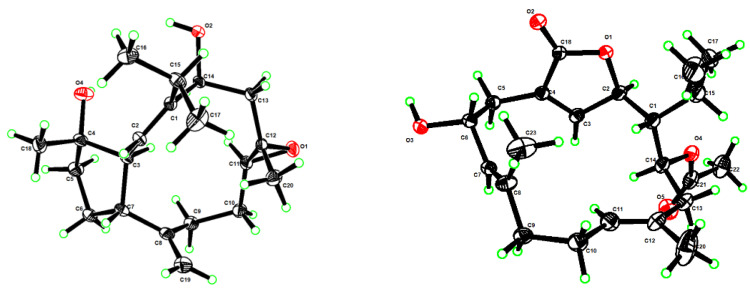
Perspective ORTEP drawing of the X-ray structures of compounds **1** (**left**) and **4** (**right**) (displacement ellipsoids are drawn at the 50% probability level).

The frozen animals were cut into pieces and extracted exhaustively with acetone. Then an Et_2_O-soluble portion of the acetone extract was repeatedly chromatographed over silica gel, Sephadex LH-20, and RP-HPLC to yield six new compounds **1**, **3**, **5**–**8**, and four known analogs **2**, **4**, **9**, and **10** (Figure 1). The known compounds were readily identified as sarcophyolide D (**2**) [42], sarcophytonolide H (**4**) [43], sarcophytrol O (**9**) [44], sinulaflexiolide I (**10**) [45], respectively, based on the comparison of their NMR spectral and specific optical data with those reported in the literature. In our previous work [43], the absolute configuration of sarcophytonolide H (**4**) was established by the modified Mosher’s method. In the present study, its crystals were obtained, which were suitable for X-ray diffraction experiment with Cu K_α_ (*λ* = 1.54178 Å) radiation. The X-ray diffraction analysis allowed the assignment of the absolute configuration of **4** as 1*R*,2*R*,6*R*,14*S* (Flack parameter: –0.03 (9)) (Figure 2, Table S2, CCDC 2205721), which was consistent with our previous study.

**Figure 3:**
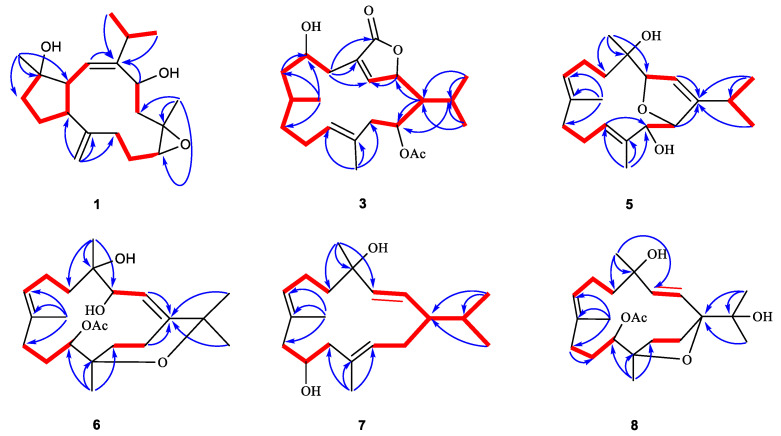
The selected key ^1^H–^1^H COSY (red lines) and HMBC (blue arrows, from ^1^H to ^13^C) correlations of compounds **1**, **3**, **5**–**8**.

Sartrocheliol A (**1**) was obtained as optically active colorless crystals. Its molecular formula C_20_H_32_O_3_ was deduced from the protonated molecule peak at *m*/*z* 343.2243 ([M + Na]^+^, calcd. for C_20_H_32_O_3_Na, 343.2244) in the HRESIMS spectrum, implying five degrees of unsaturation. The IR spectrum indicated the presence of hydroxyl (*ν*_max_ 3441 cm^−1^) and olefinic (*ν*_max_ 1660, 910 cm^−1^) groups. The ^1^H and ^13^C NMR spectra displayed the signals of a trisubstituted double bond (*δ*_H_ 5.14 (1H, d, *J* = 12.6 Hz, H-2), *δ*_C_ 149.90 (qC, C-1), 125.85 (CH, C-2)), a terminal double bond (*δ*_H_ 4.88 (1H, s, H-19a), 4.69 (1H, s, H-19b), *δ*_C_ 148.54 (qC, C-8), 112.26 (CH_2_, C-19)), an epoxide (*δ*_H_ 2.89 (1H, dd, *J* = 10.6, 2.9 Hz, H-11), *δ*_C_ 62.28 (CH, C-11), 59.06 (qC, C-12)), an oxygenated methine (*δ*_H_ 4.88 (1H, dd, *J* = 11.2, 5.1 Hz, H-14), *δ*_C_ 69.34 (CH, C-14)), and an oxygenated carbon (*δ*_C_ 81.64 (C, C-4)) (Table 1 and Table 2). As revealed by the ^1^H and ^13^C NMR data, there were two double bonds and one epoxide, accounting for three degrees of unsaturation. The remaining two degrees of unsaturation were due to the presence of two rings in the molecule. Considering the co-isolated secondary metabolite sarcophyolide D (**2**) [42], compound **1** was likely a capnosane-type diterpene. Indeed, the NMR data of **1** was almost identical to those of **2**, except for the chemical shift of C-11 (*δ*_C_ 62.28 for **1** vs. *δ*_C_ 59.54 for **2**). The interpretation of ^1^H–^1^H COSY and HMBC spectra (Figure 3) indicated they shared the same gross structure. Analysis of the NOESY spectrum (Figure S6) revealed that there was lack of correlation between H-11 (*δ*_H_ 2.89) and H_3_-20 (*δ*_H_ 1.12), which revealed the *trans*-orientation of H-11 and H_3_-20 in **1**, the orientation of which was different from that of the isolate **2**. In order to check the proposed structure as well as establish the absolute configuration of 1, a suitable single crystal was obtained in MeOH after many attempts. A successful performance of X-ray crystallography study using Cu K_α_ (*λ* = 1.54178 Å) radiation firmly confirmed the structure of **1** and determined its absolute configuration ambiguously as 3*S*,4*S*,7*R*,11*S*,12*S*,14*S* (Flack parameter: 0.05 (6)) (Figure 2, Table S1, CCDC 2196207). Hereto, the structure of **1** was established, as shown in Figure 1.

**Figure 4:**
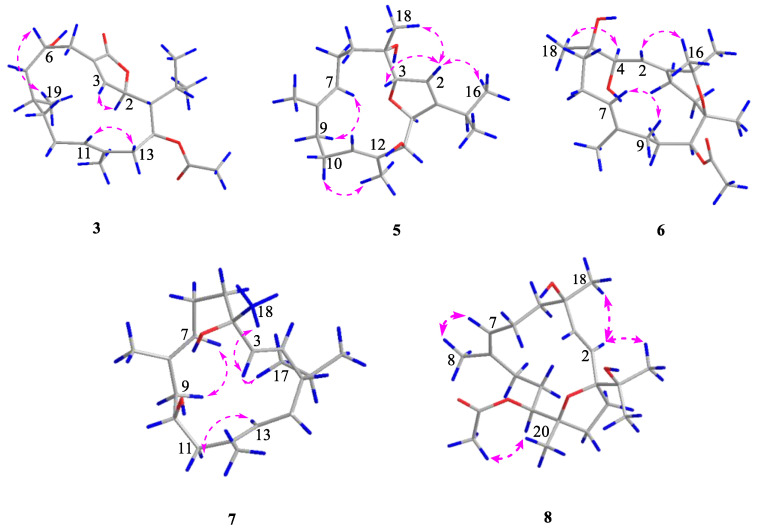
The selected key NOESY (pink dashed lines, from ^1^H to ^1^H) correlations of compounds **3**, **5**–**8**.

**Figure 5:**
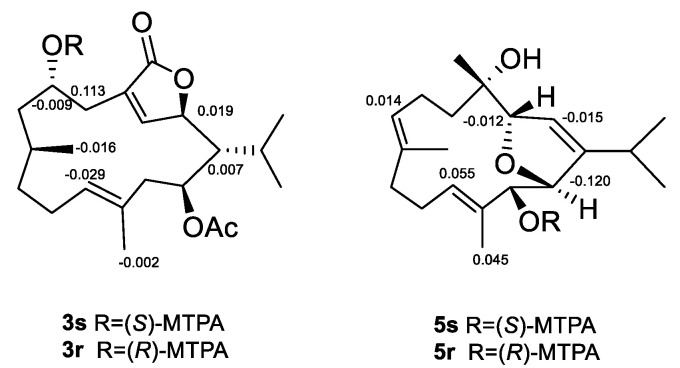
Δ*δ* values (*δ_S_–δ_R_*) (ppm) for (*S*)-and (*R*)-MTPA esters of **3** and **5**.

**Figure 6:**
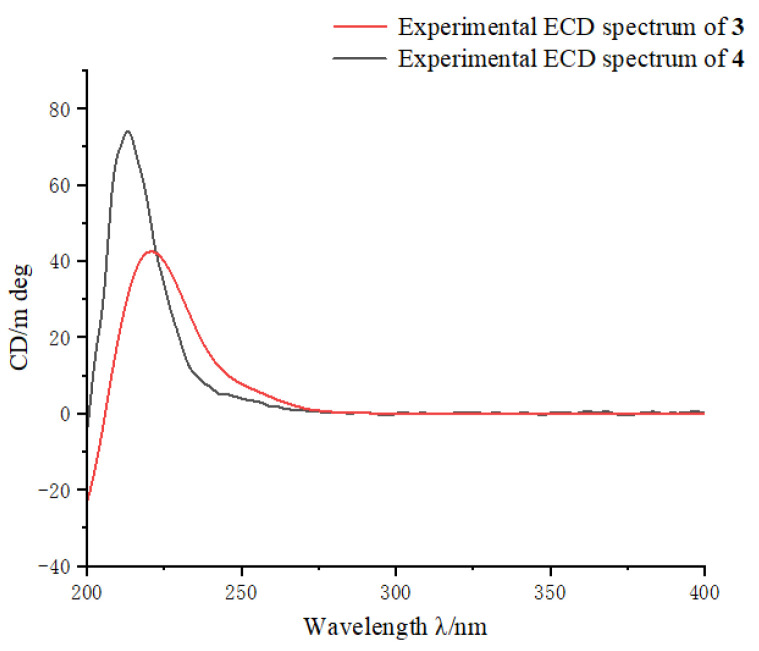
ECD curves of compounds **3** (up) and **4** (down).

Sartrocheliol B (**3**) was isolated as optically active colorless oil. Its molecular formula C_22_H_34_O_5_ was established by the HRESIMS protonated molecule peak at *m*/*z* 379.2482 ([M + H]^+^, calcd. for C_22_H_35_O_5_, 379.2479), which was indicative of six degrees of unsaturation. The IR spectrum of **3** suggested the presence of lactone (*ν*_max_ 1759 cm^−1^), ester (*ν*_max_ 1736 cm^−1^) and hydroxyl (*ν*_max_ 3442 cm^−1^) groups, while a strong UV absorption at *λ*_max_ 231 nm (log*ε* 3.83) suggested the presence of *α*,*β*-unsaturated *γ*-lactone group [43]. According to the ^1^H and ^13^C NMR data of **3** (Table 1 and Table 2), five degrees of unsaturation were attributed to one *α*,*β*-unsaturated *γ*-lactone group (*δ*_H_ 4.95 (1H, d, *J* = 10.7 Hz, H-2), 7.44 (1H, t, *J* = 1.8 Hz, H-3), *δ*_C_ 81.19 (CH, C-2), 151.06 (CH, C-3), 131.39 (qC, C-4), 172.97 (qC, C-18)), one trisubstituted double bond (*δ*_H_ 5.05 (1H, t, *J* = 7.7 Hz, H-11), *δ*_C_ 129.20 (CH, C-11), 130.80 (qC, C-12)) and one acetyl group (*δ*_H_ 2.09 (3H, s), *δ*_C_ 21.35 (CH_3_), 171.13 (qC)). The remaining one degree of unsaturation implied the monocyclic nature of this molecule. Considering the co-isolated cembranoids **4**, **9** and **10**, metabolite **3** was likely a cembrane-type diterpene. Through detailed literature reviews on diterpenes from the genus *Sarcophyton*, the above-mentioned structural features were reminiscent of previously reported sarcophytonolide H (**11**) [43], a cembrane from South China Sea soft coral *Sarcophyton latum*. Our interpretation of the 2D NMR spectra (Figure 3) suggested they differed by the lack of the double bond Δ^7^, which was supported by the significantly up-field shifted chemical shifts of C-7 and C-8 (Δ*δ*_C_ ca. 80 and 110 ppm, respectively). Similar patterns of NOE correlations in the NOESY spectra of **3** and **11**, especially for the key NOE cross-peaks of H-2 (*δ*_H_ 4.95)/H-14 (*δ*_H_ 5.02), H-2/H_3_-16 (*δ*_H_ 1.11), H-3 (*δ*_H_ 7.44)/H-5 (*δ*_H_ 2.45), and H-11 (*δ*_H_ 5.05)/H-13 (*δ*_H_ 2.20), indicated they shared the same configurations for the olefinic bonds Δ^3^ and Δ^11^ and the chiral centers C-1, C-2, and C-14 (Figure 4). The clear NOE correlation of H-6 (*δ*_H_ 4.22)/H_3_-19 (*δ*_H_ 0.77) suggested that H-6 and H_3_-19 possessed the same orientation. Due to the lack of NOE correlations between the two sets: **a**. H-6 and H_3_-19 and **b**. H-1, H-,2 and H-14, the absolute configurations of these chiral centers corresponding to the above-mentioned two sets of protons were determined by the modified Mosher’s method and CD spectrum, respectively. Treatment of **3** with (*R*)- and (*S*)-*α*-methoxy-*α*-trifluoromethylphenyl acetyl chlorides (MTPA-Cl) in dry pyridine successfully afforded the (*S*)- and (*R*)-MTPA ester derivatives **3s** and **3r**, respectively. The distribution pattern of observed Δ*δ*_H(*S*-*R*)_ values (Figure 5) established the absolute configuration *S* for C-6 in **3**. Considering the correlation between H-6 and H_3_-19, the absolute configuration of C-8 could be determined as *S*. It might be worth pointing out that the absolute configuration of sarcophytonolide H (**4**) had already been ambiguously confirmed as 1*R*,2*R*,6*R*,14*S* by the single crystal X-ray diffraction experiment in this study (Figure 2). The CD spectrum of **3** displayed the Cotton effect resembling that of co-isolated **4** (Figure 6), suggesting they shared the same absolute configuration *R* for the chrial carbon C-2 of the chromophore *α*,*β*-unsaturated *γ*-lactone. With the relationships of H-1, H-2, and H-14 in hand, the absolute configurations of C-1 and C-14 could be assigned as *R* and *S*, respectively. Consequently, the absolute configuration of **3** could be established as 1*R*,2*R*,6*S*,8*S*,14*S*.

**Figure 7:**
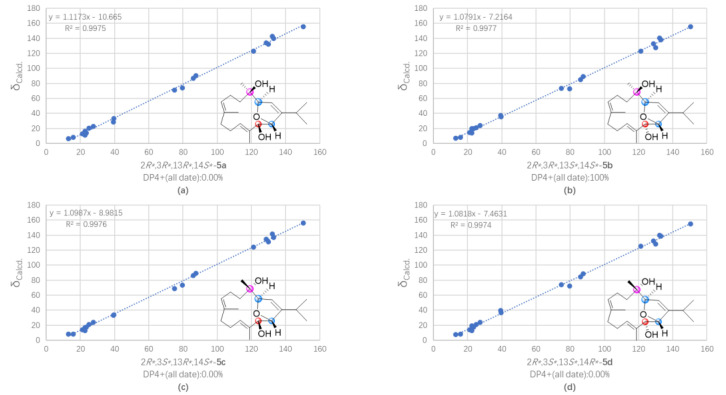
Regression analysis of experimental vs. calculated ^13^C NMR chemical shifts of (**a**) (2R*,3R*,13R*,14S*)-**5a**, (**b**) (2R*,3R*,13S*,14S*)-**5b**, (**c**) (2R*,3S*,13R*,14S*)-**5c**, and (**d**) (2R*,3S*,13S*,14R*)-**5d** at the PCM/mPW1PW91/6-31 + G** level, using the DP4+ method.

The protonated molecule peak at *m*/*z* 343.2237 ([M + Na]^+^, calcd. for C_20_H_32_O_3_Na, 343.2244) displayed in the HRESIMS spectrum of sartrocheliol C (**5**) revealed that compound **5** had the molecular formula C_20_H_32_O_3_, demonstrating the presence of an additional oxygen atom with respect to that of a dihydrofuran cembranoid **12** [46]. The IR spectrum of **5** showed the presence of olefinic (*ν*_max_ 3726 cm^−1^) and hydroxyl (*ν*_max_ 3441 cm^−1^) groups. A careful analysis of its NMR spectra revealed that the NMR spectroscopic features of **5** (Table 1 and Table 2) highly resembled those of **12**. In fact, the main difference between compounds **5** and **12** was that the CH_2_-13 in **12** was hydroxylated in **5**. The presence of a hydroxyl group at C-13 was supported by the dramatically down-field shifted carbon chemical shift (Δ*δ*_C_ 56 ppm), and further confirmed by the diagnostic HMBC correlations of from H-11 (*δ*_H_ 5.32) to C-13 (*δ*_C_ 79.67) and from H_3_-20 (*δ*_H_ 1.56) to C-13 (Figure 3). The cross-peak of H_3_-18 (*δ*_H_ 1.00)/H-2 (*δ*_H_ 5.51) observed in the NOESY spectrum of **5** (Figure 4) together with the small coupling constant (1.4 Hz) between H-2 and H-3 (*δ*_H_ 4.66) suggested the same orientation of H_3_-18 and H-3. Moreover, the coupling constant (4.6 Hz) between H-13 (*δ*_H_ 4.29) and H-14 (*δ*_H_ 4.84) indicated these two protons were *cis*-orientated. Whereas the lack of the NOE correlation between H-3 and H-14 revealed the *trans*-orientation of these two protons of the 2,5-dihydrofuran ring. Thus, the relative configuration of **5** could be assigned as 3R^*^,4R^*^,13S^*^,14S^*^ based on the extensive analysis of the NOESY spectrum. Meanwhile, the relative configuration of **5** could also be elucidated via QM-NMR protocol by using the DP4+ method, which has become one of the most popular and reliable methods to find the most likely structure from a set of putative candidates [34,47]. Four possible isomers (2R*,3R*,13R*,14S*)-**5a**, (2R*,3R*,13S*,14S*)-**5b**, (2R*,3S*,13R*,14S*)-**5c** and (2R*,3S*,13S*,14R*)-**5d** (Table S3) were subjected to QM-NMR calculations. As a result, the experimental NMR data of compound **5** gave the best match for **5b**, with 100% probability (Figure 7, Table S4). Herein, the assigned relative configuration 3R^*^,4R^*^,13S^*^,14S^*^ was consistent with the observations deduced from the NOESY spectrum. As there was a secondary hydroxyl group at C-13, the modified Mosher’s method was applied. The resulting distribution pattern of observed Δ*δ*_H(*S*-*R*)_ values (Figure 5) established the absolute configuration *R* for C-13 in **5**. Subsequently, the absolute configuration of **5** could be assigned as 3*S*,4*S*,13*R*,14*R*.

The HRESIMS spectrum of sartrocheliol D (**6**) displayed a protonated molecule peak at *m*/*z* 403.2459 ([M + Na]^+^, calcd. for C_22_H_36_O_5_Na, 403.2455), suggesting that **6** possessed the molecular formula C_22_H_36_O_5_. Thus, five degrees of unsaturation were determined for **6**. The NMR data (Table 1 and Table 2) revealed the presence of two trisubstituted double bonds (*δ*_H_ 5.42 (1H, m, H-2), *δ*_C_ 146.95 (qC, C-1), 121.34 (CH, C-2); *δ*_H_ 5.24 (1H, t, *J* = 6.3 Hz, H-7), *δ*_C_ 127.40 (CH, C-7), 134.03 (qC, C-8)), one acetyl group (*δ*_H_ 2.08 (3H, s), *δ*_C_ 21.75 (CH_3_), 170.47 (qC)), two oxygenated methines (*δ*_H_ 4.40 (1H, br s, H-3), 5.04 (1H, d, *J* = 10.3 Hz, H-11), *δ*_C_ 72.70 (CH, C-11), 73.16 (CH, C-3)), and three oxygenated quaternary carbons (*δ*_C_ 75.86 (qC, C-4), 74.15 (qC, C-12), 74.60 (qC, C-15)), which accounted for three degrees of unsaturation. The remaining two degrees of unsaturation strongly indicated one macrocyclic carbon skeleton fused with an oxacycle. Careful analysis of the 2D NMR spectrum (Figure 3) of **6** revealed this compound had almost the same gross bicyclic framework of sarcophytrol R (**13**) [44] except the hydroxyl group at C-11 in **13** was acetylated in **6**. Due to the acetylation, the chemical shifts of H-11 and C-11 shifted down-field (Δ*δ*_H_ 1.5 ppm, Δ*δ*_C_ 3.2 ppm, respectively). It might be worth pointing out that the lists of ^1^H and ^13^C NMR data of sarcophytrols R and S in the reference [44] were exchanged inadvertently by the authors. The high similarity of the ^1^H and ^13^C NMR data as well as similar patterns of NOE correlations of compounds **6** and **13** suggested they shared the same relative configuration 3S*,4R*,11S*,12R*. With the relative configuration in hand, we tried to use the Mosher’s method to establish the absolute configuration of this compound. However, to our disappointment, the left compound after bioassay was degraded although kept in a fridge.

**Figure 8:**
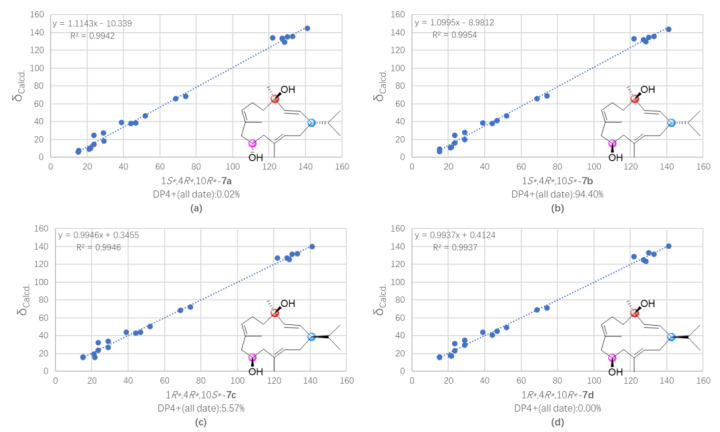
Regression analysis of experimental vs. calculated ^13^C NMR chemical shifts of (**a**) (1S*,4R*,10R*)-**7a**, (**b**) (1S*,4R*,10S*)-**7b**, (**c**) (1R*,4R*,10S*)-**7c**, and (**d**) (1R*,4R*,10R*)-**7d** at the PCM/mPW1PW91/6-31 + G** level, using the DP4+ method.

Sartrocheliol E (**7**) was isolated as colorless oil, and its molecular formula was assigned as C_20_H_34_O_2_ by protonated molecule peak at *m*/*z* 329.2448 ([M + Na]^+^, calcd. for C_20_H_34_O_2_Na, 329.2451) in the HRESIMS spectrum. Its ^13^C NMR data (Table 2), in combination with the DEPT and HSQC spectra, allowed the identification of 20 carbon resonances, involving six olefinic carbons (δ_C_ 141.04, 132.94, 130.05, 128.47, 127.31, 122.10), two oxygenated carbons (δ_C_ 74.20, 68.82), and five methyl carbons (δ_C_ 29.17, 21.85, 21.15, 15.52, 15.09). The presence of six olefinic carbons was attributed to three double bonds, which accounted for three degrees of unsaturation, indicating that **7** had a monocyclic carbon framework. In fact, the NMR spectroscopic characters of **7** were reminiscent of those of cembrendiol (**14**) [48]. Careful comparison of their NMR data disclosed their structures differed at the position of the secondary hydroxyl substituent. The secondary hydroxyl group substituted at C-10 in **7** was supported by the consecutive proton system extending from H_2_-9 to H_2_-11 through H-10 in the ^1^H−^1^H COSY spectrum (Figure 3). Due to the lack of the evidence regarding the orientations of H-10 and H_3_-18, it was hard to figure out the relative configurations of C-4 and C-10. In order to solve this problem, the QM-NMR method was applied (Table S5). By means of this approach, the experimentally observed NMR data for compound **7** gave the best match with the 1S*,4R*,10S* isomer (>90% probability) (Figure 8, Table S6). Thus, the relative configuration of compound **7** was determined as 1S*,4R*,10S*. Unfortunately, the application of Mosher’s reaction failed, probably due to the insufficient amounts left after an array of bioassays.

The protonated molecule peak at *m*/*z* 403.2446 ([M + Na]^+^, calcd. for C_22_H_36_O_5_Na, 403.2455) in the HRESIMS spectrum of sartrocheliol F (**8**) suggested compound **8** and co-isolate **9** [44] had the same molecular formula C_22_H_36_O_5_. Moreover, the NMR data of **8** (Table 1 and Table 2) highly resembled those of **9**, implying that they shared the same gross structure. The distinct difference was found as the chemical shift of C-4, which was δ_C_ 72.47 ppm in **8** whereas δ_C_ 74.49 ppm in **9**, disclosing the reverse configuration of the hydroxyl group at C-4. This reversion was further deduced from the NOE interactions between H_3_-18 and H-2 and between H-2 and H_3_-16 (Figure 4). As the absolute configuration of the deacetylation derivative of **9** was determined as 1*S*,4*S*,11*S*,12*R* by the Mosher’s method [44], the absolute configuration of **8** was consequently established as 1*S*,4*R*,11*S*,12*R*.

Although chemical investigations of the soft coral *S. trocheliophorum* have been well documented in the literature, the present study of this species collected from Ximao Island provided further intriguing results. In the current study, six new cembranoids diterpenoids, sartrocheliols A–E (**1**, **3**, **5**–**8**), along with four known related ones (**2**, **4**, **9**, and **10**) were obtained. Among them, compound **1** was a capnosane diterpene, while others were cembrane diterpenes. They displayed diverse structural features not only at the distinctly different carbon frameworks but also at the various types of heterocycles, including the epoxide, γ-lactone, furan, and pyran rings. Compared with the previous research of the Ximao Island specimen [41], the major difference was the discovery of a capnosane diterpene in this study. This observation further indicated the possible impact of temporal variations on the different metabolic processes for the title soft corals.

The anti-tumor effects of all the ten secondary metabolites were evaluated against a list of tumor cells including A549 (human lung cancer cell), H1975 (human lung adenocarcinoma cell), MDA-MB-231 (human breast cancer cell) and H1299 (human non-small cell lung cancer cell). The results showed compound **4** displayed moderate cytotoxic activities against these four cells with the IC_50_ values of 47.9, 26.3, 44.7, 33.1 μM, respectively, while **6** only showed moderate cytotoxicity against H1975 (IC_50_ = 40.4 μM). Further, we also conducted molecular interaction experiments on all the compounds, looking for compounds that have the potential to bind to BRD4 and ROR1 anti-tumor targets, respectively. Unfortunately, we did not obtain satisfactory results. In addition, these compounds were tested for their antibacterial activities against a vast array of bacteria including the human pathogens *Staphylococcus aureus* ATCC27154, *Enterococcus faecium*, *Escherichia coli* ATCC25922, *Enterobacter cloacae* ZR042, *Enterobacter hormaechei* 2R043, *Pseudomonas aeruginosa* ATCC10145, methicillin-resistant *Staphylococcus aureus* (MRSA), and *Candida albicans* ATCC76485 and the marine strains *Streptococcus parauberis* KSP28, *Streptococcus parauberis* SPOF3K, *Lactococcus garvieae* MP5245, *Aeromonas salmonicida* AS42, Phoyobacterium damselae FP2244, *Pseudomonas fulva* ZXM181, *Photobacterium halotolerans* LMG22194T. To our disappointment, all of them were judged as inactive. Other bioassays such as neuroprotective and anti-inflammatory are currently on the way.

## 3. Materials and Methods

### 3.1. Subsection

Melting points were measured on an X-4 digital micromelting point apparatus. The X-ray measurements were made on a Bruker D8 Venture X-ray diffractometer with Cu Kα radiation (Bruker Biospin AG, Fällanden, Germany). IR spectra were recorded on a Nicolet iS50 spectrometer (Thermo Fisher Scientific, Madison, WI, USA). Optical rotations were measured on a PerkinElmer 241MC polarimeter (PerkinElmer, Fremont, CA, USA). CD & UV spectra were measured on a JASCO J-810 instrument (JASCO Corporation, Tokyo, Japan). ^1^H and ^13^C NMR spectra were acquired on a Bruker AVANCE III 400 and 600 MHz spectrometer (Bruker Biospin AG, Fällanden, Germany). Chemical shifts were reported with the residual CHCl_3_ (*δ*_H_ 7.26; *δ*_C_ 77.16) as the internal standard for ^1^H and ^13^C NMR spectra. The LREIMS and HREIMS data were recorded on a Finnigan-MAT-95 mass spectrometer (Finnigan-MAT, San Jose, CA, USA). HRESIMS spectra were recorded on Agilent G6250 Q-TOF (Agilent, Santa Clara, CA, USA). Commercial silica gel (Qingdao Haiyang Chemical Co., Ltd., Qingdao, China, 200–300 mesh, 300–400 mesh) was used for column chromatography, and precoated silica gel GF254 plates (Sinopharm Chemical Reagent Co., Shanghai, China) were used for analytical TLC. Sephadex LH-20 (Pharmacia, Piscataway, NJ, USA) was also used for column chromatography. Reversed-phase (RP) HPLC was performed on an Agilent 1260 series liquid chromatography equipped with a DAD G1315D detector at 210 nm (Agilent, Santa Clara, CA, USA). An Agilent semi-preparative XDB-C18 column (5 μm, 250 × 9.4 mm) was employed for the purification. All solvents used for column chromatography and HPLC were of analytical grade (Shanghai Chemical Reagents Co., Ltd., Shanghai, China) and chromatographic grade (Dikma Technologies Inc., Foothill Ranch, CA, USA), respectively.

### 3.2. Animal Material

The soft coral *Sarcophyton trocheliophorum* was collected by scuba at a depth of 15 m in May 2018 in Ximao Island, Hainan Province, China. The animal material was identified by Prof. Xiu-Bao Li from Hainan University. A voucher specimen (No. 18XD-19) is available for inspection at the Shanghai Institute of Materia Medica, CAS.

### 3.3. Extraction and Isolation

The frozen animals (551 g, dry weight) were cut into pieces and extracted exhaustively with acetone at room temperature (3 × 3 L, 30 min in ultrasonic bath). The organic extract was evaporated to give a brown residue (80 g), which was partitioned between Et_2_O and H_2_O. The Et_2_O solution was concentrated under reduced pressure to give a dark brown residue (55.3 g), which was fractionated by gradient silica gel (200–300 mesh) column chromatography (0 → 100% Et_2_O in petroleum ether (PE)), yielding seven fractions (A–G). Fractions E and F were subjected to a column of Sephadex LH-20 eluted with CH_2_Cl_2_ and PE/CH_2_Cl_2_/MeOH (2:1:1) to remove the fatty acids and give ten subfractions (EA–EF and FA–FF), respectively. The subfraction EE was purified by semi-preparative HPLC (60% → 100% MeCN in 20 min, 2.5 mL/min), yielding compounds **5** (3.6 mg; *t*_R_ 10.0 min) and **10** (1.5 mg; *t*_R_ 12.0 min). FCA-FCC was got from subfraction FC by silica gel column chromatography (300—400 mesh, PE/Et_2_O (100:1 → 70:1)). The subfraction FCA afforded compounds **2** (1.0 mg; *t*_R_ 7.1 min), **6** (0.9 mg; *t*_R_ 17.0 min), **8** (0.9 mg; *t*_R_ 18.9 min) and **9** (2.0 mg; *t*_R_ 12.0 min) through semi-preparative HPLC (70% MeCN, 2.5 mL/min). While subfraction FCB gave compounds **1** (1.3 mg; *t*_R_ 7.0 min) and **7** (1.6 mg; *t*_R_ 16.0 min), through semi-preparative HPLC (70% MeCN, 2.5 mL/min) as well. The subfraction FD afforded compounds **3** (3.2 mg; *t*_R_ 6.2 min) and **4** (8.0 mg; *t*_R_ 5.5 min) through semi-preparative HPLC (70% MeCN, 2.5 mL/min).

### 3.4. Spectroscopic Data of Compounds

Sartrocheliol A (**1**): colorless crystals; [α${]}_{D}^{20}$ +250.0 (*c* 0.05, MeOH); IR (KBr) *ν*_max_: 3441, 2925, 1384 cm^−1^; ^1^H and ^13^C NMR data, see Table 1 and Table 2; HRESIMS *m*/*z* 343.2243 [M + Na]^+^ (calcd. for C_20_H_32_NaO_3_, 343.2244).

Sartrocheliol B (**3**): colorless oil; [α${]}_{D}^{20}$ +12.7 (*c* 0.05, MeOH); UV (MeOH) *λ*_max_ (log *ε*) 240 (3.23) nm; CD (MeOH) *λ*_max_ (∆*ε*) 240 (+4.06), 285 (−1.92) nm; IR (KBr) ν_max_: 3442, 2932, 1759, 1736, 1234, 1047, 1023 cm^−1^; ^1^H and ^13^C NMR data, see Table 1 and Table 2; HRESIMS *m*/*z* 379.2479 [M + H]^+^ (calcd. for C_22_H_34_O_5_, 379.2482).

Sartrocheliol C (**5**): colorless oil; [α${]}_{D}^{20}$ -12.7 (*c* 0.1, MeOH); IR (KBr) *ν*_max_: 3726, 3624, 3441, 2960, 1384, 1087, 1032 cm^−1^; ^1^H and ^13^C NMR data, see Table 1 and Table 2; HRESIMS *m*/*z* 343.2237 [M + Na]^+^ (calcd. for C_20_H_32_NaO_3_, 343.2244).

Sartrocheliol D (**6**): colorless oil; [α${]}_{D}^{20}$ -20.0 (*c* 0.05, MeOH); IR (KBr) *ν*_max_: 3443, 2927, 1384, 1038 cm^−1^; ^1^H and ^13^C NMR data, see Table 1 and Table 2; HRESIMS *m*/*z* 403.2459 [M + Na]^+^ (calcd. for C_22_H_36_NaO_5_, 403.2455).

Sartrocheliol E (**7**): colorless oil; [α${]}_{D}^{20}$ +120.0 (*c* 0.01, MeOH); IR (KBr) *ν*_max_: 3446, 2922, 1384, 1142, 1044 cm^−1^; ^1^H and ^13^C NMR data, see Table 1 and Table 2; HR-ESIMS *m*/*z* 329.2448 [M + Na]^+^ (calcd. for C_20_H_34_NaO, 329.2451).

Sartrocheliol F (**8**): colorless oil; [α${]}_{D}^{20}$ +40.0 (*c* 0.05, MeOH); IR (KBr) *ν*_max_: 3442, 1384 cm^−1^; ^1^H and ^13^C NMR data, see Table 1 and Table 2; HR-ESIMS *m*/*z* 403.2446 [M + Na]^+^ (calcd. for C_22_H_36_NaO_5_, 403.2455).

### 3.5. X-ray Crystallographic Analysis for Compounds ***1*** and ***4***

The crystals of **1** and **4** were both recrystallized from methanol at 4 °C. X-ray analysis of **1** and **4** were carried out on a Bruker D8 Venture diffractometer with Cu Kα radiation (λ = 1.54178 Å) at 170 K, respectively. The acquisition parameters for **1** and **4** are provided in the Supplementary Materials, and crystallographic data for compounds **1** and **4** (deposition no. CCDC 2,196,207 and CCDC 2205721) have been deposited at the Cambridge Crystallographic Data Center. Copies of the data can be obtained free of charge via www.ccdc.cam.ac.uk/conts/retrieving.html (accessed on 11 August 2022).

### 3.6. Esterification of Compounds ***3*** and ***5*** with MTPA Chlorides

Compounds **3** and **5** (2.0 mg each) were dissolved in dry pyridine (1.2 mL), divided them into two sets (0.6 mL each), then treated with (*R*)-(–)-2-methoxy-2-(trifluoromethyl) phenylacetyl chloride ((*R*)-(–)-MTPA-Cl) (10 μL) and (*S*)-(+)-2-methoxy-2-(trifluoromethyl) phenylacetyl chloride ((*S*)-(+)-MTPA-Cl) (10 μL), respectively. After stirring overnight at room temperature, the solutions were evaporated in vacuo and the residues were purified by silica gel column chromatography (PE/Et_2_O = 90:10) to obtain the *S*-MTPA ester **3r** (0.3 mg), *R*-MTPA ester **3s** (0.3 mg), *S*-MTPA ester **5r** (0.3 mg), and *R*-MTPA ester **5s** (0.3 mg), respectively. The obtained products were then subjected to the ^1^H NMR experiment.

### 3.7. QM-NMR Calculation of Compounds ***5*** and ***7***

Theoretical calculations of all theoretical stereoisomers were carried out to determine the relative configuration of **5** and **7**, based on the alignment of its ^1^D NMR chemical shifts (^13^C NMR chemical shifts herein) and calculation-generated chemical shifts. Confab was used to search the conformational space of **5a**–**5d** and **7a**–**7d**. Conformational searches were carried out using the torsional sampling (MCMM) method and OPLS_2005 force field in the Macromodel 9.9.223 software applying an energy window of 21 kJ/mol. Conformers above 1% population were re-optimized with Gaussian 09 at the B3LYP/6-311G(d,p) level with IEFPCM (Polarizable Continuum Model using the Integral Equation Formalism variant) solvent model for acetonitrile. The Boltzmann populations of the conformers were obtained based on the potential energy provided by the OPLS_2005 force field, leading to 11, 9, 13 and 24 conformers for **5a**–**5d**; 11, 9, 13 and 24 conformers for **7a**–**7d** above 1% population for further re-optimization, respectively. The obtained conformers were subjected to optimization and frequency calculations on B3LYP/6-311G(d,p) level of theory. GIAO DFT ^13^C NMR calculations were calculated on mPW1PW91/6-31G* (CHCl_3_) level of theory, and the calculated shielding tensors were Boltzmann averaged according to Gibbs free energy and then converted into chemical shifts following MSTD protocol. The experimental ^13^C NMR data of **5** and **7** were compared with the calculated NMR chemical shifts of **5a**–**5d** and **7a**–**7d** using the mean absolute error (MAE) values, maximum deviation (MD) values, correlation coefficient (R^2^), and DP4+ probability analysis. XYZ data for all conformations and detailed data for DP4+ analysis are provided in the Supplementary Materials.

### 3.8. Cytotoxic Bioassays

H1975, MDA-MB-231, A549, and H1299 cancer cell lines were purchased from the Procell Life Science & Technology Co., Ltd. Cells were cultured at 37 °C in a 5% CO_2_ humidified incubator and maintained in high glucose Dulbecco’s Modified Eagle Medium (DMEM, Nissui, Tokyo, Japan) containing 100 mg/mL streptomycin, 2.5 mg/L amphotericin B and 10% heat-inactivated fetal bovine serum (FBS). The cells were inoculated in 96-well culture plates for 12 h and then treated with different concentrations of compounds for 72 h. Water-soluble tezole (WST) reagent was added to each (10 µL) well and cultured at 37 °C for 2 h to assess cell viability. The absorbance was read with a microplate reader at 450 nm. Adriamycin (DOX) was the positive control.

## 4. Conclusions

In summary, the present investigation of the soft coral *S. trocheliophorum* from Ximao Island provided intriguing results including six new cembranoids diterpenoids, sartrocheliols A–E (**1**, **3**, **5**–**8**), along with four known related ones (**2**, **4**, **9**, and **10**). This study not only extended the members of capnosane and cembrane diterpenes but also enriched the chemical diversity of the title species. In addition, a whole set of NMR computations, modified Mosher’s method, and X-ray diffraction analysis were applied to assign the absolute configurations of compounds **1**, **3**, **4**, and **5**. However, the absolute configurations of other new metabolites remain undefined. To achieve it, a collection of the biological materials and accumulation of these new compounds should be conducted in future, which could supply sufficient amounts of metabolites for either the subsequent chemical transformations or recrystallization experiments. Anti-tumor and antibacterial bioassays were carried out. Among these secondary metabolites, compound **4** displayed moderate cytotoxicity against A549, H1975, MDA-MB-231, and H1299 cells (IC_50_ = 47.9, 26.3, 44.7, 33.1 μM, respectively), while **6** only exhibited moderate cytotoxicity against H1975 (IC_50_ = 40.4 μM). However, none of them were active in the antibacterial bioassay. Moreover, the bioactivities of these compounds such as anti-virus, will be evaluated in future.

## Figures and Tables

**Table 1:** ^1^H NMR spectroscopic data of sartrocheliols A–E (**1**, **3**, **5**–**8**) in CDCl_3_.

No.	1 ^a^	3 ^b^	5 ^b^	6 ^b^	7 ^b^	8 ^a^
1		1.55 (d, 10.7)			1.54 (br s)	
2	5.14 (d, 10.6)	4.95 (d, 10.7)	5.51 (d, 1.4)	5.42 (m)	5.51 (dd, 15.6, 9.6)	5.56 (d, 15.6)
3	2.84 (t, 10.6)	7.44 (t, 1.8)	4.66 (dq, 5.5, 1.4)	4.40 (br s)	5.63 (d, 15.6)	6.13 (d, 15.9)
4						
5	1.84 (m)	2.75 (dt, 13.2, 1.2)	1.83 (m)	1.94 (m)	1.89 (ddd, 13.4, 8.6, 1.9)	1.87 (m)
	1.78 (m)	2.45 (dd, 13.3, 10.5)	1.53 (m)	1.76 (m)	1.58 (m)	1.69 (m)
6	1.66 (m)	4.22 (t, 9.8)	2.34 (m)	2.06 (m)	2.24 (m)	2.64 (m)
			1.92 (m)			
7	2.48 (t, 7.1)	1.63 (m)	5.22 (m)	5.24 (t, 6.3)	5.11 (d, 7.8)	5.43 (dd, 10.6, 4.0)
		1.35 (m)				
8		1.29 (m)				
9	2.33 (m)	1.36 (m)	2.10 (m)	1.96 (m)	2.25 (m)	2.00 (m)
	2.00 (t, 13.5)	1.26 (m)		1.67 (m)	2.12 (m)	
10	2.21 (m)	2.08 (m)	2.26 (m)	1.86 (m)	4.07 (d, 10,9)	1.90 (m)
	1.46 (m)		2.11 (m)	1.43 (m)		1.55 (m)
11	2.89 (dd, 10.6, 2.9)	5.05 (t, 7.7)	5.32 (m)	5.04 (d, 10.3)	2.09 (m)	5.08 (d, 9.4)
12						
13	2.25 (m)	2.26 (dd, 13.5, 3.0)	4.29 (t, 4.3)	1.94 (m)	5.24 (t, 7.7)	1.78 (m)
	1.65 (m)	2.20 (dd, 13.5, 10.9)		1.61 (m)		1.59 (m)
14	4.88 (dd, 11.2, 5.1)	5.02 (ddd, 10.8, 4.2, 1.1)	4.84 (td, 5.0, 1.6)	3.04 (m)	2.07 (m)	2.33 (td, 11.8, 7.4)
				2.38 (m)		1.68 (m)
15	2.48 (m)	2.22 (m)	2.65 (m)		1.70 (m)	
16	1.12 (d, 6.5)	1.11 (d, 6.6)	1.16 (d, 6.8)	1.37 (s)	0.96 (d, 6.6)	1.11 (s)
17	1.16 (d, 6.5)	1.12 (d, 6.6)	1.08 (d, 6.9)	1.30 (s)	0.84 (d, 6.7)	1.13 (s)
18	1.16 (s)		1.00 (s)	1.20 (s)	1.30 (s)	1.35 (s)
19	4.88 (s)	0.77 (d, 6.0)	1.56 (s)	1.56 (s)	1.54 (s)	1.71 (s)
	4.69 (s)					
20	1.12 (s)	1.67 (s)	1.56 (s)	1.10 (s)	1.57 (m)	1.17 (s)
OAc		2.09 (s)		2.08 (s)		2.05 (s)

^a^ 500 MHz. ^b^ 600 MHz.

**Table 2:** ^13^C NMR spectroscopic data (125 MHz, CDCl_3_) of sartrocheliols A–E (**1**, **3**, **5**–**8**).

No.	1	3	5	6	7	8
1	149.90, qC	49.67, CH	150.34, qC	146.95, qC	51.96, CH	91.52, qC
2	125.85, CH	81.19, CH	121.20, CH	121.34, CH	122.10, CH	129.84, CH
3	51.08, CH	151.06, CH	87.63, CH	73.16, CH	141.04, CH	137.41, CH
4	81.64, qC	131.39, qC	74.93, qC	75.86, qC	74.20, qC	72.47, qC
5	41.04, CH_2_	37.42, CH_2_	39.37, CH_2_	39.18, CH_2_	44.21, CH_2_	41.64, CH_2_
6	25.90, CH_2_	66.40, CH	22.57, CH_2_	21.75, CH_2_	23.66, CH_2_	22.90, CH_2_
7	54.09, CH	47.19, CH_2_	128.74, CH	127.40, CH	127.31, CH	130.06, CH
8	148.54, qC	28.31, CH	133.16, qC	134.03, qC	130.05, qC	132.11, qC
9	24.65, CH_2_	37.25, CH_2_	39.64, CH_2_	33.90, CH_2_	46.88, CH_2_	34.86, CH_2_
10	28.07, CH_2_	24.31, CH_2_	25.00, CH_2_	25.03, CH_2_	68.82, CH	27.37, CH_2_
11	62.28, CH	129.20, CH	130.08, CH	72.70, CH	38.96, CH_2_	77.36, CH
12	59.06, qC	130.80, qC	132.19, qC	74.15, qC	132.94, qC	84.58, qC
13	45.46, CH_2_	41.43, CH_2_	79.67, CH	29.33, CH_2_	128.47, CH	35.80, CH_2_
14	69.34, CH	73.92, CH	86.05, CH	20.95, CH_2_	23.68, CH_2_	30.76, CH_2_
15	27.09, CH	25.81, CH	27.43, CH	74.60, qC	29.13, CH	72.84, qC
16	21.23, CH_3_	24.93, CH_3_	21.11, CH_3_	30.42, CH_3_	21.15, CH_3_	26.01, CH_3_
17	23.55, CH_3_	18.74, CH_3_	22.57, CH_3_	28.85, CH_3_	21.85, CH_3_	24.62, CH_3_
18	27.70, CH_3_	172.97, qC	23.36, CH_3_	21.72, CH_3_	29.17, CH_3_	29.00, CH_3_
19	112.26, CH_2_	18.25, CH_3_	15.84, CH_3_	17.41, CH_3_	15.52, CH_3_	16.33, CH_3_
20	23.05, CH_3_	18.47, CH_3_	12.89, CH_3_	23.63, CH_3_	15.09, CH_3_	20.69, CH_3_
OAc		171.13, qC		170.47, qC		170.17, qC
		21.35, CH_3_		21.75, CH_3_		21.42, CH_3_

## Data Availability

The data presented in this study are available on request from the corresponding author.

## Supplementary Materials

The following supporting information can be downloaded at: https://www.mdpi.com/article/10.3390/md21020069/s1, Figures S1–S48: NMR, HRESIMS and IR data of compounds **1**, **3**, **5**–**8**; Figures S49 and S50: Regression analysis of experimental vs. calculated ^13^C NMR chemical shifts of different isomers of compounds **5** and **7** at the PCM/mPW1PW91/6-31 + G** level using DP4+ method; Tables S1 and S2: X-ray crystallographic data for compounds **1** and **4**; Tables S3 and S5: Cartesian coordinates of all conformers of isomers **5a**–**5b** and **7a**–**7b** used after optimization at the B3LYP/6-311G (d,p) level of theory as required for DP4+ analysis; Tables S4 and S6: DP4+ results obtained using experimental data of compounds **5** *versus* isomers **5a**–**5b** and 7 *versus* isomers **7a**–**7b**.
