# Supplementary material for: Structurally Diverse Diterpenes from the South China Sea Soft Coral Sarcophyton trocheliophorum
Source: Mar Drugs. 2023 Jan 20;21(2):69. doi: 10.3390/md21020069 (PMC9960596; doi:10.3390/md21020069)
Supplement: Supplementary file 1 [file marinedrugs-21-00069-s001.zip › marinedrugs-2131546-supplementary.pdf]

Structurally Diverse Diterpenes from the South China Sea Soft Coral *Sarcophyton trocheliophorum*

Contents

Figure S1. ^{1}H NMR spectrum (400 MHz) of compound 1 in $CDCl_3$ 3
Figure S2. 13C NMR spectrum (125 MHz) of compound 1 in $CDCl_3$ 3
Figure S3. HSQC spectrum (500 MHz) of compound 1 in $CDCl_3$ 4
Figure S4. HMBC spectrum (500 MHz) of compound 1 in $CDCl_3$ 4
Figure S5. ^{1}H-^{1}H COSY spectrum (500 MHz) of compound 1 in $CDCl_3$ 5
Figure S6. NOESY spectrum (500 MHz) of compound 1 in $CDCl_3$ 5
Figure S7. HR-ESIMS of compound 1 6
Figure S8. IR spectrum of compound 1 6
Figure S9. 1H NMR spectrum (400 MHz) of compound 3 in CDCl3 7
Figure S10. ^{13}C NMR spectrum (125 MHz) of compound 3 in $CDCl_3$ 7
Figure S11. HSQC spectrum (500 MHz) of compound 3 in $CDCl_3$ 8
Figure S12. HMBC spectrum (500 MHz) of compound 3 in $CDCl_3$ 8
Figure S13. ^{1}H-^{1}H COSY spectrum (500 MHz) of compound 3 in $CDCl_3$ 9
Figure S14. NOESY spectrum (500 MHz) of compound 3 in $CDCl_3$ 9
Figure S15. HR-ESIMS of compound 3 10
Figure S16. IR spectrum of compound 3 10
Figure S17. 1H NMR spectrum (400 MHz) of compound 5 in $CDCl_3$ 11
Figure S18. ^{13}C NMR spectrum (125 MHz) of compound 5 in $CDCl_3$ 11
Figure S19. HSQC spectrum (500 MHz) of compound 5 in $CDCl_3$ 12
Figure S20. HMBC spectrum (500 MHz) of compound 5 in $CDCl_3$ 12
Figure S21. ^{1}H-^{1}H COSY spectrum (500 MHz) of compound 5 in $CDCl_3$ 13
Figure S22. NOESY spectrum (500 MHz) of compound 5 in $CDCl_3$ 13
Figure S23. HR-ESIMS of compound 5 14
Figure S24. IR spectrum of compound 5 14
Figure S25. ^{1}H NMR spectrum (400 MHz) of compound 6 in $CDCl_3$ 15
Figure S26. ^{13}C NMR spectrum (125 MHz) of compound 6 in $CDCl_3$ 15
Figure S27. HSQC spectrum (500 MHz) of compound 6 in $CDCl_3$ 16
Figure S28. HMBC spectrum (500 MHz) of compound 6 in $CDCl_3$ 16
Figure S29. ^{1}H-^{1}H COSY spectrum (500 MHz) of compound 6 in $CDCl_3$ 17
Figure S30. NOESY spectrum (500 MHz) of compound 6 in $CDCl_3$ 17
Figure S31. HR-ESIMS of compound 6 18
Figure S32. IR spectrum of compound 6 18
Figure S33. ^{1}H NMR spectrum (400 MHz) of compound 7 in $CDCl_3$ 19
Figure S34. ^{13}C NMR spectrum (125 MHz) of compound 7 in $CDCl_3$ 19
Figure S35. HSQC spectrum (500 MHz) of compound 7 in $CDCl_3$ 20
Figure S36. HMBC spectrum (500 MHz) of compound 7 in $CDCl_3$ 20
Figure S37. ^{1}H-^{1}H COSY spectrum (500 MHz) of compound 7 in $CDCl_3$ 21

Figure S38. NOESY spectrum (500 MHz) of compound 7 in $CDCl_3$ 21
Figure S39. HR-ESIMS of compound 7 22
Figure S40. IR spectrum of compound 7 22
Figure S41. ^{1}H NMR spectrum (400 MHz) of compound 8 in $CDCl_3$ 23
Figure S42. 13C NMR spectrum (125 MHz) of compound 8 in $CDCl_3$ 23
Figure S43. HSQC spectrum (500 MHz) of compound 8 in $CDCl_3$ 24
Figure S44. HMBC spectrum (500 MHz) of compound 8 in $CDCl_3$ 24
Figure S45. ^{1}H-^{1}H COSY spectrum (500 MHz) of compound 8 in $CDCl_3$ 25
Figure S46. NOESY spectrum (500 MHz) of compound 8 in $CDCl_3$ 25
Figure S47. HR-ESIMS of compound 8 26
Figure S48. IR spectrum of compound 8 26
Table S1. X-ray crystallographic data for compound 1 26
Table S2. X-ray crystallographic data for compound 4 27
Figure S49. Regression analysis of experimental vs calculated ^{13}C NMR chemical shifts of (2*R**, 3*R**, 13*R**, 14*S**)-5a, (2*R**, 3*R**, 13*S**, 14*S**)-5b, (2*R**, 3*S**, 13*R**, 14*S**)-5c, (2*R**, 3*S**, 13*S**, 14*R**)-5d at the PCM/mPW1PW91/6-31+G** level using DP4+ method 28
Figure S50. Regression analysis of experimental vs calculated ^{13}C NMR chemical shifts of (1*S**, 4*R**, 10*R**)-7a, (1*S**, 4*R**, 10*S**)-7b, (1*R**, 4*R**, 10*S**)-7c, (1*R**, 4*R**, 10*R**)-7d at the PCM/mPW1PW91/6-31+G** level using DP4+ method 29
Table S3. Cartesian coordinates of all conformers of isomers 5a-5b used after optimization at the B3LYP/ 6-311G (d,p) level of theory as required for DP4+ analysis29
Table S4.. DP4+ results obtained using experimental data of compound 5 *versus* isomers 5a-5d 65
Table S5. Cartesian coordinates of all conformers of isomers 7a-7b used after optimization at the B3LYP/ 6-311G (d,p) level of theory as required for DP4+ analysis66
Table S6. DP4+ results obtained using experimental data of compound 7 *versus* isomers 7a-7d 134

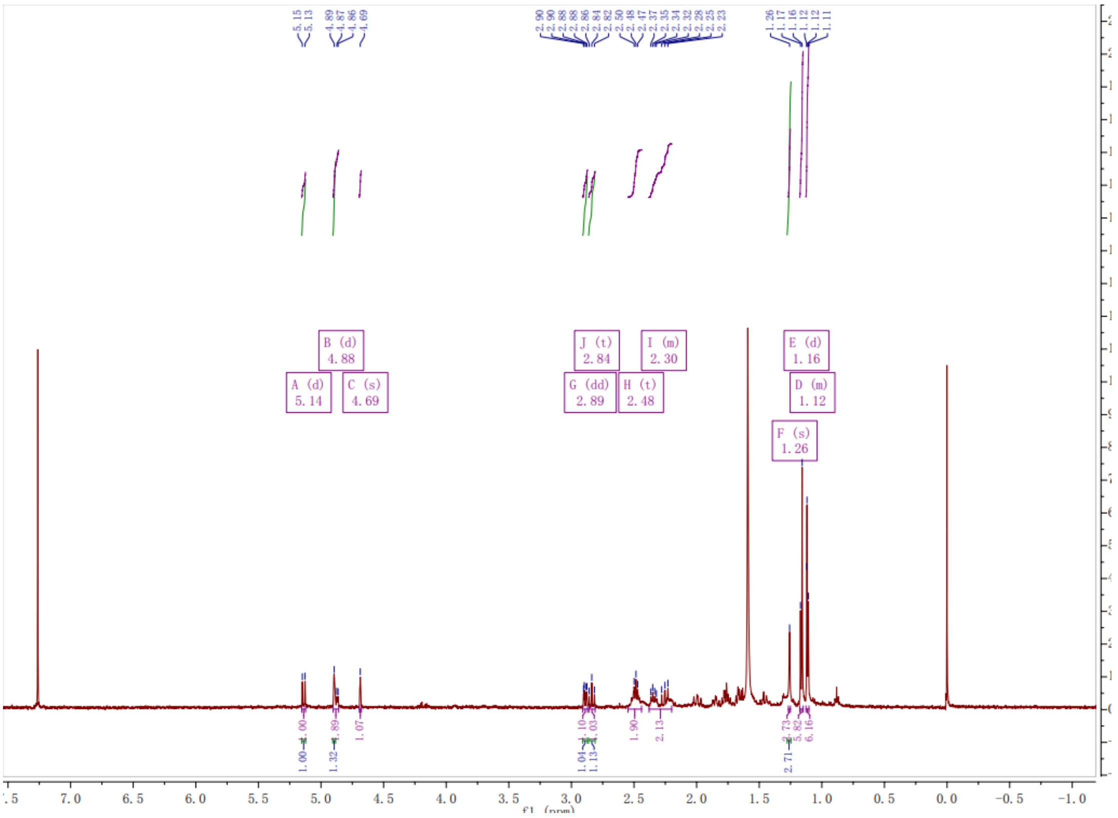

Figure S1. ^{1}H NMR spectrum (400 MHz) of compound 1 in $CDCl_3$

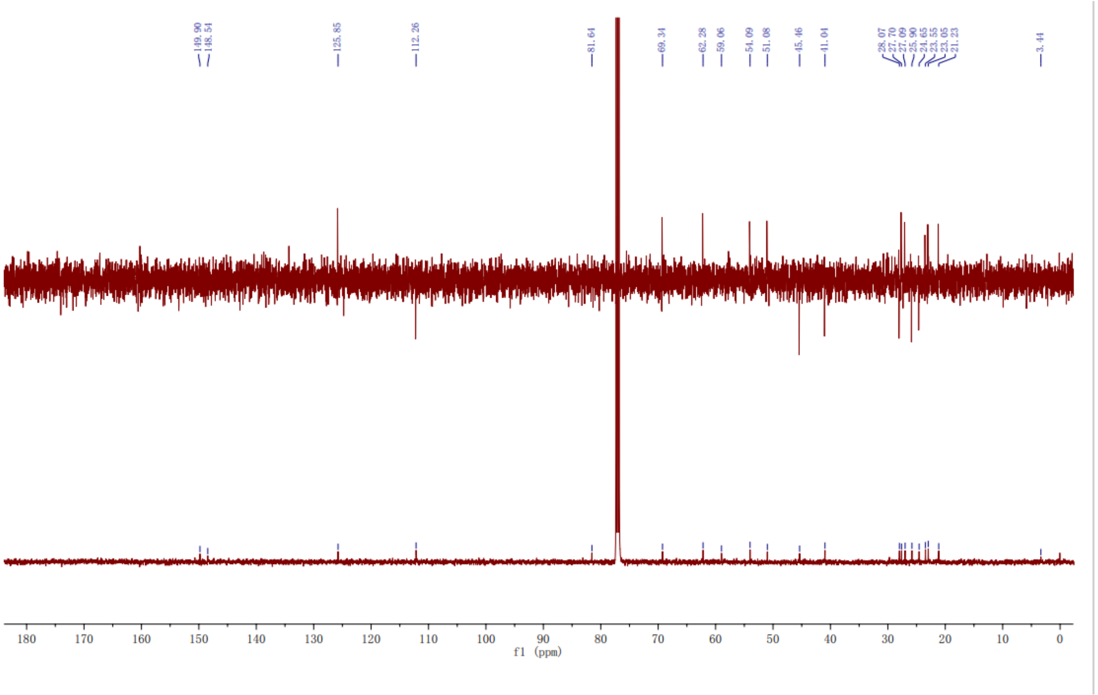

Figure S2. 13C NMR spectrum (125 MHz) of compound 1 in $CDCl_3$

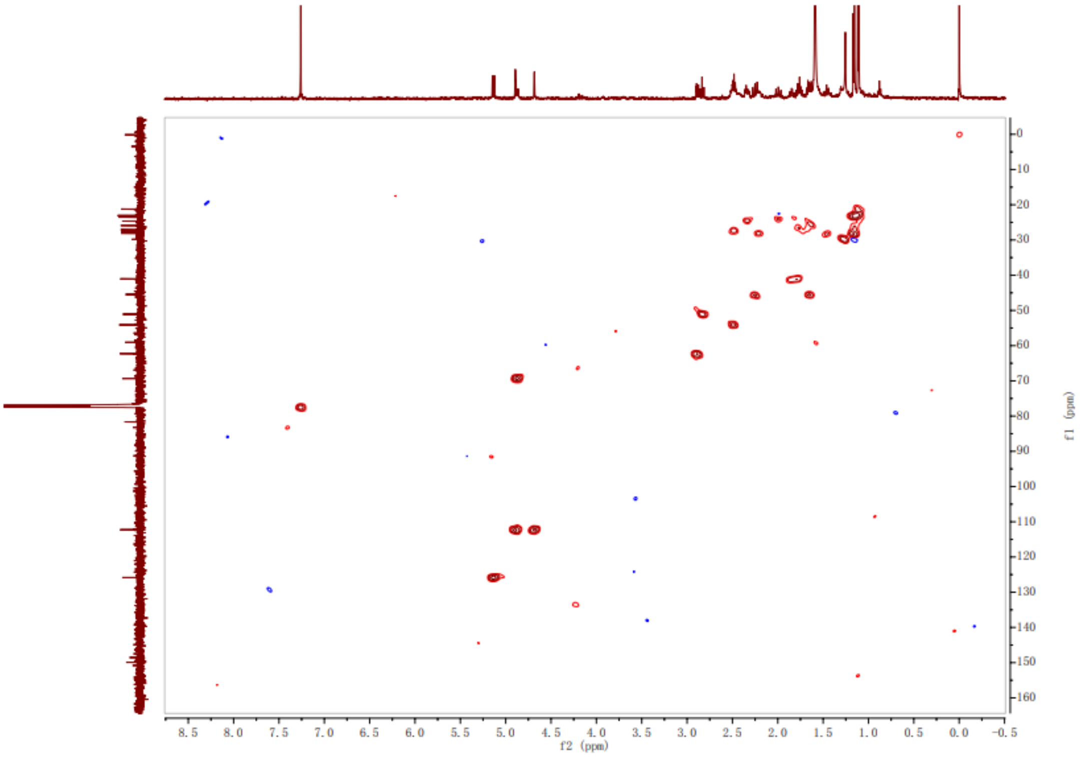

Figure S3. HSQC spectrum (500 MHz) of compound 1 in $CDCl_3$

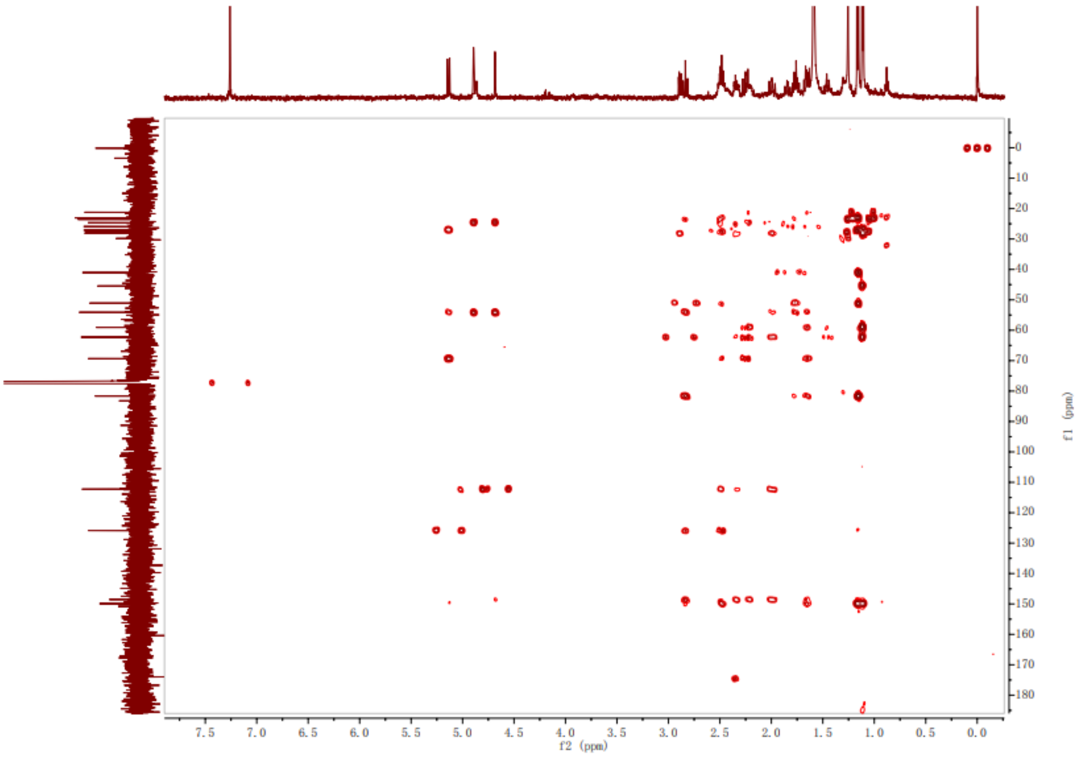

Figure S4. HMBC spectrum (500 MHz) of compound 1 in $CDCl_3$

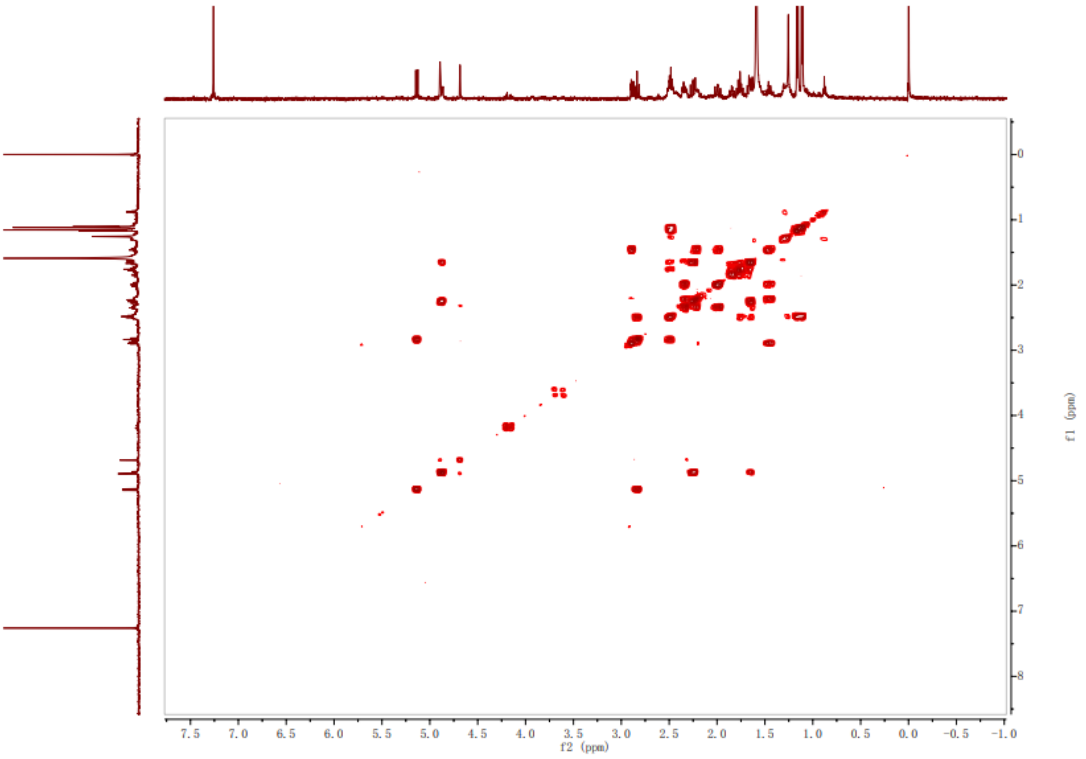

Figure S5. ^{1}H-^{1}H COSY spectrum (500 MHz) of compound 1 in $CDCl_3$

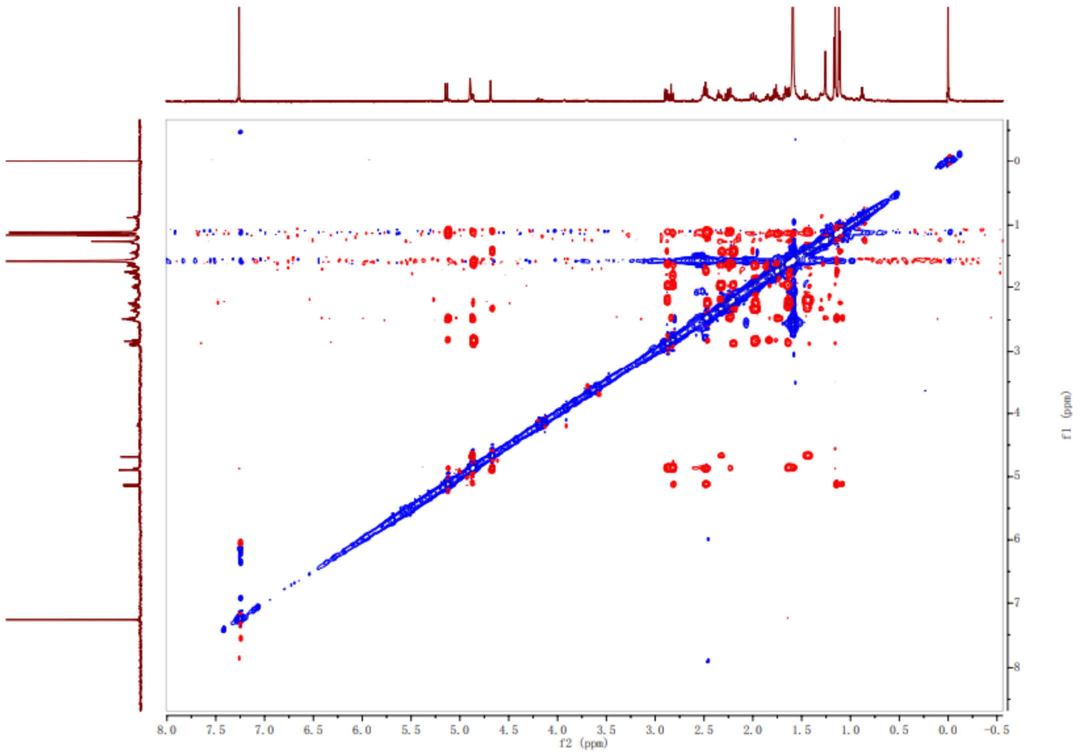

Figure S6. NOESY spectrum (500 MHz) of compound 1 in $CDCl_3$

Qualitative Analysis Report

**Data Filename**	ESIH202105812.d	**Sample Name**	A8-FCB-4
**Sample ID**		**Position**	P1-B2
**Instrument Name**	Agilent G6520 Q-TOF	**Acq Method**	20160322_MS_ESIH_POS_1min.m
**Acquired Time**	12/30/2021 15:45:49	**IRM Calibration Status**	Success
**DA Method**	small molecular data analysis method.m	**Comment**	ESIH by fangsu

User Spectra

Fragmentor Voltage	Collision Energy	Ionization Mode
175	0	ESI

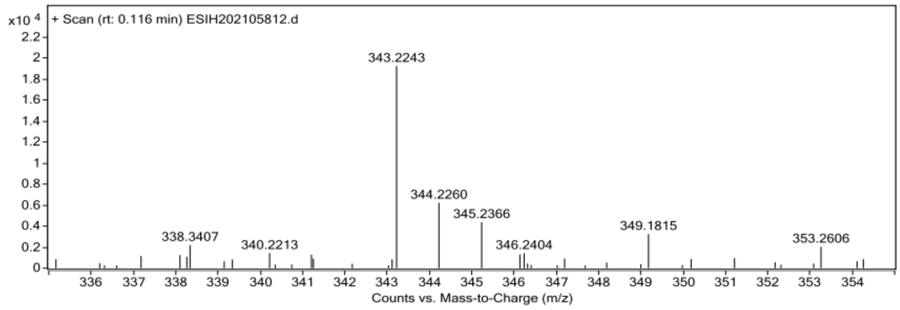

Formula Calculator Results

m/z	Calc m/z	Diff (mDa)	Diff (ppm)	Ion Formula	Ion
343.2243	343.2244	0.07	0.22	C20 H32 Na O3	(M+Na)+

--- End Of Report ---

Figure S7. HR-ESIMS of compound 1

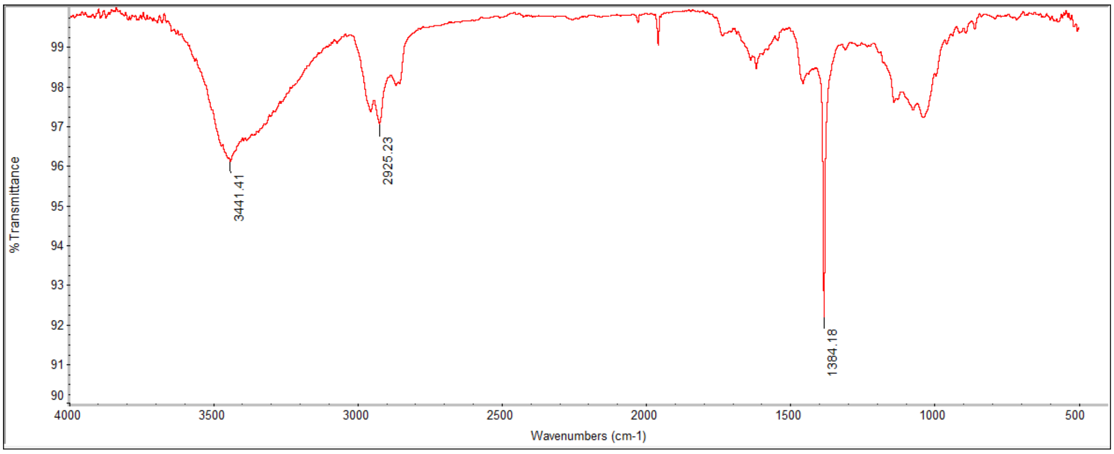

Figure S8. IR spectrum of compound 1

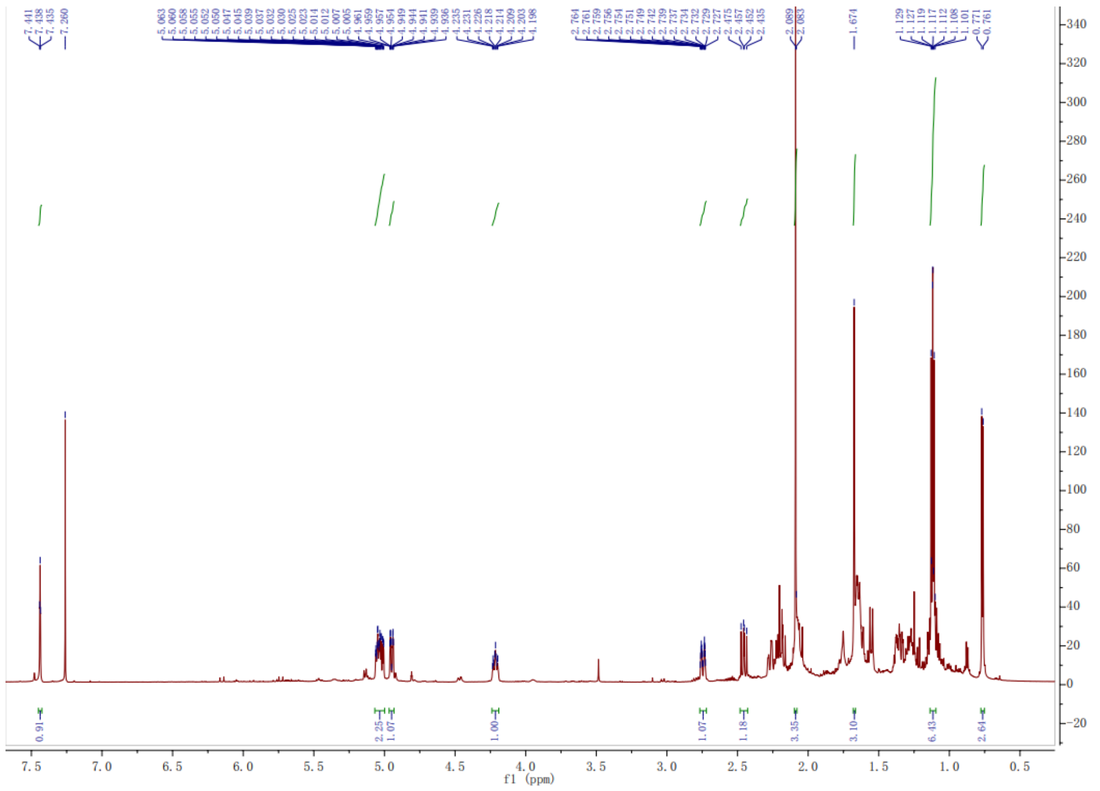

Figure S9. 1H NMR spectrum (400 MHz) of compound 3 in CDCl3

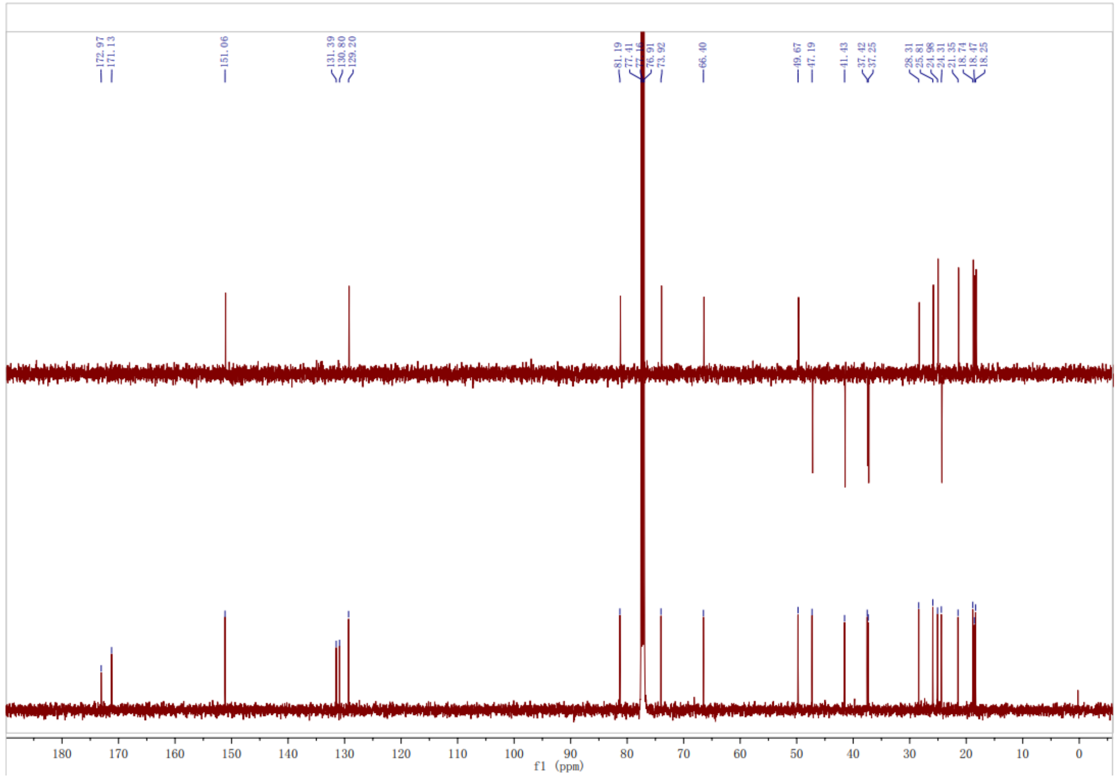

Figure S10. ^{13}C NMR spectrum (125 MHz) of compound 3 in $CDCl_3$

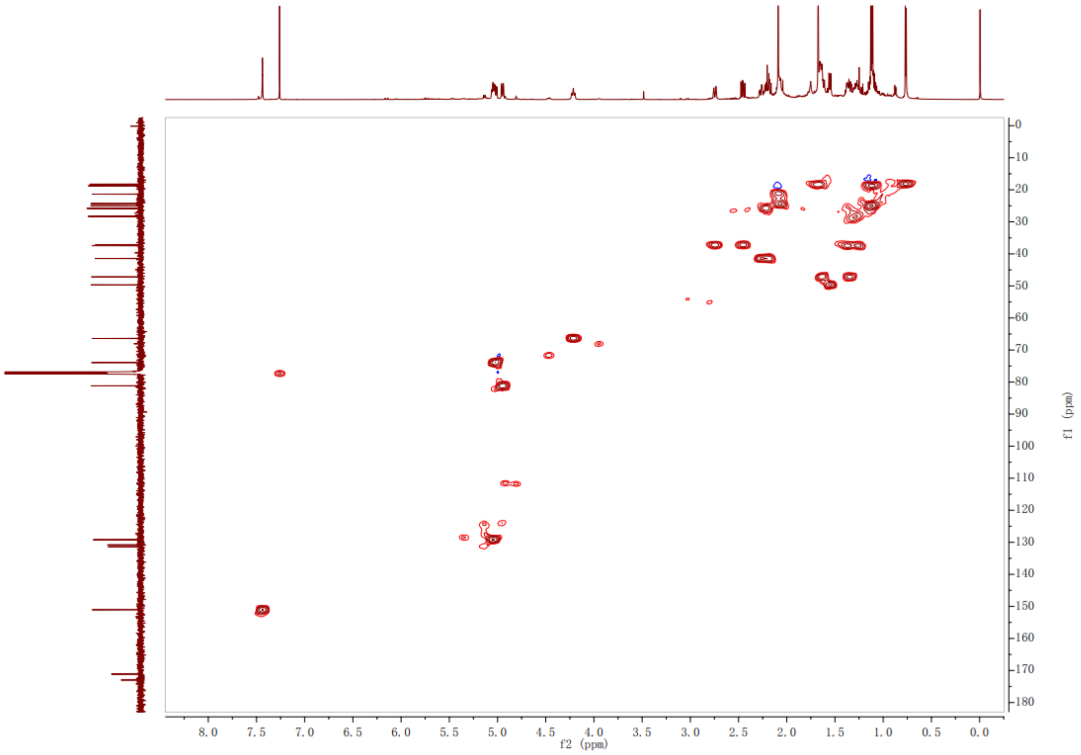

Figure S11. HSQC spectrum (500 MHz) of compound 3 in $CDCl_3$

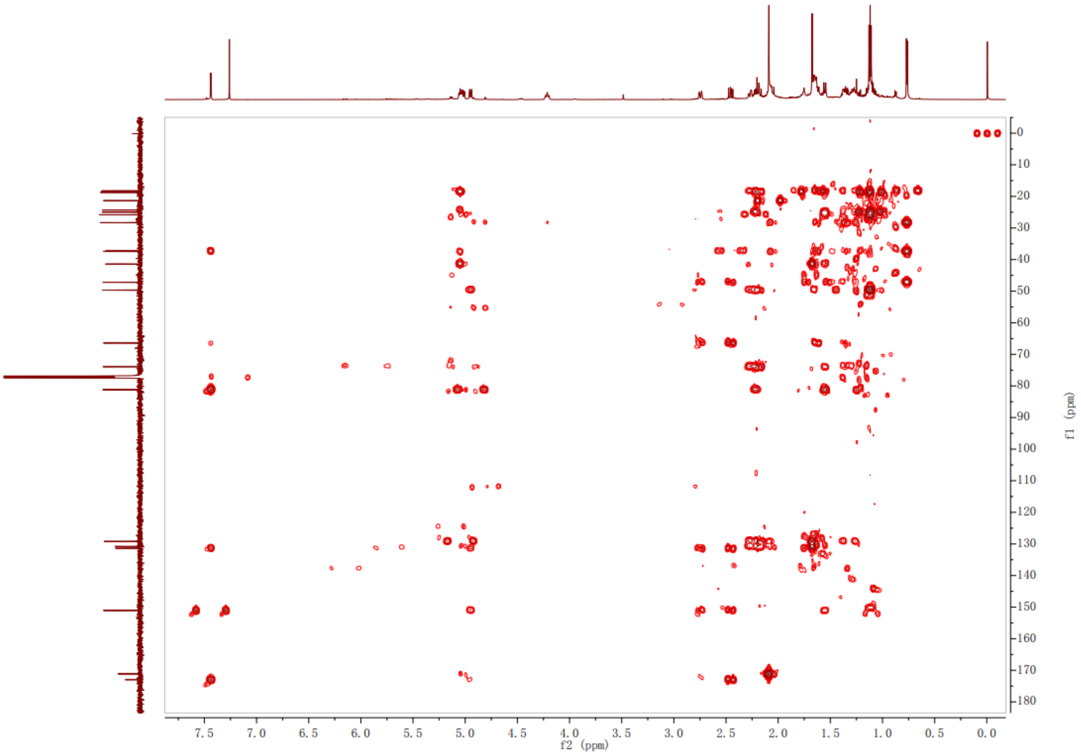

Figure S12. HMBC spectrum (500 MHz) of compound 3 in $CDCl_3$

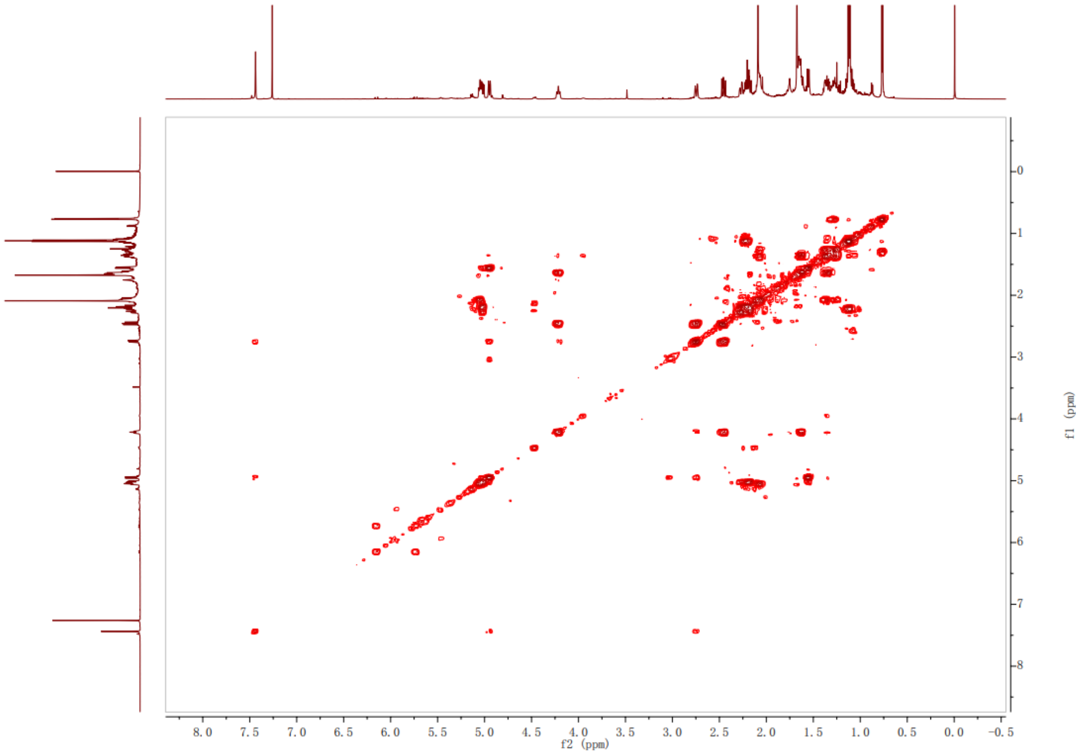

Figure S13. ^{1}H-^{1}H COSY spectrum (500 MHz) of compound 3 in $CDCl_3$

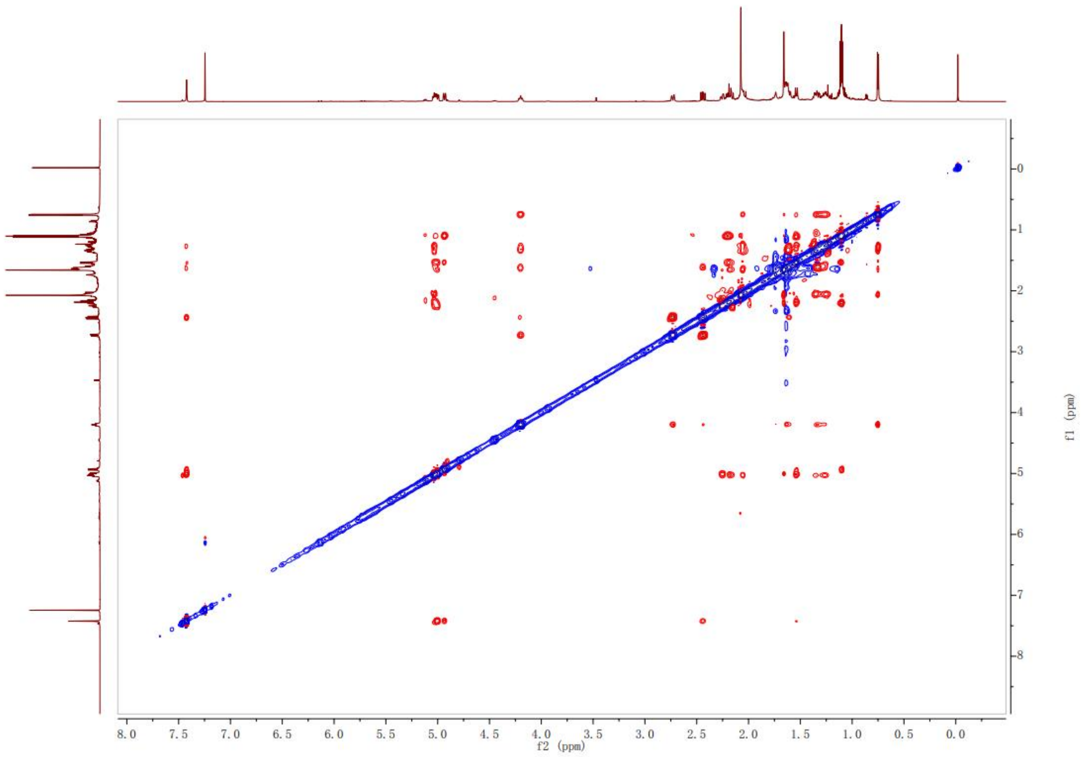

Figure S14. NOESY spectrum (500 MHz) of compound 3 in $CDCl_3$

Qualitative Analysis Report

**Data Filename**	ESIH202105814.d	**Sample Name**	A8-FDA-3
**Sample ID**		**Position**	P1-B4
**Instrument Name**	Agilent G6520 Q-TOF	**Acq Method**	20160322_MS_ESIH_POS_1min.m
**Acquired Time**	12/30/2021 14:34:46	**IRM Calibration Status**	Success
**DA Method**	small molecular data analysis method.m	**Comment**	ESIH by fangsu

User Spectra

Fragmentor Voltage	Collision Energy	Ionization Mode
175	0	ESI

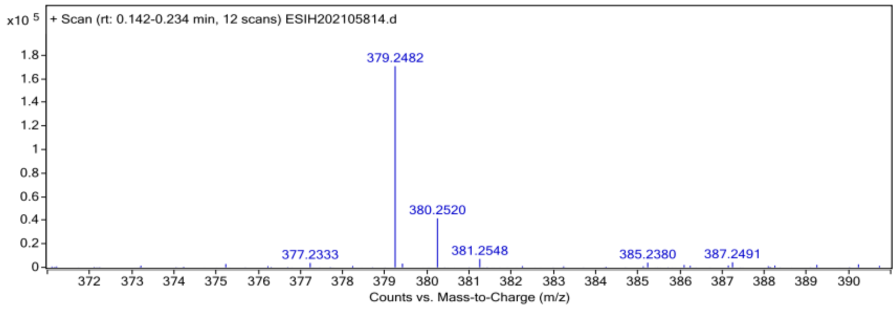

Formula Calculator Results

m/z	Calc m/z	Diff (mDa)	Diff (ppm)	Ion Formula	Ion
379.2482	379.2479	-0.3	-0.8	C22 H35 O5	(M+H)+

--- End Of Report ---

Figure S15. HR-ESIMS of compound 3

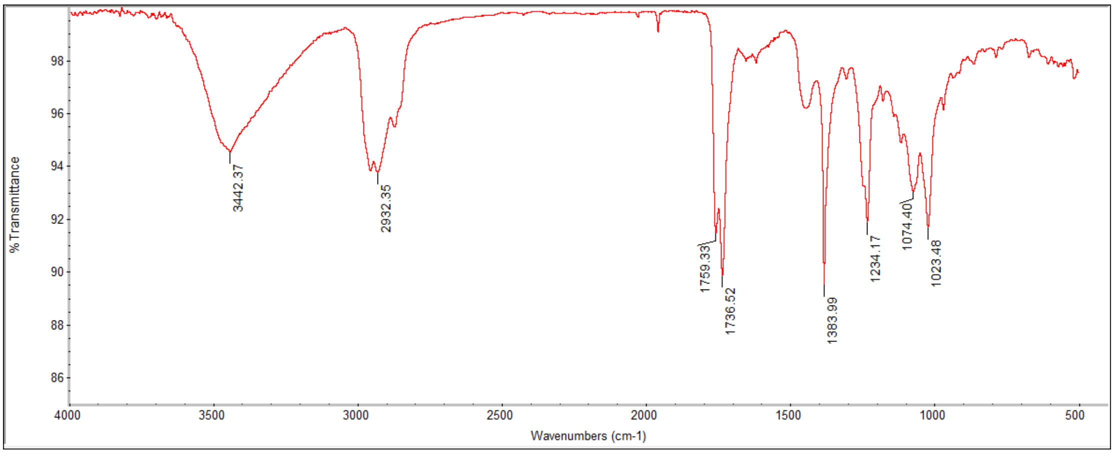

Figure S16. IR spectrum of compound 3

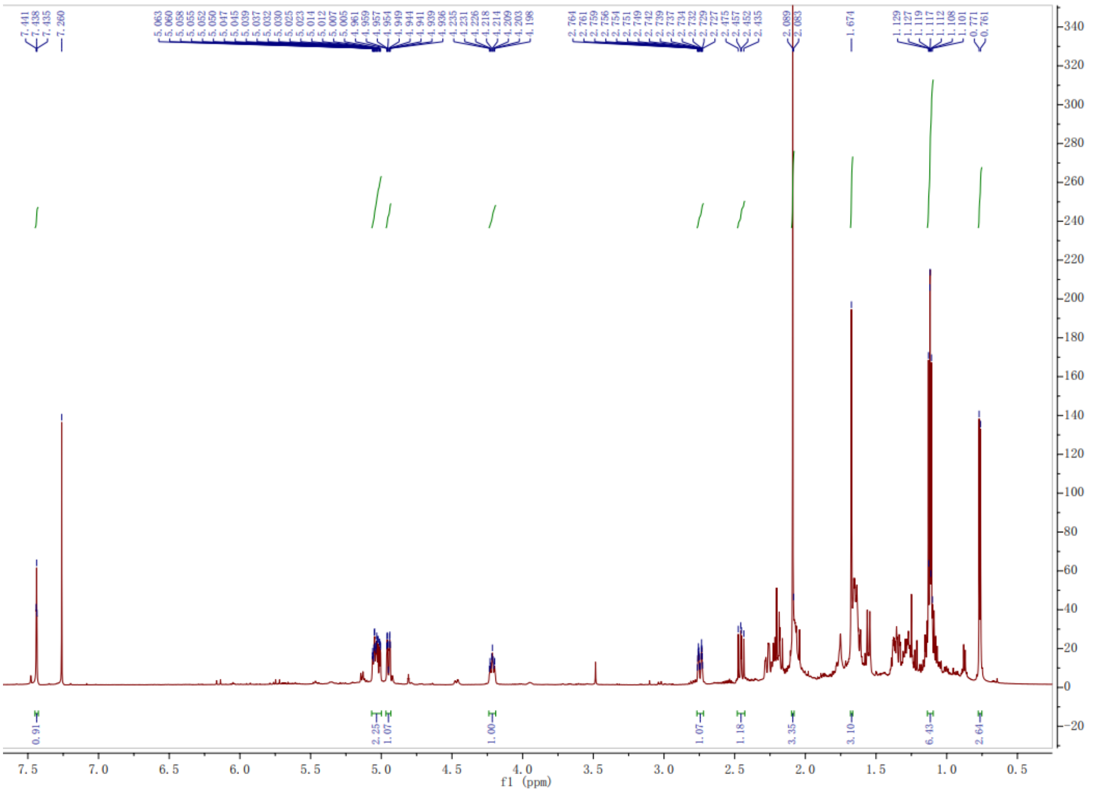

Figure S17. 1H NMR spectrum (400 MHz) of compound 5 in $CDCl_3$

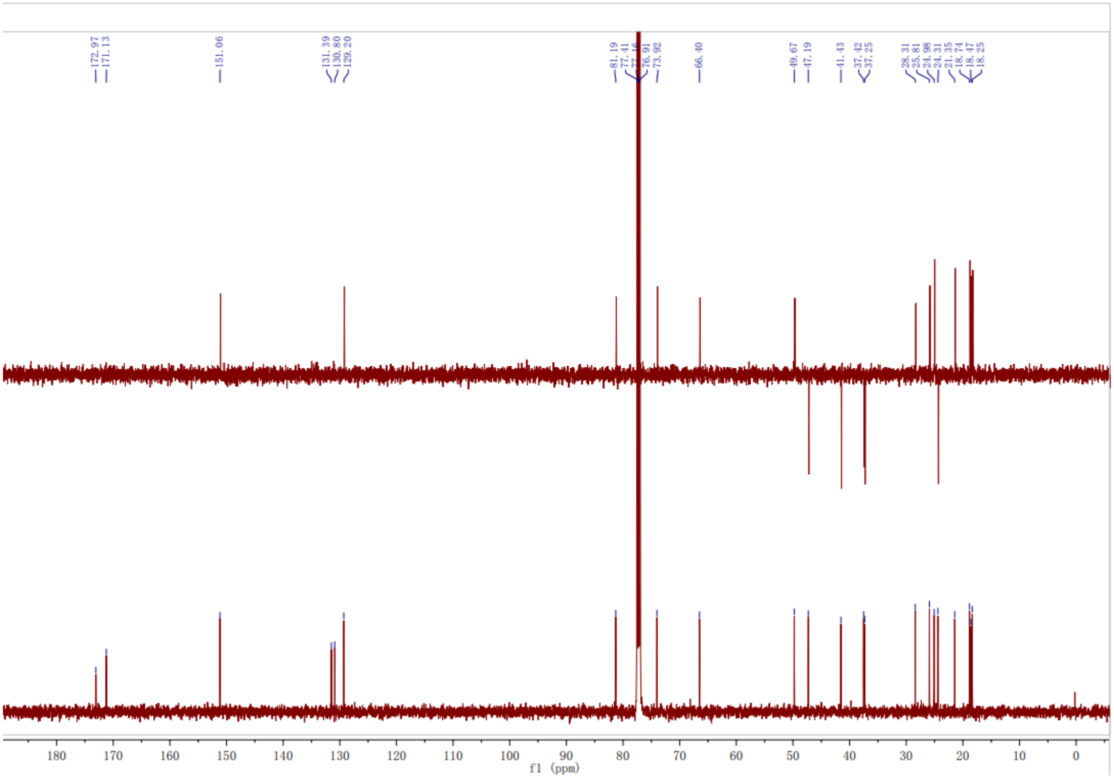

Figure S18. ^{13}C NMR spectrum (125 MHz) of compound 5 in $CDCl_3$

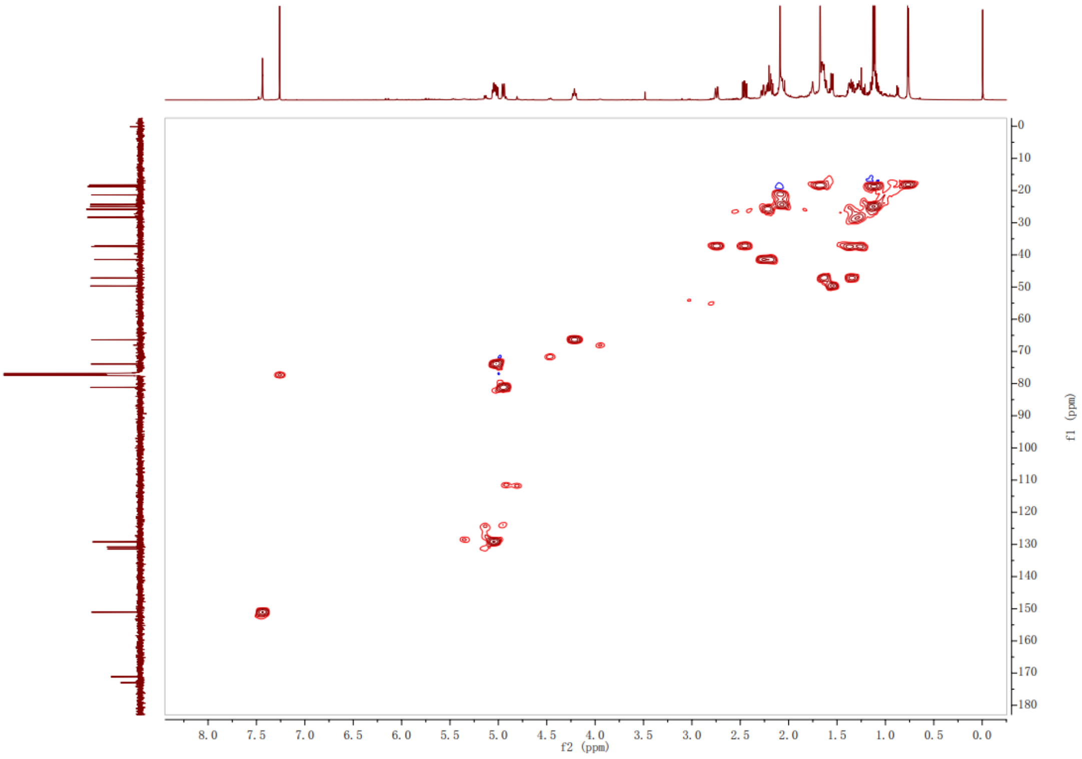

Figure S19. HSQC spectrum (500 MHz) of compound 5 in $CDCl_3$

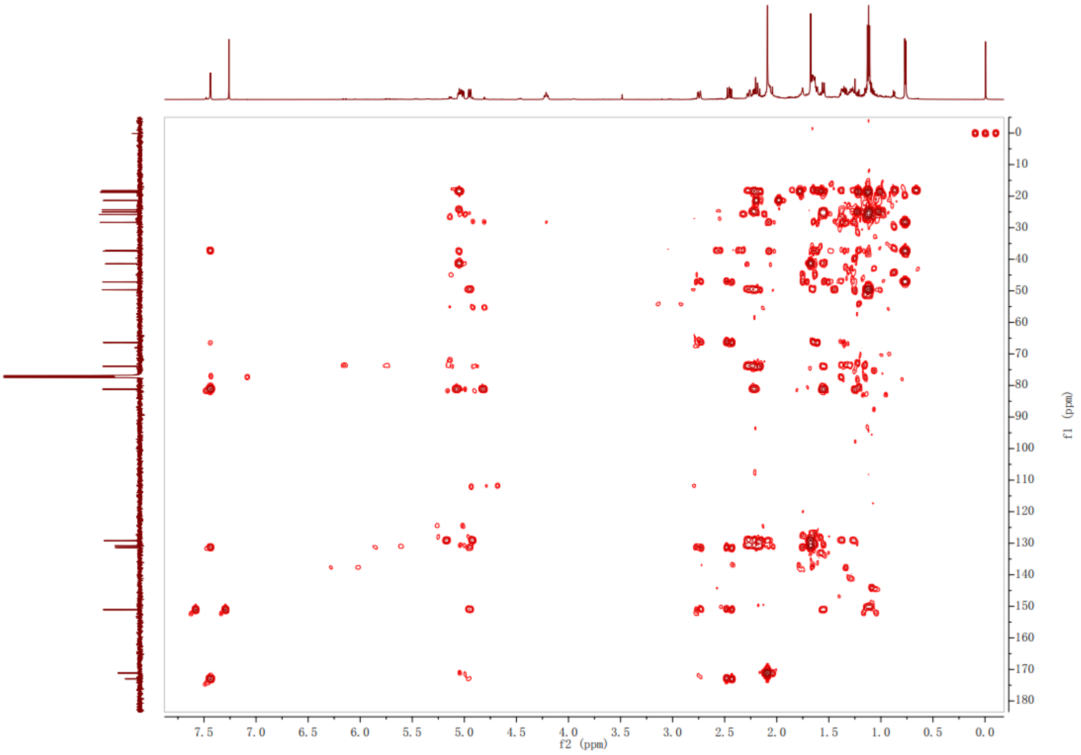

Figure S20. HMBC spectrum (500 MHz) of compound 5 in $CDCl_3$

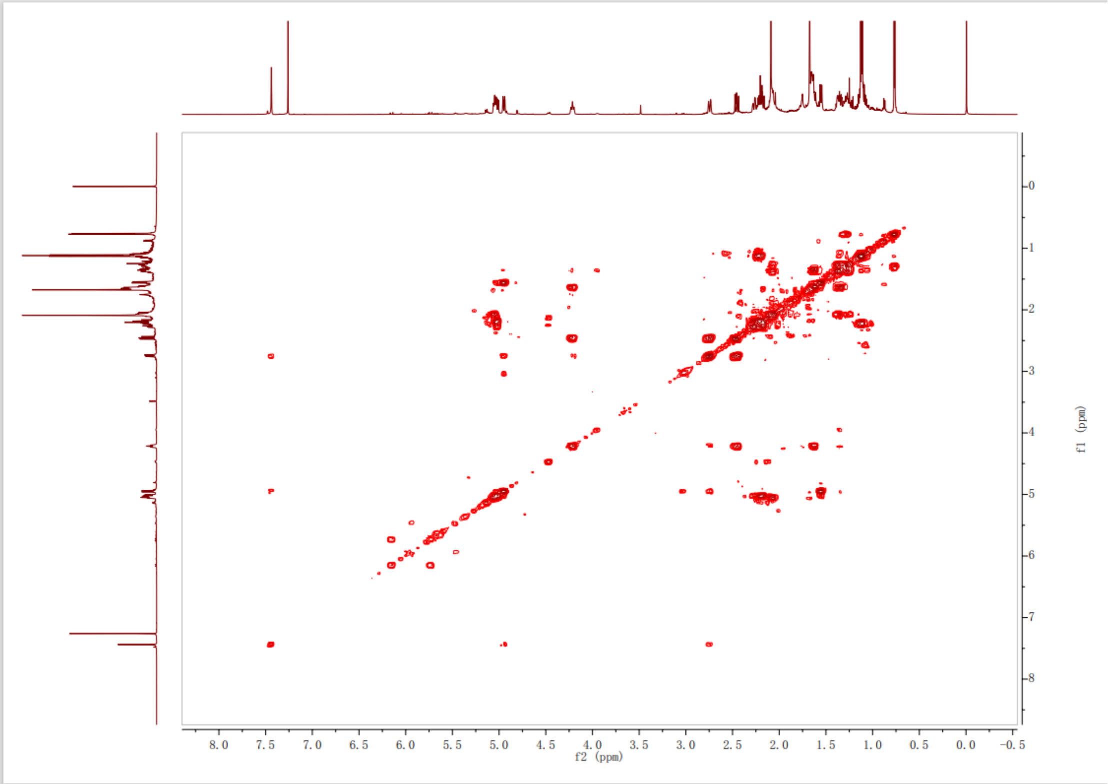

Figure S21. ^{1}H-^{1}H COSY spectrum (500 MHz) of compound 5 in $CDCl_3$

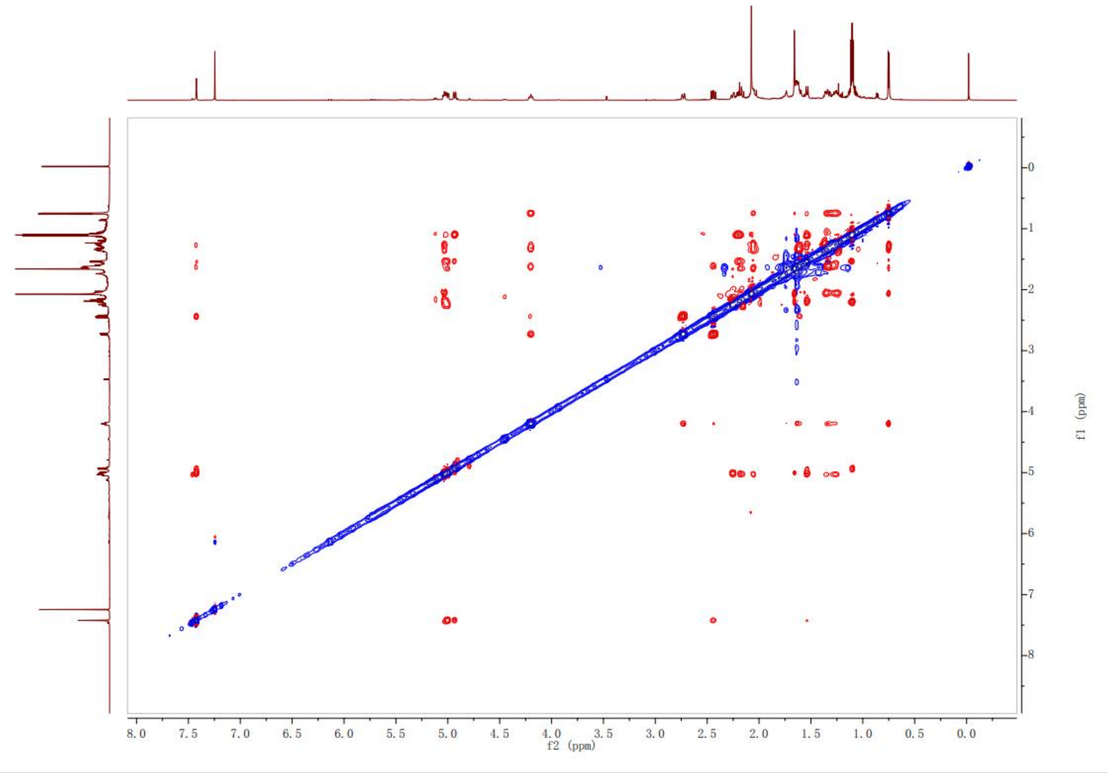

Figure S22. NOESY spectrum (500 MHz) of compound 5 in $CDCl_3$

Qualitative Analysis Report

**Data Filename**	ESIH202105115.d	**Sample Name**	A8-A8-EEA-5
**Sample ID**		**Position**	P1-A5
**Instrument Name**	Agilent G6520 Q-TOF	**Acq Method**	20160322_MS_ESIH_POS_1min.m
**Acquired Time**	11/22/2021 17:52:44	**IRM Calibration Status**	Success
**DA Method**	small molecular data analysis method.m	**Comment**	ESIH by fangsu

User Spectra

Fragmentor Voltage	Collision Energy	Ionization Mode
175	0	ESI

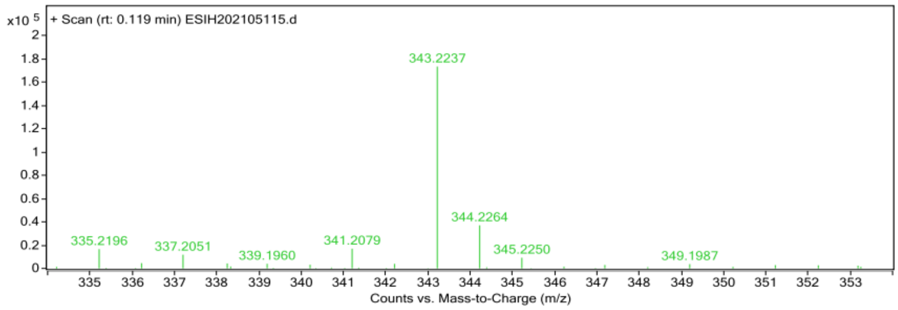

Formula Calculator Results

m/z	Calc m/z	Diff (mDa)	Diff (ppm)	Ion Formula	Ion
343.2237	343.2244	0.71	2.05	C20 H32 Na O3	(M+Na)+

--- End Of Report ---

Figure S23. HR-ESIMS of compound 5

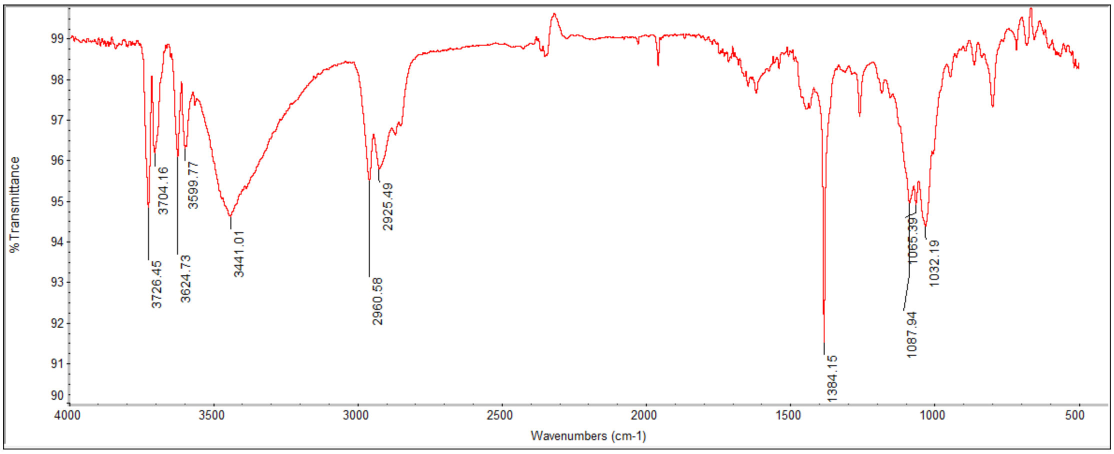

Figure S24. IR spectrum of compound 5

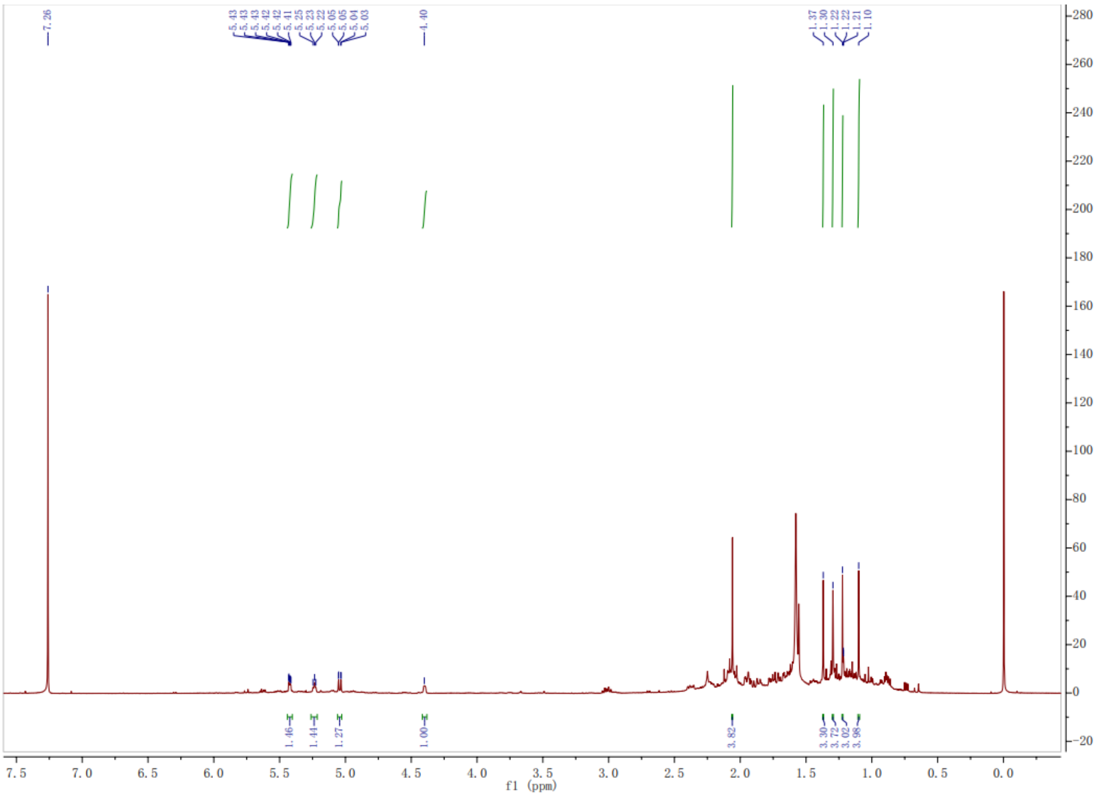

Figure S25. ^{1}H NMR spectrum (400 MHz) of compound 6 in $CDCl_3$

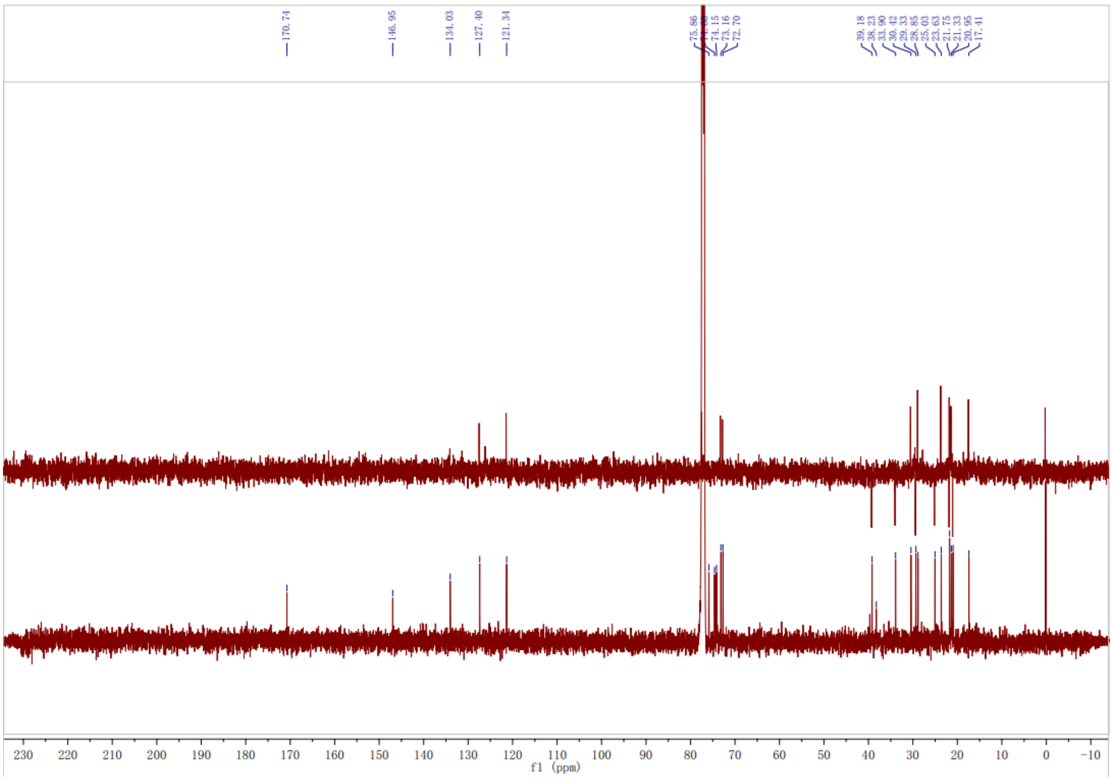

Figure S26. ^{13}C NMR spectrum (125 MHz) of compound 6 in $CDCl_3$

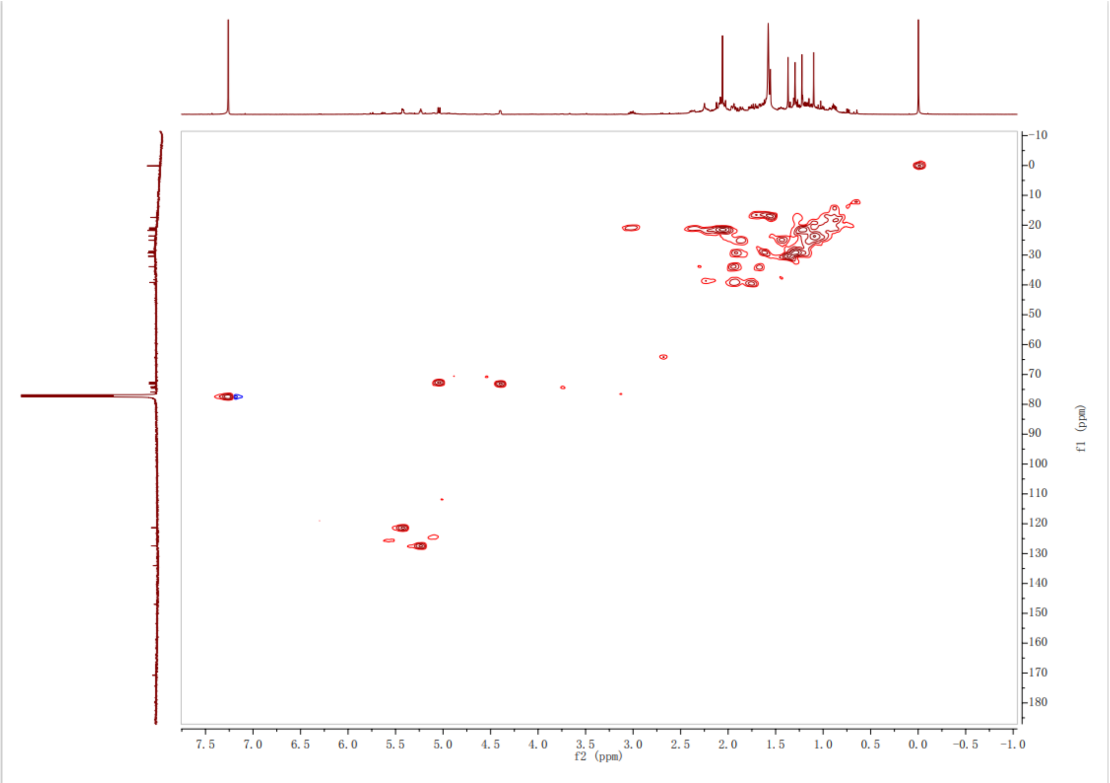

Figure S27. HSQC spectrum (500 MHz) of compound 6 in $CDCl_3$

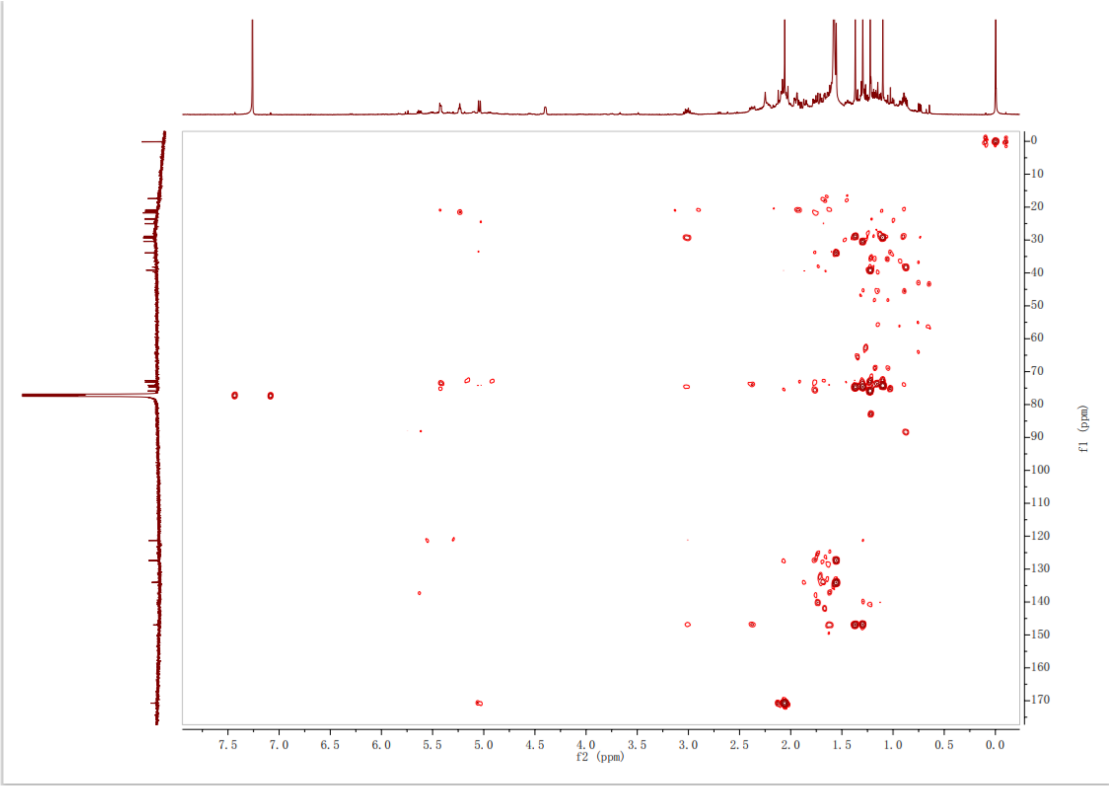

Figure S28. HMBC spectrum (500 MHz) of compound 6 in $CDCl_3$

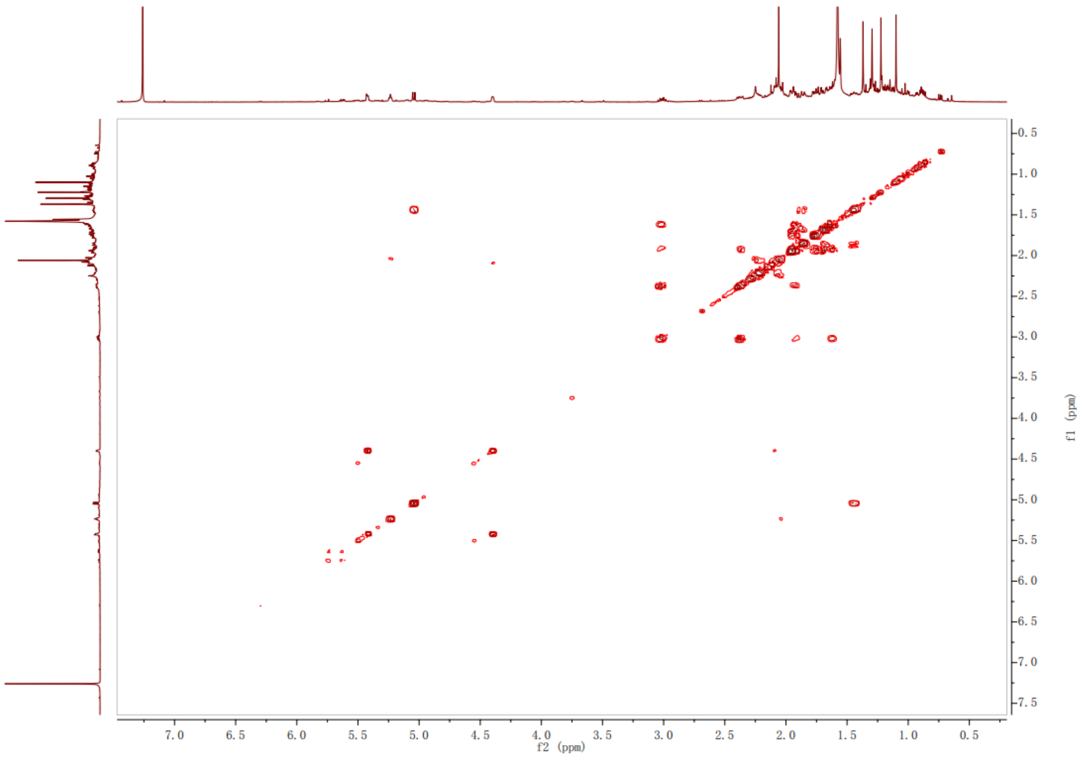

Figure S29. ^{1}H-^{1}H COSY spectrum (500 MHz) of compound 6 in $CDCl_3$

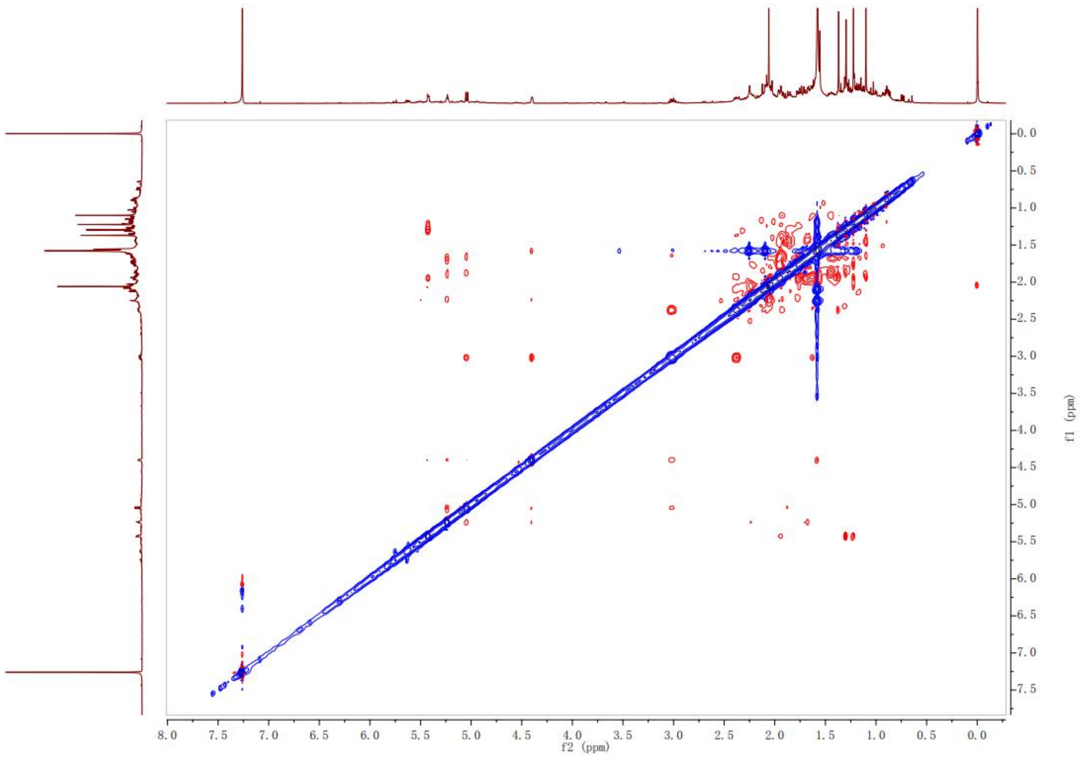

Figure S30. NOESY spectrum (500 MHz) of compound 6 in $CDCl_3$

Qualitative Analysis Report

**Data Filename**	ESIH202105273-1.d	**Sample Name**	A8-FCA-10
**Sample ID**		**Position**	P1-D9
**Instrument Name**	Agilent G6520 Q-TOF	**Acq Method**	20160322_MS_ESIH_POS_1min.m
**Acquired Time**	12/3/2021 18:22:56	**IRM Calibration Status**	Success
**DA Method**	small molecular data analysis method.m	**Comment**	ESIH by fangsu

User Spectra

Fragmentor Voltage	Collision Energy	Ionization Mode
175	0	ESI

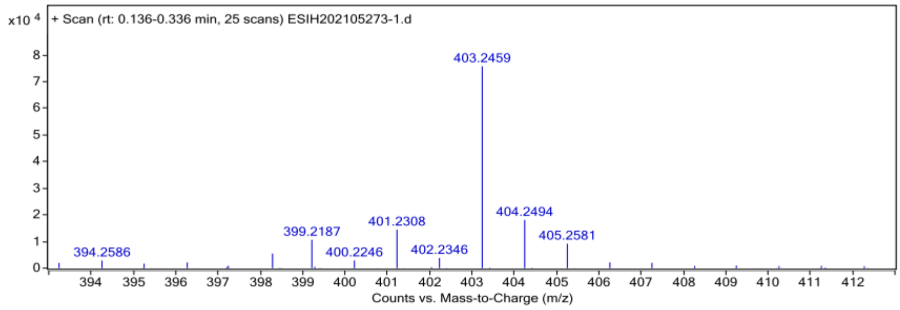

Formula Calculator Results

m/z	Calc m/z	Diff (mDa)	Diff (ppm)	Ion Formula	Ion
403.2459	403.2455	-0.4	-1	C22 H36 Na O5	(M+Na)+

--- End Of Report ---

Figure S31. HR-ESIMS of compound 6

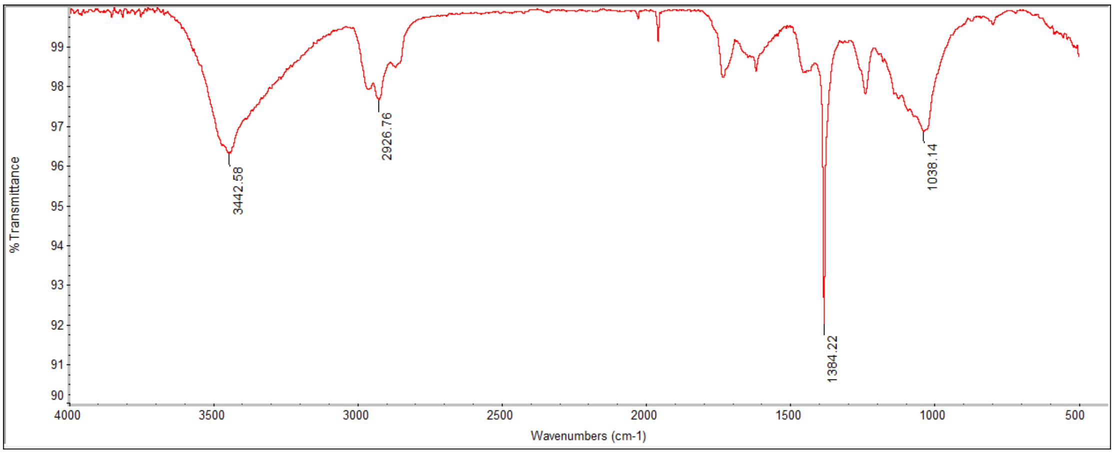

Figure S32. IR spectrum of compound 6

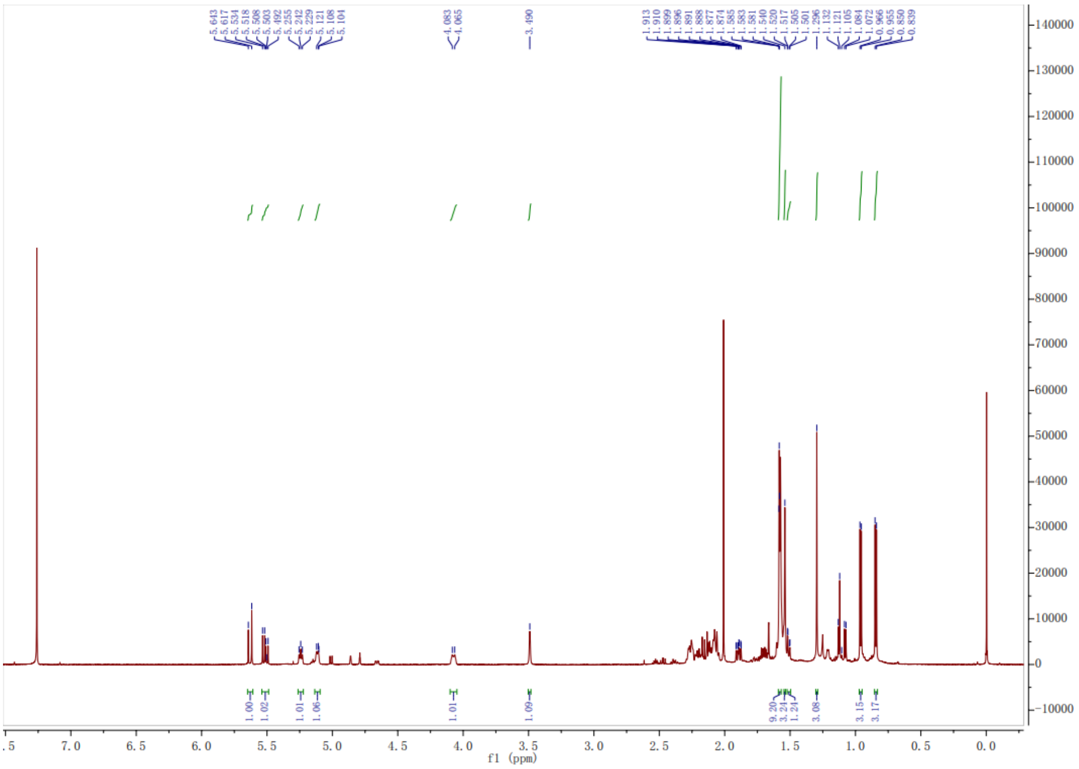

Figure S33. ^{1}H NMR spectrum (400 MHz) of compound 7 in $CDCl_3$

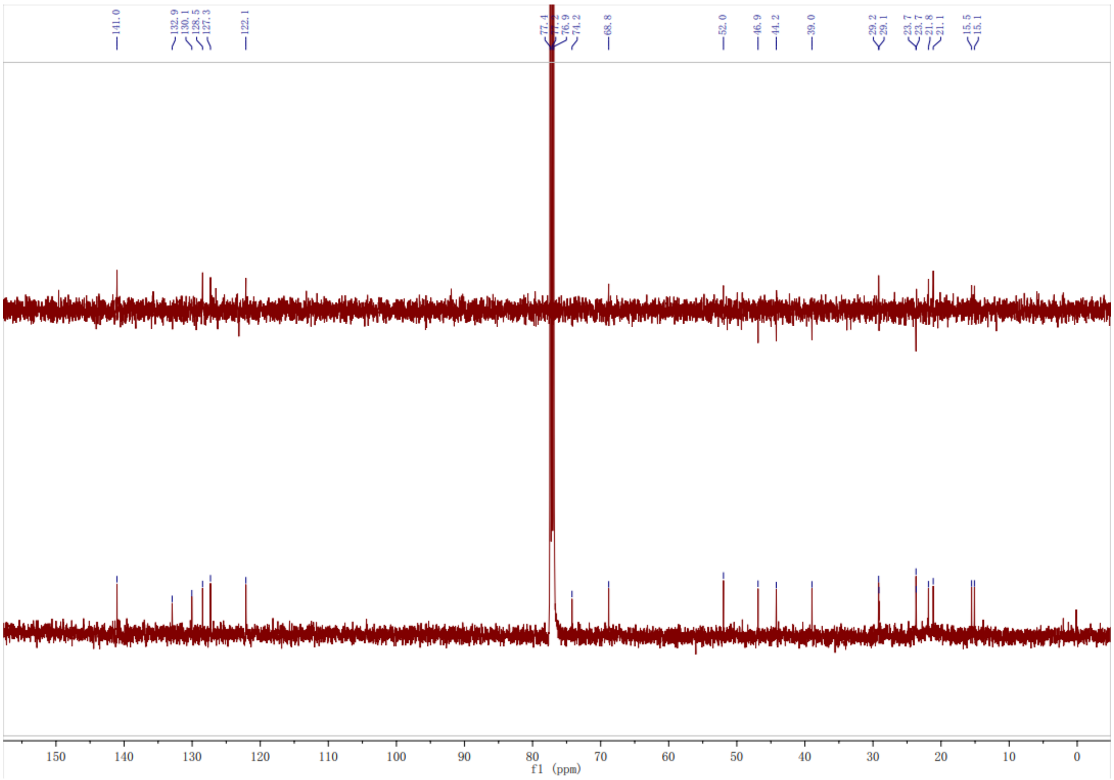

Figure S34. ^{13}C NMR spectrum (125 MHz) of compound 7 in $CDCl_3$

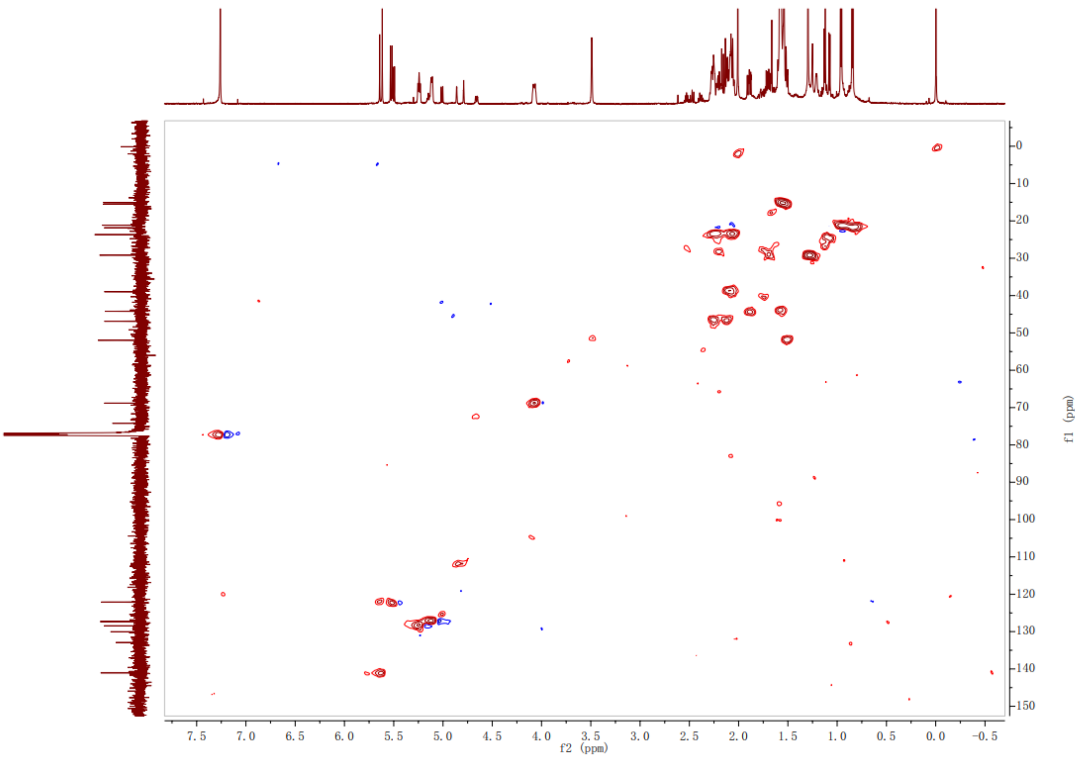

Figure S35. HSQC spectrum (500 MHz) of compound 7 in $CDCl_3$

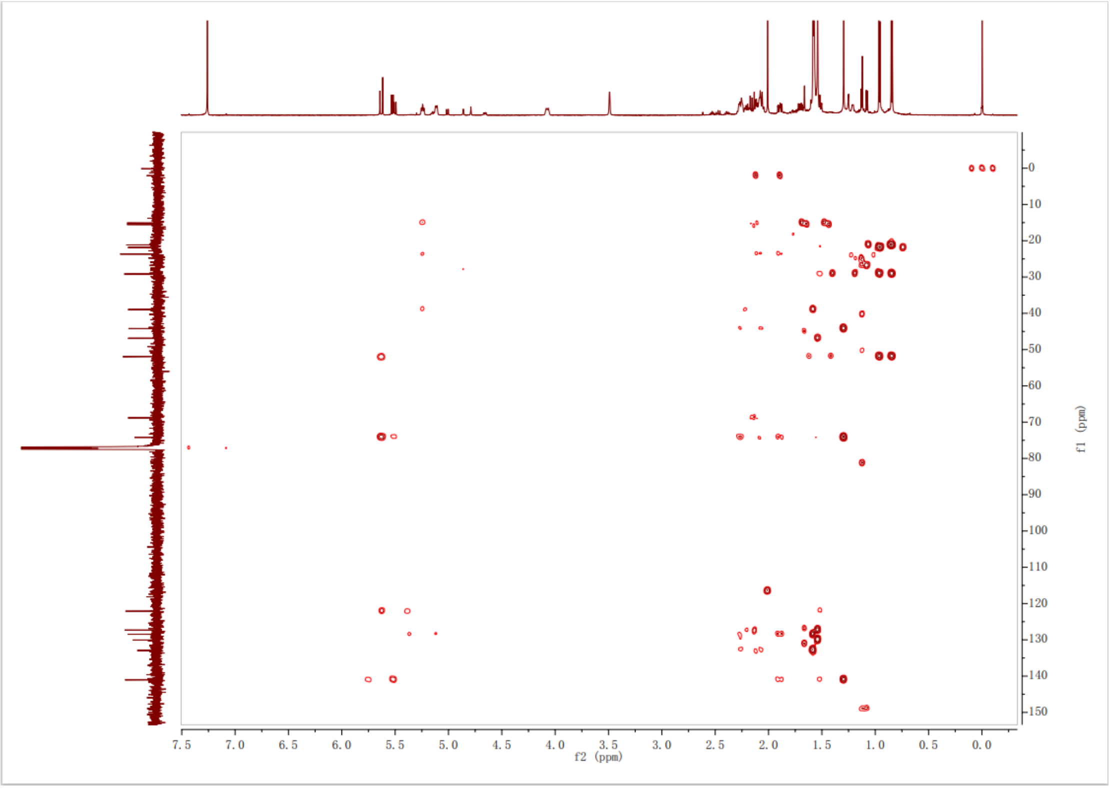

Figure S36. HMBC spectrum (500 MHz) of compound 7 in $CDCl_3$

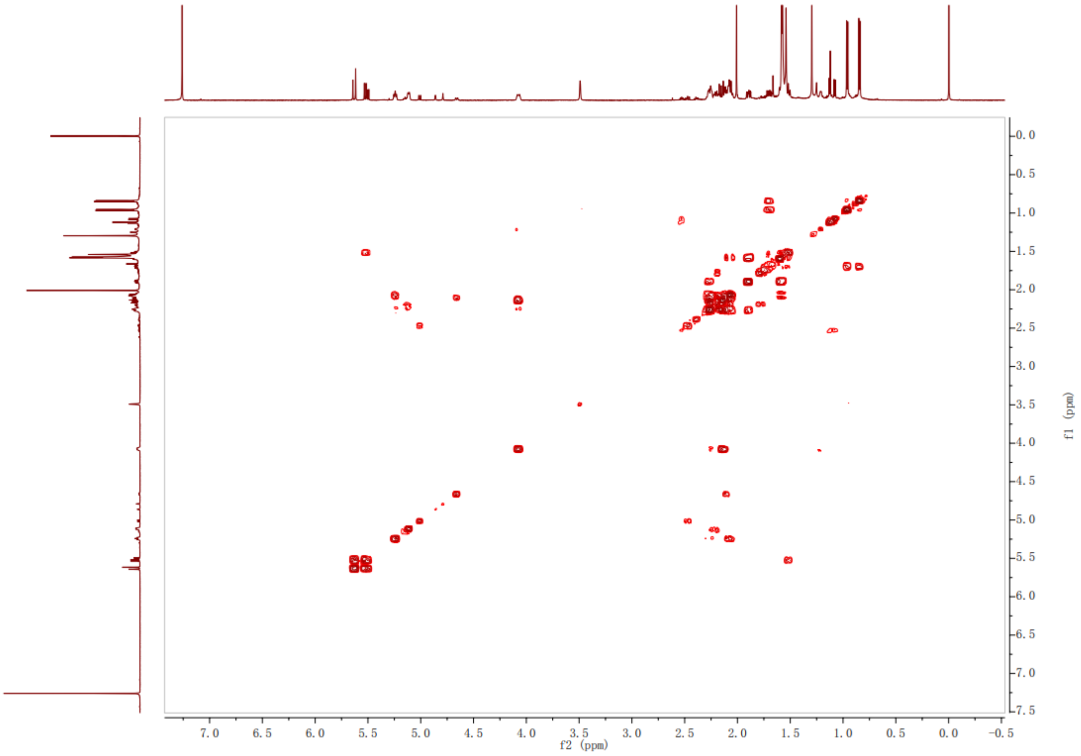

Figure S37. ^{1}H-^{1}H COSY spectrum (500 MHz) of compound 7 in $CDCl_3$

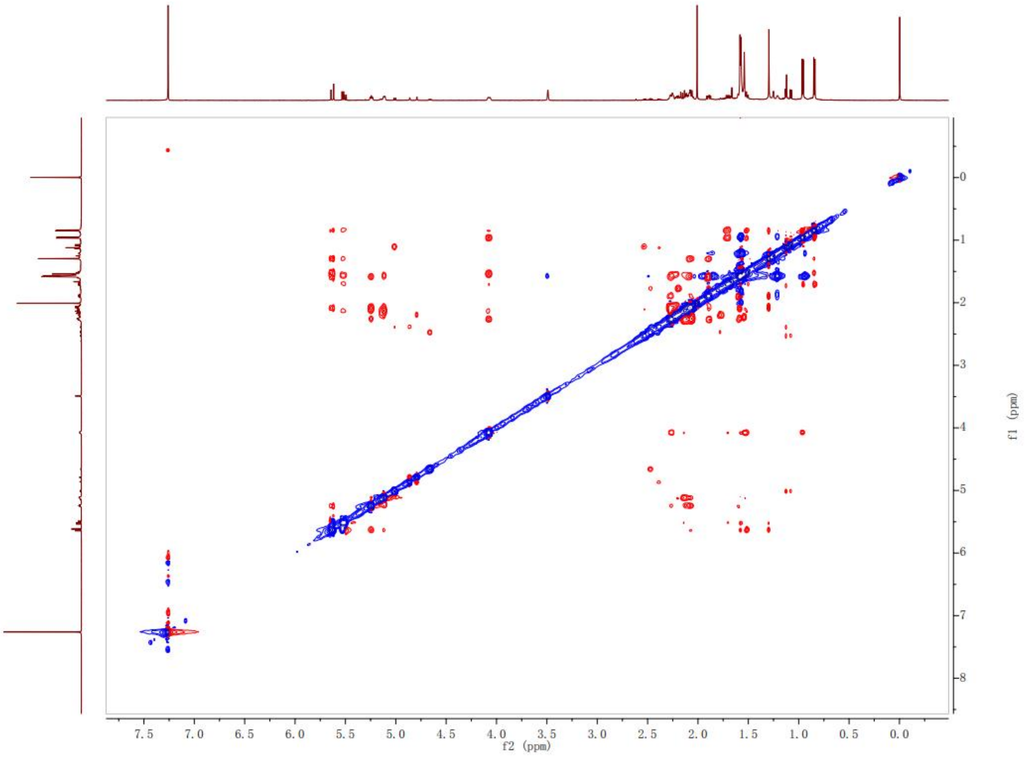

Figure S38. NOESY spectrum (500 MHz) of compound 7 in $CDCl_3$

Qualitative Analysis Report

**Data Filename**	ESIH202105813.d	**Sample Name**	A8-FCB-7
**Sample ID**		**Position**	P1-B3
**Instrument Name**	Agilent G6520 Q-TOF	**Acq Method**	20160322_MS_ESIH_POS_1min.m
**Acquired Time**	12/30/2021 14:33:30	**IRM Calibration Status**	Success
**DA Method**	small molecular data analysis method.m	**Comment**	ESIH by fangsu

User Spectra

Fragmentor Voltage	Collision Energy	Ionization Mode
175	0	ESI

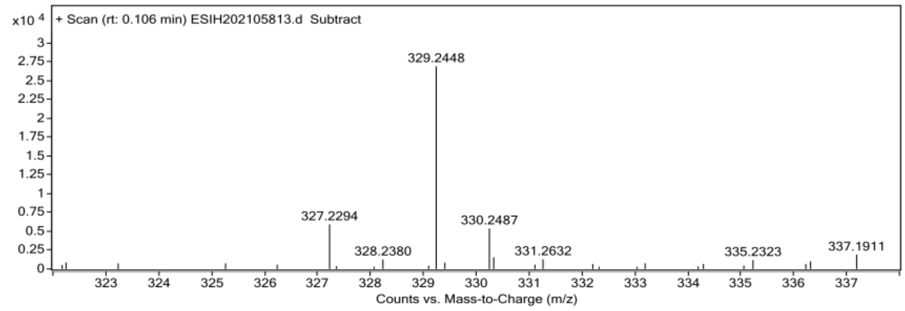

Formula Calculator Results

m/z	Calc m/z	Diff (mDa)	Diff (ppm)	Ion Formula	Ion
329.2448	329.2451	0.34	1.02	C20 H34 Na O2	(M+Na)+

--- End Of Report ---

Figure S39. HR-ESIMS of compound 7

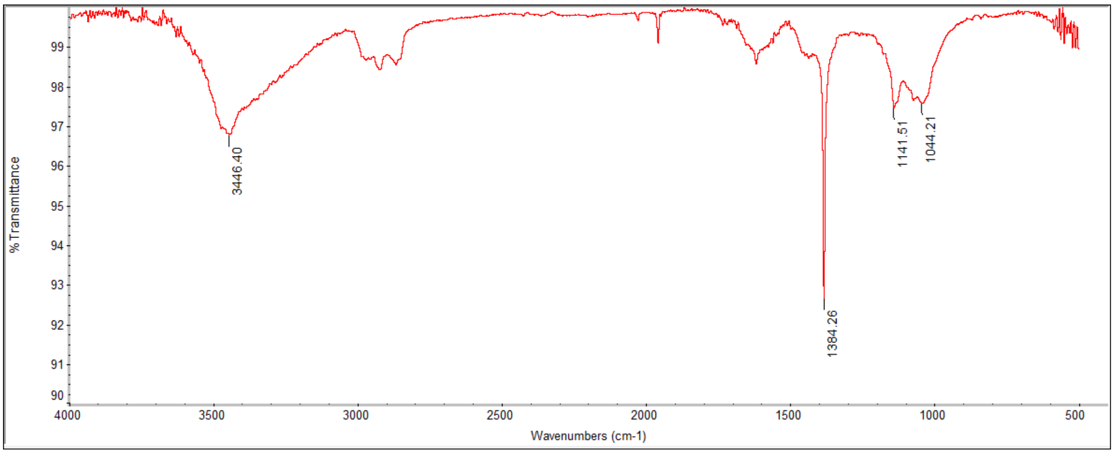

Figure S40. IR spectrum of compound 7

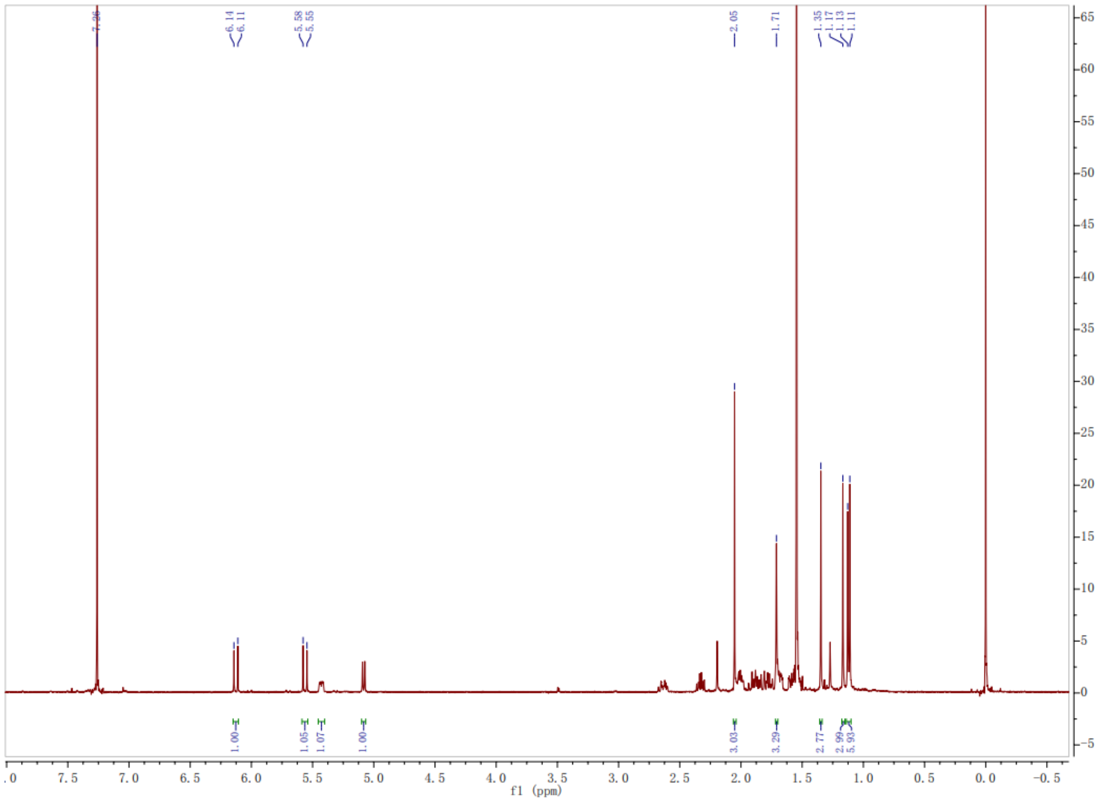

Figure S41. ^{1}H NMR spectrum (400 MHz) of compound 8 in $CDCl_3$

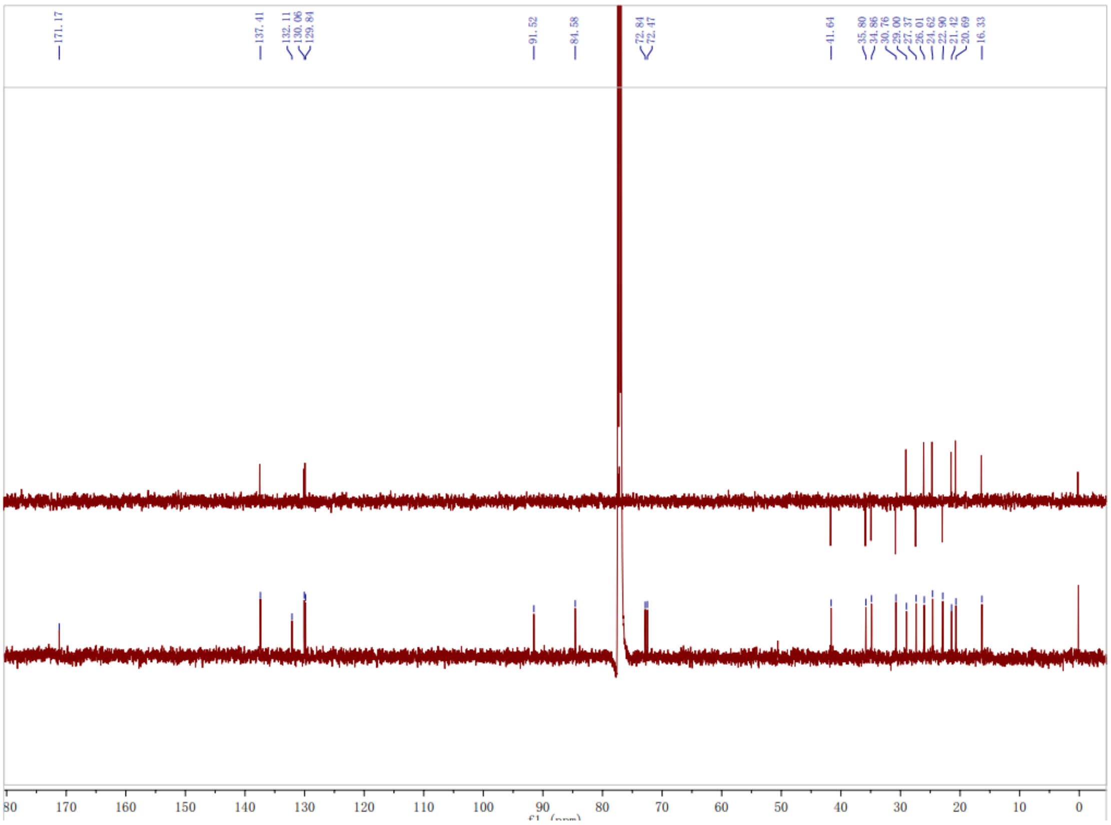

Figure S42. 13C NMR spectrum (125 MHz) of compound 8 in $CDCl_3$

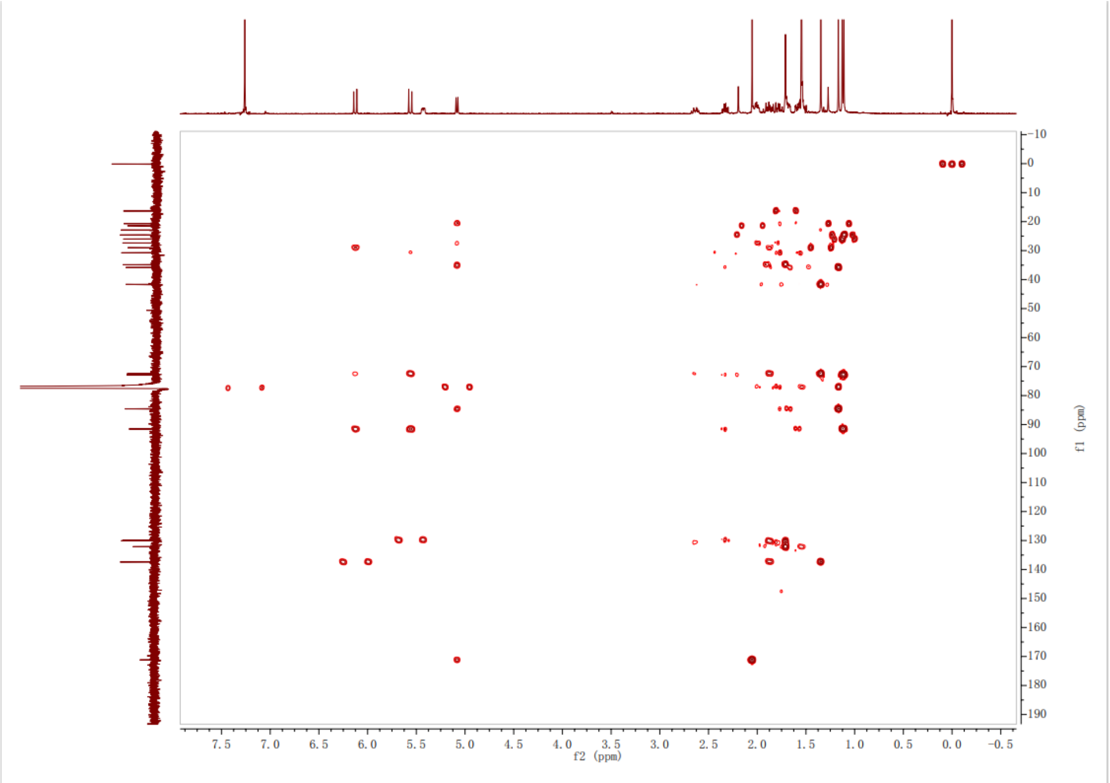

Figure S43. HSQC spectrum (500 MHz) of compound 8 in $CDCl_3$

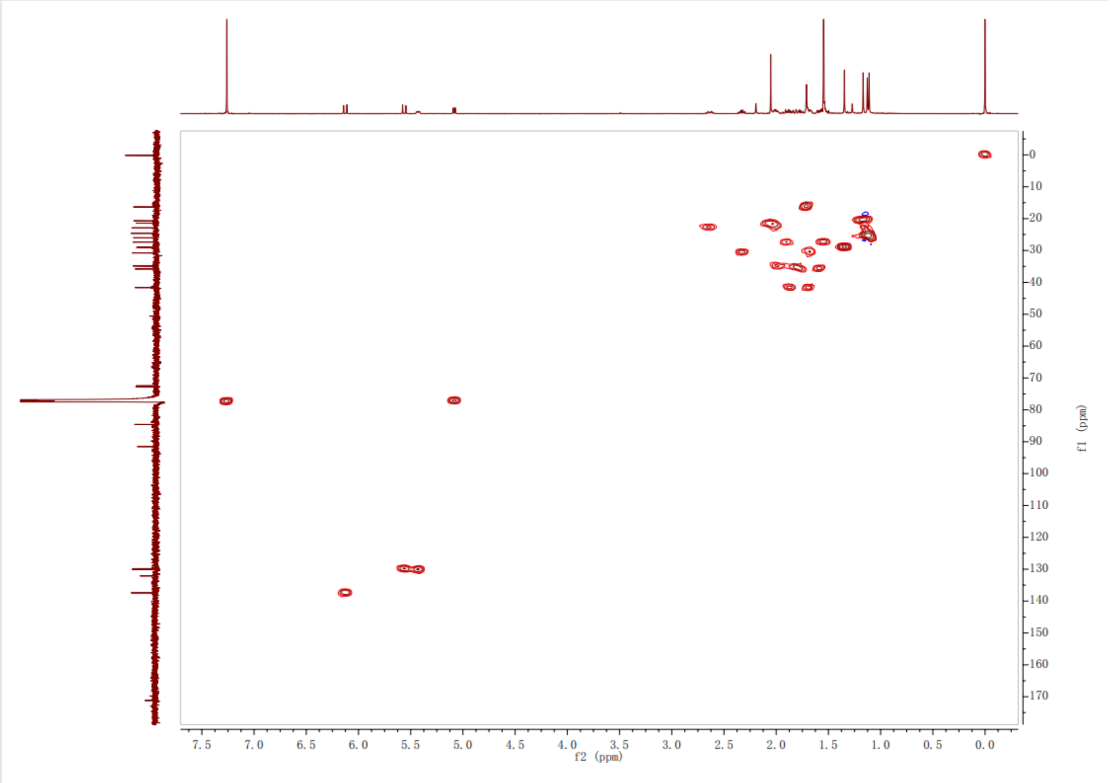

Figure S44. HMBC spectrum (500 MHz) of compound 8 in $CDCl_3$

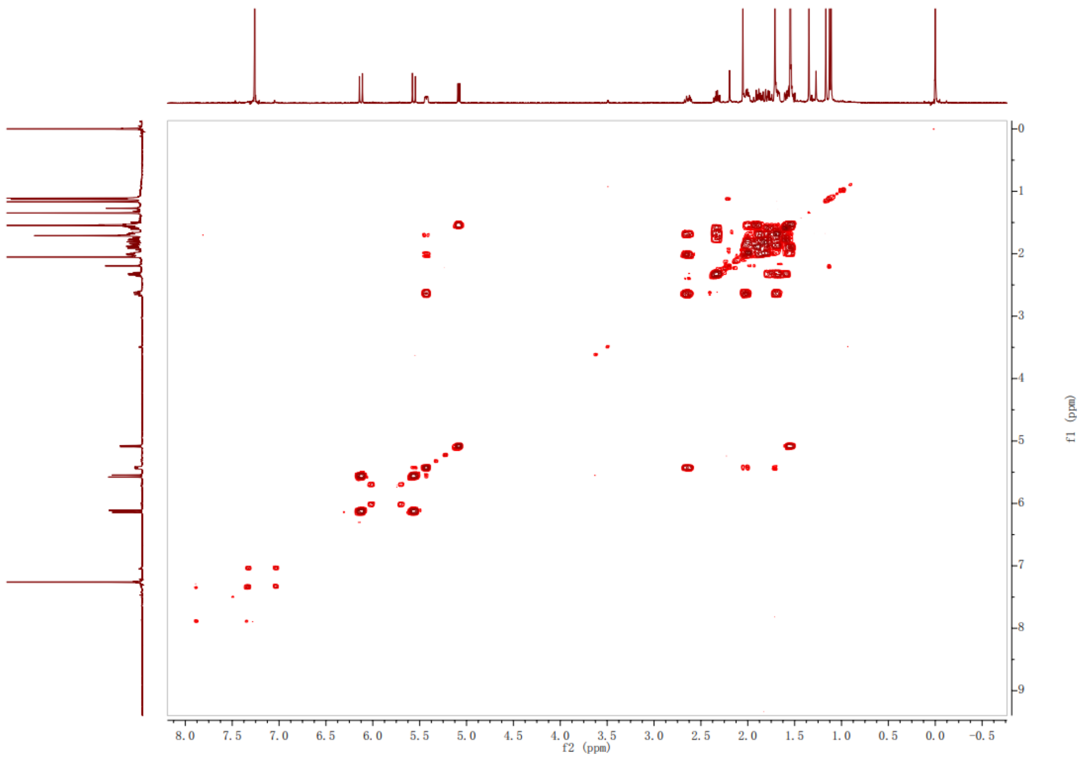

Figure S45. ^{1}H-^{1}H COSY spectrum (500 MHz) of compound 8 in $CDCl_3$

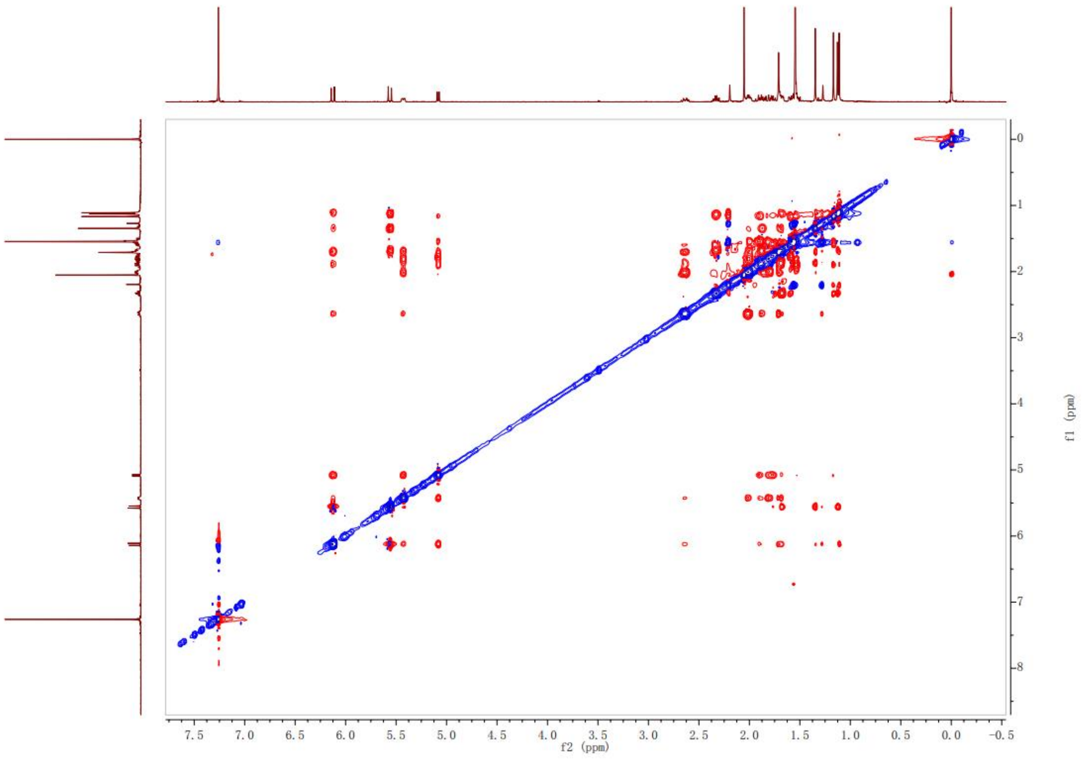

Figure S46. NOESY spectrum (500 MHz) of compound 8 in $CDCl_3$

Qualitative Analysis Report

**Data Filename**	ESIH202202457.d	**Sample Name**	A8-FCA-4-2
**Sample ID**		**Position**	P1-A8
**Instrument Name**	Agilent G6520 Q-TOF	**Acq Method**	20160322_MS_ESIH_POS_1min.m
**Acquired Time**	7/22/2022 14:23:25	**IRM Calibration Status**	Success
**DA Method**	small molecular data analysis method.m	**Comment**	ESIH by fangsu

User Spectra

Fragmentor Voltage	Collision Energy	Ionization Mode
175	0	ESI

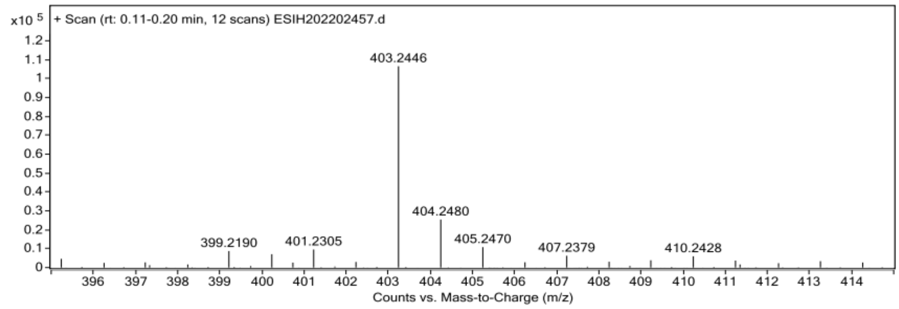

Formula Calculator Results

m/z	Calc m/z	Diff (mDa)	Diff (ppm)	Ion Formula	Ion
403.2446	403.2455	0.92	2.27	C22 H36 Na O5	(M+Na)+

--- End Of Report ---

Figure S47. HR-ESIMS of compound 8

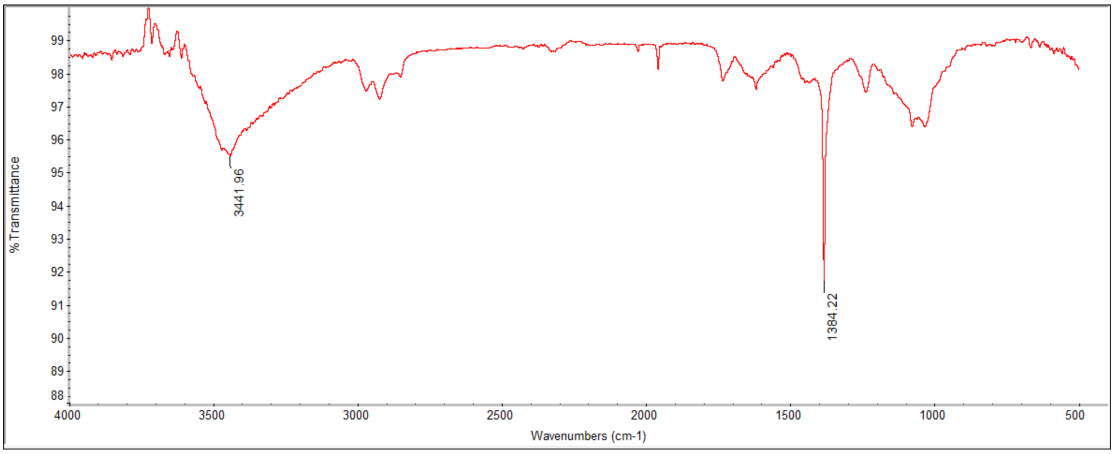

Figure S48. IR spectrum of compound 8

Table S1. X-ray crystallographic data for compound 1

Empirical formula	$C_{20}H_{34}O_4$
Formula weight	338.47
Temperature/K	170.0
Crystal system	monoclinic
Space group	$P2_1$
a/Å	9.3012(3)
b/Å	24.6100(8)
c/Å	9.6893(3)
α/°	90
β/°	117.9940(10)
γ/°	90
Volume/Å^3	1958.40(11)
Z	4
ρ_{calc}g/cm^3	1.148
μ/mm^{-1}	0.620
F (000)	744.0
Crystal size/mm^3	0.19*0.08*0.07
Radiation	Cu Kα (λ = 1.54178)
2Θ range for data collection/°	10.34 to 149.606
Index ranges	-11 ≤ h ≤ 11, -30 ≤ k ≤ 30, -11 ≤ l ≤ 12
Reflections collected	26834
Independent reflections	7843 [R_{int} = 0.0395, R_{sigma} = 0.0351]
Data/restraints/parameters	7843/1/470
Goodness-of-fit on F^2	1.036
Final R indexes [I>=2σ (I)]	R_1 = 0.0336, wR_2 = 0.0772
Final R indexes [all data]	R_1 = 0.0359, wR_2 = 0.0796
Largest diff. peak/hole / e Å^{-3}	0.20/-0.15
Flack parameter	-0.05(6)

Table S2. X-ray crystallographic data for compound 4

Empirical formula	$C_{22}H_{32}O_5$
Formula weight	376.47
Temperature/K	170.0
Crystal system	tetragonal
Space group	$I4_1$
a/Å	23.0912(7)
b/Å	23.0912(7)
c/Å	8.3133(4)
α/°	90
β/°	90

γ/°	90
Volume/Å^3	4432.7(3)
Z	8
ρ_{calc}g/cm^3	1.128
μ/mm^{-1}	0.635
F (000)	1632.0
Crystal size/mm^3	0.12*0.08*0.05
Radiation	Cu Kα (λ = 1.54178)
2Θ range for data collection/°	5.412 to 149.152
Index ranges	-27 ≤ h ≤ 27, -27 ≤ k ≤ 20, -10 ≤ l ≤ 10
Reflections collected	11788
Independent reflections	4302 [R_{int} = 0.0409, R_{sigma} = 0.0369]
Data/restraints/parameters	4302/1/260
Goodness-of-fit on F^2	1.050
Final R indexes [I>=2σ (I)]	R_1 = 0.0378, wR_2 = 0.0943
Final R indexes [all data]	R_1 = 0.0418, wR_2 = 0.0980
Largest diff. peak/hole / e Å^{-3}	0.36/-0.24
Flack parameter	-0.03 (9)

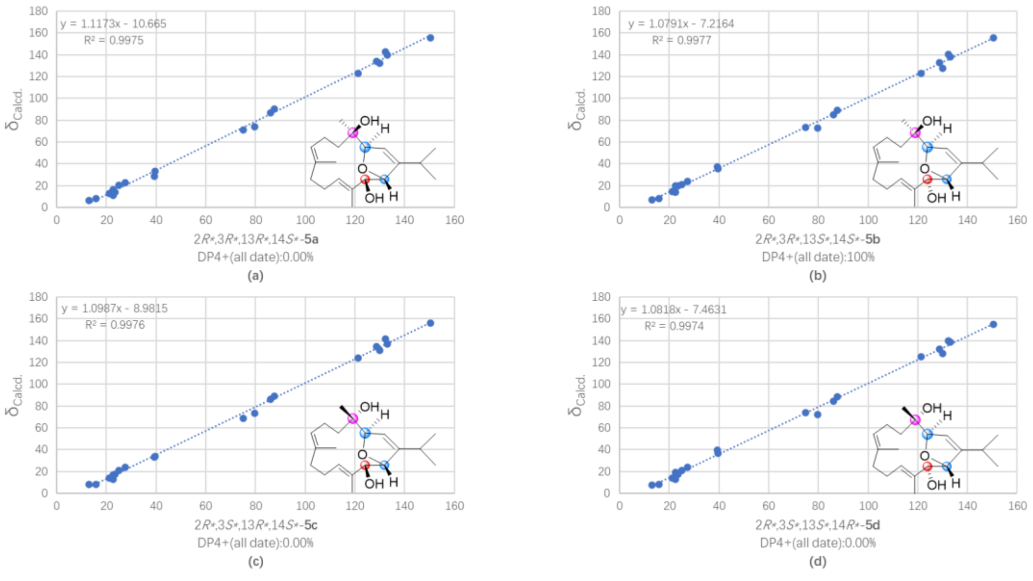

Figure S49. Regression analysis of experimental vs calculated ^{13}C NMR chemical shifts of (2*R**, 3*R**, 13*R**, 14*S**)-**5a**, (2*R**, 3*R**, 13*S**, 14*S**)-**5b**, (2*R**, 3*S**, 13*R**, 14*S**)-**5c**, (2*R**, 3*S**, 13*S**, 14*R**)-**5d** at the PCM/mPW1PW91/6-31+G** level using DP4+ method

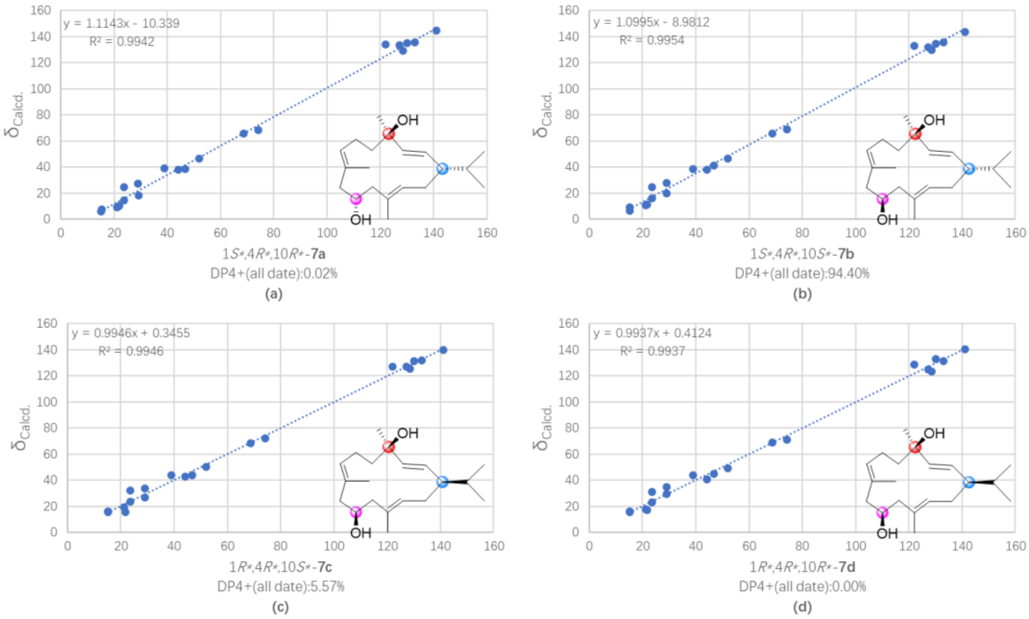

Figure S50. Regression analysis of experimental vs calculated ^{13}C NMR chemical shifts of (1*S**, 4*R**, 10*R**)-**7a**, (1*S**, 4*R**, 10*S**)-**7b**, (1*R**, 4*R**, 10*S**)-**7c**, (1*R**, 4*R**, 10*R**)-**7d** at the PCM/mPW1PW91/6-31+G** level using DP4+ method

Table S3. Cartesian coordinates of all conformers of isomers **5a**-**5b** used after optimization at the B3LYP/ 6-311G (d,p) level of theory as required for DP4+ analysis

05_3R4R13R14S_3

C	-7.7717	8.9752	7.5353
C	-8.3176	10.1438	7.1497
C	-7.6122	8.6512	9.0103
C	-8.6187	10.6204	5.7494
C	-6.1715	8.8286	9.5186
C	-7.8892	11.9175	5.3572
C	-6.4067	11.7958	4.9374
C	-5.4764	11.3959	6.0849
C	-4.0141	11.3988	5.7525
C	-3.3507	12.1398	6.643
C	-5.714	10.2628	9.4232
C	-4.4624	10.7011	9.1953
C	-4.1763	12.1849	9.0597
C	-4.3435	12.6917	7.6269
C	-1.8696	12.4189	6.7004
C	-1.0518	11.541	5.7389
C	-1.5949	13.9051	6.4219
C	-7.2767	7.9026	6.5935
C	-6.2426	10.8625	3.7249
O	-6.0052	13.0791	4.5028
C	-3.2725	9.7883	9.0143
O	-5.0307	12.9309	9.8948
O	-5.6254	12.332	7.1392
H	-8.6187	10.8524	7.9396
H	-8.2967	9.2802	9.6269
H	-7.9483	7.601	9.1788
H	-9.7179	10.8143	5.7324
H	-8.4619	9.8407	4.9747
H	-5.4984	8.1389	8.9626
H	-6.1306	8.5388	10.5952
H	-8.4349	12.3574	4.488
H	-7.9956	12.6633	6.1798
H	-5.7401	10.3922	6.4852
H	-3.5749	10.8415	4.911
H	-6.5095	11.0137	9.5566
H	-3.1523	12.4183	9.4339
H	-4.3098	13.8056	7.5984
H	-1.526	12.18	7.7354
H	0.0403	11.7282	5.8581
H	-1.2196	10.4567	5.9359
H	-1.3071	11.7496	4.6747
H	-0.5034	14.1268	6.4584
H	-1.9694	14.1981	5.4142

H	-2.0844	14.5638	7.1752
H	-7.3896	8.1456	5.5165
H	-6.193	7.7123	6.7598
H	-7.8296	6.9529	6.7719
H	-5.2429	10.9754	3.248
H	-6.3487	9.7928	4.0127
H	-6.9975	11.0844	2.9365
H	-5.9522	13.6377	5.2623
H	-3.4621	8.7381	9.3239
H	-2.9487	9.7737	7.9502
H	-2.418	10.1392	9.6356
H	-4.8702	12.6738	10.7879

05_3R4R13R14S_4

C	-7.7403	8.9618	7.5699
C	-8.2869	10.1481	7.2431
C	-7.5371	8.5829	9.0265
C	-8.6241	10.6778	5.8702
C	-6.082	8.7463	9.4983
C	-7.8898	11.9788	5.4989
C	-6.4199	11.8552	5.0373
C	-5.4636	11.4328	6.1549
C	-4.0107	11.4113	5.7841
C	-3.3091	12.1181	6.6731
C	-5.633	10.1853	9.441
C	-4.3856	10.6361	9.2136
C	-4.0976	12.1224	9.106
C	-4.2651	12.6727	7.6898
C	-1.8211	12.3621	6.7027
C	-1.0438	11.4728	5.7184
C	-1.5175	13.8437	6.4293
C	-7.2822	7.9202	6.576
C	-6.2935	10.9398	3.8069
O	-6.0213	13.1417	4.61
C	-3.1976	9.7269	9.0004
O	-4.9168	12.8591	9.983
O	-5.5669	12.3627	7.2213
H	-8.5579	10.8302	8.0664
H	-8.205	9.1865	9.6854
H	-7.8659	7.5263	9.1663
H	-9.7205	10.8857	5.8938
H	-8.5021	9.9233	5.0648
H	-5.424	8.0821	8.8948
H	-6.0078	8.4165	10.5615
H	-8.4528	12.4495	4.6574
H	-7.9668	12.7005	6.3459
H	-5.7331	10.4301	6.5536
H	-3.6044	10.8607	4.922
H	-6.4347	10.9225	9.602
H	-3.0698	12.3319	9.4812
H	-4.2004	13.7857	7.6906
H	-1.4636	12.1077	7.7293
H	0.0547	11.6319	5.8184
H	-1.2351	10.3914	5.9092
H	-1.3137	11.697	4.661
H	-0.4207	14.04	6.4461
H	-1.9044	14.1527	5.4311
H	-1.9773	14.5079	7.1964
H	-7.4364	8.197	5.5125
H	-6.1932	7.7251	6.6957
H	-7.8289	6.9651	6.7441
H	-5.3079	11.0579	3.3026
H	-6.3934	9.8662	4.0817
H	-7.0706	11.1752	3.0443
H	-5.9374	13.6914	5.3727
H	-3.3832	8.6718	9.2959
H	-2.8901	9.7292	7.9314
H	-2.3333	10.0658	9.6147
H	-5.7742	12.9294	9.5966

05_3R4R13R14S_5

C	-7.7362	8.9494	7.5861
C	-8.2989	10.1389	7.3009
C	-7.5093	8.5311	9.0284
C	-8.6629	10.703	5.9486
C	-6.0495	8.6947	9.4853
C	-7.951	12.0229	5.6006
C	-6.4898	11.935	5.103
C	-5.5171	11.4815	6.1953
C	-4.0685	11.4752	5.8063
C	-3.3586	12.1598	6.7058
C	-5.614	10.1387	9.4603
C	-4.3744	10.607	9.2262
C	-4.1034	12.0986	9.1518
C	-4.3034	12.6789	7.7518
C	-1.8719	12.413	6.7215
C	-1.1038	11.5608	5.698
C	-1.5824	13.9046	6.4908
C	-7.2829	7.9409	6.5566
C	-6.3822	11.0623	3.8393

O	-6.1342	13.2498	4.7238
C	-3.1816	9.7152	8.9713
O	-4.9177	12.8031	10.0591
O	-5.6101	12.3594	7.3046
H	-8.5644	10.7948	8.147
H	-8.1735	9.1094	9.7133
H	-7.8266	7.4678	9.1423
H	-9.7611	10.8968	5.996
H	-8.5465	9.9721	5.1211
H	-5.3937	8.0529	8.8558
H	-5.9582	8.3374	10.5383
H	-8.539	12.5102	4.7859
H	-8.0179	12.7194	6.4693
H	-5.7745	10.4611	6.5549
H	-3.6723	10.9538	4.9216
H	-6.4197	10.8642	9.6526
H	-3.072	12.3113	9.5151
H	-4.2533	13.7923	7.7759
H	-1.4971	12.1289	7.7339
H	-0.0051	11.7247	5.7863
H	-1.2849	10.4726	5.8573
H	-1.3916	11.8163	4.6524
H	-0.4869	14.1081	6.4978
H	-1.9862	14.2422	5.5086
H	-2.0353	14.5409	7.2852
H	-7.447	8.2502	5.5035
H	-6.1921	7.747	6.6605
H	-7.8241	6.9787	6.7002
H	-5.4036	11.1933	3.3248
H	-6.4799	9.9801	4.0786
H	-7.1698	11.3251	3.0969
H	-5.2374	13.2652	4.4329
H	-3.352	8.6519	9.2457
H	-2.8931	9.7451	7.8975
H	-2.3101	10.0488	9.5783
H	-5.7755	12.8901	9.6757

05_3R4R13R14S_6

C	-7.7723	8.9638	7.5535
C	-8.333	10.1379	7.2076
C	-7.5877	8.6019	9.0167
C	-8.662	10.6478	5.8253
C	-6.1411	8.7784	9.5084
C	-7.9526	11.9614	5.451
C	-6.478	11.87	4.9962
C	-5.531	11.4481	6.1238
C	-4.0724	11.4663	5.7749
C	-3.4028	12.1886	6.6757
C	-5.6977	10.2187	9.4442
C	-4.4539	10.6747	9.2082
C	-4.1849	12.1644	9.1059
C	-4.386	12.7043	7.6893
C	-1.9234	12.4796	6.719
C	-1.1131	11.6398	5.7178
C	-1.667	13.9763	6.4819
C	-7.2845	7.9203	6.576
C	-6.3312	10.9659	3.7586
O	-6.1223	13.1768	4.5902
C	-3.2587	9.7785	8.9847
O	-5.0288	12.8795	9.9776
O	-5.6719	12.3373	7.2181
H	-8.6267	10.8232	8.0204
H	-8.2684	9.208	9.66
H	-7.9123	7.5446	9.1618
H	-9.763	10.8315	5.8333
H	-8.5136	9.8888	5.0287
H	-5.4705	8.1097	8.9244
H	-6.0818	8.4606	10.5762
H	-8.5226	12.4157	4.6051
H	-8.0486	12.6865	6.2933
H	-5.7805	10.428	6.4903
H	-3.6417	10.937	4.9112
H	-6.4976	10.9583	9.6097
H	-3.156	12.3987	9.4657
H	-4.3673	13.8189	7.6868
H	-1.5607	12.2124	7.7404
H	-0.0211	11.8344	5.8249
H	-1.2671	10.5484	5.8843
H	-1.3881	11.878	4.6648
H	-0.5772	14.2075	6.5076
H	-2.0608	14.2964	5.4899
H	-2.1501	14.6067	7.2631
H	-7.4136	8.192	5.5077
H	-6.1974	7.7324	6.722
H	-7.8292	6.9627	6.7362
H	-5.3389	11.0891	3.2691
H	-6.4313	9.8895	4.0224
H	-7.0986	11.2056	2.9877
H	-5.2086	13.2014	4.3596

H	-3.4336	8.719	9.2701
H	-2.952	9.7944	7.9156
H	-2.3976	10.1216	9.6013
H	-4.8519	12.5979	10.8598

05_3R4R13R14S_7

C	-7.7337	8.9863	7.5834
C	-8.263	10.1903	7.2946
C	-7.5271	8.5617	9.027
C	-8.6064	10.7617	5.9402
C	-6.0678	8.693	9.4957
C	-7.8625	12.064	5.5924
C	-6.4011	11.9351	5.1048
C	-5.4457	11.4661	6.2054
C	-3.9936	11.4509	5.8304
C	-3.2862	12.1208	6.7429
C	-5.6037	10.1281	9.4792
C	-4.3548	10.5738	9.2502
C	-4.0568	12.0605	9.1811
C	-4.2375	12.645	7.7803
C	-1.7971	12.3574	6.7767
C	-1.0273	11.5061	5.7537
C	-1.488	13.8477	6.5636
C	-7.3011	7.9656	6.557
C	-6.2927	11.0465	3.8522
O	-5.9751	13.2292	4.7287
C	-3.1782	9.6613	8.9939
O	-4.8666	12.7771	10.0833
O	-5.5429	12.3374	7.3202
H	-8.5161	10.8534	8.1389
H	-8.1843	9.1535	9.707
H	-7.8683	7.5055	9.1372
H	-9.7001	10.9809	5.9816
H	-8.5021	10.0275	5.1138
H	-5.4204	8.0401	8.869
H	-5.9921	8.3305	10.5481
H	-8.4356	12.5588	4.7718
H	-7.9181	12.7669	6.4565
H	-5.7169	10.4462	6.557
H	-3.5923	10.9307	4.9472
H	-6.3959	10.868	9.6726
H	-3.0249	12.255	9.5531
H	-4.1779	13.758	7.8072
H	-1.4365	12.0594	7.7903
H	0.0722	11.6571	5.8551
H	-1.2218	10.4184	5.9014
H	-1.3011	11.7739	4.7075
H	-0.3903	14.0383	6.584
H	-1.8775	14.1995	5.5807
H	-1.9417	14.4814	7.3596
H	-7.449	8.2792	5.5029
H	-6.2169	7.7415	6.669
H	-7.8699	7.0188	6.6964
H	-5.31	11.1674	3.3431
H	-6.4017	9.9678	4.1024
H	-7.0745	11.3058	3.1023
H	-6.4912	13.5291	3.9989
H	-2.3016	9.9771	9.6031
H	-3.3685	8.6004	9.2647
H	-2.8876	9.6895	7.9206
H	-5.7165	12.8847	9.6881

05_3R4R13R14S_8

C	-7.771	9.005	7.5524
C	-8.2958	10.1954	7.2054
C	-7.6041	8.6351	9.0158
C	-8.6067	10.7143	5.8224
C	-6.1572	8.7773	9.5178
C	-7.8613	12.0076	5.4468
C	-6.388	11.8696	4.9997
C	-5.4578	11.4304	6.134
C	-3.9962	11.4434	5.7985
C	-3.3298	12.151	6.7133
C	-5.6846	10.2085	9.4638
C	-4.4318	10.6414	9.2329
C	-4.1356	12.1263	9.1357
C	-4.3201	12.6697	7.7183
C	-1.848	12.426	6.7762
C	-1.0359	11.5951	5.7692
C	-1.5725	13.9237	6.569
C	-7.3092	7.9486	6.5761
C	-6.2473	10.9487	3.7738
O	-5.9545	13.1538	4.5982
C	-3.2535	9.7239	9.0066
O	-4.9725	12.8543	10.0039
O	-5.604	12.3105	7.2355
H	-8.573	10.8882	8.0177
H	-8.2755	9.2548	9.656
H	-7.9537	7.5852	9.1562
H	-9.7024	10.9277	5.8274

H	-8.4768	9.951	5.0267
H	-5.497	8.098	8.9341
H	-6.1114	8.4529	10.5842
H	-8.417	12.4702	4.596
H	-7.9414	12.7403	6.284
H	-5.7198	10.4094	6.4901
H	-3.5602	10.9165	4.936
H	-6.4704	10.9628	9.6311
H	-3.105	12.3424	9.5022
H	-4.2947	13.7842	7.718
H	-1.4987	12.1369	7.7963
H	0.0569	11.777	5.8902
H	-1.202	10.5026	5.9158
H	-1.2981	11.8537	4.7177
H	-0.4805	14.142	6.6097
H	-1.9525	14.2662	5.5791
H	-2.0563	14.5453	7.3568
H	-7.4243	8.2252	5.5075
H	-6.2292	7.7281	6.7281
H	-7.8832	7.0075	6.7319
H	-5.2502	11.0536	3.2899
H	-6.367	9.8769	4.0482
H	-7.0069	11.1906	2.9957
H	-6.4783	13.4481	3.8721
H	-2.3865	10.0488	9.6247
H	-3.4487	8.6669	9.2884
H	-2.946	9.7377	7.9377
H	-4.8007	12.5716	10.8866

05_3R4R13R14S_9

C	-7.5628	8.9731	7.3369
C	-7.1292	9.0372	6.0631
C	-6.6913	8.3492	8.4117
C	-7.7906	9.7312	4.8983
C	-6.4822	9.2226	9.6614
C	-6.8746	10.77	4.218
C	-6.4906	12.0069	5.0625
C	-5.5176	11.6866	6.1986
C	-4.1096	11.3547	5.8086
C	-3.271	12.0003	6.6237
C	-5.871	10.5785	9.3935
C	-4.5652	10.8637	9.2318
C	-4.0843	12.2918	9.0316
C	-4.0913	12.8092	7.5909
C	-1.7638	11.9831	6.6164
C	-1.1813	10.8373	5.7727
C	-1.2194	13.3325	6.1218
C	-8.8967	9.5026	7.8065
C	-5.923	13.117	4.1587
O	-7.6786	12.5057	5.6378
C	-3.4777	9.8153	9.2519
O	-4.854	13.1723	9.8168
O	-5.4069	12.8011	7.0645
H	-6.149	8.5914	5.8233
H	-7.1737	7.3903	8.7153
H	-5.6932	8.077	7.9968
H	-8.0584	8.9396	4.1587
H	-8.7438	10.2289	5.1799
H	-5.8444	8.6729	10.3921
H	-7.449	9.368	10.1979
H	-5.9503	10.2705	3.8454
H	-7.4133	11.1293	3.3088
H	-5.9092	10.8695	6.8319
H	-3.841	10.6676	4.9914
H	-6.5917	11.411	9.3313
H	-3.0514	12.3872	9.4419
H	-3.7701	13.8763	7.56
H	-1.4289	11.826	7.6699
H	-0.0701	10.8081	5.8529
H	-1.5603	9.8464	6.1144
H	-1.43	10.9539	4.693
H	-0.1051	13.337	6.1102
H	-1.5725	13.5504	5.0876
H	-1.5409	14.1736	6.7775
H	-9.631	9.6667	6.9894
H	-9.3743	8.7737	8.4995
H	-8.7645	10.4682	8.344
H	-5.641	14.0194	4.7479
H	-5.0157	12.7714	3.613
H	-6.669	13.4421	3.3978
H	-7.4328	13.1077	6.3237
H	-3.8496	8.779	9.3999
H	-2.9234	9.8169	8.288
H	-2.7562	10.0245	10.0735
H	-4.7512	12.9297	10.7224

05_3R4R13R14S_10

C	-7.8161	9.0186	7.6096
C	-8.3368	10.1942	7.2118
C	-7.642	8.7207	9.0879

C	-8.6679	10.6438	5.8102
C	-6.1847	8.8439	9.5607
C	-7.9277	11.919	5.3729
C	-6.4681	11.7605	4.8915
C	-5.485	11.3511	5.9933
C	-4.0435	11.4304	5.5833
C	-3.3703	12.2081	6.4342
C	-5.6647	10.2527	9.4127
C	-4.4214	10.6287	9.0604
C	-4.0676	12.0982	8.9132
C	-4.321	12.6514	7.5105
C	-1.9217	12.6082	6.2975
C	-1.6842	14.0635	6.7283
C	-0.9685	11.6533	7.0292
C	-7.3753	7.9122	6.6803
C	-6.379	10.8129	3.6816
O	-6.0664	13.0327	4.4244
C	-3.3098	9.6539	8.7558
O	-4.8142	12.8688	9.8266
O	-5.6128	12.2587	7.0755
H	-8.5995	10.9275	7.9927
H	-8.2835	9.3938	9.7042
H	-8.0217	7.6908	9.2867
H	-8.5476	9.8449	5.0487
H	-9.7635	10.8571	5.8202
H	-5.5631	8.1001	9.0161
H	-6.1332	8.5844	10.6446
H	-8.5001	12.3596	4.5214
H	-7.9849	12.6781	6.1881
H	-5.7039	10.3301	6.3789
H	-3.6191	10.9442	4.6907
H	-6.4046	11.0408	9.6271
H	-3.0095	12.277	9.2117
H	-4.3423	13.7659	7.529
H	-1.6713	12.5399	5.2103
H	-0.6305	14.3718	6.5371
H	-2.3429	14.7644	6.165
H	-1.8772	14.2033	7.8164
H	0.0948	11.9229	6.8327
H	-1.1136	11.6883	8.1311
H	-1.1082	10.6018	6.6887
H	-7.4916	8.1424	5.6008
H	-6.2975	7.6834	6.8353
H	-7.9614	6.9875	6.8825

H	-5.3985	10.8955	3.161
H	-6.4988	9.7485	3.9836
H	-7.1615	11.0464	2.924
H	-5.9769	13.6017	5.1726
H	-3.5211	8.6096	9.0693
H	-3.1023	9.6394	7.6635
H	-2.3817	9.9436	9.2971
H	-4.591	12.5923	10.7002
05_3R4R13R14S_11			
C	-7.5136	8.9393	7.3648
C	-7.1073	9.0784	6.088
C	-6.6086	8.2822	8.3911
C	-7.8082	9.819	4.9759
C	-6.3802	9.1105	9.668
C	-6.9142	10.8784	4.2979
C	-6.4987	12.0892	5.1658
C	-5.517	11.7258	6.2814
C	-4.1287	11.3567	5.8544
C	-3.2516	11.9647	6.6576
C	-5.7873	10.4801	9.4287
C	-4.4856	10.7849	9.2671
C	-4.0121	12.2188	9.0866
C	-4.0245	12.7748	7.6604
C	-1.7461	11.9118	6.6079
C	-1.2151	10.7594	5.7397
C	-1.1853	13.252	6.1061
C	-8.8493	9.415	7.8844
C	-5.9237	13.2091	4.2787
O	-7.6721	12.592	5.7666
C	-3.3893	9.7446	9.2647
O	-4.7466	13.0862	9.9173
O	-5.3523	12.82	7.1659
H	-6.1243	8.6649	5.8056
H	-7.0714	7.3057	8.6678
H	-5.6173	8.0406	7.9422
H	-8.1087	9.0563	4.2188
H	-8.7456	10.3117	5.314
H	-5.7238	8.5405	10.3657
H	-7.3364	9.2293	10.2297
H	-6.0052	10.3891	3.8772
H	-7.4826	11.2674	3.4194
H	-5.9259	10.9125	6.9085
H	-3.8989	10.6793	5.0177
H	-6.5235	11.2992	9.3843

H	-2.9766	12.2959	9.4916
H	-3.6725	13.8326	7.6509
H	-1.3851	11.7408	7.6505
H	-0.1033	10.7021	5.7897
H	-1.6096	9.7752	6.0831
H	-1.4896	10.8913	4.6681
H	-0.072	13.2318	6.064
H	-1.5617	13.4844	5.0833
H	-1.4702	14.096	6.7751
H	-9.6048	9.592	7.0894
H	-9.2937	8.6466	8.5564
H	-8.7292	10.361	8.4584
H	-5.6199	14.0942	4.8829
H	-5.0283	12.8609	3.7151
H	-6.673	13.5619	3.5336
H	-7.4161	13.2482	6.3949
H	-3.7519	8.7007	9.3781
H	-2.829	9.7771	8.305
H	-2.6745	9.9353	10.0967
H	-5.5832	13.2382	9.5103

05_3R4R13R14S_12

C	-7.8743	9.019	7.5698
C	-8.3602	10.2237	7.217
C	-7.6674	8.6755	9.0339
C	-8.6883	10.7324	5.8351
C	-6.198	8.7847	9.4735
C	-7.8908	11.9825	5.4244
C	-6.4332	11.7724	4.9553
C	-5.473	11.3612	6.0764
C	-4.0289	11.3285	5.6742
C	-3.3047	12.0707	6.5158
C	-5.6831	10.1988	9.3667
C	-4.4245	10.5865	9.0906
C	-4.0727	12.0587	8.9793
C	-4.2291	12.6225	7.5648
C	-1.8571	12.4388	6.3032
C	-1.7827	13.8	5.5912
C	-1.0082	12.4525	7.5803
C	-7.4885	7.926	6.6011
C	-6.3647	10.8002	3.7642
O	-5.9888	13.0222	4.467
C	-3.2816	9.6273	8.855
O	-4.8798	12.8239	9.8443
O	-5.5411	12.3367	7.1043
H	-8.5805	10.9427	8.024
H	-8.2938	9.3308	9.6841
H	-8.0435	7.6406	9.2123
H	-9.772	10.9981	5.8657
H	-8.6154	9.9521	5.0487
H	-5.5862	8.0698	8.8801
H	-6.1185	8.4833	10.5447
H	-8.4368	12.4568	4.5737
H	-7.925	12.7315	6.2502
H	-5.7531	10.376	6.5109
H	-3.642	10.8234	4.7753
H	-6.438	10.9813	9.5452
H	-3.041	12.224	9.3613
H	-4.1438	13.7341	7.5734
H	-1.4144	11.6763	5.6165
H	-0.7291	14.0724	5.3507
H	-2.3512	13.7866	4.6323
H	-2.2045	14.6133	6.225
H	0.0624	12.6515	7.3436
H	-1.338	13.2527	8.2806
H	-1.0483	11.472	8.1065
H	-7.6579	8.1741	5.5328
H	-6.4067	7.6854	6.7019
H	-8.0734	7.0028	6.8138
H	-5.3862	10.8566	3.2365
H	-6.5001	9.7445	4.0886
H	-7.1467	11.032	3.0055
H	-5.8738	13.5968	5.2076
H	-2.4247	9.8843	9.5173
H	-3.5263	8.5662	9.0742
H	-2.9386	9.6789	7.7981
H	-4.722	12.5379	10.7291

05_3R4R13R14S_13

C	-7.0818	8.7012	7.1522
C	-6.851	9.0609	5.8755
C	-6.0274	7.9706	7.9635
C	-7.7568	9.8835	4.9882
C	-5.6093	8.6862	9.262
C	-7.0278	11.0268	4.2534
C	-6.5887	12.2325	5.1168
C	-5.6881	11.8405	6.2877
C	-4.3695	11.2362	5.9099
C	-3.4133	11.7591	6.6799
C	-4.7918	9.937	9.0326

C	-5.0975	11.2088	9.3557
C	-4.1147	12.3324	9.064
C	-4.0541	12.7652	7.5956
C	-1.9371	11.4564	6.6597
C	-1.6089	10.1344	5.9466
C	-1.1672	12.6175	6.0116
C	-8.3818	8.9876	7.865
C	-5.9175	13.297	4.2295
O	-7.7603	12.8104	5.651
C	-6.3976	11.6365	9.997
O	-4.4187	13.4705	9.8365
O	-5.3581	12.9723	7.075
H	-5.8934	8.7654	5.4172
H	-6.4513	6.9703	8.2171
H	-5.1151	7.7716	7.3536
H	-8.1817	9.1807	4.233
H	-8.6157	10.319	5.5451
H	-4.9617	7.9969	9.8545
H	-6.4993	8.8592	9.9044
H	-6.1512	10.6122	3.7034
H	-7.7177	11.4148	3.466
H	-6.2428	11.1641	6.9712
H	-4.2329	10.4716	5.1308
H	-3.8153	9.7558	8.5537
H	-3.0946	12.0237	9.3935
H	-3.5271	13.7422	7.4938
H	-1.6031	11.3495	7.7194
H	-0.5229	9.8972	6.0265
H	-2.1663	9.2813	6.3988
H	-1.8553	10.1781	4.861
H	-0.0719	12.4131	5.9928
H	-1.5021	12.7816	4.9615
H	-1.3123	13.5708	6.5694
H	-9.2504	9.0558	7.1744
H	-8.6304	8.1682	8.5764
H	-8.3132	9.9418	8.4314
H	-5.6367	14.2023	4.8146
H	-4.9903	12.9027	3.7547
H	-6.5983	13.6311	3.4135
H	-7.4986	13.4655	6.2792
H	-7.1262	10.8167	10.1602
H	-6.2085	12.0827	10.999
H	-6.9066	12.3945	9.3595
H	-4.1993	13.2904	10.736

05_3R4R13S14S_3

C	2.924407	2.042198	-0.34286
C	3.141767	0.556377	-0.48185
C	1.644037	2.571412	-1.03986
C	-0.11528	1.103115	0.116976
C	-1.41048	0.950097	0.869441
C	3.366214	-0.37054	0.464176
C	1.14762	-2.3754	-0.42237
C	3.574036	-1.82187	0.069908
C	-1.21385	-1.92468	-1.02245
C	-1.54103	-0.40624	-1.00369
C	2.371813	-2.75469	0.369096
C	-0.13618	-2.35659	-0.03165
C	0.308263	2.546973	-0.25144
C	-2.23186	0.096519	0.251956
C	-3.6279	-0.32918	0.645821
C	3.436536	-0.08892	1.946495
C	-0.6341	-2.75973	1.335547
O	-0.35541	0.391878	-1.11802
C	-4.03439	0.178121	2.036007
C	-4.66136	0.108895	-0.41524
C	0.376232	3.435423	0.992619
O	-0.70395	3.113386	-1.09487
O	-0.8719	-2.20556	-2.38176
H	0.712837	0.623497	0.655735
H	-2.14333	-0.23168	-1.90615
H	2.949843	2.359724	0.704628
H	3.776671	2.546803	-0.82292
H	3.123677	0.210618	-1.51733
H	1.800208	3.620112	-1.32026
H	1.494076	2.019457	-1.97624
H	-1.60662	1.457338	1.806361
H	1.3472	-2.07212	-1.44924
H	3.794175	-1.88001	-1.00348
H	4.453815	-2.22334	0.593477
H	-2.15001	-2.45226	-0.76887
H	2.679414	-3.77848	0.103893
H	2.168184	-2.77176	1.445218
H	-3.64702	-1.42966	0.67575
H	4.427739	-0.3511	2.342521
H	3.245781	0.958013	2.194552
H	2.711259	-0.69899	2.501614
H	-1.31416	-3.62019	1.262996
H	0.178249	-3.03757	2.011184

H	-1.20155	-1.94925	1.808479
H	-5.03085	-0.19303	2.29926
H	-3.3328	-0.15611	2.808356
H	-4.06904	1.273897	2.061042
H	-5.66306	-0.23588	-0.13464
H	-4.68787	1.201654	-0.49956
H	-4.43303	-0.30083	-1.40482
H	0.766718	4.423829	0.727364
H	-0.62212	3.570643	1.418127
H	1.025991	3.007048	1.762946
H	-0.89788	2.41195	-1.7431
H	-0.51329	-3.10767	-2.39953

05_3R4R13S14S_4

C	2.971636	1.969558	-0.29842
C	3.152275	0.476178	-0.41847
C	1.706831	2.538388	-0.99529
C	-0.11342	1.090743	0.075899
C	-1.38575	0.921684	0.864546
C	3.310631	-0.44883	0.543834
C	1.112072	-2.42022	-0.46382
C	3.506125	-1.90944	0.176188
C	-1.18045	-1.83808	-1.22392
C	-1.6054	-0.34698	-1.05863
C	2.268581	-2.81502	0.416518
C	-0.19296	-2.33978	-0.16758
C	0.365155	2.532933	-0.2145
C	-2.23349	0.094241	0.246341
C	-3.61086	-0.35673	0.674348
C	3.313263	-0.15572	2.02576
C	-0.79235	-2.74898	1.157806
O	-0.4288	0.47288	-1.19832
C	-3.93796	0.024477	2.124334
C	-4.69384	0.182723	-0.28626
C	0.462865	3.35083	1.075311
O	-0.61996	3.183413	-1.028
O	-0.68772	-1.96398	-2.55833
H	0.709811	0.540998	0.548738
H	-2.25308	-0.13628	-1.92065
H	3.009804	2.298414	0.744787
H	3.836577	2.443501	-0.78653
H	3.173845	0.12268	-1.45171
H	1.891122	3.588063	-1.25369
H	1.55126	2.009513	-1.94456
H	-1.53691	1.381346	1.833939
H	1.405171	-2.17022	-1.48188
H	3.784891	-1.98554	-0.88241
H	4.346974	-2.32182	0.752284
H	-2.08854	-2.45523	-1.18784
H	2.572395	-3.84897	0.190194
H	1.995203	-2.80362	1.477013
H	-3.63666	-1.45514	0.603769
H	4.265062	-0.46623	2.478709
H	3.164239	0.901868	2.256236
H	2.526286	-0.72195	2.542234
H	-1.58437	-3.49533	1.004079
H	-0.05008	-3.19421	1.824723
H	-1.25104	-1.9042	1.683798
H	-4.92173	-0.36547	2.407211
H	-3.19869	-0.38001	2.824582
H	-3.96288	1.113652	2.249756
H	-5.68204	-0.18647	0.010358
H	-4.7176	1.278493	-0.26305
H	-4.5183	-0.13005	-1.32101
H	0.904352	4.330941	0.865233
H	-0.53367	3.512531	1.495619
H	1.080615	2.852724	1.829671
H	-0.85955	2.518505	-1.69895
H	-0.06835	-1.22289	-2.67747

05_3R4R13S14S_5

C	2.913127	2.030466	-0.49911
C	3.104245	0.536385	-0.58272
C	1.588596	2.55285	-1.10936
C	-0.11163	1.078097	0.125517
C	-1.43127	0.964073	0.848771
C	3.380066	-0.34914	0.389244
C	1.13488	-2.40435	-0.35726
C	3.56583	-1.81842	0.051836
C	-1.22865	-1.96918	-0.94763
C	-1.51818	-0.43965	-0.99141
C	2.373362	-2.73412	0.433617
C	-0.14385	-2.36903	0.048048
C	0.323275	2.523104	-0.2267
C	-2.24891	0.110624	0.222018
C	-3.66096	-0.28616	0.58581
C	3.538207	-0.00231	1.851015
C	-0.6251	-2.70544	1.438927
O	-0.31448	0.321502	-1.07755
C	-4.1067	0.271482	1.944351

C	-4.65462	0.123079	-0.52354
C	0.514318	3.340745	1.063647
O	-0.68296	3.156289	-1.03358
O	-0.90641	-2.31455	-2.29709
H	0.696645	0.619499	0.712835
H	-2.08353	-0.28102	-1.9208
H	3.726499	2.496341	-1.07602
H	3.035373	2.397692	0.524801
H	3.019794	0.144457	-1.59811
H	1.726345	3.602764	-1.39709
H	1.363577	2.003069	-2.02936
H	-1.64508	1.487205	1.773459
H	1.319476	-2.14958	-1.39984
H	3.747627	-1.92578	-1.02493
H	4.46311	-2.19933	0.560808
H	-2.17008	-2.47006	-0.66236
H	2.679267	-3.77182	0.226844
H	2.190192	-2.68383	1.51271
H	-3.69073	-1.38461	0.651901
H	4.553349	-0.24181	2.197922
H	3.354331	1.053363	2.064674
H	2.85226	-0.59149	2.474707
H	-1.29643	-3.5756	1.418709
H	0.197107	-2.93864	2.119739
H	-1.19738	-1.87778	1.875375
H	-5.11596	-0.0793	2.185322
H	-3.43612	-0.04551	2.750692
H	-4.1289	1.367936	1.933043
H	-5.66734	-0.20593	-0.2651
H	-4.67154	1.212806	-0.64437
H	-4.39544	-0.31984	-1.49065
H	0.922008	4.327307	0.818893
H	-0.44505	3.494787	1.568973
H	1.192986	2.855171	1.773749
H	-1.54534	2.875088	-0.68271
H	-0.55655	-3.22011	-2.27859

05_3R4R13S14S_6

C	2.904105	2.023112	-0.4721
C	3.096385	0.530358	-0.567
C	1.585767	2.554937	-1.09209
C	-0.13461	1.108879	0.137278
C	-1.45543	0.979028	0.849227
C	3.370057	-0.36119	0.399911
C	1.144807	-2.41993	-0.37334
C	3.569235	-1.826	0.051225
C	-1.21537	-1.96373	-0.96123
C	-1.50821	-0.43528	-0.98209
C	2.38176	-2.75462	0.417247
C	-0.13341	-2.37584	0.032004
C	0.304956	2.545062	-0.22287
C	-2.24766	0.099267	0.232247
C	-3.65546	-0.31906	0.590397
C	3.514829	-0.02587	1.865669
C	-0.61679	-2.70787	1.423029
O	-0.30344	0.326734	-1.0539
C	-4.11629	0.239558	1.943627
C	-4.65124	0.069344	-0.52423
C	0.487566	3.375149	1.059488
O	-0.76711	3.106952	-0.99785
O	-0.88904	-2.29328	-2.31439
H	0.676628	0.672666	0.739806
H	-2.07155	-0.26362	-1.91118
H	3.013874	2.380681	0.556592
H	3.724886	2.493985	-1.0344
H	3.021051	0.146525	-1.58605
H	1.750248	3.603785	-1.38028
H	1.36319	2.007183	-2.01381
H	-1.68966	1.522456	1.756646
H	1.33119	-2.16453	-1.41551
H	3.758138	-1.92224	-1.02539
H	4.466782	-2.20393	0.562071
H	-2.15691	-2.46967	-0.68532
H	2.695704	-3.78717	0.196989
H	2.195235	-2.72035	1.496333
H	-3.67078	-1.41761	0.664325
H	4.529194	-0.26062	2.218144
H	3.320575	1.026235	2.087673
H	2.82869	-0.62595	2.47852
H	-1.30508	-3.56469	1.401768
H	0.202439	-2.95917	2.101048
H	-1.17188	-1.86991	1.861801
H	-5.12009	-0.12744	2.184309
H	-3.44227	-0.05971	2.754036
H	-4.15724	1.335292	1.924349
H	-5.66117	-0.27013	-0.26696
H	-4.67904	1.15751	-0.65412
H	-4.38327	-0.37784	-1.48714
H	0.896843	4.363338	0.814579

H	-0.4761	3.525157	1.554892
H	1.170165	2.901228	1.773088
H	-0.48542	3.995623	-1.26992
H	-0.54762	-3.20219	-2.30615

05_3R4R13S14S_7

C	-2.94469	2.013206	-0.85006
C	-2.76434	0.582171	-1.28889
C	-1.62527	2.811517	-0.68152
C	0.009492	1.030489	0.120056
C	1.219662	1.03984	-0.77338
C	-3.19218	-0.54753	-0.69866
C	-0.8984	-2.31591	0.482226
C	-2.78864	-1.894	-1.2716
C	1.465308	-1.80434	1.085212
C	1.707031	-0.2702	1.075425
C	-2.08387	-2.86709	-0.28045
C	0.396155	-2.32283	0.119432
C	-0.68668	2.371338	0.464671
C	2.199622	0.295262	-0.24939
C	3.581163	0.01901	-0.79655
C	-4.05733	-0.58857	0.537928
C	0.909302	-2.86324	-1.19348
O	0.49951	0.444924	1.34652
C	3.760304	0.516173	-2.23731
C	4.671252	0.612566	0.12255
C	0.331586	3.473087	0.789918
O	-1.51408	2.141102	1.615873
O	1.168412	-2.11241	2.449792
H	-0.75502	0.36712	-0.28655
H	2.400225	-0.07509	1.905288
H	-3.53681	2.547792	-1.60876
H	-3.50277	2.073672	0.086381
H	-2.15981	0.467645	-2.1926
H	-1.05807	2.80015	-1.62295
H	-1.89789	3.859891	-0.50478
H	1.244508	1.54776	-1.73101
H	-1.12625	-1.876	1.451568
H	-3.68016	-2.42062	-1.64762
H	-2.13161	-1.73366	-2.13467
H	2.425202	-2.27463	0.807228
H	-2.82336	-3.21484	0.451824
H	-1.79197	-3.75424	-0.85561
H	3.725867	-1.07248	-0.80012
H	-3.51222	-1.0032	1.396181
H	-4.42602	0.39692	0.829124
H	-4.92753	-1.23982	0.375934
H	1.673329	-3.63623	-1.03254
H	0.113696	-3.29576	-1.80592
H	1.384366	-2.07028	-1.78517
H	4.756541	0.253574	-2.60959
H	3.018449	0.074247	-2.91168
H	3.660618	1.606933	-2.29309
H	5.667639	0.370276	-0.26418
H	4.580969	1.704052	0.172694
H	4.604734	0.22118	1.14312
H	-0.19054	4.355057	1.174921
H	1.035757	3.125464	1.553807
H	0.908873	3.768078	-0.09415
H	-0.97015	1.603417	2.2185
H	0.801688	-3.01106	2.460041

05_3R4R13S14S_8

C	3.127613	1.774153	-0.30301
C	3.18178	0.286805	-0.53934
C	1.89466	2.473717	-0.93204
C	0.007752	1.17977	0.240722
C	-1.28651	1.178224	1.009113
C	3.326245	-0.71567	0.334862
C	0.874361	-2.42317	-0.54796
C	3.366629	-2.14965	-0.16042
C	-1.44131	-1.65999	-0.96538
C	-1.61523	-0.11626	-0.8817
C	2.090841	-2.97788	0.143652
C	-0.35643	-2.24104	-0.06091
C	0.593914	2.575086	-0.09398
C	-2.22332	0.454032	0.39187
C	-3.68063	0.35703	0.804877
C	3.461533	-0.54199	1.828657
C	-0.78715	-2.59762	1.340901
O	-0.34851	0.546969	-1.00814
C	-4.62048	0.71885	-0.36252
C	-4.09352	-0.98906	1.432712
C	0.809504	3.40182	1.176052
O	-0.3703	3.291191	-0.87811
O	-1.2001	-1.91227	-2.35262
H	0.782205	0.584355	0.74328
H	-2.21276	0.147605	-1.76474
H	3.208194	2.020284	0.760837
H	4.018184	2.216462	-0.77478

H	3.103234	0.013037	-1.59352
H	2.160859	3.507478	-1.18336
H	1.648539	1.978432	-1.87947
H	-1.42561	1.719431	1.93876
H	1.037537	-2.1269	-1.58314
H	3.52962	-2.15505	-1.2455
H	4.22402	-2.67134	0.289494
H	-2.40729	-2.10929	-0.68175
H	2.276933	-4.00416	-0.21
H	1.943182	-3.0542	1.226072
H	-3.81832	1.120175	1.582587
H	4.416514	-0.95701	2.180544
H	3.416494	0.50306	2.145433
H	2.672365	-1.08591	2.365131
H	-1.62249	-3.31102	1.323656
H	0.02033	-3.04895	1.922274
H	-1.14093	-1.71483	1.885466
H	-5.65652	0.775303	-0.00973
H	-4.35742	1.688069	-0.80033
H	-4.58338	-0.0346	-1.1584
H	-5.13377	-0.93275	1.774593
H	-4.02823	-1.81536	0.71635
H	-3.46792	-1.23755	2.295882
H	1.304216	4.347134	0.9279
H	-0.14965	3.638105	1.645267
H	1.431044	2.872462	1.905672
H	-0.67	2.644854	-1.54317
H	-0.94935	-2.84734	-2.42731

05_3R4R13S14S_9

C	-2.88588	1.939381	-0.87486
C	-2.66115	0.513401	-1.31501
C	-1.59251	2.787531	-0.76656
C	0.012501	1.053353	0.183935
C	1.143372	0.908175	-0.79859
C	-3.13947	-0.6267	-0.78697
C	-0.92621	-2.31595	0.569926
C	-2.6673	-1.96258	-1.33185
C	1.358473	-1.69191	1.37757
C	1.752476	-0.19863	1.139062
C	-2.01933	-2.91754	-0.28565
C	0.398122	-2.3081	0.34897
C	-0.61814	2.451291	0.385301
C	2.149542	0.204454	-0.26774
C	3.486991	-0.15731	-0.87184
C	-4.12919	-0.68783	0.351647
C	1.05141	-2.93466	-0.86043
O	0.603128	0.616069	1.435896
C	3.556611	0.131658	-2.37728
C	4.638108	0.557053	-0.12924
C	0.449931	3.544436	0.527534
O	-1.40707	2.400713	1.584717
O	0.865636	-1.76174	2.714362
H	-0.79808	0.365618	-0.0586
H	2.518065	0.036649	1.889593
H	-3.5285	2.447007	-1.61085
H	-3.4117	1.982625	0.082057
H	-1.97557	0.41291	-2.16046
H	-1.04113	2.744075	-1.71585
H	-1.89317	3.834884	-0.63671
H	1.100439	1.301939	-1.8078
H	-1.27479	-1.85254	1.491182
H	-3.51481	-2.50589	-1.77887
H	-1.94571	-1.78426	-2.13783
H	2.283899	-2.28391	1.369561
H	-2.80628	-3.29061	0.382142
H	-1.64985	-3.79331	-0.83241
H	3.63645	-1.23864	-0.73005
H	-3.69074	-1.15413	1.243751
H	-4.50183	0.29691	0.641791
H	-4.99496	-1.30467	0.072706
H	1.874147	-3.59835	-0.56123
H	0.345459	-3.52375	-1.45199
H	1.480833	-2.17748	-1.52725
H	4.525158	-0.18258	-2.78103
H	2.771373	-0.39819	-2.92797
H	3.44421	1.204065	-2.57695
H	5.605029	0.248128	-0.54214
H	4.551677	1.644376	-0.23961
H	4.642939	0.325307	0.941013
H	-0.02492	4.492328	0.800245
H	1.165718	3.275678	1.312127
H	1.008711	3.688732	-0.40399
H	-0.83474	1.992512	2.257013
H	0.288963	-0.98293	2.810994

05_3R4R13S14S_10

C	-3.05752	1.880472	0.363755
C	-3.16707	0.391854	0.571899
C	-1.77759	2.516835	0.964926

C	0.03069	1.134334	-0.23132
C	1.302964	1.073442	-1.03274
C	-3.38229	-0.58818	-0.32092
C	-0.97979	-2.39007	0.504996
C	-3.46499	-2.0305	0.144632
C	1.378456	-1.74973	0.933461
C	1.629138	-0.21518	0.862629
C	-2.21859	-2.89014	-0.19062
C	0.267389	-2.27079	0.023392
C	-0.49141	2.556538	0.099498
C	2.232218	0.330609	-0.42394
C	3.683507	0.222747	-0.85013
C	-3.55512	-0.3787	-1.80643
C	0.691285	-2.65526	-1.37402
O	0.396524	0.505457	1.017925
C	4.587055	1.004	0.128922
C	4.222601	-1.20643	-1.04469
C	-0.69924	3.379067	-1.17466
O	0.517172	3.239567	0.857314
O	1.124902	-2.00021	2.319073
H	-0.77903	0.562099	-0.70336
H	2.254814	0.01174	1.736661
H	-3.16237	2.152836	-0.69145
H	-3.91263	2.349241	0.874143
H	-3.06561	0.092191	1.617142
H	-1.98828	3.562106	1.221071
H	-1.53585	2.009431	1.907157
H	1.436731	1.595102	-1.97418
H	-1.13499	-2.08439	1.538786
H	-3.61632	-2.0546	1.23116
H	-4.34343	-2.51464	-0.30633
H	2.318485	-2.24521	0.645395
H	-2.43338	-3.91897	0.138743
H	-2.08111	-2.94451	-1.27578
H	3.747454	0.72792	-1.8233
H	-4.54164	-0.73521	-2.13466
H	-3.46351	0.66791	-2.10696
H	-2.81352	-0.95395	-2.37704
H	1.471376	-3.42871	-1.34556
H	-0.13806	-3.04591	-1.96813
H	1.116109	-1.79947	-1.91152
H	5.61965	1.021955	-0.23891
H	4.244349	2.03792	0.241853
H	4.597362	0.538794	1.121913
H	5.233194	-1.16369	-1.46724
H	4.290636	-1.7491	-0.09513
H	3.597243	-1.78952	-1.7281
H	-1.159	4.342303	-0.92816
H	0.260082	3.57947	-1.65997
H	-1.34855	2.864422	-1.89052
H	0.805717	2.585537	1.519864
H	0.825063	-2.92123	2.386052

05_3R4R13S14S_11

C	2.837268	2.351429	0.614494
C	2.891173	0.955904	1.188496
C	1.431915	2.984461	0.448507
C	-0.17628	1.059814	0.040432
C	-1.51894	1.224257	0.710853
C	3.321433	-0.17697	0.604569
C	1.040215	-2.38178	0.759467
C	3.342267	-1.48357	1.379202
C	-1.26424	-1.99339	0.005409
C	-1.62278	-0.62758	-0.67405
C	2.5212	-2.6491	0.761459
C	0.166947	-2.42995	-0.26022
C	0.500786	2.314285	-0.58875
C	-2.37124	0.283303	0.28893
C	-3.80067	0.018543	0.69149
C	3.832815	-0.26402	-0.81402
C	0.484933	-2.85156	-1.67259
O	-0.45458	0.120076	-1.01718
C	-4.23833	0.836103	1.913709
C	-4.75932	0.251995	-0.49653
C	-0.5389	3.312187	-1.12008
O	1.318875	1.883566	-1.68435
O	-2.24841	-2.91747	-0.4821
H	0.545221	0.598539	0.725059
H	-2.1615	-0.85252	-1.60149
H	3.396216	3.016637	1.290384
H	3.341494	2.394026	-0.35243
H	2.5444	0.880129	2.222417
H	0.912776	3.014269	1.417199
H	1.582152	4.030894	0.15341
H	-1.71485	1.976166	1.466888
H	0.648911	-2.07525	1.732887
H	4.384151	-1.83013	1.461977
H	2.990362	-1.30964	2.40436
H	-1.3841	-1.87794	1.091894

H	2.890493	-2.87256	-0.24427
H	2.726179	-3.54341	1.369669
H	-3.86939	-1.04945	0.949554
H	3.120448	-0.79942	-1.45472
H	3.996581	0.714226	-1.26786
H	4.77686	-0.82502	-0.85045
H	0.432061	-1.99222	-2.35259
H	1.477929	-3.29814	-1.76454
H	-0.25294	-3.5818	-2.02381
H	-5.26478	0.579579	2.198289
H	-3.58902	0.647935	2.775917
H	-4.2122	1.911394	1.698727
H	-5.78771	0.003622	-0.2102
H	-4.73706	1.302027	-0.81157
H	-4.49345	-0.36747	-1.35934
H	-0.02988	4.097643	-1.6881
H	-1.24884	2.807349	-1.78493
H	-1.10846	3.782268	-0.31027
H	0.817375	1.167066	-2.11247
H	-2.13799	-3.74438	0.014755

05_3R4S13R14S_3

C	3.36323	-0.31547	0.436455
C	3.100705	0.626918	-0.48416
C	3.615614	-1.74955	0.007501
C	2.842085	2.100639	-0.29548
C	2.454293	-2.732	0.311631
C	1.582578	2.637349	-1.01798
C	0.251779	2.566689	-0.23244
C	-0.14068	1.110185	0.11428
C	-1.43335	0.943153	0.864276
C	-2.22648	0.056726	0.255446
C	1.204085	-2.38306	-0.45109
C	-0.06941	-2.37052	-0.02801
C	-1.1739	-1.96285	-0.99832
C	-1.51224	-0.44525	-0.98908
C	-3.61254	-0.40154	0.648272
C	-4.04889	0.134397	2.018563
C	-4.64971	-0.0316	-0.43458
C	3.435488	-0.06656	1.924493
C	-0.8549	3.276342	-1.02085
O	0.405701	3.182938	1.061924
C	-0.52911	-2.74743	1.359726
O	-0.85903	-2.25802	-2.36205
O	-0.33326	0.359113	-1.09449

H	3.084815	0.307431	-1.52818
H	4.519923	-2.12911	0.505483
H	3.815913	-1.77813	-1.07105
H	2.784499	2.374687	0.762224
H	3.705832	2.646271	-0.7057
H	2.271969	-2.77634	1.390678
H	2.794178	-3.73848	0.020545
H	1.749061	3.697612	-1.25795
H	1.449367	2.124264	-1.97799
H	0.692597	0.677967	0.684383
H	-1.63859	1.47668	1.784094
H	1.373123	-2.09403	-1.48746
H	-2.09835	-2.49785	-0.71888
H	-2.10926	-0.28218	-1.89817
H	-3.5965	-1.50066	0.712836
H	-5.03608	-0.25942	2.28411
H	-3.34471	-0.15407	2.806665
H	-4.11693	1.228949	2.010266
H	-5.64256	-0.39986	-0.15209
H	-4.71148	1.056628	-0.55407
H	-4.39833	-0.46391	-1.4088
H	3.204325	0.965307	2.198427
H	2.739543	-0.71801	2.469911
H	4.439861	-0.29895	2.306281
H	-1.78815	3.289836	-0.44967
H	-0.56786	4.313316	-1.23619
H	-1.03793	2.769443	-1.97309
H	0.642421	4.11257	0.91025
H	0.301839	-3.01335	2.017267
H	-1.08111	-1.92606	1.832486
H	-1.21294	-3.60725	1.323375
H	-0.48768	-3.15505	-2.37506

05_3R4S13R14S_4

C	3.349848	-0.3514	0.462507
C	3.12379	0.603748	-0.4546
C	3.589309	-1.78488	0.024618
C	2.879323	2.079109	-0.26003
C	2.410989	-2.75407	0.303984
C	1.635666	2.632946	-0.99994
C	0.29344	2.573578	-0.24442
C	-0.11899	1.115355	0.093625
C	-1.40497	0.951718	0.859223
C	-2.2163	0.078661	0.254329
C	1.17143	-2.37847	-0.46429

C	-0.10648	-2.37116	-0.05407
C	-1.20104	-1.94115	-1.02618
C	-1.52399	-0.42046	-1.00326
C	-3.59815	-0.37441	0.666955
C	-4.01075	0.154021	2.047436
C	-4.64991	0.007359	-0.39772
C	3.389704	-0.11703	1.954129
C	-0.78874	3.294381	-1.0552
O	0.516278	3.257768	1.005343
C	-0.58073	-2.7783	1.320252
O	-0.8812	-2.22364	-2.39105
O	-0.33866	0.37126	-1.11708
H	3.128133	0.293941	-1.50181
H	3.804286	-1.80632	-1.05125
H	4.481278	-2.18145	0.531604
H	2.819467	2.351058	0.797399
H	3.752437	2.616165	-0.66154
H	2.218256	-2.8108	1.380623
H	2.739013	-3.76141	0.002437
H	1.813665	3.691275	-1.22722
H	1.512261	2.117423	-1.95962
H	0.714875	0.668966	0.650882
H	-1.59886	1.453178	1.800984
H	1.353288	-2.06545	-1.49155
H	-2.13245	-2.46975	-0.7577
H	-2.13149	-0.24473	-1.90303
H	-3.58609	-1.47383	0.723759
H	-4.99559	-0.23695	2.325213
H	-3.29661	-0.14371	2.823111
H	-4.07448	1.248956	2.047883
H	-5.63963	-0.35858	-0.10199
H	-4.70898	1.096543	-0.50948
H	-4.41547	-0.41994	-1.37827
H	3.162302	0.9148	2.231553
H	2.67471	-0.76643	2.476957
H	4.382367	-0.36284	2.357514
H	-1.74181	3.31268	-0.51226
H	-0.48572	4.32757	-1.25484
H	-0.96009	2.786447	-2.00862
H	-0.35275	3.446533	1.393602
H	0.242682	-3.06336	1.979213
H	-1.1334	-1.96592	1.807827
H	-1.26786	-3.63392	1.257776
H	-0.50986	-3.12053	-2.41089

05_3R4S13R14S_5

C	3.28434	-0.4321	0.543255
C	3.143823	0.511701	-0.40348
C	3.517572	-1.88085	0.153123
C	2.935101	1.997805	-0.24575
C	2.300141	-2.81896	0.372092
C	1.695912	2.580901	-0.97135
C	0.355954	2.541234	-0.19486
C	-0.1173	1.090768	0.052328
C	-1.38002	0.920298	0.853415
C	-2.22038	0.077906	0.244534
C	1.134291	-2.42856	-0.49726
C	-0.16836	-2.35197	-0.18938
C	-1.16593	-1.85038	-1.2359
C	-1.59269	-0.36014	-1.06307
C	-3.58851	-0.39059	0.683702
C	-3.91479	0.002991	2.130614
C	-4.68603	0.116588	-0.27775
C	3.227621	-0.16976	2.029835
C	-0.70514	3.363262	-0.93442
O	0.535276	3.058505	1.138369
C	-0.75508	-2.75924	1.142226
O	-0.67946	-1.96745	-2.57411
O	-0.41929	0.45848	-1.20715
H	3.208324	0.183486	-1.4435
H	3.804753	-1.93267	-0.90476
H	4.364227	-2.28319	0.728432
H	2.89222	2.291981	0.806611
H	3.817668	2.501278	-0.66865
H	2.028296	-2.8394	1.432704
H	2.626622	-3.84011	0.120341
H	1.900929	3.637462	-1.19628
H	1.553987	2.08631	-1.94031
H	0.709522	0.558787	0.538243
H	-1.51958	1.399165	1.814652
H	1.417457	-2.17393	-1.51698
H	-2.07077	-2.47235	-1.19702
H	-2.24671	-0.15038	-1.9216
H	-3.59614	-1.49017	0.627305
H	-4.89144	-0.39769	2.423427
H	-3.16576	-0.38159	2.831582
H	-3.95422	1.093185	2.243295
H	-5.66594	-0.26781	0.02727
H	-4.7313	1.212059	-0.26706

H	-4.50851	-0.20425	-1.30969
H	3.016346	0.873541	2.274431
H	2.45345	-0.78423	2.508677
H	4.179084	-0.44402	2.506937
H	-1.64877	3.361196	-0.37992
H	-0.37311	4.403044	-1.04634
H	-0.88881	2.955513	-1.93284
H	0.827226	3.980895	1.052944
H	-0.0101	-3.21741	1.797294
H	-1.1938	-1.90964	1.677628
H	-1.56002	-3.4934	0.997094
H	-0.07052	-1.21736	-2.69217
05_3R4S13R14S_6			
C	3.258634	-0.46177	0.567702
C	3.165369	0.492163	-0.37475
C	3.489318	-1.90953	0.172915
C	2.96904	1.979525	-0.21362
C	2.262269	-2.84112	0.363042
C	1.743942	2.576512	-0.95443
C	0.396	2.544811	-0.20411
C	-0.09314	1.092781	0.033767
C	-1.349	0.922143	0.848387
C	-2.20711	0.095966	0.241259
C	1.106917	-2.42933	-0.51082
C	-0.19804	-2.35252	-0.21226
C	-1.18678	-1.83252	-1.25827
C	-1.59849	-0.33854	-1.07596
C	-3.57332	-0.36406	0.69436
C	-3.87888	0.018778	2.148603
C	-4.6773	0.162151	-0.24945
C	3.149452	-0.20958	2.053171
C	-0.64512	3.376666	-0.9599
O	0.651999	3.126248	1.089985
C	-0.79633	-2.7796	1.108171
O	-0.69733	-1.94285	-2.59546
O	-0.41902	0.46846	-1.22643
H	3.263747	0.171266	-1.41457
H	4.321982	-2.32188	0.761655
H	3.79688	-1.95587	-0.87944
H	2.926058	2.27538	0.837551
H	3.85929	2.473797	-0.6311
H	1.979288	-2.8767	1.420285
H	2.58365	-3.86061	0.09839
H	1.957787	3.631502	-1.16576
H	1.610771	2.080618	-1.92373
H	0.733581	0.549885	0.50605
H	-1.47717	1.36492	1.829872
H	1.400155	-2.15925	-1.52375
H	-2.09792	-2.44556	-1.22658
H	-2.25926	-0.1182	-1.9266
H	-3.59158	-1.46269	0.627434
H	-4.85505	-0.37685	2.449371
H	-3.12524	-0.37839	2.83755
H	-3.9092	1.108228	2.271804
H	-5.65671	-0.21601	0.064461
H	-4.71247	1.257826	-0.22883
H	-4.51517	-0.15137	-1.28605
H	2.910761	0.828473	2.295154
H	2.371239	-0.84055	2.502906
H	4.089409	-0.46918	2.560479
H	-1.60507	3.381869	-0.4284
H	-0.29957	4.410893	-1.0599
H	-0.82202	2.965665	-1.95779
H	-0.20583	3.351907	1.483468
H	-0.05912	-3.2555	1.759253
H	-1.2319	-1.93723	1.657686
H	-1.60589	-3.50467	0.944441
H	-0.08526	-1.19415	-2.70696
05_3R4S13R14S_7			
C	3.144737	0.607676	-0.78714
C	2.66396	-0.51454	-1.35066
C	2.6675	1.960739	-1.2835
C	2.893973	-1.95672	-0.9718
C	2.01655	2.884501	-0.21169
C	1.594329	-2.75973	-0.69685
C	0.73292	-2.34542	0.517794
C	0.013087	-1.01065	0.24022
C	-1.14458	-1.03735	-0.72757
C	-2.17127	-0.32651	-0.24986
C	0.841131	2.313417	0.554852
C	-0.45228	2.304796	0.187641
C	-1.52483	1.762878	1.138333
C	-1.76364	0.227519	1.108423
C	-3.52916	-0.07773	-0.8644
C	-3.60444	-0.50342	-2.33711
C	-4.64148	-0.76649	-0.04312
C	4.146183	0.638303	0.343129
C	1.57314	-2.25176	1.800902

O	-0.23309	-3.40218	0.668466
C	-0.9632	2.857183	-1.12151
O	-1.2467	2.061308	2.50831
O	-0.58274	-0.49951	1.452877
H	1.960719	-0.38595	-2.17713
H	3.518256	2.519408	-1.70456
H	1.952345	1.812009	-2.10099
H	3.399526	-2.46788	-1.80495
H	3.567872	-2.04148	-0.11408
H	1.722547	3.808255	-0.72542
H	2.786867	3.177455	0.512037
H	0.964426	-2.7489	-1.59592
H	1.871512	-3.8087	-0.53458
H	0.767724	-0.29878	-0.09708
H	-1.10526	-1.53883	-1.68721
H	1.074101	1.871123	1.522037
H	-2.48515	2.228344	0.852527
H	-2.5076	0.030916	1.892614
H	-3.72256	1.005227	-0.81951
H	-4.58827	-0.26138	-2.75391
H	-2.84458	0.003165	-2.9427
H	-3.45477	-1.58462	-2.44289
H	-5.62576	-0.5383	-0.46783
H	-4.50909	-1.85479	-0.05435
H	-4.64332	-0.43719	1.001402
H	4.554202	-0.34776	0.576884
H	4.989193	1.29614	0.09009
H	3.703758	1.041866	1.263821
H	0.921737	-2.08422	2.663086
H	2.121999	-3.18778	1.952766
H	2.291494	-1.42634	1.763217
H	-0.89977	-3.06264	1.289907
H	-0.17147	3.319416	-1.71689
H	-1.41409	2.065456	-1.73313
H	-1.74597	3.609649	-0.95381
H	-0.90637	2.97001	2.53271

05_3R4S13R14S_8

C	3.385224	-0.32339	0.408962
C	3.090936	0.600706	-0.52049
C	3.603926	-1.76973	0.001844
C	2.869859	2.084059	-0.36481
C	2.432697	-2.7277	0.342433
C	1.582228	2.634949	-1.02829
C	0.266138	2.564583	-0.21697
C	-0.14919	1.106033	0.109309
C	-1.44619	0.951863	0.854579
C	-2.2423	0.066958	0.247772
C	1.183906	-2.38758	-0.42684
C	-0.08991	-2.36566	-0.00562
C	-1.19141	-1.96649	-0.9831
C	-1.52516	-0.44785	-0.98991
C	-3.63379	-0.37822	0.636165
C	-4.07286	0.169293	2.001036
C	-4.66264	-0.00714	-0.45417
C	3.531121	-0.0442	1.886147
C	-0.83722	3.306002	-0.97132
O	0.410208	3.259827	1.037283
C	-0.55199	-2.72473	1.386075
O	-0.87415	-2.2765	-2.34259
O	-0.34354	0.353919	-1.09831
H	3.020041	0.257538	-1.55424
H	3.785101	-1.82126	-1.07912
H	4.510857	-2.15392	0.490799
H	2.920195	2.396715	0.685195
H	3.713633	2.597669	-0.85009
H	2.254539	-2.73538	1.42328
H	2.759475	-3.74689	0.082472
H	1.746254	3.696164	-1.2525
H	1.424593	2.128131	-1.98789
H	0.677069	0.654034	0.681824
H	-1.65491	1.496147	1.76742
H	1.354612	-2.11583	-1.4676
H	-2.11791	-2.49582	-0.69983
H	-2.11776	-0.29034	-1.90253
H	-3.62615	-1.47703	0.707388
H	-5.06436	-0.21538	2.263771
H	-3.37516	-0.12007	2.794637
H	-4.13255	1.264225	1.985906
H	-5.65943	-0.3665	-0.17421
H	-4.71587	1.080698	-0.5805
H	-4.40963	-0.4474	-1.42437
H	3.329944	0.996847	2.150759
H	2.853667	-0.67444	2.477935
H	4.549222	-0.28124	2.225521
H	-1.75775	3.335119	-0.38072
H	-0.52805	4.335805	-1.1785
H	-1.04823	2.803399	-1.91932
H	1.084374	2.794863	1.559109

H	0.278714	-2.97424	2.05044
H	-1.11308	-1.90135	1.844225
H	-1.22837	-3.59077	1.360133
H	-0.51341	-3.17794	-2.34738

05_3R4S13R14S_9

C	3.339852	-0.41548	0.495766
C	3.106314	0.503808	-0.45655
C	3.529123	-1.87593	0.123132
C	2.931734	1.997067	-0.3271
C	2.303901	-2.78694	0.403583
C	1.659497	2.590871	-0.98626
C	0.338504	2.540998	-0.17863
C	-0.14316	1.087977	0.057169
C	-1.41198	0.922611	0.849795
C	-2.24578	0.072738	0.24268
C	1.130291	-2.41952	-0.46566
C	-0.17154	-2.34655	-0.15431
C	-1.17453	-1.86839	-1.20669
C	-1.60619	-0.37743	-1.05509
C	-3.6181	-0.39137	0.672827
C	-3.95481	0.007117	2.116005
C	-4.70674	0.115862	-0.29876
C	3.442808	-0.11803	1.973195
C	-0.72926	3.378709	-0.8808
O	0.51513	3.150358	1.114838
C	-0.75092	-2.73594	1.185571
O	-0.69144	-2.00608	-2.544
O	-0.43441	0.44458	-1.19933
H	3.06211	0.148148	-1.48785
H	4.390219	-2.28238	0.672601
H	3.774293	-1.95296	-0.94366
H	3.002993	2.327121	0.715959
H	3.787983	2.472533	-0.82868
H	2.04821	-2.75646	1.468311
H	2.613105	-3.82267	0.193036
H	1.855992	3.650129	-1.19264
H	1.492188	2.105696	-1.95577
H	0.679196	0.548665	0.551465
H	-1.56397	1.415129	1.802456
H	1.405808	-2.18576	-1.49229
H	-2.0766	-2.49333	-1.15525
H	-2.2544	-0.17881	-1.92041
H	-3.62739	-1.49111	0.619366
H	-4.93441	-0.39102	2.402185
H	-3.21213	-0.37711	2.823991
H	-3.99343	1.097642	2.225319
H	-5.68975	-0.266	-0.00076
H	-4.74975	1.211379	-0.29069
H	-4.52157	-0.20759	-1.32851
H	3.235998	0.926085	2.221439
H	2.747099	-0.74128	2.550732
H	4.449862	-0.35335	2.344826
H	-1.65865	3.381458	-0.303
H	-0.38615	4.412882	-0.98779
H	-0.93929	2.971599	-1.87355
H	1.164948	2.623375	1.60754
H	0.004784	-3.15517	1.854456
H	-1.21752	-1.88666	1.697174
H	-1.53298	-3.49743	1.056872
H	-0.08524	-1.25663	-2.67811

05_3R4S13R14S_10

C	3.067222	0.716609	-0.84191
C	2.592378	-0.41888	-1.38324
C	2.541207	2.056433	-1.32511
C	2.874555	-1.8541	-1.01204
C	1.916834	2.966793	-0.22649
C	1.604376	-2.72067	-0.79182
C	0.695373	-2.43137	0.43664
C	0.029194	-1.04833	0.285576
C	-1.0632	-0.93467	-0.75305
C	-2.11453	-0.26656	-0.26403
C	0.836602	2.334491	0.623905
C	-0.48588	2.298426	0.394321
C	-1.44035	1.649222	1.408098
C	-1.78689	0.143699	1.157413
C	-3.43437	0.056794	-0.92424
C	-3.42682	-0.21073	-2.43522
C	-4.58889	-0.71065	-0.24178
C	4.108441	0.77271	0.250494
C	1.481345	-2.51518	1.746884
O	-0.29192	-3.47238	0.524297
C	-1.14203	2.922526	-0.81508
O	-0.96165	1.720259	2.749113
O	-0.63304	-0.63794	1.503447
H	1.858326	-0.30975	-2.18526
H	3.359439	2.630362	-1.78803
H	1.792299	1.886266	-2.10744
H	3.430842	-2.33175	-1.83305

H	3.523794	-1.91955	-0.13335
H	1.537698	3.864051	-0.73078
H	2.717651	3.312933	0.438867
H	0.988613	-2.68019	-1.7021
H	1.918665	-3.76627	-0.68989
H	0.824769	-0.333	0.070068
H	-0.96098	-1.303	-1.76802
H	1.189587	1.872652	1.544412
H	-2.38151	2.215622	1.392198
H	-2.57768	-0.1107	1.875161
H	-3.62992	1.129069	-0.77229
H	-4.3862	0.077719	-2.87801
H	-2.63593	0.35326	-2.94222
H	-3.26995	-1.27561	-2.64531
H	-5.54779	-0.43031	-0.69185
H	-4.4579	-1.79227	-0.36296
H	-4.6467	-0.4956	0.83023
H	4.531	-0.20679	0.486204
H	4.937499	1.430706	-0.04446
H	3.696809	1.190951	1.17879
H	0.798863	-2.40711	2.592904
H	1.980864	-3.4874	1.820812
H	2.239747	-1.72896	1.811064
H	-0.95735	-3.29892	-0.16244
H	-0.45408	3.565312	-1.37118
H	-1.51255	2.163751	-1.5149
H	-2.00665	3.532369	-0.52023
H	-0.39581	0.933215	2.85231

05_3R4S13R14S_11

C	-2.97471	0.507587	1.102365
C	-2.49221	-0.71783	1.372963
C	-2.48008	1.718117	1.876164
C	-2.78897	-2.03228	0.689838
C	-1.74389	2.802204	1.032804
C	-1.53725	-2.84243	0.262133
C	-0.64853	-2.27401	-0.8683
C	0.005543	-0.95087	-0.43702
C	0.972186	-1.01353	0.720446
C	2.066068	-0.29521	0.455054
C	-0.34397	2.438823	0.598202
C	0.130818	2.233808	-0.64328
C	1.604504	1.865426	-0.83332
C	1.889152	0.331881	-0.92
C	3.299866	-0.08445	1.300114
C	3.112755	-0.54445	2.752234
C	4.526176	-0.77456	0.663049
C	-4.02975	0.793481	0.058075
C	-1.44188	-2.08255	-2.17097
O	0.37104	-3.26447	-1.08227
C	-0.68492	2.331907	-1.91054
O	2.15394	2.435166	-2.02876
O	0.818902	-0.41886	-1.51206
H	-1.75525	-0.79147	2.175913
H	-3.34126	2.212755	2.350796
H	-1.81913	1.392112	2.688974
H	-3.34261	-2.67623	1.390231
H	-3.44906	-1.89281	-0.17155
H	-1.67564	3.700347	1.663898
H	-2.3649	3.085126	0.177642
H	-0.90509	-3.02498	1.139488
H	-1.87216	-3.83089	-0.07685
H	-0.79522	-0.23963	-0.2261
H	0.769258	-1.55246	1.637477
H	0.362888	2.339318	1.424953
H	2.169315	2.243038	0.032286
H	2.767474	0.221211	-1.56763
H	3.514157	0.995556	1.317591
H	4.011752	-0.3284	3.33994
H	2.264738	-0.03858	3.226743
H	2.934277	-1.62524	2.803342
H	5.427912	-0.56979	1.251543
H	4.380648	-1.8606	0.627826
H	4.70688	-0.42461	-0.35889
H	-4.45006	-0.11311	-0.38282
H	-4.85864	1.362818	0.500811
H	-3.63634	1.407484	-0.76228
H	-0.77077	-1.77329	-2.97794
H	-1.91707	-3.027	-2.4576
H	-2.21923	-1.317	-2.07165
H	1.062836	-2.81142	-1.59562
H	-1.73059	2.577342	-1.71437
H	-0.27984	3.096727	-2.58327
H	-0.64317	1.383705	-2.45633
H	2.057408	3.398919	-1.96198

05_3R4S13R14S_12

C	3.144147	0.632145	-0.77695
C	2.680198	-0.49542	-1.344
C	2.662073	1.979979	-1.2822

C	2.917246	-1.93424	-0.95815
C	1.994177	2.901589	-0.21904
C	1.621636	-2.75247	-0.69856
C	0.728956	-2.37297	0.516491
C	0.025736	-1.02399	0.247684
C	-1.13187	-1.04161	-0.72382
C	-2.16041	-0.33571	-0.23996
C	0.823382	2.319289	0.545431
C	-0.472	2.312083	0.184893
C	-1.53713	1.761752	1.13827
C	-1.74785	0.221081	1.114864
C	-3.51723	-0.08519	-0.85467
C	-3.59536	-0.51205	-2.32679
C	-4.62853	-0.77134	-0.02938
C	4.129325	0.672701	0.36716
C	1.540077	-2.31543	1.812442
O	-0.23055	-3.42537	0.722302
C	-0.9917	2.874182	-1.11674
O	-1.26051	2.068035	2.506777
O	-0.55038	-0.47704	1.446972
H	1.987298	-0.3747	-2.18048
H	3.512418	2.543907	-1.69703
H	1.955534	1.823572	-2.10578
H	3.440922	-2.44214	-1.78227
H	3.57819	-2.01129	-0.08953
H	1.691389	3.818858	-0.73898
H	2.757342	3.207553	0.507011
H	1.004841	-2.73752	-1.60908
H	1.908669	-3.79973	-0.54623
H	0.794483	-0.3355	-0.10819
H	-1.0882	-1.51277	-1.70012
H	1.061366	1.868971	1.507616
H	-2.50418	2.21332	0.853427
H	-2.48315	0.011992	1.904513
H	-3.70825	0.997874	-0.80965
H	-4.57948	-0.26853	-2.74167
H	-2.83604	-0.0073	-2.93451
H	-3.44854	-1.59378	-2.43222
H	-5.61256	-0.54356	-0.45448
H	-4.49682	-1.85969	-0.037
H	-4.62858	-0.43911	1.013944
H	4.543262	-0.30945	0.606889
H	4.969567	1.338465	0.125956
H	3.66998	1.071929	1.281391
H	0.867159	-2.15274	2.656727
H	2.073312	-3.26112	1.961601
H	2.271088	-1.50183	1.792533
H	-0.93516	-3.30744	0.063776
H	-0.20264	3.333526	-1.71779
H	-1.45429	2.089256	-1.72868
H	-1.76766	3.631221	-0.93821
H	-0.91906	2.976379	2.52618

05_3R4S13R14S_13

C	2.837804	-0.68892	1.137339
C	2.459227	0.569615	1.420281
C	2.233339	-1.86205	1.890718
C	2.861482	1.862488	0.749097
C	1.448241	-2.89155	1.022905
C	1.674857	2.772145	0.333753
C	0.753737	2.301302	-0.81614
C	0.060068	0.976692	-0.45899
C	-0.84566	0.982635	0.747899
C	-1.97924	0.326751	0.489373
C	0.108977	-2.42259	0.503502
C	-0.26683	-2.18677	-0.76523
C	-1.69637	-1.71337	-1.07119
C	-1.91206	-0.17206	-0.94176
C	-3.16278	0.069745	1.392138
C	-2.87229	0.405628	2.861073
C	-4.41421	0.827181	0.896374
C	3.873807	-1.04753	0.096507
C	1.524878	2.162574	-2.13876
O	-0.23554	3.334092	-0.95599
C	0.627653	-2.35267	-1.97064
O	-2.14065	-2.14987	-2.36075
O	-0.8295	0.569983	-1.53243
H	1.72921	0.695397	2.222266
H	3.04096	-2.41964	2.389457
H	1.573619	-1.49025	2.684631
H	3.464871	2.45424	1.454319
H	3.50794	1.679242	-0.11488
H	1.270641	-3.76877	1.661831
H	2.089382	-3.24323	0.208902
H	1.048798	2.974013	1.211115
H	2.08118	3.743887	0.026001
H	0.834459	0.21534	-0.34547
H	-0.57271	1.432614	1.693598
H	-0.64095	-2.27566	1.283365

H	-2.38886	-2.19444	-0.37157
H	-2.81932	0.053189	-1.51777
H	-3.39664	-1.00507	1.336117
H	-3.7347	0.154349	3.488213
H	-2.00527	-0.14991	3.235059
H	-2.66942	1.47588	2.987827
H	-5.27862	0.588126	1.526276
H	-4.25036	1.910375	0.9388
H	-4.67011	0.564233	-0.13553
H	4.389773	-0.17376	-0.30766
H	4.634547	-1.71125	0.529966
H	3.433636	-1.59154	-0.74885
H	0.838124	1.903568	-2.9506
H	2.010251	3.112832	-2.38567
H	2.29257	1.382386	-2.08738
H	-0.95044	2.931392	-1.47978
H	1.622665	-2.71499	-1.70727
H	0.187959	-3.06204	-2.68101
H	0.746867	-1.39805	-2.49946
H	-1.66246	-1.62161	-3.02099

05_3R4S13R14S_14

C	3.033701	-0.45809	1.062282
C	2.528037	0.755338	1.344207
C	2.590474	-1.67816	1.851096
C	2.774901	2.075703	0.653359
C	1.854518	-2.77695	1.028194
C	1.494204	2.868949	0.278055
C	0.56637	2.327146	-0.84725
C	-0.03455	0.972767	-0.42414
C	-1.03134	1.021498	0.710777
C	-2.09645	0.264549	0.428897
C	0.441428	-2.43525	0.621242
C	-0.06538	-2.26061	-0.61214
C	-1.54934	-1.92333	-0.77133
C	-1.85452	-0.39597	-0.92058
C	-3.34468	0.032002	1.245367
C	-3.20607	0.504175	2.699007
C	-4.56792	0.690742	0.569816
C	4.063069	-0.72034	-0.01317
C	1.309697	2.201686	-2.17872
O	-0.46774	3.299291	-1.07846
C	0.71928	-2.37367	-1.89734
O	-2.12584	-2.55141	-1.9242
O	-0.77689	0.350305	-1.49258
H	1.811894	0.812273	2.167483
H	3.475695	-2.15328	2.300736
H	1.946003	-1.36633	2.682569
H	3.346848	2.726152	1.333087
H	3.401785	1.949197	-0.23455
H	1.812741	-3.67354	1.66399
H	2.462121	-3.0536	0.161416
H	0.891649	3.017405	1.184911
H	1.798964	3.872241	-0.04344
H	0.802355	0.322246	-0.15995
H	-0.86305	1.565084	1.633398
H	-0.24692	-2.3295	1.463044
H	-2.08019	-2.27512	0.125853
H	-2.71169	-0.32103	-1.60165
H	-3.53513	-1.05198	1.263344
H	-4.11468	0.269631	3.264224
H	-2.35925	0.020919	3.198488
H	-3.05537	1.589334	2.749938
H	-5.48087	0.464533	1.132343
H	-4.44853	1.780063	0.53635
H	-4.70854	0.334885	-0.45614
H	4.457018	0.195802	-0.4587
H	4.9126	-1.28023	0.401376
H	3.655321	-1.33396	-0.82685
H	0.610938	1.900674	-2.96273
H	1.750618	3.166422	-2.45348
H	2.110102	1.45721	-2.12305
H	-1.14933	3.159943	-0.39946
H	1.771605	-2.60815	-1.72419
H	0.302245	-3.15215	-2.54658
H	0.655293	-1.43392	-2.45518
H	-2.01098	-3.50991	-1.82047

05_3R4S13R14S_15

C	2.87124	-0.68001	1.11134
C	2.474725	0.573813	1.38988
C	2.287122	-1.8575	1.872951
C	2.854737	1.869227	0.711676
C	1.500375	-2.89214	1.013706
C	1.654898	2.784994	0.346258
C	0.6876	2.361188	-0.79774
C	0.041152	1.002489	-0.46931
C	-0.8646	0.981195	0.740318
C	-1.98453	0.301258	0.477557
C	0.154432	-2.43147	0.504308

C	-0.2316	-2.19898	-0.76188
C	-1.66963	-1.74814	-1.05953
C	-1.89619	-0.20544	-0.94967
C	-3.16232	0.018172	1.379215
C	-2.88576	0.367736	2.847609
C	-4.431	0.740183	0.87389
C	3.909453	-1.02717	0.069027
C	1.403999	2.30795	-2.14853
O	-0.31192	3.38481	-0.93426
C	0.657874	-2.35241	-1.97249
O	-2.11961	-2.20534	-2.33971
O	-0.8158	0.534069	-1.5348
H	1.747538	0.692161	2.195836
H	3.104909	-2.40815	2.362671
H	1.633128	-1.49061	2.673911
H	3.486891	2.455298	1.396541
H	3.467257	1.689575	-0.17767
H	1.332619	-3.76974	1.654761
H	2.136183	-3.24124	0.194477
H	1.058794	2.96177	1.251836
H	2.049747	3.76622	0.056108
H	0.854152	0.283836	-0.33769
H	-0.59756	1.418944	1.694496
H	-0.59259	-2.29387	1.288968
H	-2.3496	-2.2304	-0.3487
H	-2.80217	0.006559	-1.53376
H	-3.3678	-1.06223	1.32667
H	-3.74406	0.097033	3.472078
H	-2.00668	-0.16419	3.227576
H	-2.71125	1.44341	2.971453
H	-5.29042	0.482614	1.50304
H	-4.29595	1.827666	0.909708
H	-4.6759	0.464153	-0.15702
H	4.397224	-0.14635	-0.35439
H	4.692035	-1.66067	0.508956
H	3.479881	-1.59925	-0.76301
H	0.690301	2.058045	-2.93732
H	1.848176	3.283394	-2.3748
H	2.199959	1.556297	-2.14889
H	-0.98363	3.221806	-0.25051
H	1.660514	-2.69738	-1.71493
H	0.225839	-3.07024	-2.67912
H	0.75651	-1.39605	-2.50188
H	-1.65726	-1.67656	-3.01081

05_3R4S13S14R_3

C	-2.80822	0.879468	1.117618
C	-2.42092	-0.35176	1.494128
C	-2.1413	2.109711	1.711221
C	-2.88569	-1.6983	0.989029
C	-1.37283	3.017289	0.702304
C	-1.74396	-2.67926	0.611612
C	-0.87546	-2.35535	-0.62588
C	-0.12199	-1.02832	-0.4397
C	0.850848	-0.9479	0.709429
C	1.985261	-0.35251	0.330681
C	-0.07989	2.442347	0.175262
C	0.209298	2.049085	-1.07741
C	1.600018	1.523762	-1.44152
C	1.84344	0.007065	-1.14196
C	3.227466	-0.09067	1.151733
C	3.046261	-0.46035	2.630218
C	4.451736	-0.82081	0.55845
C	-3.91931	1.154655	0.130727
C	-1.71567	-2.31802	-1.91286
O	0.07349	-3.43079	-0.71722
C	-0.74502	2.094664	-2.24758
O	2.662889	2.223875	-0.7913
O	0.719402	-0.7667	-1.593
H	-1.63578	-0.40931	2.250555
H	-2.90981	2.74065	2.183226
H	-1.45199	1.80631	2.509028
H	-3.46118	-2.1947	1.785323
H	-3.57583	-1.59101	0.146461
H	-1.13701	3.949446	1.235876
H	-2.04303	3.302428	-0.11451
H	-1.07765	-2.80834	1.472784
H	-2.18937	-3.6653	0.427864
H	-0.86532	-0.232	-0.36694
H	0.628308	-1.31141	1.704142
H	0.713487	2.352222	0.917259
H	1.707839	1.633967	-2.5329
H	2.714772	-0.30063	-1.7326
H	3.432827	0.984435	1.08836
H	3.949559	-0.20933	3.197519
H	2.204653	0.078711	3.079717
H	2.864058	-1.53516	2.753509
H	5.347856	-0.60075	1.150227
H	4.300393	-1.90721	0.563704

H	4.650685	-0.51015	-0.47255
H	-4.41882	0.246273	-0.21326
H	-4.68214	1.799672	0.588966
H	-3.55695	1.689041	-0.75644
H	-1.06894	-2.16102	-2.78165
H	-2.24082	-3.27081	-2.0398
H	-2.45713	-1.51133	-1.89635
H	0.777906	-3.09616	-1.30005
H	-1.73946	2.456175	-1.9785
H	-0.35454	2.747168	-3.04109
H	-0.85186	1.096198	-2.68806
H	2.474655	3.171973	-0.88067

05_3R4S13S14R_4

C	-2.85604	0.865255	1.08707
C	-2.44065	-0.36095	1.448897
C	-2.21299	2.102468	1.691238
C	-2.87657	-1.71155	0.92972
C	-1.44304	3.017158	0.690415
C	-1.71442	-2.69462	0.62449
C	-0.78671	-2.4276	-0.59632
C	-0.09454	-1.06046	-0.44686
C	0.875395	-0.94056	0.704591
C	1.993569	-0.31551	0.32002
C	-0.13906	2.456284	0.175138
C	0.158507	2.053259	-1.07228
C	1.561858	1.562702	-1.43501
C	1.831138	0.046731	-1.15015
C	3.226266	-0.01272	1.140078
C	3.073718	-0.42109	2.611516
C	4.47981	-0.66873	0.522071
C	-3.97756	1.125315	0.108186
C	-1.55613	-2.50549	-1.91673
O	0.183557	-3.48729	-0.65294
C	-0.79808	2.061461	-2.24129
O	2.607709	2.281318	-0.77807
O	0.717732	-0.73684	-1.59618
H	-1.65529	-0.40916	2.206031
H	-2.99411	2.724256	2.154487
H	-1.52932	1.806346	2.496633
H	-3.49734	-2.20185	1.695606
H	-3.5182	-1.61072	0.048508
H	-1.22189	3.952281	1.224941
H	-2.10761	3.294542	-0.13369
H	-1.0845	-2.78496	1.519687
H	-2.14332	-3.69123	0.463334
H	-0.88269	-0.30838	-0.35625
H	0.658933	-1.28833	1.707375
H	0.655865	2.394256	0.918576
H	1.669463	1.682947	-2.5253
H	2.704387	-0.24309	-1.74765
H	3.378237	1.072081	1.096686
H	3.969729	-0.14179	3.176832
H	2.213458	0.070609	3.079525
H	2.940758	-1.50544	2.714023
H	5.367217	-0.4216	1.115981
H	4.380744	-1.76103	0.499376
H	4.657315	-0.32168	-0.50092
H	-4.42657	0.208086	-0.27929
H	-4.77527	1.706047	0.592616
H	-3.64319	1.71953	-0.75129
H	-0.87088	-2.34965	-2.75351
H	-2.01823	-3.4933	-2.02139
H	-2.34457	-1.74744	-1.96739
H	0.917105	-3.23305	-0.0675
H	-1.80615	2.380607	-1.96996
H	-0.43582	2.730011	-3.03493
H	-0.8627	1.059206	-2.68045
H	2.405568	3.226126	-0.87195

05_3R4S13S14R_5

C	-3.49123	0.116806	-0.35127
C	-2.75002	1.069705	-0.94236
C	-3.74501	-1.21194	-1.03849
C	-2.4433	2.455223	-0.4111
C	-3.02624	-2.43677	-0.40729
C	-1.3474	2.586684	0.679022
C	0.130238	2.558866	0.236858
C	0.5365	1.18191	-0.35365
C	1.993726	0.98444	-0.68528
C	2.460925	-0.14096	-0.1358
C	-1.53267	-2.32665	-0.54739
C	-0.57757	-2.46915	0.381409
C	0.874142	-2.1733	0.01963
C	1.324282	-0.80468	0.625125
C	3.836686	-0.75342	-0.2336
C	4.738005	-0.0329	-1.2449
C	4.518957	-0.82491	1.149737
C	-4.13064	0.277949	1.007806
C	1.018599	2.94116	1.427389

O	0.25883	3.538934	-0.81042
C	-0.80993	-2.83717	1.825352
O	1.110962	-2.13352	-1.38559
O	0.267635	0.154717	0.607666
H	-2.33694	0.837331	-1.92448
H	-4.82573	-1.41886	-1.0357
H	-3.44242	-1.14207	-2.09119
H	-2.17669	3.109087	-1.24836
H	-3.35892	2.880137	0.021374
H	-3.38657	-3.33269	-0.93635
H	-3.33122	-2.55432	0.63795
H	-1.49884	3.554531	1.174656
H	-1.4866	1.812259	1.439935
H	-0.08216	1.027441	-1.25084
H	2.546352	1.648032	-1.34174
H	-1.20471	-2.03018	-1.54333
H	1.520938	-2.9437	0.475677
H	1.57497	-0.99324	1.678526
H	3.698611	-1.78602	-0.58634
H	5.706133	-0.54003	-1.32273
H	4.282787	-0.01345	-2.24087
H	4.929074	1.002829	-0.93779
H	5.493457	-1.32017	1.069421
H	4.681913	0.181273	1.554284
H	3.919266	-1.38573	1.874967
H	-4.0526	1.298015	1.393099
H	-5.1953	0.00833	0.973328
H	-3.66419	-0.38679	1.74764
H	2.073775	2.990953	1.132919
H	0.723329	3.919514	1.820435
H	0.932507	2.200096	2.227961
H	1.206338	3.694782	-0.95095
H	-1.84283	-3.13605	2.018583
H	-0.15973	-3.66861	2.129991
H	-0.57836	-1.99419	2.490434
H	0.766537	-2.96489	-1.75102

05_3R4S13S14R_6

C	-3.44454	0.401886	0.120516
C	-2.68116	1.399762	-0.34894
C	-4.00828	-0.6426	-0.82395
C	-2.03713	2.526667	0.428878
C	-3.39747	-2.06149	-0.64606
C	-0.83241	3.20081	-0.27049
C	0.607306	2.67146	-0.0482
C	0.860192	1.26466	-0.65908
C	2.292016	0.803612	-0.63962
C	2.398006	-0.40239	-0.07619
C	-1.90675	-2.03055	-0.83423
C	-0.94082	-2.42711	0.006298
C	0.517116	-2.14603	-0.33756
C	1.014414	-0.85193	0.358689
C	3.628551	-1.25477	0.120472
C	4.875612	-0.6567	-0.54454
C	3.891524	-1.53171	1.616455
C	-3.79077	0.220628	1.578816
C	1.019391	2.73334	1.420453
O	1.494381	3.583236	-0.73502
C	-1.15542	-3.09985	1.339717
O	0.760894	-1.99544	-1.73227
O	0.154865	0.251984	0.063759
H	-2.51391	1.435976	-1.42713
H	-5.09761	-0.72452	-0.69035
H	-3.84092	-0.32251	-1.86015
H	-2.78968	3.316111	0.581738
H	-1.75199	2.198851	1.433827
H	-3.86354	-2.71849	-1.39595
H	-3.67436	-2.46732	0.33222
H	-1.02207	3.216939	-1.35548
H	-0.79345	4.251471	0.041776
H	0.482932	1.296757	-1.69845
H	3.092888	1.39337	-1.06717
H	-1.58087	-1.54632	-1.75422
H	1.135379	-2.97048	0.06172
H	0.969435	-1.03942	1.442433
H	3.427607	-2.22451	-0.36078
H	5.732761	-1.32815	-0.42218
H	4.718977	-0.49648	-1.61658
H	5.138482	0.307265	-0.0923
H	4.761232	-2.18792	1.735919
H	4.095427	-0.59682	2.152211
H	3.038349	-2.01823	2.101594
H	-3.39463	1.019195	2.210721
H	-4.88089	0.193886	1.71859
H	-3.40471	-0.73183	1.966181
H	2.084069	2.50407	1.525529
H	0.840857	3.736679	1.821245
H	0.452917	2.01014	2.012207
H	1.190376	3.643846	-1.65651

H	-2.21123	-3.27485	1.558431
H	-0.64232	-4.07102	1.373798
H	-0.7416	-2.50172	2.163397
H	0.304648	-2.7284	-2.17676

05_3R4S13S14R_7

C	-3.46797	0.405249	0.036721
C	-2.57471	1.301247	-0.41136
C	-3.98577	-0.69987	-0.86502
C	-1.979	2.464229	0.357448
C	-3.44669	-2.12424	-0.53233
C	-0.75505	3.151855	-0.29436
C	0.683889	2.628165	-0.03622
C	0.981958	1.301409	-0.78117
C	2.392729	0.785999	-0.69208
C	2.428051	-0.42667	-0.13017
C	-1.96714	-2.21262	-0.77905
C	-0.97828	-2.33677	0.115087
C	0.475501	-2.14817	-0.31617
C	1.017087	-0.81752	0.270179
C	3.616024	-1.32241	0.123876
C	4.920251	-0.7583	-0.45494
C	3.776271	-1.636	1.627002
C	-4.03274	0.420779	1.438436
C	1.006906	2.536684	1.457688
O	1.592054	3.548193	-0.67736
C	-1.17845	-2.5648	1.595671
O	0.668476	-2.15608	-1.72337
O	0.187761	0.234793	-0.24982
H	-2.24912	1.212183	-1.4482
H	-5.0843	-0.73638	-0.81047
H	-3.7309	-0.4717	-1.9079
H	-2.75141	3.241614	0.462652
H	-1.73431	2.165934	1.383402
H	-3.97973	-2.83702	-1.17815
H	-3.70459	-2.38568	0.499271
H	-0.89937	3.207083	-1.3819
H	-0.74554	4.194254	0.057094
H	0.691131	1.467914	-1.83081
H	3.235904	1.349761	-1.07116
H	-1.6779	-2.07023	-1.81953
H	1.087893	-2.97198	0.07516
H	0.917906	-0.85329	1.365653
H	3.408626	-2.27531	-0.3875
H	5.743588	-1.46328	-0.29574
H	4.834337	-0.5748	-1.53132
H	5.190402	0.187438	0.030277
H	4.616358	-2.3217	1.784669
H	3.97489	-0.71935	2.195299
H	2.880216	-2.10579	2.047208
H	-3.60693	1.213925	2.058319
H	-5.12249	0.565887	1.415143
H	-3.86075	-0.53331	1.954624
H	2.066	2.303751	1.601056
H	0.794021	3.491925	1.95375
H	0.411965	1.761993	1.950007
H	1.438735	4.424544	-0.28809
H	-2.16853	-2.97002	1.819545
H	-0.43591	-3.27307	1.986985
H	-1.06602	-1.63692	2.172723
H	0.342272	-1.29313	-2.03319

05_3R4S13S14R_8

C	-3.43318	0.358274	0.043965
C	-2.6983	1.370747	-0.43982
C	-3.95441	-0.72599	-0.88002
C	-2.12931	2.552096	0.313687
C	-3.33637	-2.13134	-0.63685
C	-0.88644	3.213298	-0.32209
C	0.524872	2.661738	-0.02026
C	0.788077	1.255015	-0.6368
C	2.231824	0.82653	-0.67314
C	2.397429	-0.36015	-0.08294
C	-1.84298	-2.09111	-0.79436
C	-0.89016	-2.44735	0.078417
C	0.569763	-2.16173	-0.25133
C	1.044969	-0.84001	0.412718
C	3.658644	-1.17413	0.075272
C	4.849477	-0.57928	-0.6884
C	4.013576	-1.37499	1.564569
C	-3.80253	0.202391	1.499901
C	0.851063	2.695928	1.475052
O	1.378155	3.591142	-0.72794
C	-1.12241	-3.06888	1.43324
O	0.830187	-2.05194	-1.64685
O	0.143971	0.232392	0.13131
H	-2.51159	1.385184	-1.51538
H	-5.04643	-0.81513	-0.77441
H	-3.76125	-0.43908	-1.92149
H	-2.90332	3.335147	0.351798

H	-1.92657	2.296067	1.358682
H	-3.77938	-2.81935	-1.37276
H	-3.63089	-2.50482	0.349248
H	-1.00593	3.237212	-1.41408
H	-0.85514	4.261794	-0.0017
H	0.369055	1.270142	-1.65656
H	3.000606	1.395727	-1.18295
H	-1.50428	-1.63523	-1.72417
H	1.192073	-2.96638	0.180711
H	1.040911	-1.01355	1.499834
H	3.454313	-2.16834	-0.35009
H	5.728499	-1.22543	-0.5876
H	4.626403	-0.47206	-1.75529
H	5.116244	0.409761	-0.29597
H	4.906135	-2.00341	1.662251
H	4.222299	-0.41153	2.045138
H	3.202692	-1.85903	2.119608
H	-3.44894	1.029686	2.11966
H	-4.89366	0.13919	1.618856
H	-3.39037	-0.72533	1.918736
H	1.894794	2.405785	1.648235
H	0.704386	3.707686	1.867988
H	0.220899	2.002189	2.036962
H	2.286054	3.434486	-0.4225
H	-2.17838	-3.26865	1.629257
H	-0.58016	-4.02016	1.525289
H	-0.75271	-2.42122	2.24041
H	0.383492	-2.80057	-2.07469

05_3R4S13S14R_9

C	-3.43734	0.386483	0.092327
C	-2.68968	1.391105	-0.38836
C	-3.98662	-0.67598	-0.84097
C	-2.07748	2.546	0.372534
C	-3.3755	-2.0907	-0.63548
C	-0.84788	3.200163	-0.30135
C	0.580729	2.657242	-0.04082
C	0.839394	1.262623	-0.67166
C	2.275413	0.813261	-0.66517
C	2.398342	-0.38421	-0.08715
C	-1.88412	-2.06023	-0.81632
C	-0.92152	-2.43398	0.038249
C	0.536788	-2.15343	-0.30436
C	1.023725	-0.84182	0.366606
C	3.638932	-1.22252	0.107337
C	4.870926	-0.62719	-0.5875
C	3.925947	-1.46698	1.604612
C	-3.78784	0.217235	1.551149
C	0.945609	2.685448	1.446208
O	1.486944	3.504798	-0.78098
C	-1.14065	-3.07618	1.385855
O	0.786256	-2.03216	-1.70069
O	0.148811	0.246027	0.058394
H	-2.51826	1.415603	-1.46617
H	-5.07714	-0.7583	-0.71668
H	-3.81024	-0.37157	-1.88038
H	-2.83785	3.337977	0.462756
H	-1.83477	2.259344	1.40087
H	-3.83638	-2.76051	-1.37717
H	-3.65741	-2.48091	0.347766
H	-0.99749	3.224362	-1.38923
H	-0.82049	4.2521	0.020457
H	0.452238	1.301795	-1.70355
H	3.064884	1.403043	-1.11292
H	-1.5552	-1.59537	-1.74518
H	1.157559	-2.96559	0.115784
H	0.988292	-1.0123	1.453795
H	3.439707	-2.20315	-0.35173
H	5.735546	-1.28904	-0.46505
H	4.696758	-0.48888	-1.65983
H	5.132384	0.347558	-0.15802
H	4.802779	-2.11356	1.724897
H	4.129722	-0.51959	2.118073
H	3.083896	-1.95019	2.112058
H	-3.41189	1.031305	2.175478
H	-4.87793	0.16982	1.685469
H	-3.38439	-0.72212	1.95235
H	1.997256	2.415445	1.581508
H	0.792329	3.691335	1.857006
H	0.333818	1.984594	2.020752
H	1.332515	4.41691	-0.48598
H	-2.19654	-3.25648	1.600075
H	-0.61819	-4.04091	1.448109
H	-0.7398	-2.45361	2.197798
H	0.3351	-2.77644	-2.13135

05_3R4S13S14R_10

C	-3.46133	0.386548	0.019986
C	-2.58804	1.297591	-0.43758
C	-3.96557	-0.73125	-0.87423

C	-2.01723	2.482261	0.316368	H	1.980133	2.29024	1.65748
C	-3.42069	-2.14978	-0.52679	H	0.703278	3.485217	1.970362
C	-0.78204	3.166847	-0.31339	H	0.322777	1.748789	1.945537
C	0.642364	2.634868	-0.02237	H	2.396073	3.476019	-0.30812
C	0.951119	1.306873	-0.77013	H	-2.147	-2.98204	1.832973
C	2.369913	0.804985	-0.69724	H	-0.41477	-3.28509	2.00438
C	2.427808	-0.40552	-0.13133	H	-1.04512	-1.64846	2.186276
C	-1.9399	-2.23264	-0.76736	H	0.360876	-1.31227	-2.02153
C	-0.95343	-2.35158	0.130084	05_3R4S13S14R_11			
C	0.500203	-2.15369	-0.29728	C	-2.80888	0.878289	1.11747
C	1.025651	-0.81344	0.282875	C	-2.42042	-0.3526	1.493904
C	3.628432	-1.28823	0.105976	C	-2.14264	2.109115	1.710664
C	4.919957	-0.71048	-0.48745	C	-2.88439	-1.69958	0.989258
C	3.80891	-1.60213	1.606859	C	-1.37476	3.016807	0.701367
C	-4.02247	0.398983	1.423294	C	-1.74207	-2.68003	0.612311
C	0.925641	2.53235	1.478431	C	-0.87366	-2.35607	-0.6252
O	1.490476	3.64313	-0.61565	C	-0.12101	-1.02849	-0.43955
C	-1.15644	-2.57732	1.610499	C	0.851839	-0.94707	0.709488
O	0.699087	-2.16763	-1.70374	C	1.98585	-0.35112	0.330411
O	0.178636	0.224509	-0.23407	C	-0.08149	2.442485	0.174482
H	-2.26654	1.208553	-1.47598	C	0.207771	2.048823	-1.07806
H	-5.0642	-0.77433	-0.82497	C	1.598871	1.524511	-1.44216
H	-3.70725	-0.51056	-1.91787	C	1.843596	0.008029	-1.14229
H	-2.79603	3.258239	0.376402	C	3.228232	-0.08876	1.151003
H	-1.80357	2.214418	1.357377	C	3.046313	-0.45415	2.630466
H	-3.94759	-2.87076	-1.16855	C	4.451449	-0.82248	0.559911
H	-3.68116	-2.40376	0.505957	C	-3.92079	1.152546	0.131277
H	-0.90629	3.215944	-1.40403	C	-1.71369	-2.31981	-1.91232
H	-0.7625	4.207748	0.03125	O	0.075978	-3.43098	-0.71583
H	0.649572	1.470351	-1.81703	C	-0.74684	2.093	-2.24803
H	3.208018	1.355907	-1.10919	O	2.661182	2.225839	-0.7923
H	-1.64819	-2.09073	-1.80721	O	0.720057	-0.7667	-1.59302
H	1.118988	-2.96946	0.100598	H	-1.63488	-0.40943	2.249973
H	0.936615	-0.84815	1.379378	H	-2.91148	2.73975	2.182531
H	3.423332	-2.2419	-0.40443	H	-1.45308	1.806361	2.508491
H	5.751817	-1.40748	-0.33864	H	-3.45968	-2.19599	1.785687
H	4.820255	-0.52707	-1.56267	H	-3.57452	-1.59301	0.146601
H	5.187178	0.237013	-0.00375	H	-1.13951	3.949312	1.234575
H	4.657025	-2.28022	1.753166	H	-2.04517	3.301174	-0.11554
H	4.005967	-0.68467	2.174399	H	-1.07578	-2.80838	1.473609
H	2.922233	-2.0807	2.036686	H	-2.18688	-3.6664	0.428903
H	-3.61083	1.203435	2.037979	H	-0.86486	-0.23263	-0.36697
H	-5.11486	0.523171	1.40202	H	0.629596	-1.31033	1.704362
H	-3.83106	-0.54885	1.944076	H	0.712066	2.353233	0.916388

H	1.706497	1.634539	-2.53357
H	2.715069	-0.29912	-1.73301
H	3.435134	0.985859	1.08475
H	3.949766	-0.20254	3.197266
H	2.205164	0.087045	3.078256
H	2.862984	-1.52843	2.75673
H	5.34796	-0.60184	1.150884
H	4.298497	-1.90864	0.56864
H	4.650785	-0.51551	-0.47213
H	-4.41798	0.243584	-0.21453
H	-4.68527	1.794589	0.59098
H	-3.56001	1.689782	-0.75479
H	-1.06695	-2.16279	-2.78111
H	-2.2383	-3.27295	-2.03893
H	-2.4556	-1.51353	-1.89628
H	0.779562	-3.09677	-1.29989
H	-1.74139	2.454255	-1.979
H	-0.35685	2.745027	-3.04217
H	-0.85329	1.09412	-2.68766
H	2.470941	3.173669	-0.88024

05_3R4S13S14R_12

C	-3.44656	0.441338	0.097655
C	-2.58441	1.357925	-0.3693
C	-4.01436	-0.63036	-0.81432
C	-1.94139	2.492428	0.402795
C	-3.47943	-2.06929	-0.54593
C	-0.7236	3.169467	-0.27198
C	0.714644	2.636852	-0.03367
C	1.004862	1.307213	-0.78297
C	2.405919	0.772016	-0.66959
C	2.413673	-0.44945	-0.12643
C	-2.00337	-2.15242	-0.81419
C	-1.00325	-2.35508	0.052987
C	0.445571	-2.1457	-0.38698
C	0.98895	-0.83044	0.233251
C	3.586694	-1.35851	0.149179
C	4.909751	-0.80275	-0.39394
C	3.707642	-1.68355	1.653631
C	-3.91869	0.393713	1.532399
C	1.054988	2.547127	1.45186
O	1.627659	3.615377	-0.57757
C	-1.18094	-2.70095	1.51304
O	0.624525	-2.10383	-1.7954
O	0.183861	0.239281	-0.29131
H	-2.32504	1.310881	-1.42779
H	-5.11026	-0.65715	-0.71691
H	-3.79822	-0.37318	-1.85923
H	-2.69368	3.283321	0.546366
H	-1.67034	2.169108	1.413906
H	-4.02432	-2.75228	-1.2139
H	-3.72672	-2.37212	0.476811
H	-0.89713	3.21184	-1.35908
H	-0.68576	4.211973	0.066939
H	0.738519	1.485544	-1.84026
H	3.265784	1.333545	-1.01305
H	-1.7263	-1.93538	-1.84507
H	1.064247	-2.97942	-0.02841
H	0.863663	-0.87987	1.325295
H	3.383942	-2.30642	-0.3733
H	5.721906	-1.51745	-0.22137
H	4.851139	-0.60952	-1.47048
H	5.178098	0.135963	0.105662
H	4.539258	-2.37575	1.826929
H	3.897829	-0.77212	2.232966
H	2.798655	-2.15052	2.048229
H	-3.49668	1.194867	2.144348
H	-5.01373	0.477626	1.583194
H	-3.65976	-0.56071	2.010378
H	2.123733	2.355058	1.583994
H	0.809158	3.488736	1.954066
H	0.496411	1.739474	1.93217
H	1.337791	3.814053	-1.48392
H	-2.18862	-3.06297	1.731633
H	-0.47543	-3.48656	1.81589
H	-0.99255	-1.83996	2.168853
H	0.305915	-1.22477	-2.06575

05_3R4S13S14R_13

C	-3.4592	0.112256	-0.3643
C	-2.72315	1.066728	-0.96011
C	-3.73102	-1.21187	-1.05374
C	-2.41517	2.454508	-0.43244
C	-3.07188	-2.46006	-0.39838
C	-1.31699	2.597629	0.654675
C	0.160359	2.55267	0.211553
C	0.55051	1.156924	-0.34147
C	1.999744	0.930365	-0.69551
C	2.462727	-0.18878	-0.12558
C	-1.57534	-2.4329	-0.54113

C	-0.63684	-2.40466	0.413878
C	0.827049	-2.18078	0.041358
C	1.328988	-0.82719	0.660415
C	3.830645	-0.81828	-0.2278
C	4.726186	-0.13257	-1.26799
C	4.528281	-0.86185	1.149025
C	-4.08355	0.273885	1.001823
C	1.056866	2.959019	1.386991
O	0.293104	3.498417	-0.86554
C	-0.90116	-2.51456	1.896275
O	1.080272	-2.25392	-1.35918
O	0.289008	0.157779	0.651338
H	-2.32123	0.836857	-1.94782
H	-4.81793	-1.38392	-1.07696
H	-3.40147	-1.15547	-2.09914
H	-2.15312	3.105686	-1.2732
H	-3.33029	2.87927	0.001253
H	-3.47657	-3.34639	-0.90938
H	-3.38304	-2.53793	0.64839
H	-1.4624	3.574909	1.133064
H	-1.46055	1.837878	1.429472
H	-0.08961	0.988346	-1.22225
H	2.551257	1.574898	-1.37147
H	-1.23093	-2.37052	-1.57275
H	1.443788	-2.98184	0.471554
H	1.589482	-1.01529	1.710176
H	3.676	-1.85747	-0.55218
H	5.687255	-0.65268	-1.34483
H	4.259822	-0.13332	-2.25913
H	4.93259	0.908217	-0.98945
H	5.4959	-1.37	1.068584
H	4.707298	0.15223	1.525888
H	3.931704	-1.39799	1.895024
H	-4.02072	1.299329	1.375642
H	-5.14276	-0.01734	0.984915
H	-3.59294	-0.37358	1.741126
H	2.111615	2.988046	1.08747
H	0.774112	3.951731	1.752051
H	0.965461	2.242945	2.209272
H	1.239425	3.676481	-0.9858
H	-1.91758	-2.85301	2.111599
H	-0.20989	-3.22913	2.364012
H	-0.75548	-1.55057	2.399805
H	0.759386	-1.43143	-1.76327

05_3R4S13S14R_14

C	-3.47373	0.115156	-0.35293
C	-2.71691	1.058797	-0.93946
C	-3.74278	-1.21049	-1.04108
C	-2.40378	2.446719	-0.41592
C	-3.07729	-2.45639	-0.38768
C	-1.30087	2.587998	0.665538
C	0.177634	2.549536	0.205195
C	0.566092	1.163786	-0.36037
C	2.017517	0.93747	-0.69651
C	2.467761	-0.18817	-0.12978
C	-1.58183	-2.4278	-0.54143
C	-0.63634	-2.40735	0.406554
C	0.824663	-2.17875	0.025469
C	1.323812	-0.82507	0.64257
C	3.834463	-0.82277	-0.21942
C	4.746336	-0.12852	-1.23964
C	4.513927	-0.88508	1.165609
C	-4.12654	0.290051	0.998053
C	1.087281	2.950392	1.373089
O	0.390082	3.43287	-0.91173
C	-0.88987	-2.52567	1.89021
O	1.069634	-2.24871	-1.37663
O	0.282197	0.159048	0.617705
H	-2.29708	0.818155	-1.91689
H	-4.82907	-1.38606	-1.0624
H	-3.4153	-1.1535	-2.08707
H	-2.13313	3.0895	-1.26146
H	-3.31673	2.880334	0.012807
H	-3.48506	-3.34415	-0.89392
H	-3.38071	-2.5324	0.661573
H	-1.449	3.562897	1.15369
H	-1.43929	1.829632	1.443115
H	-0.06212	1.008691	-1.25249
H	2.571056	1.602096	-1.34892
H	-1.24399	-2.35703	-1.5747
H	1.446269	-2.97802	0.452354
H	1.572054	-1.00968	1.696056
H	3.680504	-1.8578	-0.55785
H	5.706338	-0.65164	-1.31042
H	4.292534	-0.11506	-2.23646
H	4.952389	0.908121	-0.94617
H	5.48139	-1.39472	1.09202
H	4.690369	0.123966	1.556962

H	3.906367	-1.42866	1.897322
H	-3.68407	-0.38504	1.742892
H	-4.03109	1.308052	1.384821
H	-5.19618	0.043268	0.949971
H	2.132914	2.987644	1.053505
H	0.807363	3.939797	1.755389
H	1.000736	2.233587	2.195775
H	0.199291	4.335311	-0.60795
H	-1.90308	-2.87029	2.110977
H	-0.19162	-3.23839	2.350242
H	-0.74622	-1.56312	2.397401
H	0.771643	-1.41415	-1.77337

05_3R4S13S14R_15

C	-2.87299	0.885096	1.078895
C	-2.44331	-0.34019	1.425741
C	-2.22632	2.122341	1.67815
C	-2.88346	-1.68971	0.906454
C	-1.45654	3.028513	0.669916
C	-1.72355	-2.68534	0.643447
C	-0.76832	-2.44784	-0.56081
C	-0.05985	-1.09325	-0.4148
C	0.921037	-0.96043	0.720713
C	2.013561	-0.30158	0.326715
C	-0.15254	2.463427	0.158905
C	0.138395	2.03614	-1.08186
C	1.543538	1.555207	-1.4484
C	1.833142	0.047349	-1.14472
C	3.251271	0.024969	1.13152
C	3.125596	-0.3935	2.602856
C	4.511072	-0.60276	0.497623
C	-4.0137	1.143175	0.122042
C	-1.51334	-2.52193	-1.9005
O	0.252107	-3.46024	-0.52392
C	-0.82969	2.008524	-2.24101
O	2.586053	2.295283	-0.80943
O	0.726904	-0.75495	-1.57795
H	-1.64529	-0.38913	2.169241
H	-3.00457	2.747755	2.141247
H	-1.54082	1.827465	2.482382
H	-3.53136	-2.16661	1.658251
H	-3.49894	-1.58842	0.006582
H	-1.23546	3.968724	1.195393
H	-2.12129	3.297871	-0.15688
H	-1.10842	-2.76677	1.548181
H	-2.16727	-3.68007	0.493757
H	-0.85165	-0.34472	-0.31483
H	0.732528	-1.34311	1.715805
H	0.648375	2.423396	0.897282
H	1.641066	1.663959	-2.5412
H	2.706678	-0.23724	-1.74526
H	3.382315	1.112899	1.093817
H	4.020684	-0.09519	3.160344
H	2.258386	0.074901	3.08209
H	3.019223	-1.48107	2.700827
H	5.40061	-0.34199	1.083071
H	4.43169	-1.69638	0.46896
H	4.670816	-0.24641	-0.52526
H	-4.44999	0.22555	-0.27909
H	-4.81576	1.696539	0.63082
H	-3.70433	1.763206	-0.72854
H	-0.80096	-2.42699	-2.72357
H	-2.03358	-3.48407	-1.99434
H	-2.26051	-1.72676	-1.99804
H	-0.18767	-4.31386	-0.66999
H	-1.8421	2.307559	-1.96305
H	-0.49087	2.674248	-3.04741
H	-0.87472	0.998874	-2.66509
H	2.368585	3.23593	-0.91039

05_3R4S13S14R_16

C	-3.49368	0.12054	-0.31892
C	-2.76522	1.088964	-0.9003
C	-3.75424	-1.19297	-1.03183
C	-2.4605	2.4637	-0.34105
C	-3.03587	-2.43139	-0.42783
C	-1.32043	2.593889	0.703944
C	0.149357	2.566133	0.215198
C	0.554249	1.182965	-0.35768
C	2.012483	0.984674	-0.67478
C	2.467182	-0.14699	-0.12873
C	-1.5427	-2.31968	-0.56923
C	-0.58656	-2.46866	0.357495
C	0.864665	-2.16937	-0.00347
C	1.319067	-0.80974	0.616257
C	3.841707	-0.76449	-0.21302
C	4.7584	-0.03723	-1.20562
C	4.506146	-0.85202	1.177926
C	-4.11148	0.24759	1.053519
C	1.065175	2.978888	1.368703

O	0.369079	3.543886	-0.8184
C	-0.81717	-2.84997	1.798323
O	1.098469	-2.11319	-1.40862
O	0.267831	0.158035	0.597946
H	-2.36518	0.879648	-1.89309
H	-4.8356	-1.39608	-1.02713
H	-3.45704	-1.10286	-2.08451
H	-2.26472	3.153491	-1.1736
H	-3.36243	2.860005	0.142647
H	-3.39884	-3.3154	-0.97457
H	-3.33885	-2.56987	0.615314
H	-1.45626	3.561339	1.204547
H	-1.44129	1.818205	1.467162
H	-0.0561	1.017511	-1.26408
H	2.567024	1.670895	-1.30366
H	-1.21611	-2.01484	-1.5631
H	1.511446	-2.94576	0.442295
H	1.558275	-1.0075	1.670422
H	3.704932	-1.79344	-0.57758
H	5.725887	-0.54677	-1.27618
H	4.315753	-0.00622	-2.20694
H	4.947945	0.99487	-0.88612
H	5.480371	-1.34911	1.105134
H	4.666386	0.149616	1.594511
H	3.895921	-1.41863	1.889835
H	-4.0324	1.258245	1.462503
H	-5.17488	-0.02756	1.030264
H	-3.62845	-0.4317	1.768902
H	2.106238	3.030492	1.037367
H	0.773906	3.963456	1.748936
H	0.995717	2.252873	2.184114
H	-0.16568	3.287065	-1.58749
H	-1.8485	-3.15579	1.989089
H	-0.16278	-3.68068	2.095714
H	-0.58962	-2.01176	2.470758
H	0.758401	-2.94269	-1.7823

05_3R4S13S14R_17

C	3.308056	-0.73748	0.409764
C	3.26274	0.258957	-0.49084
C	3.402118	-2.18056	-0.05251
C	3.196292	1.749399	-0.26939
C	2.118684	-3.0258	0.167212
C	2.022239	2.469878	-0.98176
C	0.686411	2.543992	-0.21408
C	0.088172	1.135122	0.037697
C	-1.18683	1.075779	0.835175
C	-2.10741	0.32737	0.217667
C	0.952609	-2.50731	-0.63147
C	-0.3238	-2.32192	-0.25589
C	-1.27351	-1.68982	-1.26461
C	-1.51629	-0.16096	-1.09525
C	-3.51508	0.00577	0.665858
C	-3.8214	0.529548	2.075226
C	-4.55538	0.528731	-0.34828
C	3.276985	-0.54095	1.907178
C	-0.29636	3.450013	-0.96248
O	1.004479	3.112594	1.072516
C	-0.88672	-2.69735	1.094356
O	-2.57293	-2.29399	-1.28173
O	-0.26137	0.517566	-1.2149
H	3.295652	-0.02669	-1.54481
H	3.652438	-2.20526	-1.12069
H	4.227844	-2.68116	0.474611
H	3.181265	2.007665	0.792724
H	4.123988	2.182762	-0.67318
H	1.883088	-3.08069	1.234952
H	2.350021	-4.05568	-0.14698
H	2.326078	3.505939	-1.17635
H	1.833203	2.005515	-1.95697
H	0.868892	0.542377	0.530668
H	-1.29387	1.53802	1.810287
H	1.211356	-2.22359	-1.65339
H	-0.81184	-1.78339	-2.25856
H	-2.14765	0.130257	-1.94735
H	-3.60915	-1.08779	0.676177
H	-4.83244	0.235977	2.378831
H	-3.11827	0.132027	2.815938
H	-3.76734	1.624687	2.112562
H	-5.56858	0.277977	-0.01354
H	-4.49182	1.619307	-0.44856
H	-4.41092	0.083043	-1.33711
H	3.164246	0.505568	2.199214
H	2.449598	-1.10163	2.362458
H	4.199401	-0.92255	2.367522
H	-1.24328	3.537256	-0.41545
H	0.129104	4.452095	-1.08096
H	-0.52242	3.042674	-1.952
H	0.170895	3.394511	1.481389

H	-1.75907	-3.35111	0.978386
H	-0.15501	-3.22021	1.714717
H	-1.23013	-1.81353	1.644107
H	-2.45583	-3.22817	-1.51719

05_3R4S13S14R_18

C	3.32554	-0.70731	0.391772
C	3.247842	0.286163	-0.50982
C	3.431719	-2.14997	-0.06901
C	3.162958	1.775793	-0.28851
C	2.161335	-3.00981	0.169641
C	1.976614	2.480831	-0.99357
C	0.64579	2.546912	-0.20487
C	0.065582	1.137784	0.059253
C	-1.21731	1.077105	0.840447
C	-2.11957	0.309484	0.22015
C	0.983326	-2.50987	-0.62283
C	-0.29034	-2.3268	-0.23749
C	-1.25023	-1.7097	-1.24513
C	-1.50776	-0.18274	-1.08209
C	-3.53028	-0.02111	0.652977
C	-3.86213	0.524617	2.048301
C	-4.56327	0.4697	-0.38413
C	3.322075	-0.50946	1.889227
C	-0.35659	3.441183	-0.94229
O	0.879049	3.062264	1.121283
C	-0.84025	-2.6881	1.121679
O	-2.54344	-2.32752	-1.25447
O	-0.25629	0.505957	-1.19157
H	3.265525	-0.00059	-1.56372
H	3.667445	-2.17426	-1.14054
H	4.27077	-2.63872	0.447981
H	3.138476	2.030746	0.774889
H	4.085727	2.22253	-0.68904
H	1.935881	-3.05929	1.239849
H	2.402377	-4.03902	-0.13936
H	2.271247	3.519302	-1.20313
H	1.7832	2.015731	-1.9678
H	0.847554	0.559876	0.56893
H	-1.33455	1.575692	1.794661
H	1.230507	-2.236	-1.6503
H	-0.79122	-1.8049	-2.24027
H	-2.13122	0.100431	-1.94272
H	-3.61151	-1.11545	0.682904
H	-4.87366	0.224125	2.343851
H	-3.16383	0.149584	2.80507
H	-3.82091	1.620686	2.066173
H	-5.57806	0.213996	-0.0577
H	-4.51077	1.558787	-0.50506
H	-4.40104	0.006286	-1.36207
H	3.221708	0.537628	2.183489
H	2.499756	-1.06475	2.360003
H	4.250473	-0.89661	2.332527
H	-1.2886	3.522878	-0.37465
H	0.055904	4.449183	-1.07655
H	-0.58746	3.033876	-1.93114
H	1.233625	3.961107	1.022521
H	-0.10044	-3.1978	1.743543
H	-1.18572	-1.79889	1.661208
H	-1.70904	-3.34911	1.020566
H	-2.41706	-3.26089	-1.48827

05_3R4S13S14R_19

C	-3.53171	0.096397	0.331044
C	-2.60484	0.87518	0.913821
C	-3.61414	-1.37873	0.684322
C	-2.38628	2.357881	0.73448
C	-3.07471	-2.35955	-0.39878
C	-1.3294	2.833985	-0.29742
C	0.194822	2.642314	-0.05468
C	0.719307	1.280141	-0.57749
C	2.209715	1.065773	-0.55275
C	2.501405	-0.14017	-0.05712
C	-1.63086	-2.09289	-0.73502
C	-0.5403	-2.59788	-0.13797
C	0.836853	-2.06536	-0.517
C	1.199439	-0.82028	0.338321
C	3.846251	-0.81053	0.088813
C	4.983303	-0.00347	-0.55225
C	4.166478	-1.12326	1.56647
C	-4.53828	0.576594	-0.68837
C	0.607465	2.908083	1.388747
O	0.876759	3.646708	-0.84346
C	-0.54959	-3.61589	0.975092
O	0.940413	-1.67902	-1.88545
O	0.205009	0.195008	0.195709
H	-1.92283	0.391149	1.608472
H	-4.66218	-1.64778	0.886996
H	-3.05453	-1.56019	1.609343
H	-3.32765	2.835412	0.43633

H	-2.13452	2.792317	1.711084
H	-3.68496	-2.27162	-1.30697
H	-3.2205	-3.38112	-0.028
H	-1.57765	2.40888	-1.28107
H	-1.45781	3.920637	-0.3868
H	0.35623	1.190212	-1.61908
H	2.905534	1.790091	-0.95726
H	-1.46197	-1.33094	-1.49212
H	1.589396	-2.84143	-0.28956
H	1.200989	-1.1443	1.389285
H	3.785841	-1.77488	-0.44017
H	5.930991	-0.54722	-0.47028
H	4.789989	0.185397	-1.61362
H	5.109491	0.965023	-0.05319
H	5.125405	-1.64806	1.646015
H	4.235717	-0.19817	2.151139
H	3.401143	-1.75675	2.027895
H	-4.4604	1.647322	-0.89436
H	-4.41454	0.050249	-1.64508
H	-5.56486	0.369694	-0.35411
H	1.698775	2.928801	1.469518
H	0.218733	3.873998	1.728634
H	0.228643	2.122726	2.047684
H	0.557799	3.561704	-1.75804
H	-1.55683	-3.9633	1.217893
H	0.058892	-4.4929	0.714454
H	-0.11568	-3.20112	1.896193
H	0.456281	-2.34551	-2.39919

05_3R4S13S14R_20

C	-3.54333	0.085785	0.290269
C	-2.63422	0.891632	0.864473
C	-3.60675	-1.38215	0.676527
C	-2.43492	2.373245	0.657045
C	-3.04882	-2.38043	-0.38079
C	-1.34461	2.839673	-0.34148
C	0.162107	2.634003	-0.03331
C	0.69593	1.279854	-0.57669
C	2.190385	1.073877	-0.57714
C	2.503257	-0.12142	-0.06791
C	-1.60466	-2.10787	-0.70988
C	-0.51481	-2.60296	-0.10307
C	0.861455	-2.06947	-0.4833
C	1.215225	-0.80969	0.355361
C	3.856356	-0.77856	0.05704
C	4.975551	0.031522	-0.61085
C	4.204581	-1.07559	1.531761
C	-4.54896	0.52651	-0.7477
C	0.507285	2.847291	1.441412
O	0.795488	3.657671	-0.83785
C	-0.5236	-3.61029	1.0197
O	0.969287	-1.70193	-1.8562
O	0.208692	0.191123	0.211847
H	-1.95059	0.431179	1.573862
H	-3.04968	-1.53473	1.608321
H	-4.65217	-1.66081	0.87992
H	-3.37026	2.826476	0.307167
H	-2.23455	2.83503	1.633376
H	-3.65156	-2.31708	-1.29607
H	-3.18856	-3.3953	0.00997
H	-1.55569	2.412939	-1.33047
H	-1.46199	3.925611	-0.44442
H	0.318212	1.193807	-1.60936
H	2.886072	1.769935	-1.03242
H	-1.43611	-1.35127	-1.47252
H	1.616629	-2.83868	-0.24146
H	1.233454	-1.12198	1.409813
H	3.793227	-1.74751	-0.46258
H	5.928569	-0.50414	-0.53965
H	4.763741	0.208424	-1.67073
H	5.103395	1.0054	-0.12248
H	5.169698	-1.59045	1.598894
H	4.274935	-0.14507	2.107699
H	3.453343	-1.71234	2.011273
H	-4.48447	1.592555	-0.98033
H	-4.41071	-0.02177	-1.68989
H	-5.57498	0.313454	-0.41547
H	1.594148	2.815299	1.588382
H	0.144339	3.823422	1.780754
H	0.067622	2.068259	2.069683
H	1.734088	3.67819	-0.59072
H	-1.52992	-3.96089	1.261709
H	0.090757	-4.48649	0.770396
H	-0.09591	-3.18406	1.938548
H	0.482441	-2.37216	-2.36243

05_3R4S13S14R_21

C	-3.53581	0.09885	0.310628
C	-2.61306	0.884164	0.891181
C	-3.61808	-1.37299	0.677365

C	-2.39767	2.366068	0.702891
C	-3.07201	-2.36304	-0.39366
C	-1.32432	2.837745	-0.31402
C	0.195616	2.632462	-0.04985
C	0.713618	1.275637	-0.58896
C	2.204445	1.061339	-0.57594
C	2.500917	-0.13779	-0.06683
C	-1.62678	-2.09775	-0.72484
C	-0.53903	-2.59678	-0.11768
C	0.83979	-2.06974	-0.49779
C	1.202622	-0.81512	0.344137
C	3.847389	-0.80562	0.075906
C	4.979347	-0.00348	-0.58015
C	4.177719	-1.10369	1.554397
C	-4.53777	0.567647	-0.71849
C	0.585252	2.864236	1.410902
O	0.900583	3.56586	-0.89993
C	-0.5533	-3.60334	1.005716
O	0.948027	-1.7004	-1.8702
O	0.205986	0.196769	0.196807
H	-1.93305	0.406727	1.592425
H	-4.66685	-1.64127	0.877403
H	-3.06288	-1.54498	1.606867
H	-3.33582	2.836793	0.384398
H	-2.16646	2.80847	1.681338
H	-3.67748	-2.2842	-1.30586
H	-3.21855	-3.38137	-0.01429
H	-1.55515	2.427883	-1.30563
H	-1.46065	3.926185	-0.4001
H	0.340363	1.191897	-1.62361
H	2.895662	1.779073	-0.99902
H	-1.4548	-1.34275	-1.48827
H	1.590716	-2.84395	-0.25842
H	1.209751	-1.12951	1.39815
H	3.784582	-1.77497	-0.44349
H	5.928255	-0.54529	-0.49894
H	4.778955	0.175088	-1.64192
H	5.107458	0.969946	-0.09118
H	5.13793	-1.62632	1.633132
H	4.249248	-0.17285	2.129702
H	3.416204	-1.73365	2.026873
H	-4.4596	1.636085	-0.93577
H	-4.40837	0.031409	-1.66887
H	-5.56587	0.363204	-0.38733

H	1.674142	2.834166	1.51669
H	0.233148	3.845102	1.755449
H	0.154885	2.099225	2.063075
H	0.622989	4.458419	-0.63765
H	-1.56148	-3.94908	1.247127
H	0.057178	-4.48252	0.757333
H	-0.1242	-3.17861	1.924568
H	0.459614	-2.36898	-2.37708
05_3R4S13S14R_22			
C	-3.45706	0.35023	0.074138
C	-2.70992	1.330353	-0.45573
C	-3.99148	-0.76565	-0.80399
C	-2.10776	2.529292	0.242146
C	-3.35463	-2.1578	-0.53293
C	-0.8845	3.161813	-0.46539
C	0.554453	2.692713	-0.13073
C	0.887167	1.269468	-0.66095
C	2.321725	0.842859	-0.49889
C	2.398731	-0.3362	0.122889
C	-1.86268	-2.10464	-0.70612
C	-0.89897	-2.44279	0.162033
C	0.55662	-2.1362	-0.17309
C	0.994738	-0.79299	0.466014
C	3.625415	-1.14935	0.478037
C	4.169343	-1.91125	-0.74888
C	4.725675	-0.28543	1.118611
C	-3.81945	0.260651	1.537029
C	0.874997	2.832177	1.354875
O	1.454996	3.600847	-0.80497
C	-1.11639	-3.06339	1.520131
O	0.816524	-2.0485	-1.57111
O	0.140835	0.270884	0.040789
H	-2.53078	1.29449	-1.5321
H	-5.08003	-0.86055	-0.67246
H	-3.82307	-0.50879	-1.85752
H	-2.87591	3.316839	0.293677
H	-1.85973	2.297972	1.28296
H	-3.79801	-2.86892	-1.24637
H	-3.63437	-2.50981	0.465282
H	-1.02356	3.075481	-1.55482
H	-0.88051	4.236871	-0.24822
H	0.596837	1.251959	-1.72844
H	3.146595	1.440812	-0.86806
H	-1.53768	-1.65306	-1.64272

H	1.193251	-2.91845	0.277601
H	0.886593	-0.91742	1.5563
H	3.320376	-1.89969	1.222177
H	4.988357	-2.57926	-0.45579
H	3.385036	-2.50373	-1.22893
H	4.556026	-1.20582	-1.49418
H	5.582617	-0.90508	1.408189
H	5.087014	0.475723	0.417116
H	4.358392	0.22988	2.012898
H	-3.42644	1.094495	2.123673
H	-4.9112	0.247388	1.666198
H	-3.4431	-0.66819	1.986276
H	1.942792	2.667565	1.526793
H	0.618776	3.837118	1.706946
H	0.315966	2.098914	1.941344
H	1.203301	3.615122	-1.74388
H	-2.17039	-3.25934	1.729619
H	-0.57668	-4.01689	1.604062
H	-0.73346	-2.41893	2.323582
H	0.387965	-2.81579	-1.98422

05_3R4S13S14R_23

C	-2.80908	0.878337	1.117419
C	-2.42041	-0.35253	1.493694
C	-2.14281	2.10919	1.710482
C	-2.88438	-1.69954	0.989103
C	-1.37497	3.016836	0.701103
C	-1.74202	-2.68004	0.612421
C	-0.87349	-2.3562	-0.62505
C	-0.12088	-1.0286	-0.43948
C	0.851962	-0.94708	0.709556
C	1.985921	-0.35104	0.330471
C	-0.08163	2.442576	0.174321
C	0.207643	2.048626	-1.07813
C	1.598786	1.524466	-1.4422
C	1.843674	0.008036	-1.14223
C	3.228239	-0.08846	1.151085
C	3.046475	-0.45424	2.63048
C	4.451708	-0.82162	0.559823
C	-3.92126	1.15252	0.131495
C	-1.71341	-2.32011	-1.91225
O	0.076167	-3.43111	-0.71549
C	-0.74702	2.092477	-2.24808
O	2.661029	2.225921	-0.79238
O	0.720211	-0.76685	-1.59297
H	-1.63469	-0.40932	2.249578
H	-2.91162	2.739859	2.182352
H	-1.4532	1.806509	2.508292
H	-3.45985	-2.19585	1.785471
H	-3.57437	-1.59301	0.146317
H	-1.13981	3.949405	1.234237
H	-2.04536	3.301098	-0.11586
H	-1.07583	-2.80826	1.473814
H	-2.18677	-3.66645	0.429081
H	-0.86474	-0.23275	-0.36698
H	0.629781	-1.31041	1.70442
H	0.711971	2.353638	0.916223
H	1.706387	1.634434	-2.53363
H	2.715189	-0.29908	-1.73292
H	3.434751	0.986251	1.085068
H	3.949841	-0.20241	3.197322
H	2.205136	0.086544	3.078406
H	2.863545	-1.52861	2.756513
H	5.348137	-0.60078	1.150847
H	4.299149	-1.90783	0.568328
H	4.650943	-0.51436	-0.47215
H	-4.41866	0.243539	-0.21396
H	-4.68552	1.794745	0.591288
H	-3.56068	1.689524	-0.7548
H	-1.06657	-2.16326	-2.781
H	-2.23804	-3.27325	-2.03877
H	-2.45528	-1.5138	-1.89643
H	0.779917	-3.09689	-1.29933
H	-1.74195	2.452466	-1.97879
H	-0.35775	2.745434	-3.04181
H	-0.85239	1.093752	-2.68829
H	2.470713	3.173728	-0.88038

05_3R4S13S14R_24

C	-3.4593	0.114589	-0.33778
C	-2.73749	1.087901	-0.92006
C	-3.73073	-1.19425	-1.0555
C	-2.43641	2.464362	-0.36045
C	-3.07217	-2.4556	-0.42594
C	-1.29476	2.605802	0.6825
C	0.17454	2.557847	0.194146
C	0.558975	1.157413	-0.34865
C	2.008437	0.931379	-0.69264
C	2.464131	-0.19037	-0.12256
C	-1.5753	-2.42484	-0.56394

C	-0.64034	-2.40859	0.394657	05_3R4S13S14R_25			
C	0.824993	-2.18204	0.029845	C	-3.47875	0.32658	-0.02288
C	1.322964	-0.82939	0.652518	C	-2.70926	1.299669	-0.53398
C	3.833947	-0.81841	-0.21024	C	-3.95087	-0.82231	-0.89291
C	4.7398	-0.12718	-1.23788	C	-2.15679	2.515965	0.176547
C	4.516239	-0.86531	1.174036	C	-3.3092	-2.19445	-0.5429
C	-4.06933	0.239155	1.038521	C	-0.91525	3.162607	-0.479
C	1.099767	2.983355	1.334809	C	0.503358	2.676383	-0.10394
O	0.402861	3.508402	-0.86161	C	0.823129	1.241341	-0.61749
C	-0.90847	-2.53456	1.874987	C	2.272574	0.831758	-0.54417
O	1.084442	-2.2541	-1.36984	C	2.409353	-0.32666	0.106387
O	0.282101	0.157027	0.635958	C	-1.81292	-2.13293	-0.66915
H	-2.34557	0.884484	-1.91766	C	-0.87088	-2.43876	0.234228
H	-4.81778	-1.36464	-1.08166	C	0.59162	-2.14687	-0.08175
H	-3.40153	-1.11579	-2.09955	C	1.036926	-0.79658	0.544315
H	-2.24819	3.154628	-1.19456	C	3.671326	-1.11631	0.384767
H	-3.33869	2.856915	0.125556	C	4.252343	-1.72549	-0.90713
H	-3.47537	-3.33051	-0.95719	C	4.724865	-0.26456	1.116601
H	-3.38629	-2.55642	0.617946	C	-3.92138	0.273594	1.420311
H	-1.42446	3.582775	1.165736	C	0.788039	2.81973	1.393135
H	-1.42099	1.844919	1.459626	O	1.349542	3.578895	-0.85302
H	-0.0765	0.982246	-1.23658	C	-1.12053	-3.01204	1.607086
H	2.5598	1.598138	-1.34476	O	0.869621	-2.08055	-1.47765
H	-1.22601	-2.34865	-1.593	O	0.148344	0.257871	0.175968
H	1.439615	-2.98283	0.463775	H	-2.46667	1.240952	-1.59671
H	1.573852	-1.01735	1.70446	H	-5.04325	-0.93002	-0.81446
H	3.684848	-1.85691	-0.5399	H	-3.73147	-0.59641	-1.94422
H	5.702652	-0.64529	-1.30611	H	-2.93833	3.291899	0.185721
H	4.284239	-0.12475	-2.23404	H	-1.95527	2.296416	1.229959
H	4.940416	0.912977	-0.95339	H	-3.71931	-2.93908	-1.24202
H	5.486283	-1.37026	1.102599	H	-3.62025	-2.50984	0.458433
H	4.6878	0.147906	1.556479	H	-1.00677	3.102065	-1.57218
H	3.913064	-1.40616	1.91135	H	-0.91798	4.232557	-0.23865
H	-4.01271	1.255709	1.436703	H	0.463314	1.188387	-1.65875
H	-5.12546	-0.06273	1.026452	H	3.069795	1.392329	-1.02155
H	-3.56354	-0.42016	1.756518	H	-1.46485	-1.70954	-1.61067
H	2.139191	3.02154	0.99601	H	1.21667	-2.9287	0.385654
H	0.818404	3.976951	1.698221	H	0.990458	-0.92708	1.637763
H	1.029934	2.272912	2.163631	H	3.402673	-1.94928	1.049937
H	-0.17124	3.269523	-1.60771	H	5.112033	-2.367	-0.67864
H	-1.92473	-2.87713	2.084613	H	3.497935	-2.31763	-1.43275
H	-0.21682	-3.25219	2.337302	H	4.591148	-0.93409	-1.58658
H	-0.76593	-1.57546	2.388919	H	5.610871	-0.86577	1.352567
H	0.809448	-1.41254	-1.76811	H	5.048336	0.577995	0.493876

H	4.329315	0.142681	2.053788
H	-3.5967	1.143319	1.996404
H	-5.01723	0.218832	1.488263
H	-3.53423	-0.622	1.925122
H	1.835923	2.578423	1.610184
H	0.595841	3.848385	1.71669
H	0.16766	2.140839	1.983501
H	2.251145	3.480691	-0.50759
H	-2.17907	-3.20567	1.795173
H	-0.57926	-3.95906	1.74009
H	-0.76225	-2.3364	2.396133
H	0.443467	-2.85211	-1.88545

05_3R4S13S14R_26

C	-3.48994	-0.25895	-0.25581
C	-2.883	0.741073	-0.91793
C	-3.61009	-1.64181	-0.86871
C	-2.72001	2.176097	-0.45939
C	-2.71072	-2.73246	-0.22383
C	-1.61994	2.478998	0.592165
C	-0.16496	2.635876	0.10284
C	0.414892	1.301109	-0.43674
C	1.882493	1.293285	-0.78195
C	2.50775	0.259214	-0.21072
C	-1.25421	-2.4357	-0.45299
C	-0.24096	-2.41383	0.423117
C	1.138518	-1.946	-0.03124
C	1.470646	-0.54255	0.566935
C	3.992331	-0.02881	-0.25947
C	4.611898	0.011808	1.15317
C	4.365025	-1.34253	-0.97367
C	-4.0998	-0.1036	1.117559
C	0.690481	3.194292	1.247825
O	-0.20054	3.572822	-0.98982
C	-0.34656	-2.75906	1.887599
O	1.26505	-1.86195	-1.44721
O	0.312309	0.296109	0.579876
H	-2.47601	0.508679	-1.90275
H	-4.65475	-1.97955	-0.79758
H	-3.36992	-1.5894	-1.93837
H	-2.54738	2.814879	-1.33191
H	-3.66721	2.51709	-0.02077
H	-2.97719	-3.69324	-0.6911
H	-2.94481	-2.833	0.841297
H	-1.88453	3.430467	1.071611
H	-1.63072	1.712132	1.372945
H	-0.19261	1.017064	-1.30989
H	2.350621	2.025253	-1.43375
H	-1.02397	-2.13143	-1.47331
H	1.89796	-2.64238	0.365728
H	1.765781	-0.70449	1.612599
H	4.441641	0.790678	-0.83707
H	5.700765	-0.10023	1.094507
H	4.393167	0.95933	1.657947
H	4.229432	-0.80204	1.781202
H	5.455054	-1.41386	-1.06976
H	4.024702	-2.21864	-0.41027
H	3.91979	-1.394	-1.97037
H	-4.1173	0.934939	1.458243
H	-5.13103	-0.48277	1.132088
H	-3.54425	-0.68376	1.867004
H	1.72246	3.371056	0.921641
H	0.274175	4.142846	1.602579
H	0.724007	2.49003	2.08471
H	0.711897	3.85183	-1.16693
H	-1.30332	-3.22344	2.137992
H	0.449689	-3.45372	2.187802
H	-0.23833	-1.86242	2.512594
H	1.02454	-2.73259	-1.80396

Table S4.. DP4+ results obtained using experimental data of compound 5 *versus* isomers 5a-5d

Functional		Solvent?		Basis Set		Type of Data	
mPW1PW91		PCM		6-311G(d,p)		Shielding Tensors	
		DP4+	0.00%	100.00%	0.00%	0.00%	-
Nuclei	sp2?	xperimenta	Isomer 1	Isomer 2	Isomer 3	Isomer 4	Isomer 5
C	x	150.34	29.9963132	30.4660645	28.6578485	29.888422	
C	x	121.2	60.6559923	59.1232248	59.1380858	58.9206303	
C		87.63	91.896291	94.7805839	92.8179325	94.1620483	
C		74.93	110.00521	111.260747	112.054249	108.156839	
C		39.37	150.034075	145.453329	146.312277	141.25874	
C		22.57	161.791613	159.974185	161.805824	160.864477	
C	x	128.74	50.06382	50.5656532	49.041335	52.1531393	
C	x	133.16	44.8073429	46.2602058	47.1018255	45.9825299	
C		39.64	145.484394	144.144856	145.663905	143.93389	
C		25	157.915356	158.833991	157.495743	158.791626	
C	x	130.08	51.9886508	50.8585059	52.5583317	55.8119476	
C	x	132.19	42.2618611	43.8253549	42.8530437	44.512168	
C		79.67	107.275425	108.125782	107.934471	109.534397	
C		86.05	95.2122679	94.2835959	95.7216244	97.847462	
C		27.43	155.490321	155.44348	154.913007	156.187818	
C		21.11	165.000747	166.650397	164.249778	165.397248	
C		22.57	166.582839	165.148593	165.664722	166.554474	
C		23.36	169.615827	163.917173	169.906948	171.189815	
C		15.84	163.668655	171.631476	161.094738	162.43471	
C		12.89	171.109143	169.319718	169.983784	171.877057	

Table S5. Cartesian coordinates of all conformers of isomers **7a-7b** used after optimization at the B3LYP/ 6-311G (d,p) level of theory as required for DP4+ analysis

07_1S4R10R_3

C	2.765412	1.037503	-0.77217
C	1.507437	1.501682	-0.88326
C	3.076782	-0.38054	-1.22395
C	0.983309	2.884803	-0.58628
C	3.527864	-1.40503	-0.15688
C	0.012741	3.005954	0.617051
C	-1.48141	2.68383	0.361689
C	-1.68072	1.279269	-0.17258
C	-2.17299	0.218996	0.475612
C	-2.25067	-1.18215	-0.08472
C	2.534318	-1.61577	0.995769
C	1.212169	-2.26926	0.631744
C	0.060331	-1.65354	0.949242
C	-1.35557	-2.13513	0.766287
C	-3.72504	-1.68185	-0.15942
C	-4.58219	-0.76148	-1.04324
C	-3.84158	-3.13151	-0.65977
C	3.943055	1.852839	-0.29034
C	-2.28066	2.959809	1.64419
O	-1.89505	3.622194	-0.65919
C	1.306743	-3.63519	-0.00928
O	4.769247	-1.04576	0.467924
H	0.765715	0.798539	-1.25689
H	3.872994	-0.34405	-1.98679
H	2.197944	-0.80267	-1.72629
H	1.817669	3.56947	-0.4043
H	0.463307	3.27124	-1.47323
H	3.666281	-2.36271	-0.68351
H	0.366495	2.381693	1.445941
H	0.039424	4.04642	0.966779
H	-1.35713	1.154446	-1.20566
H	-2.51033	0.323322	1.508736
H	-1.84736	-1.16828	-1.10849
H	2.352598	-0.6504	1.480805
H	3.054477	-2.24956	1.729928
H	0.143567	-0.67405	1.417856
H	-1.37306	-3.13173	0.319782
H	-1.82481	-2.23274	1.759462
H	-4.13344	-1.6446	0.86309
H	-5.62703	-1.09343	-1.04973
H	-4.5566	0.275913	-0.69626
H	-4.22321	-0.77401	-2.08115
H	-4.89566	-3.41045	-0.77444
H	-3.36102	-3.24867	-1.64022
H	-3.38685	-3.85218	0.026604
H	4.416004	1.373592	0.573112
H	4.714883	1.910777	-1.07206
H	3.672056	2.875014	-0.01558
H	-3.34856	2.769155	1.486308
H	-1.9446	2.333739	2.477614
H	-2.15712	4.008364	1.931897
H	-2.80595	3.389775	-0.90323
H	1.971189	-4.28895	0.572228
H	0.338183	-4.13338	-0.09215
H	1.733862	-3.5802	-1.01957
H	5.416576	-0.89038	-0.23859

07_1S4R10R_4

C	-2.71996	1.199767	0.655733
C	-1.44245	1.59157	0.814515
C	-3.14768	-0.16526	1.172227
C	-0.81563	2.922664	0.480453
C	-3.65188	-1.216	0.155969
C	0.215223	2.92877	-0.67842
C	1.670762	2.513567	-0.34369
C	1.739842	1.120847	0.249821
C	2.152461	-0.00368	-0.34442
C	2.074913	-1.37812	0.277727
C	-2.65345	-1.57433	-0.95497
C	-1.38696	-2.28841	-0.51487
C	-0.19065	-1.7773	-0.85119
C	1.190335	-2.31995	-0.59339
C	3.472386	-2.00892	0.5663
C	4.385889	-2.10029	-0.66719
C	4.186354	-1.26864	1.707023
C	-3.81383	2.059986	0.066468
C	2.541591	2.678815	-1.59813
O	2.108082	3.459887	0.659293
C	-1.58411	-3.59164	0.225206
O	-4.84781	-0.80476	-0.52291
H	-0.76864	0.86678	1.266843
H	-3.95585	-0.02569	1.910438
H	-2.31499	-0.62159	1.721386
H	-1.59502	3.648305	0.22707
H	-0.31456	3.317921	1.374346
H	-3.87569	-2.12538	0.735999
H	-0.14418	2.297838	-1.49977
H	0.277107	3.954291	-1.06591

H	1.373916	1.066678	1.27504
H	2.520232	0.032757	-1.37029
H	1.569669	-1.27733	1.248708
H	-2.39624	-0.65882	-1.49933
H	-3.20303	-2.21968	-1.65693
H	-0.19907	-0.83222	-1.3928
H	1.156973	-3.30322	-0.11145
H	1.688217	-2.46809	-1.56356
H	3.27708	-3.03598	0.910177
H	5.31065	-2.63243	-0.41498
H	3.914201	-2.63823	-1.49681
H	4.670307	-1.1049	-1.02932
H	5.144355	-1.74643	1.944341
H	4.389238	-0.22624	1.433729
H	3.579234	-1.2618	2.620455
H	-4.27509	1.563893	-0.79355
H	-4.61837	2.21401	0.80078
H	-3.45975	3.044312	-0.24889
H	3.582931	2.409306	-1.38659
H	2.190622	2.051067	-2.42412
H	2.515277	3.722661	-1.92584
H	2.991241	3.177131	0.948051
H	-2.26623	-4.25149	-0.3285
H	-0.64914	-4.13428	0.383205
H	-2.04098	-3.43164	1.210962
H	-5.49555	-0.55328	0.154961

07_1S4R10R_5

C	2.765634	1.036943	-0.77211
C	1.507797	1.501363	-0.88365
C	3.076957	-0.38116	-1.22376
C	0.983919	2.884618	-0.58678
C	3.527523	-1.40572	-0.15654
C	0.013555	3.006037	0.616693
C	-1.48063	2.683951	0.361464
C	-1.6801	1.279302	-0.17253
C	-2.17278	0.219312	0.475819
C	-2.25084	-1.18192	-0.08426
C	2.533692	-1.61628	0.99586
C	1.211399	-2.26927	0.631524
C	0.059744	-1.65389	0.950343
C	-1.35627	-2.13506	0.76715
C	-3.72534	-1.6812	-0.15923
C	-4.5822	-0.7605	-1.04298
C	-3.84209	-3.13075	-0.65987
C	3.943256	1.852055	-0.28983
C	-2.27979	2.960228	1.643966
O	-1.89422	3.622154	-0.65961
C	1.305574	-3.63429	-0.0115
O	4.768795	-1.04668	0.468593
H	0.766035	0.798405	-1.25755
H	3.873448	-0.3448	-1.98631
H	2.19823	-0.80315	-1.72643
H	1.818429	3.569188	-0.40506
H	0.463823	3.271025	-1.47367
H	3.665864	-2.36346	-0.68312
H	0.367337	2.381826	1.445606
H	0.040301	4.046539	0.966301
H	-1.35635	1.154171	-1.20554
H	-2.51025	0.323983	1.508867
H	-1.84727	-1.16837	-1.10793
H	2.352212	-0.65092	1.481012
H	3.053526	-2.25037	1.730007
H	0.143264	-0.67505	1.420258
H	-1.37395	-3.13174	0.320832
H	-1.82577	-2.2323	1.760233
H	-4.13386	-1.64405	0.863236
H	-5.62708	-1.09229	-1.04967
H	-4.5565	0.276818	-0.69579
H	-4.22309	-0.77287	-2.08086
H	-4.89621	-3.40953	-0.77459
H	-3.36155	-3.24773	-1.64036
H	-3.38744	-3.85163	0.026333
H	4.41543	1.373013	0.574174
H	4.715639	1.909372	-1.07103
H	3.672425	2.874434	-0.01567
H	-3.34771	2.769667	1.486176
H	-1.94373	2.334251	2.477469
H	-2.15611	4.008816	1.931502
H	-2.80526	3.389905	-0.90333
H	1.971162	-4.28857	0.568113
H	0.337082	-4.13274	-0.09359
H	1.731148	-3.57772	-1.02235
H	5.416109	-0.89061	-0.23778

07_1S4R10R_6

C	2.772053	1.052328	-0.74877
C	1.515718	1.526893	-0.82926
C	3.063507	-0.36158	-1.22595
C	1.009396	2.906973	-0.49086

C	3.522546	-1.40329	-0.1787
C	-0.02044	2.99568	0.664619
C	-1.5109	2.688331	0.336672
C	-1.69904	1.276474	-0.18686
C	-2.17515	0.215824	0.470532
C	-2.24492	-1.19001	-0.07944
C	2.542529	-1.62154	0.984285
C	1.216081	-2.27167	0.630042
C	0.067233	-1.65378	0.954526
C	-1.35	-2.13468	0.780042
C	-3.71835	-1.69272	-0.15575
C	-4.57159	-0.78285	-1.0542
C	-3.83106	-3.14811	-0.64021
C	3.965483	1.849609	-0.27611
C	-2.36114	2.982484	1.575797
O	-1.98756	3.623643	-0.65818
C	1.303958	-3.63799	-0.01139
O	4.774759	-1.06079	0.433374
H	0.761494	0.836272	-1.2007
H	3.847522	-0.3209	-2.0009
H	2.172939	-0.76976	-1.71898
H	1.847714	3.560423	-0.23136
H	0.565615	3.360763	-1.39143
H	3.646859	-2.35464	-0.71986
H	0.302028	2.344858	1.486892
H	-0.00515	4.025002	1.045744
H	-1.37319	1.131862	-1.21912
H	-2.5129	0.326449	1.502814
H	-1.8391	-1.18266	-1.10248
H	2.36737	-0.65981	1.478916
H	3.070656	-2.26148	1.707174
H	0.154351	-0.67396	1.421959
H	-1.36973	-3.13608	0.344696
H	-1.81703	-2.22062	1.775252
H	-4.13169	-1.64437	0.86428
H	-5.61671	-1.11397	-1.05937
H	-4.54531	0.259245	-0.7222
H	-4.20966	-0.81063	-2.09088
H	-4.88437	-3.42846	-0.75848
H	-3.34441	-3.27631	-1.61633
H	-3.38072	-3.86114	0.05702
H	4.452426	1.348935	0.567005
H	4.720222	1.916346	-1.07349
H	3.71013	2.868564	0.023963
H	-3.41568	2.770637	1.375962
H	-2.03702	2.383759	2.432313
H	-2.26476	4.039849	1.841554
H	-1.59039	3.380048	-1.50958
H	1.97382	-4.29165	0.56392
H	0.334568	-4.13597	-0.08473
H	1.72118	-3.58361	-1.02584
H	5.415812	-0.908	-0.27941

07_1S4R10R_7

C	2.772786	1.051678	-0.74878
C	1.516368	1.526237	-0.82797
C	3.063712	-0.36243	-1.22577
C	1.010529	2.90649	-0.48967
C	3.522308	-1.40408	-0.17827
C	-0.02013	2.995681	0.664974
C	-1.51041	2.688585	0.336339
C	-1.69866	1.276647	-0.18695
C	-2.17529	0.216241	0.470459
C	-2.24529	-1.18966	-0.07937
C	2.541961	-1.62191	0.984503
C	1.215372	-2.27161	0.629967
C	0.066654	-1.65372	0.954861
C	-1.35066	-2.1344	0.780219
C	-3.71886	-1.69207	-0.15572
C	-4.5719	-0.78216	-1.05431
C	-3.8319	-3.14748	-0.64002
C	3.966744	1.848964	-0.27751
C	-2.36126	2.983324	1.574937
O	-1.98646	3.623671	-0.65901
C	1.302921	-3.63756	-0.01234
O	4.774445	-1.06177	0.434018
H	0.761715	0.835451	-1.19824
H	3.84775	-0.32217	-2.00072
H	2.17301	-0.77034	-1.71876
H	1.849003	3.559411	-0.22931
H	0.567901	3.360814	-1.39059
H	3.646475	-2.35558	-0.71922
H	0.301677	2.34498	1.487615
H	-0.00478	4.025092	1.045862
H	-1.37252	1.131797	-1.21909
H	-2.51339	0.327128	1.502602
H	-1.83945	-1.18243	-1.10239
H	2.367022	-0.66008	1.47902
H	3.069707	-2.26198	1.70756

H	0.153915	-0.67416	1.422824
H	-1.37044	-3.13582	0.344912
H	-1.81775	-2.22024	1.775407
H	-4.13223	-1.64353	0.864294
H	-5.61703	-1.11324	-1.05967
H	-4.54563	0.259936	-0.72231
H	-4.20978	-0.80995	-2.09093
H	-4.88527	-3.42758	-0.75834
H	-3.34521	-3.27592	-1.61608
H	-3.38181	-3.86055	0.05733
H	4.454145	1.348552	0.565509
H	4.720875	1.915235	-1.07548
H	3.711822	2.868093	0.022381
H	-3.41578	2.771931	1.374512
H	-2.03791	2.384564	2.431718
H	-2.26453	4.040684	1.840581
H	-1.58686	3.381287	-1.50963
H	1.974607	-4.29097	0.561087
H	0.333745	-4.13625	-0.08372
H	1.717747	-3.58229	-1.02772
H	5.415366	-0.90787	-0.27864

07_1S4R10R_8

C	-2.72827	1.212991	0.631385
C	-1.44916	1.613445	0.749541
C	-3.13268	-0.14902	1.173952
C	-0.83966	2.939935	0.368798
C	-3.6478	-1.21414	0.177811
C	0.25371	2.905332	-0.73061
C	1.701699	2.516988	-0.31076
C	1.763542	1.11779	0.273543
C	2.154442	-0.00578	-0.33494
C	2.069949	-1.38634	0.272701
C	-2.66385	-1.57734	-0.94453
C	-1.3924	-2.28897	-0.51525
C	-0.19909	-1.77362	-0.85601
C	1.183619	-2.31659	-0.60844
C	3.465577	-2.02469	0.5546
C	4.379491	-2.10211	-0.67954
C	4.180572	-1.30104	1.7053
C	-3.84437	2.057976	0.062464
C	2.632498	2.700848	-1.51292
O	2.189591	3.460048	0.670064
C	-1.58231	-3.59539	0.221266
O	-4.85695	-0.81761	-0.48566

H	-0.75835	0.900307	1.194204
H	-3.92638	-0.00781	1.927118
H	-2.2845	-0.59266	1.709504
H	-1.61968	3.62789	0.029135
H	-0.4174	3.41421	1.269518
H	-3.85596	-2.11834	0.77131
H	-0.06881	2.238369	-1.53973
H	0.326646	3.9146	-1.1562
H	1.402088	1.043111	1.301502
H	2.516193	0.038399	-1.36259
H	1.565014	-1.29439	1.244907
H	-2.4138	-0.6646	-1.49683
H	-3.22175	-2.22675	-1.63591
H	-0.21244	-0.82635	-1.39388
H	1.152627	-3.30639	-0.13999
H	1.679039	-2.45106	-1.58181
H	3.26695	-3.05573	0.884559
H	5.301524	-2.64226	-0.43453
H	3.905929	-2.62568	-1.51732
H	4.668782	-1.10275	-1.02623
H	5.137551	-1.78411	1.936143
H	4.385116	-0.25537	1.446812
H	3.573343	-1.30667	2.618793
H	-4.32818	1.545743	-0.77528
H	-4.62647	2.217222	0.819374
H	-3.50807	3.040361	-0.27705
H	3.656619	2.42002	-1.24969
H	2.308253	2.09432	-2.36382
H	2.62839	3.751267	-1.82043
H	1.717119	3.29535	1.501692
H	-2.26758	-4.25397	-0.32999
H	-0.64561	-4.13741	0.370613
H	-2.03227	-3.43989	1.210952
H	-5.49821	-0.57009	0.199771

07_1S4R10R_9

C	2.79319	0.885204	-0.89193
C	1.567256	1.436535	-0.90066
C	2.947837	-0.52895	-1.42226
C	1.117933	2.786451	-0.40794
C	3.27583	-1.63291	-0.38292
C	0.130547	2.739766	0.786634
C	-1.36086	2.446646	0.47871
C	-1.5607	1.15267	-0.29066
C	-1.9601	-0.03349	0.18032

C	-2.0662	-1.29479	-0.64634	H	-0.53205	-1.93035	2.557032
C	2.527897	-1.54876	0.951332	H	1.096961	-2.30674	3.139913
C	1.039001	-1.82937	1.032775	H	5.185057	-1.62418	-0.8266
C	0.266415	-2.17029	-0.01108	07_1S4R10R_10			
C	-1.2183	-2.44125	-0.01671	C	2.762748	1.050997	-0.76742
C	-3.54042	-1.74778	-0.89026	C	1.503112	1.509318	-0.88644
C	-4.33826	-1.99518	0.400502	C	3.084204	-0.36209	-1.23142
C	-4.28356	-0.75893	-1.80009	C	0.970897	2.890082	-0.59109
C	4.054989	1.561326	-0.41169	C	3.535321	-1.39186	-0.17838
C	-2.15923	2.489369	1.790843	C	0.002475	3.009755	0.614166
O	-1.78543	3.542724	-0.36447	C	-1.49068	2.680451	0.362609
C	0.523553	-1.67536	2.447372	C	-1.68389	1.274641	-0.17024
O	4.664423	-1.60067	-0.00781	C	-2.17405	0.21356	0.478145
H	0.771047	0.813354	-1.2986	C	-2.24601	-1.1883	-0.0811
H	3.745306	-0.56374	-2.18303	C	2.542948	-1.60855	0.98231
H	2.027525	-0.81337	-1.94235	C	1.218866	-2.26491	0.628388
H	1.981069	3.386935	-0.10164	C	0.067332	-1.65174	0.951692
H	0.634463	3.33955	-1.22493	C	-1.34787	-2.13657	0.77175
H	3.059432	-2.60339	-0.85856	C	-3.71821	-1.6941	-0.15655
H	0.480768	2.006635	1.522759	C	-4.57843	-0.77738	-1.04119
H	0.14695	3.721388	1.278226	C	-3.82848	-3.14428	-0.65694
H	-1.32286	1.239597	-1.35112	C	3.931916	1.87278	-0.27691
H	-2.20175	-0.13758	1.23822	C	-2.28877	2.953483	1.646464
H	-1.64099	-1.0822	-1.63839	O	-1.9111	3.615993	-0.65807
H	2.707973	-0.55117	1.376607	C	1.313684	-3.62888	-0.01689
H	3.04116	-2.24385	1.63228	O	4.809534	-0.97363	0.330268
H	0.731976	-2.24835	-0.99254	H	0.767099	0.804145	-1.26775
H	-1.4086	-3.35288	-0.60162	H	3.88761	-0.31629	-1.98182
H	-1.5847	-2.64619	0.994152	H	2.206789	-0.78292	-1.73597
H	-3.47599	-2.70603	-1.4277	H	1.801716	3.579943	-0.4127
H	-5.33606	-2.3817	0.161773	H	0.446871	3.271643	-1.47774
H	-3.85186	-2.72565	1.056252	H	3.668038	-2.34582	-0.71296
H	-4.47432	-1.06837	0.970792	H	0.360649	2.38873	1.443654
H	-5.29795	-1.11444	-2.01697	H	0.024977	4.051049	0.961845
H	-4.36627	0.226584	-1.32696	H	-1.35769	1.149647	-1.20243
H	-3.7623	-0.62704	-2.75618	H	-2.51391	0.317761	1.510469
H	4.568769	0.94364	0.333166	H	-1.84183	-1.17398	-1.10447
H	4.76133	1.685077	-1.24574	H	2.365924	-0.64556	1.473589
H	3.873509	2.549552	0.018895	H	3.056791	-2.25123	1.717878
H	-3.22512	2.317392	1.601289	H	0.150194	-0.67276	1.421449
H	-1.81584	1.730769	2.502225	H	-1.36361	-3.13463	0.328557
H	-2.04537	3.47267	2.257861	H	-1.81549	-2.23184	1.765902
H	-2.69764	3.352406	-0.63847	H	-4.12752	-1.65831	0.865663
H	0.653549	-0.64019	2.792511	H	-5.62195	-1.1134	-1.04805

H	-4.55698	0.260245	-0.69465
H	-4.21885	-0.78896	-2.0789
H	-4.88131	-3.42746	-0.77262
H	-3.34653	-3.25945	-1.63693
H	-3.37157	-3.86328	0.029787
H	4.393794	1.404902	0.598275
H	4.716952	1.916928	-1.04482
H	3.654176	2.89796	-0.01996
H	-3.35612	2.75801	1.49074
H	-1.94834	2.329331	2.479592
H	-2.16945	4.002739	1.933397
H	-2.82106	3.378414	-0.90065
H	1.984938	-4.28249	0.557231
H	0.346476	-4.13062	-0.09309
H	1.732105	-3.56933	-1.03046
H	5.096905	-1.6404	0.974654

07_1S4R10R_11

C	2.762881	1.050717	-0.76739
C	1.503333	1.509188	-0.88675
C	3.084374	-0.36236	-1.23144
C	0.97123	2.889992	-0.59136
C	3.535158	-1.39219	-0.17831
C	0.002872	3.00972	0.613936
C	-1.49032	2.680503	0.362432
C	-1.68365	1.274659	-0.17022
C	-2.17401	0.213742	0.478297
C	-2.24611	-1.18817	-0.0808
C	2.542634	-1.60873	0.982275
C	1.21848	-2.26484	0.628248
C	0.06705	-1.65187	0.952285
C	-1.34822	-2.13649	0.772288
C	-3.71834	-1.69383	-0.15641
C	-4.57851	-0.77699	-1.04094
C	-3.82858	-3.14394	-0.65705
C	3.931945	1.87229	-0.27631
C	-2.28834	2.953799	1.646275
O	-1.91067	3.615939	-0.6584
C	1.313047	-3.62838	-0.018
O	4.809367	-0.9742	0.330459
H	0.767343	0.804164	-1.2684
H	3.888001	-0.31653	-1.98159
H	2.207054	-0.78306	-1.73625
H	1.802118	3.57977	-0.41294
H	0.44724	3.271648	-1.47798
H	3.667737	-2.3462	-0.71286
H	0.361038	2.388645	1.443389
H	0.025447	4.051002	0.961633
H	-1.35737	1.149444	-1.20236
H	-2.51388	0.318148	1.510591
H	-1.84178	-1.174	-1.10411
H	2.365744	-0.64573	1.47359
H	3.056329	-2.25154	1.717836
H	0.150082	-0.67321	1.422687
H	-1.36407	-3.13462	0.329269
H	-1.81594	-2.23149	1.766409
H	-4.12769	-1.65821	0.865789
H	-5.622	-1.11311	-1.04802
H	-4.55721	0.260564	-0.69415
H	-4.21881	-0.78826	-2.07861
H	-4.88141	-3.42717	-0.77263
H	-3.34676	-3.25886	-1.63713
H	-3.37151	-3.86303	0.029478
H	4.392573	1.4049	0.599793
H	4.717871	1.915382	-1.04335
H	3.654441	2.897822	-0.02049
H	-3.35576	2.758618	1.49059
H	-1.94804	2.32957	2.479395
H	-2.16873	4.00303	1.933169
H	-2.82059	3.378256	-0.90105
H	1.9849	-4.28223	0.555156
H	0.345871	-4.13025	-0.09371
H	1.730638	-3.56811	-1.03187
H	5.095937	-1.64029	0.975901

07_1S4R10R_12

C	-2.70773	1.217939	0.641438
C	-1.43405	1.607706	0.834273
C	-3.15765	-0.13311	1.17876
C	-0.79477	2.933786	0.501659
C	-3.66436	-1.19437	0.184129
C	0.229361	2.9378	-0.66304
C	1.682442	2.507157	-0.33893
C	1.740755	1.111566	0.248323
C	2.149509	-0.01264	-0.349
C	2.065931	-1.38817	0.269505
C	-2.66998	-1.56734	-0.93415
C	-1.40009	-2.28217	-0.50331
C	-0.20536	-1.77667	-0.85339
C	1.175386	-2.32322	-0.60254

C	3.460929	-2.0266	0.553554	C	-1.44941	1.613226	0.749897
C	4.371465	-2.11925	-0.68207	C	-3.13312	-0.14919	1.173759
C	4.180794	-1.2926	1.694622	C	-0.83983	2.93976	0.369441
C	-3.77627	2.074825	0.003515	C	-3.64788	-1.21437	0.177521
C	2.548089	2.669713	-1.59725	C	0.253376	2.90544	-0.73011
O	2.134893	3.4444	0.665973	C	1.70148	2.517052	-0.31055
C	-1.59504	-3.57879	0.248931	C	1.763411	1.117775	0.273532
O	-4.8919	-0.71686	-0.38382	C	2.154391	-0.00572	-0.33503
H	-0.77719	0.888046	1.319243	C	2.070065	-1.38635	0.272396
H	-3.97557	0.027261	1.897492	C	-2.6636	-1.57767	-0.94448
H	-2.33277	-0.58767	1.739843	C	-1.39205	-2.2889	-0.51484
H	-1.56892	3.667572	0.255742	C	-0.19885	-1.7739	-0.85652
H	-0.2859	3.320648	1.394605	C	1.183894	-2.31668	-0.60886
H	-3.88223	-2.09604	0.778036	C	3.465839	-2.02455	0.554073
H	-0.14064	2.314957	-1.48588	C	4.379999	-2.10081	-0.67996
H	0.29866	3.965018	-1.04491	C	4.18045	-1.30155	1.705414
H	1.37206	1.055244	1.272359	C	-3.84443	2.057732	0.061972
H	2.519749	0.02539	-1.37391	C	2.632034	2.701172	-1.51284
H	1.562943	-1.28754	1.241634	O	2.1895	3.459985	0.670328
H	-2.41895	-0.65856	-1.49238	C	-1.58179	-3.59477	0.222716
H	-3.21582	-2.22766	-1.63013	O	-4.85685	-0.81792	-0.48643
H	-0.21533	-0.83522	-1.40137	H	-0.75877	0.900059	1.194753
H	1.141081	-3.30867	-0.12519	H	-3.92712	-0.00791	1.926588
H	1.668847	-2.46813	-1.57541	H	-2.28516	-0.59282	1.709685
H	3.261414	-3.05357	0.895294	H	-1.61988	3.627803	0.030028
H	5.293706	-2.65736	-0.43338	H	-0.41751	3.413804	1.270263
H	3.895158	-2.65191	-1.51248	H	-3.8563	-2.1185	0.771039
H	4.660818	-1.12436	-1.04162	H	-0.06926	2.238661	-1.53933
H	5.136983	-1.77555	1.928794	H	0.326231	3.914812	-1.15547
H	4.388106	-0.25053	1.423473	H	1.40187	1.043024	1.301469
H	3.57558	-1.2851	2.609288	H	2.516239	0.038612	-1.36264
H	-4.20696	1.572081	-0.86801	H	1.565151	-1.29467	1.244639
H	-4.60967	2.232102	0.702542	H	-2.41366	-0.66501	-1.49698
H	-3.40584	3.055038	-0.30643	H	-3.22126	-2.22735	-1.63583
H	3.588146	2.390228	-1.39242	H	-0.21239	-0.82714	-1.39531
H	2.18711	2.048543	-2.42394	H	1.152963	-3.30647	-0.14039
H	2.529362	3.715128	-1.92047	H	1.679444	-2.45113	-1.58217
H	3.015756	3.150097	0.950166	H	3.26744	-3.05589	0.883245
H	-2.28029	-4.2436	-0.29526	H	5.301852	-2.64144	-0.43535
H	-0.65999	-4.12113	0.406985	H	3.906507	-2.62331	-1.51845
H	-2.04665	-3.40932	1.235423	H	4.669581	-1.10112	-1.02546
H	-5.21287	-1.39918	-0.9952	H	5.137609	-1.78441	1.935969
07_1S4R10R_13				H	4.384619	-0.2556	1.447747
C	-2.72849	1.21278	0.631271	H	3.573133	-1.30814	2.618839

H	-4.32844	1.545122	-0.77543
H	-4.62639	2.217579	0.818918
H	-3.50792	3.039832	-0.27814
H	3.656237	2.420379	-1.24988
H	2.307666	2.094782	-2.3638
H	2.627821	3.751653	-1.82014
H	1.717713	3.294712	1.502232
H	-2.26689	-4.25389	-0.32814
H	-0.64503	-4.13652	0.372618
H	-2.03193	-3.43857	1.212202
H	-5.49832	-0.57034	0.198796

07_1S4R10R_14

C	2.783634	0.901105	-0.88562
C	1.555557	1.447507	-0.90794
C	2.956139	-0.5057	-1.43273
C	1.096431	2.794992	-0.41638
C	3.286031	-1.61752	-0.41087
C	0.114576	2.745743	0.782678
C	-1.37609	2.443571	0.481357
C	-1.56897	1.149046	-0.28824
C	-1.96211	-0.03915	0.182697
C	-2.06043	-1.301	-0.64382
C	2.54627	-1.54522	0.934649
C	1.056264	-1.82472	1.027618
C	0.279761	-2.1608	-0.01487
C	-1.20322	-2.44056	-0.01478
C	-3.53166	-1.76495	-0.88523
C	-4.32541	-2.01811	0.406924
C	-4.2833	-0.7812	-1.79358
C	4.034161	1.583753	-0.38531
C	-2.16951	2.480021	1.796666
O	-1.81156	3.537368	-0.35919
C	0.548776	-1.68053	2.445973
O	4.701712	-1.53418	-0.17022
H	0.766884	0.824151	-1.32068
H	3.769773	-0.52985	-2.17175
H	2.042773	-0.79067	-1.96339
H	1.955917	3.40332	-0.11552
H	0.605635	3.341932	-1.23308
H	3.065004	-2.58298	-0.89401
H	0.471767	2.015863	1.518701
H	0.127377	3.728438	1.272352
H	-1.33123	1.237311	-1.34855
H	-2.20347	-0.14465	1.24054
H	-1.63825	-1.08572	-1.6365
H	2.736781	-0.55477	1.371381
H	3.048822	-2.25449	1.614636
H	0.740636	-2.22925	-0.99917
H	-1.38891	-3.35455	-0.59754
H	-1.56486	-2.64608	0.997668
H	-3.46129	-2.72264	-1.42292
H	-5.32073	-2.412	0.169939
H	-3.83254	-2.74492	1.061963
H	-4.46729	-1.09224	0.977281
H	-5.29581	-1.14338	-2.00815
H	-4.37139	0.203822	-1.3204
H	-3.76502	-0.64611	-2.75081
H	4.538263	0.971597	0.370537
H	4.755559	1.704116	-1.20616
H	3.840706	2.57289	0.038089
H	-3.23503	2.301348	1.611323
H	-1.81833	1.723274	2.506245
H	-2.06013	3.463781	2.263799
H	-2.72214	3.339207	-0.63297
H	0.682667	-0.64815	2.797826
H	-0.50697	-1.93375	2.558876
H	1.123346	-2.31847	3.131786
H	4.928855	-2.21954	0.478833

07_1S4R10R_15

C	2.84521	0.881044	-0.89174
C	1.602828	1.343408	-0.66615
C	3.059408	-0.59789	-1.18542
C	1.151405	2.763413	-0.42995
C	3.484429	-1.4954	0.002655
C	0.141877	2.949108	0.729055
C	-1.35798	2.715222	0.408438
C	-1.61585	1.330418	-0.15442
C	-2.18416	0.288024	0.460514
C	-2.31108	-1.10031	-0.12398
C	2.412828	-1.67828	1.096708
C	1.093035	-2.30066	0.673441
C	-0.05698	-1.6748	0.978414
C	-1.47759	-2.10866	0.728496
C	-3.80325	-1.53415	-0.23856
C	-4.60138	-0.56202	-1.12211
C	-3.97	-2.96854	-0.76754
C	4.085501	1.744225	-0.96646
C	-2.19249	3.022158	1.660873

O	-1.67344	3.687381	-0.61523
C	1.18888	-3.64251	-0.01413
O	4.709548	-1.05595	0.607444
H	0.805393	0.60418	-0.6779
H	3.836566	-0.69883	-1.95516
H	2.1412	-1.02614	-1.60533
H	2.015753	3.406822	-0.23407
H	0.686633	3.160659	-1.34397
H	3.73434	-2.47788	-0.41395
H	0.418923	2.307102	1.574102
H	0.213725	3.986175	1.08127
H	-1.26064	1.202192	-1.17717
H	-2.55097	0.395718	1.483271
H	-1.8845	-1.08757	-1.13821
H	2.219963	-0.70105	1.558352
H	2.878424	-2.30985	1.868505
H	0.032215	-0.71817	1.491397
H	-1.50744	-3.08956	0.248536
H	-1.98628	-2.22343	1.699976
H	-4.23122	-1.49797	0.776011
H	-5.65854	-0.85007	-1.15836
H	-4.54149	0.466458	-0.75331
H	-4.21956	-0.5691	-2.15183
H	-5.03237	-3.20174	-0.90508
H	-3.47735	-3.08815	-1.74166
H	-3.55723	-3.71892	-0.08651
H	4.924393	1.271691	-0.444
H	4.406187	1.861179	-2.01197
H	3.938858	2.746253	-0.55438
H	-3.26208	2.895494	1.456852
H	-1.92796	2.368255	2.498731
H	-2.02098	4.05849	1.967791
H	-2.58269	3.507435	-0.90538
H	1.802297	-4.33595	0.577601
H	0.214275	-4.11017	-0.17175
H	1.675822	-3.55894	-0.99508
H	4.49961	-0.2689	1.136097

07_1S4R10R_16

C	2.7986	0.703269	-1.02882
C	1.608962	1.328547	-1.04179
C	2.851958	-0.75132	-1.46178
C	1.259501	2.732436	-0.62411
C	3.134697	-1.7941	-0.35553
C	0.343473	2.819738	0.623543
C	-1.17636	2.594571	0.419081
C	-1.49924	1.263354	-0.23708
C	-1.97278	0.155286	0.341117
C	-2.23573	-1.15526	-0.36452
C	2.461637	-1.54733	1.005488
C	0.964262	-1.73098	1.185322
C	0.116752	-2.05853	0.196854
C	-1.37325	-2.28284	0.281876
C	-3.75598	-1.50946	-0.34935
C	-4.59877	-0.39986	-0.99916
C	-4.06486	-2.85138	-1.03562
C	4.112602	1.333897	-0.63467
C	-1.89173	2.785161	1.765994
O	-1.58809	3.649527	-0.48146
C	0.539461	-1.50715	2.620158
O	4.561739	-1.80569	-0.17429
H	0.762444	0.731376	-1.37042
H	3.637759	-0.89745	-2.21655
H	1.904235	-1.01065	-1.9432
H	2.168423	3.304188	-0.40896
H	0.757139	3.254387	-1.44983
H	2.817666	-2.77659	-0.74087
H	0.695795	2.116344	1.387315
H	0.441698	3.828445	1.045965
H	-1.3066	1.251605	-1.31009
H	-2.18077	0.150674	1.412555
H	-1.93017	-1.05115	-1.41714
H	2.729925	-0.53502	1.339341
H	2.953588	-2.22079	1.727934
H	0.521697	-2.18698	-0.80573
H	-1.59768	-3.22525	-0.2315
H	-1.70031	-2.41494	1.319886
H	-4.06164	-1.59078	0.705884
H	-5.66617	-0.64403	-0.94488
H	-4.44552	0.567651	-0.51243
H	-4.3383	-0.28392	-2.05983
H	-5.1481	-3.01506	-1.07888
H	-3.68879	-2.85857	-2.06735
H	-3.628	-3.707	-0.51204
H	4.003775	2.363356	-0.2834
H	4.608835	0.748083	0.146841
H	4.802471	1.341691	-1.49067
H	-2.97629	2.677951	1.648599
H	-1.55909	2.055956	2.512511

H	-1.68391	3.78833	2.150807
H	-2.52357	3.493311	-0.69095
H	0.780782	-0.48273	2.936395
H	-0.52831	-1.66129	2.78531
H	1.085868	-2.17716	3.298477
H	4.762675	-2.45307	0.520818

07_1S4R10R_17

C	2.802914	0.714797	-1.01343
C	1.614102	1.341273	-1.00132
C	2.846749	-0.73496	-1.46319
C	1.275165	2.739262	-0.55571
C	3.135629	-1.7889	-0.36922
C	0.315599	2.808918	0.660129
C	-1.20567	2.601284	0.404261
C	-1.51951	1.263027	-0.24597
C	-1.97369	0.151173	0.337992
C	-2.23172	-1.16353	-0.36203
C	2.471108	-1.55346	0.997804
C	0.973939	-1.73323	1.183316
C	0.122365	-2.06036	0.198189
C	-1.36705	-2.28608	0.288793
C	-3.75174	-1.51908	-0.3464
C	-4.59323	-0.41764	-1.01178
C	-4.05829	-2.86958	-1.01675
C	4.123674	1.337482	-0.63042
C	-1.95882	2.80711	1.722259
O	-1.69468	3.655195	-0.4572
C	0.555473	-1.51051	2.620141
O	4.563641	-1.80367	-0.19737
H	0.761228	0.749673	-1.32311
H	3.62517	-0.87543	-2.22644
H	1.893559	-0.98602	-1.93802
H	2.184906	3.288802	-0.29303
H	0.830975	3.30178	-1.39274
H	2.81494	-2.76701	-0.76232
H	0.646276	2.085009	1.415365
H	0.408679	3.806846	1.107789
H	-1.33472	1.23575	-1.32218
H	-2.17633	0.149317	1.410399
H	-1.92625	-1.06339	-1.41525
H	2.745227	-0.54561	1.340161
H	2.964431	-2.23595	1.710803
H	0.523408	-2.19007	-0.80593
H	-1.59112	-3.23203	-0.21825
H	-1.69108	-2.41254	1.328414
H	-4.0596	-1.58778	0.709074
H	-5.66089	-0.65977	-0.95292
H	-4.43779	0.557336	-0.54131
H	-4.33386	-0.31906	-2.07455
H	-5.14147	-3.033	-1.06248
H	-3.67813	-2.8901	-2.04687
H	-3.6243	-3.71894	-0.48063
H	4.023072	2.360453	-0.2584
H	4.632114	0.735818	0.130746
H	4.798008	1.360324	-1.49835
H	-3.0354	2.686906	1.569444
H	-1.62994	2.093668	2.483881
H	-1.77178	3.818345	2.096996
H	-1.33339	3.503467	-1.34533
H	0.804757	-0.48873	2.938621
H	-0.5127	-1.65807	2.78844
H	1.099778	-2.18614	3.294615
H	4.768416	-2.45816	0.489927

07_1S4R10R_18

C	2.847748	0.59199	-0.90363
C	1.711118	1.305557	-0.98187
C	2.822056	-0.85337	-1.36701
C	1.439398	2.727627	-0.56768
C	3.021605	-1.94285	-0.28116
C	0.436411	2.876455	0.604741
C	-1.07512	2.746953	0.283599
C	-1.42827	1.430147	-0.38729
C	-1.93361	0.3298	0.178824
C	-2.21408	-0.9618	-0.55652
C	2.315455	-1.70175	1.057068
C	0.804546	-1.78603	1.166709
C	-0.01889	-2.08099	0.147886
C	-1.52657	-2.16052	0.162953
C	-3.74617	-1.17166	-0.78415
C	-4.03558	-2.29964	-1.78747
C	-4.54833	-1.37872	0.510887
C	4.17609	1.114747	-0.41097
C	-1.88013	2.990776	1.570126
O	-1.3492	3.817547	-0.64906
C	0.3328	-1.49427	2.574725
O	4.40921	-2.07732	0.073891
H	0.847285	0.780935	-1.38139
H	3.603352	-1.01982	-2.12765

H	1.869765	-1.04421	-1.87222
H	2.369696	3.224747	-0.27262
H	1.046706	3.294405	-1.42291
H	2.671001	-2.89669	-0.70776
H	0.683864	2.153697	1.391282
H	0.566363	3.87799	1.035715
H	-1.21057	1.417324	-1.456
H	-2.15235	0.327265	1.24664
H	-1.76692	-0.87604	-1.55792
H	2.62937	-0.71714	1.431913
H	2.747846	-2.42477	1.764723
H	0.420575	-2.26977	-0.83082
H	-1.82976	-3.08824	-0.34011
H	-1.90796	-2.22907	1.186551
H	-4.10051	-0.23629	-1.24093
H	-5.10178	-2.32111	-2.04281
H	-3.47177	-2.16201	-2.71821
H	-3.77939	-3.28558	-1.38209
H	-5.62107	-1.42662	0.289222
H	-4.27689	-2.31664	1.009961
H	-4.39573	-0.56082	1.223973
H	4.578108	0.477244	0.384225
H	4.918227	1.093973	-1.22256
H	4.119189	2.14128	-0.03971
H	-2.95636	2.942858	1.367481
H	-1.64892	2.249875	2.342799
H	-1.64609	3.983593	1.966632
H	-2.27815	3.727761	-0.91803
H	0.57976	-0.46131	2.857371
H	-0.7421	-1.62693	2.710345
H	0.845437	-2.14364	3.297565
H	4.906856	-2.20277	-0.74986

07_1S4R10R_19

C	-3.40535	0.886187	0.283163
C	-2.11226	1.233804	0.413838
C	-3.89143	-0.55598	0.280525
C	-1.53368	2.625022	0.508211
C	-2.85548	-1.68516	0.386647
C	-0.49522	2.988899	-0.58812
C	0.998901	2.828635	-0.21409
C	1.342232	1.413294	0.208658
C	2.171917	0.561864	-0.40385
C	2.459102	-0.85532	0.035297
C	-2.10268	-1.93507	-0.93805
C	-0.68022	-2.42807	-0.75384
C	0.337472	-1.64105	-1.14234
C	1.816957	-1.85836	-0.97494
C	3.989198	-1.08682	0.208639
C	4.588602	-0.10293	1.226978
C	4.334191	-2.52764	0.620526
C	-4.54847	1.870504	0.165245
C	1.864317	3.34435	-1.37294
O	1.187274	3.695421	0.931303
C	-0.53305	-3.78657	-0.1124
O	-3.50934	-2.92033	0.724455
H	-1.3721	0.438101	0.473433
H	-4.50471	-0.73283	-0.61691
H	-4.59293	-0.66709	1.124221
H	-2.33415	3.36884	0.4779
H	-1.05146	2.754068	1.486744
H	-2.13316	-1.43638	1.177739
H	-0.6897	2.41362	-1.50087
H	-0.627	4.046494	-0.84913
H	0.849216	1.096033	1.128312
H	2.673605	0.866006	-1.32435
H	1.978201	-1.02206	1.010782
H	-2.09091	-1.00729	-1.51872
H	-2.70332	-2.6633	-1.5023
H	0.073698	-0.68938	-1.60196
H	2.025671	-2.87938	-0.64716
H	2.319572	-1.73397	-1.9472
H	4.46172	-0.89233	-0.76732
H	5.672366	-0.2446	1.312228
H	4.403078	0.938504	0.946084
H	4.15309	-0.26074	2.222747
H	5.41	-2.62195	0.809675
H	3.811009	-2.80714	1.544696
H	4.073874	-3.26075	-0.14893
H	-5.13293	1.67169	-0.74357
H	-5.24474	1.762949	1.008921
H	-4.22634	2.913005	0.131624
H	2.929991	3.283717	-1.12391
H	1.69754	2.773574	-2.2929
H	1.61928	4.39256	-1.56939
H	2.087855	3.535957	1.258306
H	-0.97495	-4.5606	-0.75566
H	0.507549	-4.061	0.077376
H	-1.0841	-3.83303	0.833758

H	-3.87856	-2.8195	1.616779
07_1S4R10R_20			
C	-2.81192	0.987719	0.608768
C	-1.60382	1.54534	0.81113
C	-3.07938	-0.41189	1.143599
C	-1.14558	2.942163	0.469765
C	-3.45548	-1.52313	0.14518
C	-0.0393	3.066905	-0.60978
C	1.435176	2.821586	-0.17477
C	1.640958	1.413549	0.352541
C	2.149874	0.363098	-0.29685
C	2.2271	-1.03536	0.271609
C	-2.43704	-1.74939	-0.9909
C	-1.08851	-2.31937	-0.58459
C	0.034845	-1.65033	-0.89389
C	1.471289	-2.03566	-0.65189
C	3.699257	-1.45848	0.576088
C	3.770507	-2.74728	1.411229
C	4.592457	-1.57119	-0.66983
C	-3.981	1.689243	-0.04226
C	2.361679	3.158065	-1.34707
O	1.800276	3.767789	0.855109
C	-1.1237	-3.66771	0.098258
O	-4.74479	-1.20715	-0.39764
H	-0.86016	0.923529	1.305832
H	-3.90581	-0.3609	1.868469
H	-2.19847	-0.75904	1.695801
H	-1.9958	3.542134	0.131461
H	-0.79575	3.440281	1.388139
H	-3.54114	-2.45089	0.732779
H	-0.27483	2.398327	-1.44707
H	-0.07046	4.09309	-0.99876
H	1.287412	1.259593	1.37448
H	2.505096	0.484832	-1.32069
H	1.702108	-1.02781	1.237446
H	-2.30008	-0.80575	-1.53045
H	-2.90731	-2.45398	-1.69817
H	-0.09136	-0.69042	-1.39364
H	1.54613	-3.04136	-0.22878
H	1.987275	-2.06833	-1.62321
H	4.10838	-0.6485	1.197179
H	4.800094	-2.93583	1.737781
H	3.14262	-2.67882	2.307989
H	3.445658	-3.62473	0.839538

H	5.62844	-1.77632	-0.37517
H	4.273623	-2.38931	-1.32681
H	4.595681	-0.64741	-1.25881
H	-4.31819	1.14127	-0.9276
H	-4.84101	1.712589	0.641005
H	-3.75297	2.7174	-0.33345
H	3.405915	2.984434	-1.07069
H	2.125504	2.553851	-2.22822
H	2.243862	4.212843	-1.61461
H	1.337064	3.514249	1.669449
H	-1.73712	-4.3762	-0.47553
H	-0.13133	-4.10821	0.218256
H	-1.57637	-3.60475	1.096811
H	-4.9851	-1.9223	-1.00854
07_1S4R10R_21			
C	2.550051	1.715695	0.20896
C	1.228109	1.860495	0.396843
C	3.283207	0.540945	0.830328
C	0.332936	2.98391	-0.08188
C	3.961262	-0.436	-0.15328
C	-0.99845	3.062961	0.698286
C	-2.21892	2.345252	0.073195
C	-1.92881	0.936527	-0.42701
C	-2.16976	-0.22481	0.192564
C	-1.90368	-1.59442	-0.39422
C	2.986136	-1.19072	-1.07726
C	1.89676	-2.0134	-0.40779
C	0.634322	-1.55596	-0.39845
C	-0.5979	-2.21338	0.180487
C	-3.11743	-2.55871	-0.24687
C	-3.47556	-2.90363	1.208403
C	-4.34859	-2.033	-0.99981
C	3.392983	2.692213	-0.57783
C	-3.39056	2.388659	1.066534
O	-2.55536	3.150913	-1.0812
C	2.313592	-3.33377	0.193642
O	4.724086	-1.41276	0.56786
H	0.721787	1.088591	0.973812
H	4.079367	0.93311	1.486112
H	2.601465	-0.03424	1.46601
H	0.856553	3.942906	0.020907
H	0.113027	2.890745	-1.15452
H	4.64145	0.132879	-0.80788
H	-0.85033	2.675766	1.71389

H	-1.29881	4.112362	0.804502
H	-1.50162	0.915958	-1.43104
H	-2.57441	-0.21432	1.205084
H	-1.74225	-1.46757	-1.47462
H	3.597481	-1.84231	-1.71783
H	2.524846	-0.44287	-1.73223
H	0.456849	-0.5849	-0.85613
H	-0.59541	-3.29148	-0.03143
H	-0.60537	-2.12499	1.277077
H	-2.80999	-3.49574	-0.73508
H	-4.27002	-3.65883	1.23263
H	-2.61908	-3.30783	1.7588
H	-3.84534	-2.02824	1.755663
H	-5.16599	-2.76322	-0.96926
H	-4.71799	-1.09933	-0.55866
H	-4.11534	-1.83315	-2.05284
H	4.290897	2.97979	-0.01361
H	3.746345	2.252688	-1.5209
H	2.846071	3.604391	-0.83161
H	-4.29588	1.967355	0.614768
H	-3.17293	1.822079	1.978123
H	-3.59541	3.426316	1.349313
H	-3.27532	2.69361	-1.5466
H	3.039019	-3.17763	0.999424
H	1.465915	-3.89922	0.591133
H	2.818617	-3.95863	-0.55651
H	5.323809	-0.93249	1.161153
07_1S4R10R_22			
C	-2.19724	0.874753	1.149149
C	-1.89154	1.509225	0.003642
C	-2.69345	-0.56438	1.233049
C	-1.36271	2.909587	-0.18727
C	-3.43769	-1.22086	0.051171
C	-0.13061	3.011999	-1.11429
C	1.24944	2.660182	-0.5039
C	1.320993	1.231191	0.002193
C	2.027818	0.218189	-0.51088
C	2.040886	-1.1957	0.026366
C	-2.5964	-1.6373	-1.18798
C	-1.27988	-2.32552	-0.8849
C	-0.12909	-1.71414	-1.21499
C	1.287353	-2.14922	-0.95492
C	3.497595	-1.67127	0.306776
C	4.211954	-0.72864	1.289389

C	3.566178	-3.11332	0.837278
C	-2.03587	1.497626	2.518595
C	2.345518	2.992029	-1.52793
O	1.388522	3.546348	0.632677
C	-1.38353	-3.6814	-0.22597
O	-4.56277	-0.43677	-0.35904
H	-2.02277	0.971056	-0.93258
H	-3.3794	-0.61928	2.089473
H	-1.83898	-1.20192	1.504759
H	-2.15675	3.512987	-0.65343
H	-1.13035	3.396247	0.762794
H	-3.88326	-2.13589	0.459152
H	-0.28005	2.389619	-2.00582
H	-0.05844	4.050238	-1.4631
H	0.715544	1.051605	0.888983
H	2.63161	0.375339	-1.40688
H	1.49269	-1.20882	0.980143
H	-2.41215	-0.75568	-1.80966
H	-3.24225	-2.29878	-1.78398
H	-0.21355	-0.74392	-1.7021
H	1.314736	-3.16605	-0.55647
H	1.845021	-2.17035	-1.90488
H	4.041938	-1.63682	-0.65034
H	5.247578	-1.04986	1.451357
H	4.229226	0.303506	0.926164
H	3.707402	-0.72868	2.264913
H	4.598003	-3.37223	1.102477
H	2.954519	-3.2274	1.742193
H	3.224215	-3.85053	0.104547
H	-3.01478	1.641966	2.996521
H	-1.46631	0.8286	3.17832
H	-1.52557	2.462545	2.500038
H	3.339549	2.779596	-1.11789
H	2.230374	2.414563	-2.45171
H	2.298033	4.055823	-1.7802
H	2.213775	3.299747	1.081592
H	-1.99505	-4.35836	-0.8386
H	-0.41197	-4.15677	-0.07142
H	-1.87921	-3.61934	0.752213
H	-4.23777	0.472338	-0.4731
07_1S4R10R_23			
C	-2.53865	1.252228	0.67726
C	-1.25535	1.645831	0.782661
C	-2.94144	-0.11071	1.223705

C	-0.6574	2.996462	0.478086
C	-3.59691	-1.13499	0.278586
C	0.404414	3.078057	-0.64566
C	1.78379	2.416158	-0.38689
C	1.722241	0.907647	-0.54951
C	1.926235	0.003629	0.411895
C	1.831089	-1.50358	0.289913
C	-2.78137	-1.46293	-0.98826
C	-1.44638	-2.14563	-0.75236
C	-0.31482	-1.52684	-1.12748
C	1.109272	-2.00593	-0.99046
C	3.210212	-2.21146	0.486388
C	4.235263	-1.8626	-0.60285
C	3.796494	-1.95084	1.883419
C	-3.65885	2.119103	0.150202
C	2.804768	2.977181	-1.39926
O	2.173571	2.800955	0.938257
C	-1.50187	-3.51749	-0.12142
O	-4.89471	-0.64091	-0.08004
H	-0.55042	0.925951	1.19137
H	-3.65555	0.03891	2.048132
H	-2.05896	-0.59315	1.659076
H	-1.44473	3.7128	0.219975
H	-0.18839	3.377115	1.395079
H	-3.7235	-2.05941	0.865024
H	-0.00384	2.668363	-1.5782
H	0.591805	4.144353	-0.82963
H	1.488406	0.586462	-1.56339
H	2.146148	0.374289	1.413012
H	1.208526	-1.83533	1.136279
H	-2.63367	-0.53528	-1.5522
H	-3.41063	-2.12042	-1.61199
H	-0.43152	-0.53821	-1.56498
H	1.15064	-3.10184	-0.98137
H	1.671919	-1.69055	-1.8788
H	3.004152	-3.29031	0.418023
H	5.177873	-2.39435	-0.4267
H	3.883649	-2.13845	-1.60293
H	4.451832	-0.78786	-0.60937
H	4.686934	-2.56878	2.049114
H	4.09771	-0.90374	2.007066
H	3.073085	-2.18755	2.673417
H	-4.16642	1.634012	-0.68968
H	-4.42897	2.262985	0.921015
H	-3.31642	3.105468	-0.17251
H	3.791451	2.524099	-1.24128
H	2.502451	2.764659	-2.43109
H	2.89497	4.062025	-1.27932
H	3.06822	2.452172	1.085059
H	-2.2146	-4.16242	-0.65381
H	-0.53071	-4.01914	-0.11963
H	-1.84564	-3.46989	0.920455
H	-5.30117	-1.29716	-0.66881

07_1S4R10S_3

C	-2.46809	1.653322	0.512082
C	-1.17071	1.895238	0.769696
C	-3.10361	0.367706	1.013471
C	-0.35782	3.125875	0.45145
C	-3.82993	-0.486	-0.03655
C	0.704844	2.974157	-0.66803
C	2.060121	2.327519	-0.28448
C	1.881645	0.934287	0.286234
C	2.09425	-0.23572	-0.32529
C	1.77253	-1.58506	0.272941
C	-2.91855	-1.10297	-1.12286
C	-1.77739	-2.00544	-0.68141
C	-0.50949	-1.61829	-0.90619
C	0.774043	-2.35971	-0.63739
C	3.036429	-2.44245	0.591202
C	3.962267	-2.66934	-0.61537
C	3.825064	-1.84675	1.766827
C	-3.39821	2.616463	-0.19002
C	2.997112	2.364107	-1.5014
O	2.602374	3.180981	0.74945
C	-2.17344	-3.33468	-0.08075
O	-4.55364	-1.47609	0.709177
H	-0.63158	1.108438	1.292758
H	-3.86176	0.618166	1.770933
H	-2.35092	-0.25355	1.511229
H	-1.02	3.947541	0.159262
H	0.159624	3.46068	1.360104
H	-4.55522	0.151461	-0.56655
H	0.280305	2.413116	-1.509
H	0.944101	3.979034	-1.04065
H	1.502639	0.928156	1.308075
H	2.471285	-0.24723	-1.34855
H	1.262405	-1.41236	1.231102
H	-2.51457	-0.27221	-1.71437

H	-3.57542	-1.6683	-1.80557
H	-0.36826	-0.63808	-1.36096
H	0.585117	-3.33875	-0.18458
H	1.265202	-2.55691	-1.60292
H	2.661946	-3.42692	0.909908
H	4.775745	-3.35308	-0.34587
H	3.434673	-3.10835	-1.46924
H	4.421023	-1.7307	-0.94918
H	4.682185	-2.48008	2.025139
H	4.206978	-0.84899	1.51972
H	3.196694	-1.75113	2.660655
H	-2.94987	3.600251	-0.34747
H	-3.70485	2.237838	-1.17457
H	-4.32114	2.75547	0.389396
H	3.973324	1.931807	-1.25261
H	2.586973	1.807116	-2.3506
H	3.148881	3.40185	-1.81372
H	3.417066	2.757975	1.066629
H	-2.79207	-3.90501	-0.7893
H	-1.31462	-3.95693	0.181964
H	-2.78671	-3.18881	0.814417
H	-5.0255	-2.03435	0.070416

07_1S4R10S_4

C	2.524185	1.545475	-0.65666
C	1.226056	1.818896	-0.87538
C	3.085388	0.194869	-1.06605
C	0.47701	3.102979	-0.61956
C	3.765584	-0.61811	0.045699
C	-0.51156	3.086251	0.575265
C	-1.91913	2.488485	0.325747
C	-1.85141	1.066113	-0.19347
C	-2.14206	-0.06246	0.460958
C	-1.96256	-1.45673	-0.09245
C	2.816266	-1.11392	1.161364
C	1.630786	-1.9822	0.769304
C	0.382995	-1.52841	0.984274
C	-0.93029	-2.23812	0.776116
C	-3.32539	-2.20888	-0.18665
C	-4.32177	-1.45097	-1.07863
C	-3.17616	-3.65322	-0.69395
C	3.521907	2.523541	-0.07889
C	-2.75791	2.626091	1.604973
O	-2.49776	3.321325	-0.70665
C	1.964519	-3.35564	0.233676
O	4.445967	-1.69059	-0.62355
H	0.632811	1.014	-1.30346
H	3.852039	0.346184	-1.84085
H	2.297124	-0.41989	-1.51467
H	1.184547	3.920112	-0.44477
H	-0.08909	3.37874	-1.51888
H	4.517598	0.01836	0.538367
H	-0.05754	2.562728	1.424888
H	-0.67761	4.124916	0.8902
H	-1.50531	0.997137	-1.22462
H	-2.49875	-0.01641	1.491946
H	-1.55388	-1.37537	-1.11072
H	2.454774	-0.22653	1.69501
H	3.438022	-1.6689	1.884374
H	0.289733	-0.52291	1.392373
H	-0.7707	-3.22497	0.336799
H	-1.39379	-2.41235	1.762072
H	-3.74591	-2.25023	0.830761
H	-5.28896	-1.96655	-1.10413
H	-4.48974	-0.42924	-0.72497
H	-3.95112	-1.389	-2.11061
H	-4.16244	-4.11253	-0.82913
H	-2.66518	-3.67614	-1.66574
H	-2.61362	-4.28772	-0.00244
H	3.119776	3.535281	0.013221
H	3.858069	2.215615	0.920495
H	4.422112	2.574656	-0.7065
H	-3.77217	2.241129	1.447977
H	-2.31467	2.07997	2.444552
H	-2.82981	3.682173	1.883035
H	-3.34729	2.918486	-0.95041
H	2.535795	-3.92737	0.980068
H	1.079279	-3.94212	-0.02319
H	2.604613	-3.27949	-0.65088
H	4.876907	-2.23201	0.057299

07_1S4R10S_5

C	-2.48772	1.634693	0.507849
C	-1.19271	1.87929	0.774675
C	-3.12433	0.343035	0.993491
C	-0.38389	3.115015	0.465461
C	-3.83114	-0.51903	-0.0719
C	0.676966	2.976988	-0.65753
C	2.037928	2.340034	-0.27904
C	1.871875	0.944612	0.28999

C	2.09592	-0.22209	-0.3236
C	1.792164	-1.5763	0.272471
C	-2.90218	-1.13819	-1.13291
C	-1.75412	-2.02107	-0.6666
C	-0.48993	-1.63185	-0.90905
C	0.79982	-2.36114	-0.63574
C	3.068012	-2.41885	0.583248
C	3.994505	-2.62505	-0.62651
C	3.850952	-1.82023	1.761181
C	-3.41578	2.597068	-0.19809
C	2.971395	2.386248	-1.49828
O	2.576394	3.195731	0.755273
C	-2.1368	-3.33909	-0.03407
O	-4.55603	-1.58608	0.555344
H	-0.65326	1.092107	1.297029
H	-3.8927	0.602795	1.742321
H	-2.38048	-0.27566	1.507371
H	-1.0495	3.937003	0.182227
H	0.134058	3.443292	1.376147
H	-4.54606	0.115385	-0.61925
H	0.255061	2.415909	-1.49972
H	0.906992	3.985311	-1.02654
H	1.493673	0.933747	1.312202
H	2.471251	-0.2272	-1.3475
H	1.283737	-1.41175	1.233026
H	-2.49868	-0.31055	-1.72786
H	-3.54936	-1.72203	-1.80449
H	-0.35722	-0.66116	-1.38657
H	0.618698	-3.33999	-0.17947
H	1.293001	-2.55809	-1.60024
H	2.707446	-3.41037	0.896073
H	4.816688	-3.30102	-0.36389
H	3.470694	-3.06344	-1.48299
H	4.441104	-1.67817	-0.95342
H	4.718424	-2.4423	2.012268
H	4.216907	-0.81491	1.52053
H	3.223877	-1.74095	2.657534
H	-2.96991	3.58308	-0.34806
H	-3.7125	2.220914	-1.1865
H	-4.3443	2.731361	0.373796
H	3.951551	1.961032	-1.25285
H	2.563426	1.827748	-2.34751
H	3.114366	3.425716	-1.80901
H	3.395396	2.778426	1.068907
H	-2.77395	-3.91893	-0.71654
H	-1.27096	-3.95526	0.220535
H	-2.73276	-3.17835	0.869854
H	-5.1533	-1.18556	1.207387

07_1S4R10S_6

C	2.530434	1.534846	-0.64615
C	1.23663	1.812854	-0.88371
C	3.096285	0.184015	-1.05018
C	0.489513	3.098972	-0.63175
C	3.76766	-0.63874	0.067961
C	-0.49742	3.089809	0.564432
C	-1.90687	2.495964	0.318034
C	-1.84424	1.071782	-0.19643
C	-2.14223	-0.05307	0.461012
C	-1.97058	-1.44997	-0.08779
C	2.811915	-1.14146	1.166338
C	1.618695	-1.99384	0.758371
C	0.375395	-1.53523	0.989769
C	-0.94384	-2.23488	0.783888
C	-3.3381	-2.19388	-0.18076
C	-4.32923	-1.43276	-1.07585
C	-3.19749	-3.64077	-0.68324
C	3.520808	2.506861	-0.04598
C	-2.74422	2.640941	1.597353
O	-2.48353	3.327157	-0.71701
C	1.937118	-3.36166	0.200218
O	4.439853	-1.77766	-0.4876
H	0.647199	1.012418	-1.32546
H	3.867841	0.353271	-1.82099
H	2.316514	-0.43031	-1.51381
H	1.198948	3.915451	-0.46142
H	-0.07687	3.371772	-1.53166
H	4.513478	-0.00475	0.572942
H	-0.04405	2.567485	1.415053
H	-0.65929	4.130151	0.875955
H	-1.49658	0.997664	-1.22673
H	-2.50043	-0.00165	1.491201
H	-1.56063	-1.37417	-1.10602
H	2.453524	-0.2585	1.708029
H	3.431078	-1.71434	1.872957
H	0.290972	-0.53501	1.412938
H	-0.79207	-3.22467	0.348597
H	-1.40851	-2.40164	1.770535
H	-3.7596	-2.22939	0.836441

H	-5.2997	-1.94221	-1.09997
H	-4.49079	-0.40872	-0.72592
H	-3.95778	-1.37684	-2.10791
H	-4.18652	-4.09411	-0.81856
H	-2.68508	-3.67012	-1.65412
H	-2.64019	-4.27663	0.011187
H	3.119197	3.518778	0.045847
H	3.83934	2.191863	0.956856
H	4.431742	2.560896	-0.658
H	-3.7598	2.258605	1.442596
H	-2.30197	2.096567	2.438559
H	-2.81253	3.698292	1.871467
H	-3.33444	2.925974	-0.9587
H	2.532444	-3.93829	0.922239
H	1.043802	-3.9443	-0.03737
H	2.554048	-3.27826	-0.69965
H	5.054636	-1.44988	-1.16365

07_1S4R10S_7

C	2.535654	1.515913	-0.62624
C	1.258901	1.815732	-0.92229
C	3.108306	0.17093	-1.03944
C	0.516143	3.104664	-0.66944
C	3.763854	-0.66806	0.075743
C	-0.46098	3.103759	0.534707
C	-1.87153	2.50702	0.304467
C	-1.81516	1.077196	-0.19492
C	-2.13326	-0.0381	0.469561
C	-1.98136	-1.44129	-0.06902
C	2.798797	-1.18172	1.160439
C	1.59286	-2.00661	0.734879
C	0.357886	-1.55568	1.019733
C	-0.96948	-2.23783	0.80959
C	-3.36007	-2.16433	-0.16145
C	-4.33706	-1.39403	-1.06419
C	-3.2404	-3.61654	-0.65397
C	3.501689	2.463128	0.048262
C	-2.70003	2.666975	1.58784
O	-2.45522	3.326832	-0.73604
C	1.888823	-3.34459	0.097779
O	4.437312	-1.80296	-0.48663
H	0.683973	1.036255	-1.41834
H	3.892146	0.351469	-1.79543
H	2.335553	-0.43552	-1.52489
H	1.229784	3.919457	-0.50682
H	-0.05522	3.376037	-1.56611
H	4.507509	-0.04313	0.594851
H	0.000203	2.588194	1.38517
H	-0.62108	4.146351	0.839667
H	-1.4619	0.989286	-1.22209
H	-2.49816	0.026668	1.496607
H	-1.5666	-1.37841	-1.08613
H	2.453174	-0.30802	1.725002
H	3.410309	-1.78064	1.852221
H	0.287653	-0.57702	1.493533
H	-0.82949	-3.23066	0.377126
H	-1.4395	-2.3956	1.794896
H	-3.78489	-2.18669	0.854737
H	-5.31536	-1.88835	-1.08699
H	-4.48332	-0.36516	-0.72177
H	-3.96252	-1.35114	-2.09576
H	-4.2359	-4.05513	-0.79041
H	-2.72453	-3.66037	-1.62244
H	-2.69612	-4.25647	0.046988
H	3.101556	3.474998	0.147035
H	3.767444	2.119715	1.057079
H	4.442639	2.526214	-0.51565
H	-3.71687	2.283534	1.444759
H	-2.25193	2.132012	2.43204
H	-2.76599	3.727424	1.850258
H	-3.30735	2.922401	-0.96783
H	2.50203	-3.96049	0.770454
H	0.985573	-3.90968	-0.14441
H	2.479932	-3.21805	-0.81448
H	5.058942	-1.46974	-1.15373

07_1S4R10S_8

C	-2.48171	1.652679	0.481924
C	-1.18574	1.908232	0.732931
C	-3.10625	0.366128	0.995528
C	-0.38611	3.138197	0.381586
C	-3.83111	-0.50944	-0.04688
C	0.715795	2.955824	-0.69431
C	2.070116	2.3325	-0.24713
C	1.890048	0.931462	0.308601
C	2.087931	-0.22985	-0.32174
C	1.775348	-1.58869	0.259462
C	-2.92108	-1.13598	-1.11995
C	-1.76825	-2.01978	-0.66757
C	-0.50623	-1.63097	-0.92199

C	0.784859	-2.36184	-0.65999
C	3.047874	-2.43762	0.567585
C	3.981187	-2.62851	-0.63938
C	3.824202	-1.85538	1.758033
C	-3.42179	2.596116	-0.23366
C	3.053207	2.38508	-1.42027
O	2.67382	3.167069	0.767048
C	-2.14511	-3.3389	-0.03355
O	-4.53887	-1.57187	0.606181
H	-0.63733	1.134972	1.266486
H	-3.8613	0.633613	1.754975
H	-2.35165	-0.24511	1.502566
H	-1.05278	3.931842	0.030011
H	0.079789	3.54055	1.294641
H	-4.55911	0.116824	-0.58618
H	0.312952	2.364439	-1.52588
H	0.958626	3.949512	-1.09276
H	1.514862	0.900014	1.333929
H	2.456911	-0.22524	-1.34788
H	1.263366	-1.43237	1.219676
H	-2.52515	-0.31216	-1.72527
H	-3.58039	-1.72139	-1.7781
H	-0.37694	-0.65967	-1.3995
H	0.605108	-3.34747	-0.21813
H	1.278354	-2.54377	-1.6272
H	2.683621	-3.43265	0.864759
H	4.800648	-3.30951	-0.38128
H	3.461644	-3.05403	-1.50502
H	4.431078	-1.67773	-0.94977
H	4.690622	-2.48078	2.004513
H	4.190368	-0.84646	1.534195
H	3.192296	-1.79044	2.652294
H	-2.98814	3.584976	-0.39935
H	-3.71896	2.202694	-1.21505
H	-4.34871	2.728469	0.340953
H	4.012366	1.943277	-1.13462
H	2.663862	1.848889	-2.29093
H	3.222903	3.427238	-1.7084
H	2.16621	3.053072	1.58647
H	-2.7967	-3.91311	-0.70701
H	-1.27752	-3.95971	0.202972
H	-2.72391	-3.17963	0.881699
H	-5.1298	-1.16639	1.260881

07_1S4R10S_9

C	2.539468	1.55359	-0.60152
C	1.249836	1.860738	-0.82497
C	3.080692	0.208191	-1.05414
C	0.524183	3.145617	-0.51369
C	3.764918	-0.65123	0.028295
C	-0.53242	3.074581	0.61926
C	-1.93522	2.497226	0.272989
C	-1.85625	1.064934	-0.22244
C	-2.13886	-0.05226	0.452467
C	-1.96051	-1.45705	-0.07366
C	2.829461	-1.15847	1.142061
C	1.623027	-2.00069	0.752358
C	0.386131	-1.53403	1.001718
C	-0.93878	-2.22805	0.814936
C	-3.32856	-2.2011	-0.16374
C	-4.30845	-1.45877	-1.08668
C	-3.18649	-3.65978	-0.63065
C	3.548637	2.485875	0.029659
C	-2.84993	2.65622	1.490887
O	-2.55177	3.313252	-0.74959
C	1.924561	-3.37081	0.190368
O	4.399766	-1.78933	-0.57039
H	0.646272	1.088393	-1.29663
H	3.839002	0.389434	-1.83516
H	2.284922	-0.38487	-1.51834
H	1.241685	3.924511	-0.23747
H	0.035977	3.517294	-1.42804
H	4.536053	-0.04221	0.525783
H	-0.11668	2.509781	1.462907
H	-0.70852	4.098725	0.973062
H	-1.5013	0.964224	-1.25039
H	-2.50033	0.014172	1.480533
H	-1.54477	-1.39712	-1.09072
H	2.48879	-0.27844	1.699763
H	3.459452	-1.74214	1.829938
H	0.312954	-0.53234	1.423965
H	-0.79458	-3.22898	0.403418
H	-1.40254	-2.36795	1.806034
H	-3.76049	-2.21205	0.849595
H	-5.28141	-1.96378	-1.10438
H	-4.46668	-0.42441	-0.76748
H	-3.92889	-1.43429	-2.11711
H	-4.17547	-4.11259	-0.76804
H	-2.66204	-3.71364	-1.59405

H	-2.6406	-4.28145	0.085424
H	3.170187	3.503368	0.151921
H	3.858885	2.133041	1.022319
H	4.460915	2.538527	-0.58018
H	-3.84423	2.253113	1.277053
H	-2.4419	2.141415	2.365948
H	-2.95146	3.718248	1.735213
H	-2.10125	3.128727	-1.58923
H	2.528651	-3.95016	0.902869
H	1.02471	-3.94901	-0.03273
H	2.527031	-3.28996	-0.71935
H	5.010938	-1.4575	-1.24772

07_1S4R10S_10

C	-2.48246	1.651957	0.482963
C	-1.1862	1.90676	0.733232
C	-3.10703	0.364979	0.995472
C	-0.38651	3.137013	0.382964
C	-3.83115	-0.51007	-0.04791
C	0.714615	2.955865	-0.69394
C	2.069526	2.332912	-0.24817
C	1.890129	0.932061	0.308155
C	2.08857	-0.22946	-0.32163
C	1.77617	-1.58813	0.260105
C	-2.92024	-1.13603	-1.12057
C	-1.76756	-2.01978	-0.66771
C	-0.50545	-1.63078	-0.92136
C	0.78557	-2.36162	-0.65897
C	3.048739	-2.43689	0.568324
C	3.981873	-2.62824	-0.63871
C	3.825284	-1.85418	1.758408
C	-3.42284	2.596523	-0.23072
C	3.051358	2.385397	-1.42233
O	2.674151	3.167882	0.765173
C	-2.14462	-3.33905	-0.03415
O	-4.53939	-1.57286	0.604069
H	-0.63764	1.132584	1.265293
H	-3.86249	0.631846	1.754724
H	-2.35258	-0.24646	1.502484
H	-1.05327	3.9313	0.033029
H	0.080049	3.537932	1.296321
H	-4.55876	0.116448	-0.58743
H	0.311389	2.3648	-1.52555
H	0.956716	3.949882	-1.09201
H	1.514679	0.900945	1.333402
H	2.457739	-0.2252	-1.34769
H	1.264306	-1.43147	1.220329
H	-2.52405	-0.31191	-1.72531
H	-3.57898	-1.72131	-1.77942
H	-0.37602	-0.65937	-1.39863
H	0.60574	-3.34702	-0.21664
H	1.279051	-2.54407	-1.6261
H	2.684583	-3.43183	0.865952
H	4.801394	-3.30911	-0.38046
H	3.462231	-3.05413	-1.50411
H	4.431691	-1.67758	-0.94956
H	4.691726	-2.47951	2.005007
H	4.191447	-0.84537	1.534087
H	3.193528	-1.78885	2.652744
H	-2.98866	3.585118	-0.39661
H	-3.7218	2.203811	-1.21185
H	-4.34879	2.729321	0.345362
H	4.01095	1.943975	-1.13754
H	2.661265	1.848822	-2.29242
H	3.220467	3.427505	-1.71099
H	2.167445	3.053949	1.585159
H	-2.79531	-3.91348	-0.70831
H	-1.27707	-3.95958	0.203258
H	-2.72443	-3.18002	0.880508
H	-5.13121	-1.16769	1.258157

07_1S4R10S_11

C	-0.72292	-2.84288	-0.10772
C	0.4242	-2.41344	-0.66284
C	-2.0332	-2.18854	-0.50168
C	1.824183	-2.91525	-0.41986
C	-2.66024	-1.30994	0.602439
C	2.738523	-2.01928	0.449045
C	3.122481	-0.62208	-0.11868
C	2.044637	0.425146	0.131078
C	1.687326	1.381348	-0.73276
C	0.68283	2.505036	-0.53638
C	-3.51023	-0.16244	0.036406
C	-2.7936	0.998925	-0.63455
C	-1.45422	1.088911	-0.70624
C	-0.62365	2.182454	-1.32913
C	0.437403	2.922379	0.938056
C	1.721859	3.470888	1.583746
C	-0.68149	3.969925	1.065892
C	-0.82554	-3.94569	0.917802

C	4.416975	-0.15068	0.569026
O	3.463903	-0.71627	-1.51001
C	-3.74569	2.024202	-1.20782
O	-3.4773	-2.17664	1.40611
H	0.34229	-1.60594	-1.39132
H	-2.78414	-2.94917	-0.75929
H	-1.87784	-1.57528	-1.39411
H	1.799052	-3.90251	0.053615
H	2.322674	-3.06452	-1.38803
H	-1.86116	-0.88881	1.230032
H	2.285137	-1.88398	1.439562
H	3.67187	-2.57578	0.60404
H	1.619045	0.401191	1.133704
H	2.154631	1.366533	-1.71869
H	1.113917	3.385266	-1.04016
H	-4.1155	0.247418	0.862179
H	-4.24377	-0.59084	-0.66451
H	-0.86099	0.280375	-0.28666
H	-0.32063	1.879663	-2.34381
H	-1.2022	3.102008	-1.45588
H	0.119072	2.034038	1.501996
H	1.55095	3.717878	2.638365
H	2.5481	2.756898	1.531525
H	2.04258	4.390825	1.076961
H	-0.77359	4.302154	2.106753
H	-0.4635	4.856805	0.45584
H	-1.65399	3.575226	0.757551
H	-1.35613	-3.59192	1.809318
H	-1.42223	-4.78022	0.523276
H	0.145919	-4.34001	1.225665
H	4.709091	0.832232	0.187419
H	4.277168	-0.07685	1.6528
H	5.227091	-0.86163	0.371773
H	2.641804	-0.87991	-2.00016
H	-4.45397	2.371905	-0.44336
H	-3.23919	2.898991	-1.62173
H	-4.35112	1.580545	-2.01065
H	-3.78112	-1.6633	2.172159
07_1S4R10S_12			
C	-2.58703	1.466002	0.50935
C	-1.34526	1.841994	0.86284
C	-3.1114	0.110823	0.951928
C	-0.65321	3.159953	0.61469
C	-3.70701	-0.78664	-0.14268
C	0.413686	3.171958	-0.51095
C	1.825838	2.63705	-0.16408
C	1.781968	1.21469	0.357531
C	2.129755	0.094511	-0.28348
C	1.970877	-1.29688	0.28483
C	-2.69965	-1.29381	-1.20058
C	-1.4812	-2.07052	-0.72769
C	-0.25517	-1.56762	-0.95532
C	1.094116	-2.17073	-0.6597
C	3.350381	-1.94425	0.627662
C	3.194762	-3.24007	1.440032
C	4.258481	-2.17294	-0.59168
C	-3.56245	2.339233	-0.24616
C	2.741211	2.803429	-1.38643
O	2.300065	3.497623	0.897704
C	-1.75122	-3.41783	-0.09873
O	-4.35993	-1.85725	0.556609
H	-0.76192	1.108688	1.41612
H	-3.92032	0.26506	1.682505
H	-2.32034	-0.4448	1.46799
H	-0.169	3.49333	1.541618
H	-1.39288	3.928637	0.366281
H	-4.47005	-0.21312	-0.69248
H	0.038763	2.618944	-1.38012
H	0.554709	4.213486	-0.82892
H	1.393196	1.137376	1.372922
H	2.518351	0.153743	-1.30055
H	1.428411	-1.20453	1.236434
H	-2.36993	-0.42231	-1.77919
H	-3.27144	-1.92372	-1.90337
H	-0.20724	-0.58942	-1.43343
H	0.994571	-3.17235	-0.23248
H	1.630295	-2.29407	-1.61315
H	3.856209	-1.21507	1.277336
H	4.170094	-3.58378	1.804673
H	2.545423	-3.09015	2.311253
H	2.768095	-4.0519	0.83912
H	5.23795	-2.5465	-0.27017
H	3.836923	-2.91569	-1.27962
H	4.427534	-1.24949	-1.15656
H	-3.20427	3.364079	-0.36953
H	-3.76564	1.94365	-1.25049
H	-4.53023	2.379519	0.272422
H	3.755802	2.45446	-1.16164

H	2.373998	2.240704	-2.25134
H	2.793228	3.860994	-1.66318
H	3.152555	3.137883	1.19309
H	-2.28485	-4.06946	-0.80645
H	-0.8388	-3.93671	0.204701
H	-2.40352	-3.31435	0.774133
H	-4.73159	-2.45628	-0.11075

07_1S4R10S_13

C	-2.59867	1.487332	0.45825
C	-1.35261	1.883768	0.7716
C	-3.0993	0.141121	0.952519
C	-0.67721	3.196199	0.460922
C	-3.71023	-0.79183	-0.10341
C	0.470247	3.148585	-0.58058
C	1.861763	2.636412	-0.10839
C	1.801324	1.203417	0.38872
C	2.129541	0.091378	-0.27433
C	1.962736	-1.30823	0.271378
C	-2.72255	-1.30818	-1.17537
C	-1.4917	-2.07467	-0.7181
C	-0.27144	-1.56428	-0.96018
C	1.083107	-2.16517	-0.68494
C	3.340651	-1.96529	0.602966
C	3.182566	-3.26551	1.407796
C	4.24058	-2.19064	-0.62297
C	-3.60149	2.325579	-0.30111
C	2.872535	2.82718	-1.24364
O	2.351926	3.482627	0.956243
C	-1.74547	-3.42352	-0.08549
O	-4.32753	-1.85335	0.640318
H	-0.75114	1.17365	1.335159
H	-3.89308	0.309346	1.696252
H	-2.29205	-0.39337	1.465793
H	-1.41312	3.91994	0.096707
H	-0.28521	3.631172	1.393467
H	-4.49651	-0.24331	-0.64594
H	0.149636	2.550345	-1.44249
H	0.632285	4.172849	-0.94116
H	1.409654	1.095829	1.402707
H	2.518131	0.166001	-1.29039
H	1.421772	-1.22862	1.225153
H	-2.40892	-0.44222	-1.77103
H	-3.30533	-1.9493	-1.85857
H	-0.23347	-0.58438	-1.43594
H	0.991217	-3.17624	-0.27864
H	1.613205	-2.26605	-1.6443
H	3.853803	-1.24304	1.254316
H	4.15832	-3.61696	1.763845
H	2.539913	-3.1175	2.284331
H	2.747513	-4.07133	0.804772
H	5.220734	-2.56889	-0.3092
H	3.812436	-2.92843	-1.31224
H	4.409426	-1.2646	-1.18352
H	-3.26451	3.352532	-0.46038
H	-3.81874	1.897851	-1.28904
H	-4.55851	2.36505	0.236817
H	3.858284	2.460823	-0.94221
H	2.56081	2.29552	-2.14774
H	2.955434	3.891681	-1.48457
H	1.836845	3.285416	1.754891
H	-2.28136	-4.08014	-0.78689
H	-0.82686	-3.93549	0.210653
H	-2.38976	-3.32159	0.79347
H	-4.71238	-2.47274	-0.0005

07_1S4R10S_14

C	-2.59455	1.452104	0.503737
C	-1.35574	1.82953	0.86578
C	-3.1199	0.093176	0.935542
C	-0.66694	3.150592	0.624652
C	-3.70667	-0.80912	-0.16827
C	0.397823	3.173225	-0.50272
C	1.812799	2.64445	-0.15884
C	1.775621	1.220737	0.359145
C	2.13073	0.104031	-0.2838
C	1.978899	-1.28927	0.281296
C	-2.69156	-1.31844	-1.20865
C	-1.46772	-2.07883	-0.7195
C	-0.24545	-1.57282	-0.96146
C	1.108646	-2.16605	-0.66632
C	3.361784	-1.92894	0.625289
C	3.212558	-3.22732	1.434756
C	4.273287	-2.1498	-0.59297
C	-3.56657	2.324501	-0.25717
C	2.726129	2.818669	-1.38152
O	2.283772	3.50448	0.905025
C	-1.72423	-3.41763	-0.06775
O	-4.34537	-1.9531	0.416103
H	-0.77375	1.096254	1.420582

H	-3.93273	0.259913	1.663871
H	-2.33558	-0.46262	1.461444
H	-1.40929	3.918863	0.383079
H	-0.18203	3.478865	1.55296
H	-4.46218	-0.23564	-0.72816
H	0.024866	2.621505	-1.37351
H	0.532671	4.216778	-0.81663
H	1.385464	1.138754	1.373665
H	2.521004	0.167713	-1.29993
H	1.434397	-1.20194	1.232285
H	-2.36302	-0.44968	-1.79087
H	-3.25885	-1.96247	-1.89743
H	-0.20457	-0.60108	-1.45352
H	1.016552	-3.16962	-0.24209
H	1.646193	-2.2828	-1.61977
H	3.862246	-1.19806	1.277279
H	4.189415	-3.5664	1.799683
H	2.561489	-3.08279	2.305623
H	2.790892	-4.04015	0.831726
H	5.254467	-2.51811	-0.27058
H	3.85727	-2.89364	-1.28306
H	4.437777	-1.22425	-1.15576
H	-3.2092	3.349992	-0.37703
H	-3.76223	1.929632	-1.26317
H	-4.53819	2.363683	0.25465
H	3.742632	2.47402	-1.15875
H	2.36071	2.256616	-2.2476
H	2.772722	3.877268	-1.65533
H	3.138387	3.148088	1.198347
H	-2.28009	-4.07497	-0.75116
H	-0.80407	-3.93261	0.219074
H	-2.35559	-3.30359	0.818826
H	-4.99882	-1.62541	1.054967

07_1S4R10S_15

C	-2.51268	-1.44358	0.393823
C	-1.6895	-2.26867	-0.27534
C	-3.70348	-0.82808	-0.31841
C	-0.56679	-3.12799	0.255376
C	-3.91275	0.699831	-0.23782
C	0.70033	-3.16558	-0.64262
C	1.962981	-2.44808	-0.10655
C	1.717812	-0.96957	0.138845
C	2.252501	0.023961	-0.57833
C	2.089804	1.516215	-0.34873
C	-2.84141	1.532016	-0.97364
C	-1.68055	2.010989	-0.12248
C	-0.45659	1.484032	-0.27969
C	0.812627	1.868781	0.446403
C	3.37395	2.132015	0.299027
C	4.663883	1.669156	-0.3999
C	3.338412	3.670197	0.319764
C	-2.41551	-1.15414	1.873205
C	2.47989	-3.11144	1.185111
O	2.931354	-2.65525	-1.1489
C	-2.003	3.126822	0.845033
O	-5.16053	1.026247	-0.87256
H	-1.88418	-2.40771	-1.34085
H	-3.69179	-1.10329	-1.38002
H	-4.61258	-1.28518	0.110864
H	-0.30557	-2.84554	1.279361
H	-0.94962	-4.15666	0.331163
H	-3.95778	1.008017	0.817612
H	0.995347	-4.20634	-0.82137
H	0.47731	-2.73402	-1.62525
H	1.060173	-0.75049	0.978012
H	2.884451	-0.25186	-1.42055
H	1.995209	1.979356	-1.34498
H	-3.36714	2.408405	-1.37734
H	-2.47084	0.959994	-1.83249
H	-0.35009	0.679239	-1.00444
H	0.805205	2.941798	0.657023
H	0.862875	1.378174	1.431306
H	3.410258	1.773349	1.339258
H	5.537778	2.155005	0.049343
H	4.809565	0.587555	-0.32264
H	4.65099	1.931884	-1.46619
H	4.266425	4.067194	0.747824
H	3.245743	4.070977	-0.69833
H	2.510873	4.070086	0.913297
H	-1.63954	-1.73953	2.37159
H	-2.19797	-0.09355	2.05192
H	-3.36883	-1.37045	2.375288
H	3.422529	-2.64619	1.49848
H	1.773727	-3.00683	2.015555
H	2.659135	-4.17811	1.012231
H	3.767032	-2.26421	-0.84393
H	-2.81165	2.842285	1.531256
H	-1.14546	3.421477	1.454908

H	-2.35874	4.016174	0.305597
H	-5.84919	0.479748	-0.46036
07_1S4R10S_16			
C	-2.77817	1.328766	-0.20525
C	-1.46684	1.59477	-0.3305
C	-3.38372	0.172803	-0.98495
C	-0.66015	2.69206	0.322932
C	-3.85632	-1.0571	-0.18403
C	0.528932	3.173483	-0.54015
C	1.89598	2.513167	-0.23709
C	1.866907	0.99939	-0.37804
C	2.105999	0.125061	0.605197
C	2.171504	-1.3928	0.469916
C	-2.78934	-1.67149	0.74743
C	-1.54737	-2.19966	0.054692
C	-0.35822	-1.62055	0.284248
C	0.978258	-1.96428	-0.33389
C	3.554018	-1.84284	-0.09785
C	4.714953	-1.27852	0.738245
C	3.692656	-3.3715	-0.19626
C	-3.72629	2.133343	0.651891
C	2.957014	3.083257	-1.20078
O	2.214596	2.913605	1.10449
C	-1.74597	-3.36965	-0.88073
O	-5.01093	-0.6701	0.573566
H	-0.88985	0.933363	-0.97522
H	-4.25823	0.52848	-1.54952
H	-2.6554	-0.18408	-1.72216
H	-1.30294	3.550438	0.548708
H	-0.26521	2.355978	1.291832
H	-4.15828	-1.81583	-0.92422
H	0.30039	3.033882	-1.60416
H	0.676305	4.249939	-0.39173
H	1.669869	0.644856	-1.39078
H	2.291286	0.5234	1.601579
H	2.100477	-1.80868	1.48673
H	-3.27799	-2.49722	1.291999
H	-2.51263	-0.9176	1.49237
H	-0.34457	-0.77403	0.967537
H	1.08381	-3.05099	-0.41653
H	1.029797	-1.58495	-1.36668
H	3.636462	-1.42478	-1.11291
H	5.679643	-1.58774	0.319322
H	4.699677	-0.18455	0.771492
H	4.668662	-1.64795	1.771517
H	4.705579	-3.6414	-0.51779
H	3.519032	-3.8447	0.779401
H	2.995349	-3.81458	-0.91367
H	-3.24529	3.000075	1.11317
H	-4.57727	2.490683	0.055289
H	-4.15484	1.510683	1.444829
H	3.95039	2.688634	-0.95461
H	2.739475	2.813695	-2.24045
H	2.987912	4.175416	-1.12307
H	3.08348	2.535656	1.318517
H	-2.29385	-3.0762	-1.78706
H	-0.79919	-3.81093	-1.20395
H	-2.34103	-4.15861	-0.40056
H	-5.29342	-1.44471	1.086153
07_1S4R10S_17			
C	-2.61525	1.463395	0.454344
C	-1.37372	1.866736	0.776443
C	-3.11029	0.109537	0.935075
C	-0.70646	3.185016	0.472906
C	-3.70653	-0.82912	-0.13301
C	0.43886	3.151278	-0.57145
C	1.835701	2.650317	-0.10363
C	1.788449	1.215668	0.389241
C	2.132416	0.108958	-0.27472
C	1.978446	-1.2934	0.267205
C	-2.70517	-1.34361	-1.18418
C	-1.46628	-2.08756	-0.70756
C	-0.25189	-1.57199	-0.96892
C	1.109693	-2.15709	-0.69282
C	3.362391	-1.93694	0.600734
C	3.215617	-3.24043	1.402413
C	4.267295	-2.15076	-0.62362
C	-3.61896	2.297229	-0.30881
C	2.842322	2.85405	-1.24024
O	2.319781	3.498115	0.962803
C	-1.70095	-3.42489	-0.04453
O	-4.31137	-1.96872	0.493743
H	-0.77044	1.158702	1.34077
H	-3.90956	0.283822	1.676225
H	-2.30688	-0.42171	1.457538
H	-1.4476	3.906541	0.115023
H	-0.31519	3.615918	1.407525
H	-4.48462	-0.28234	-0.68862

H	0.121635	2.553056	-1.43456
H	0.590602	4.178214	-0.92884
H	1.392676	1.100712	1.400867
H	2.52539	0.190224	-1.28853
H	1.434458	-1.2214	1.219939
H	-2.39496	-0.47942	-1.78306
H	-3.27779	-2.00176	-1.8549
H	-0.22468	-0.60142	-1.46441
H	1.029239	-3.17018	-0.28921
H	1.642125	-2.2495	-1.65163
H	3.866863	-1.21091	1.254718
H	4.194225	-3.5836	1.75872
H	2.570634	-3.10039	2.278565
H	2.788598	-4.04878	0.79709
H	5.250651	-2.51939	-0.3084
H	3.848295	-2.89167	-1.31512
H	4.427889	-1.22208	-1.18226
H	-3.28826	3.327148	-0.4616
H	-3.82601	1.871908	-1.29986
H	-4.58036	2.328671	0.22215
H	3.83173	2.494532	-0.94263
H	2.532884	2.324041	-2.14611
H	2.915976	3.920292	-1.47654
H	1.816378	3.2853	1.764826
H	-2.26139	-4.09143	-0.71515
H	-0.77228	-3.93	0.232024
H	-2.31962	-3.30992	0.850759
H	-4.9622	-1.63668	1.133023

07_1S4R10S_18

C	2.547388	1.637417	-0.45018
C	1.230127	1.83062	-0.2744
C	3.036138	0.330895	-1.04952
C	0.517178	3.03687	0.287831
C	3.794753	-0.58775	-0.07852
C	-0.84645	3.326866	-0.377
C	-2.06256	2.47047	0.056241
C	-1.90296	1.015679	-0.34035
C	-2.04601	-0.06752	0.431275
C	-1.88415	-1.49698	-0.03392
C	2.926161	-1.1708	1.062154
C	1.7022	-1.99505	0.692071
C	0.476803	-1.55152	1.027718
C	-0.85232	-2.24351	0.863562
C	-3.25912	-2.23832	-0.06162
C	-4.25536	-1.54858	-1.00718
C	-3.12906	-3.72136	-0.45054
C	3.614898	2.648597	-0.10286
C	-2.39977	2.676671	1.538398
O	-3.14039	3.022808	-0.74507
C	1.979212	-3.32286	0.025376
O	4.400162	-1.60104	-0.89485
H	0.578319	1.008032	-0.55449
H	3.725107	0.540845	-1.88006
H	2.192767	-0.23055	-1.46652
H	0.37381	2.916261	1.372449
H	1.141156	3.931452	0.178814
H	4.594018	-0.00531	0.407873
H	-1.12591	4.369058	-0.17823
H	-0.74776	3.237066	-1.46617
H	-1.67261	0.879039	-1.3986
H	-2.28696	0.050998	1.489503
H	-1.49144	-1.48328	-1.0615
H	2.618857	-0.3286	1.693003
H	3.592869	-1.79062	1.685145
H	0.421236	-0.57728	1.512933
H	-0.71219	-3.25398	0.474471
H	-1.30611	-2.35983	1.861958
H	-3.67326	-2.19275	0.958167
H	-5.22481	-2.06007	-0.98532
H	-4.41864	-0.5011	-0.73772
H	-3.89073	-1.57299	-2.04277
H	-4.12175	-4.17678	-0.54584
H	-2.62171	-3.82995	-1.41833
H	-2.57301	-4.30487	0.289255
H	3.200099	3.617125	0.188539
H	4.244099	2.301589	0.728359
H	4.289388	2.810381	-0.95468
H	-3.33187	2.162102	1.799179
H	-1.61486	2.300511	2.20222
H	-2.53743	3.744636	1.734765
H	-3.91953	2.465954	-0.58155
H	2.60303	-3.95478	0.674701
H	1.07142	-3.8834	-0.20948
H	2.548023	-3.1752	-0.89813
H	4.875606	-2.20697	-0.30384

07_1S4R10S_19

C	-2.25681	-1.61664	-0.59267
C	-1.00882	-1.87557	-1.01831

C	-2.93565	-0.31735	-0.99794
C	-0.21272	-3.15201	-0.86369
C	-3.70865	0.456993	0.088688
C	1.325623	-3.00508	-0.88934
C	2.033253	-2.36803	0.331816
C	1.814383	-0.86717	0.479329
C	1.784334	0.025753	-0.51547
C	1.586047	1.524271	-0.41082
C	-2.85845	1.010887	1.247998
C	-1.64	1.846763	0.893019
C	-0.41822	1.360617	1.169604
C	0.929207	1.991761	0.915045
C	2.912386	2.272681	-0.76626
C	2.676137	3.755295	-1.09485
C	4.004825	2.111815	0.301362
C	-3.10267	-2.59974	0.184263
C	1.645004	-3.0802	1.630866
O	3.458115	-2.62113	0.198745
C	-1.90324	3.205217	0.288317
O	-4.40014	1.566199	-0.5048
H	-0.51699	-1.10138	-1.6055
H	-3.6754	-0.55492	-1.78302
H	-2.20423	0.359174	-1.45334
H	-0.51244	-3.69991	0.036056
H	-0.46623	-3.81797	-1.70427
H	-4.45248	-0.21764	0.541475
H	1.756883	-4.00866	-0.9864
H	1.619904	-2.46326	-1.79888
H	1.719373	-0.52096	1.507387
H	1.907688	-0.32641	-1.54178
H	0.87514	1.785622	-1.21034
H	-2.53947	0.155509	1.854832
H	-3.54311	1.60335	1.872852
H	-0.38112	0.365326	1.60613
H	0.845059	3.082823	0.888883
H	1.594167	1.759279	1.757108
H	3.283601	1.797474	-1.6874
H	3.597223	4.21758	-1.4693
H	1.90383	3.876462	-1.86425
H	2.363421	4.324739	-0.21158
H	4.954101	2.528579	-0.0553
H	3.743832	2.639297	1.226708
H	4.17028	1.057764	0.549669
H	-2.61057	-3.56634	0.314622
H	-3.35802	-2.22071	1.182857
H	-4.05685	-2.78067	-0.33058
H	2.265309	-2.7084	2.453028
H	0.595611	-2.90087	1.883732
H	1.80459	-4.16007	1.541047
H	3.775502	-2.04878	-0.51847
H	-2.53602	3.806626	0.956122
H	-0.9855	3.767276	0.094939
H	-2.46024	3.11099	-0.64965
H	-4.93651	1.216934	-1.23473
07_1S4R10S_20			
C	2.564153	1.616778	-0.45054
C	1.252602	1.805652	-0.23256
C	3.040255	0.298272	-1.03572
C	0.548741	3.016373	0.329366
C	3.786648	-0.62792	-0.05212
C	-0.80045	3.330057	-0.35548
C	-2.03519	2.488456	0.053269
C	-1.88629	1.031972	-0.34153
C	-2.05083	-0.04938	0.428348
C	-1.89857	-1.48108	-0.03313
C	2.907748	-1.20684	1.074662
C	1.673764	-2.0133	0.693608
C	0.454824	-1.56143	1.042542
C	-0.8831	-2.23629	0.875802
C	-3.28162	-2.20654	-0.07436
C	-4.25571	-1.51169	-1.03916
C	-3.16501	-3.69408	-0.45002
C	3.640242	2.637013	-0.16118
C	-2.39787	2.697629	1.528952
O	-3.09039	3.055265	-0.76788
C	1.930581	-3.33689	0.011438
O	4.388278	-1.72447	-0.75352
H	0.596997	0.974022	-0.47447
H	3.73133	0.508609	-1.86843
H	2.195233	-0.25726	-1.45714
H	0.385187	2.885042	1.409858
H	1.185826	3.903498	0.240104
H	4.582271	-0.04834	0.443809
H	-1.06788	4.375088	-0.15499
H	-0.68545	3.244865	-1.44338
H	-1.64255	0.892898	-1.39647
H	-2.30454	0.072419	1.483203
H	-1.49489	-1.47308	-1.05655

H	2.604909	-0.36467	1.70646
H	3.568449	-1.84006	1.685545
H	0.411713	-0.59332	1.541271
H	-0.75479	-3.25085	0.493385
H	-1.34441	-2.34031	1.871985
H	-3.70948	-2.14799	0.939065
H	-5.23326	-2.00777	-1.02389
H	-4.40565	-0.45892	-0.78284
H	-3.87893	-1.553	-2.06989
H	-4.16205	-4.13702	-0.55802
H	-2.6436	-3.8171	-1.40857
H	-2.62886	-4.27901	0.303162
H	3.235689	3.59633	0.171286
H	4.328115	2.285649	0.62
H	4.253785	2.819273	-1.05434
H	-3.34029	2.193235	1.771881
H	-1.6298	2.312241	2.206965
H	-2.52717	3.766949	1.723567
H	-3.88098	2.511341	-0.61612
H	2.569806	-3.9729	0.639939
H	1.014254	-3.89108	-0.20557
H	2.480946	-3.18658	-0.92228
H	4.937446	-1.34922	-1.46054

07_1S4R10S_21

C	2.551426	1.630178	-0.45515
C	1.241803	1.808018	-0.21669
C	3.031239	0.309725	-1.03347
C	0.534106	3.016626	0.344243
C	3.785704	-0.60728	-0.04691
C	-0.82168	3.324867	-0.33491
C	-2.05841	2.473865	0.064527
C	-1.89131	1.018089	-0.32861
C	-2.04364	-0.06607	0.437416
C	-1.88354	-1.49451	-0.03014
C	2.911424	-1.19511	1.079008
C	1.684202	-2.01113	0.696386
C	0.462317	-1.57283	1.052241
C	-0.8719	-2.25354	0.879102
C	-3.2683	-2.2155	-0.08464
C	-4.22974	-1.51244	-1.05668
C	-3.15432	-3.7028	-0.46094
C	3.622758	2.663447	-0.19611
C	-2.43097	2.676215	1.533404
O	-3.19442	3.006626	-0.66683
C	1.950125	-3.32797	0.004626
O	4.399879	-1.69829	-0.74594
H	0.591296	0.966197	-0.43597
H	3.718165	0.517879	-1.87006
H	2.187419	-0.25247	-1.44831
H	0.371211	2.88479	1.424771
H	1.166493	3.906883	0.25355
H	4.574191	-0.01862	0.449726
H	-1.09489	4.366944	-0.12853
H	-0.69444	3.249616	-1.42519
H	-1.63212	0.874202	-1.38137
H	-2.30432	0.05078	1.490873
H	-1.47394	-1.48078	-1.05137
H	2.601246	-0.35661	1.712095
H	3.577833	-1.82343	1.688739
H	0.412457	-0.60927	1.559196
H	-0.73914	-3.26582	0.491849
H	-1.33647	-2.36353	1.873012
H	-3.7048	-2.15622	0.924965
H	-5.21423	-1.99469	-1.0413
H	-4.36356	-0.45565	-0.80849
H	-3.85022	-1.56843	-2.08604
H	-4.15215	-4.14183	-0.57764
H	-2.62553	-3.82691	-1.41537
H	-2.62641	-4.29055	0.296014
H	3.216843	3.618268	0.147333
H	4.335738	2.319778	0.565636
H	4.209436	2.852045	-1.10589
H	-3.36902	2.161172	1.760687
H	-1.65601	2.297336	2.205277
H	-2.57184	3.74341	1.732226
H	-3.00503	2.8945	-1.6133
H	2.601419	-3.96014	0.624466
H	1.037858	-3.89084	-0.20712
H	2.490862	-3.16777	-0.93313
H	4.95022	-1.31815	-1.4494

07_1S4R10S_22

C	-0.62068	-2.83897	-0.10372
C	0.503486	-2.40984	-0.70403
C	-1.95807	-2.24356	-0.49701
C	1.920784	-2.86153	-0.46294
C	-2.63498	-1.38557	0.60597
C	2.797392	-1.94572	0.42433
C	3.136519	-0.52958	-0.12428

C	2.021889	0.477464	0.128724
C	1.642112	1.431887	-0.7275
C	0.600514	2.520732	-0.52853
C	-3.51421	-0.27604	0.025492
C	-2.82685	0.904587	-0.63966
C	-1.49083	1.038476	-0.70342
C	-0.69395	2.158274	-1.32365
C	0.338912	2.924133	0.94717
C	1.604076	3.508963	1.598791
C	-0.81201	3.936479	1.075364
C	-0.65426	-3.88173	0.990063
C	4.409399	-0.02292	0.578565
O	3.490316	-0.59646	-1.51406
C	-3.80757	1.897462	-1.22199
O	-3.50851	-2.17258	1.430667
H	0.384261	-1.64305	-1.47015
H	-2.67426	-3.03649	-0.75803
H	-1.82381	-1.62923	-1.39264
H	1.931061	-3.85565	-0.0025
H	2.427551	-2.97757	-1.43063
H	-1.85876	-0.93003	1.239209
H	2.333335	-1.83895	1.413358
H	3.748019	-2.47217	0.578632
H	1.588542	0.428318	1.12709
H	2.119905	1.443906	-1.70839
H	1.003125	3.416597	-1.02819
H	-4.14259	0.102434	0.845393
H	-4.21936	-0.7305	-0.68835
H	-0.87354	0.251123	-0.27805
H	-0.38081	1.868237	-2.33903
H	-1.30057	3.059847	-1.44787
H	0.045871	2.024465	1.506811
H	1.422154	3.746848	2.653642
H	2.451744	2.82048	1.546995
H	1.898459	4.440037	1.096339
H	-0.91799	4.261024	2.117306
H	-0.61879	4.832197	0.469857
H	-1.77079	3.51338	0.761818
H	0.29696	-4.40563	1.112775
H	-0.88913	-3.4312	1.966415
H	-1.42829	-4.63456	0.789877
H	4.66864	0.974841	0.212386
H	4.260091	0.030823	1.662225
H	5.24545	-0.70206	0.377159
H	2.675325	-0.76871	-2.01313
H	-4.53006	2.224923	-0.46209
H	-3.32669	2.786194	-1.63669
H	-4.39477	1.43209	-2.02611
H	-2.97485	-2.86774	1.845134

07_1S4R10S_23

C	-2.32137	1.671224	0.578453
C	-1.00731	1.898037	0.754387
C	-2.92327	0.358744	1.054532
C	-0.22058	3.154478	0.482666
C	-3.77325	-0.43552	0.042646
C	0.86824	3.084571	-0.61625
C	2.122011	2.214914	-0.33457
C	1.833757	0.736181	-0.53054
C	1.851876	-0.19853	0.422778
C	1.528404	-1.67134	0.278215
C	-3.00165	-0.97808	-1.17582
C	-1.78471	-1.84938	-0.90676
C	-0.56152	-1.36911	-1.1896
C	0.777243	-2.04684	-1.0273
C	2.776861	-2.58334	0.511094
C	3.874494	-2.39818	-0.54712
C	3.355897	-2.41383	1.924873
C	-3.30013	2.673024	0.009194
C	3.245693	2.631053	-1.30677
O	2.524796	2.512854	1.008924
C	-2.05667	-3.23714	-0.37693
O	-4.39727	-1.55084	0.695074
H	-0.42578	1.087656	1.185933
H	-3.58881	0.580729	1.907392
H	-2.13424	-0.29867	1.435469
H	-0.8909	3.977061	0.210592
H	0.27651	3.457572	1.413342
H	-4.56097	0.224408	-0.35434
H	0.424583	2.749802	-1.56229
H	1.219778	4.111644	-0.78191
H	1.593622	0.469368	-1.55872
H	2.08591	0.126146	1.4364
H	0.836568	-1.909	1.102134
H	-2.69898	-0.11179	-1.77558
H	-3.72888	-1.54222	-1.77802
H	-0.51708	-0.34983	-1.56656
H	0.663845	-3.13715	-1.0387
H	1.402538	-1.80046	-1.89586

H	2.408828	-3.61756	0.433661
H	4.718274	-3.06912	-0.34618
H	3.513935	-2.61756	-1.55797
H	4.255216	-1.36997	-0.54341
H	4.129922	-3.16618	2.117276
H	3.818125	-1.42827	2.05695
H	2.58069	-2.52706	2.692746
H	-2.84775	3.649786	-0.17717
H	-3.72844	2.325408	-0.94065
H	-4.14714	2.820947	0.693955
H	4.147661	2.031356	-1.13228
H	2.945258	2.483827	-2.35047
H	3.495279	3.687558	-1.16216
H	3.348087	2.024639	1.17491
H	-2.73382	-3.77955	-1.0518
H	-1.14732	-3.83295	-0.26062
H	-2.56877	-3.18716	0.589461
H	-4.87014	-1.20515	1.469283
07_1S4R10S_24			
C	-2.31595	1.677664	0.588594
C	-0.99993	1.902628	0.752171
C	-2.91388	0.366794	1.072509
C	-0.21237	3.157057	0.4738
C	-3.76668	-0.42032	0.066115
C	0.875849	3.079355	-0.62538
C	2.128422	2.208272	-0.34098
C	1.838304	0.729478	-0.53453
C	1.850857	-0.20294	0.421134
C	1.521748	-1.67497	0.280386
C	-3.00005	-0.94941	-1.16894
C	-1.79254	-1.83629	-0.91529
C	-0.56426	-1.36393	-1.1883
C	0.768857	-2.05138	-1.0241
C	2.766135	-2.59163	0.516229
C	3.864557	-2.41498	-0.54261
C	3.345819	-2.41924	1.9294
C	-3.29853	2.682187	0.030787
C	3.253466	2.620834	-1.31318
O	2.530672	2.507895	1.002136
C	-2.08166	-3.22531	-0.39769
O	-4.37546	-1.47462	0.827747
H	-0.41598	1.09099	1.177989
H	-3.57709	0.57326	1.926634
H	-2.11925	-0.29032	1.442298

H	-0.8818	3.978937	0.19734
H	0.285716	3.465095	1.402361
H	-4.56147	0.23914	-0.31725
H	0.430947	2.74115	-1.56969
H	1.229547	4.104841	-0.79629
H	1.600816	0.460936	-1.56294
H	2.082478	0.123735	1.43463
H	0.828821	-1.90715	1.104857
H	-2.6934	-0.07673	-1.75846
H	-3.72966	-1.49545	-1.78994
H	-0.51091	-0.3414	-1.55476
H	0.647667	-3.14091	-1.03085
H	1.396023	-1.8127	-1.89351
H	2.39362	-3.62455	0.442656
H	4.705836	-3.08816	-0.3387
H	3.503482	-2.63729	-1.55267
H	4.249038	-1.38818	-0.54306
H	4.116409	-3.17434	2.124757
H	3.812473	-1.43527	2.057563
H	2.570075	-2.52584	2.69763
H	-2.84634	3.658367	-0.15939
H	-3.73805	2.33664	-0.91481
H	-4.13758	2.830062	0.725046
H	4.154304	2.019849	-1.1372
H	2.953437	2.47252	-2.35686
H	3.504825	3.677115	-1.17011
H	3.352723	2.018222	1.169957
H	-2.75665	-3.75817	-1.08345
H	-1.18034	-3.83219	-0.27791
H	-2.59871	-3.17554	0.566494
H	-4.92165	-1.99612	0.217776
07_1S4R10S_25			
C	-0.87553	-2.79204	-0.17653
C	0.269194	-2.37744	-0.74812
C	-2.17589	-2.08248	-0.50215
C	1.654083	-2.93978	-0.56067
C	-2.7237	-1.20441	0.644768
C	2.58849	-2.16875	0.401012
C	3.099937	-0.77737	-0.04979
C	2.087299	0.340041	0.164088
C	1.690614	1.220783	-0.75961
C	0.784994	2.43021	-0.59061
C	-3.53168	-0.00098	0.136088
C	-2.77764	1.123724	-0.55612

C	-1.43762	1.144593	-0.65921	07_1S4R10S_26			
C	-0.56469	2.173364	-1.33137	C	1.691817	-1.56265	1.137389
C	0.621836	2.936505	0.866734	C	1.271019	-2.24728	0.062015
C	1.956477	3.458627	1.42859	C	2.790738	-0.51618	1.114133
C	-0.44087	4.04178	0.987222	C	0.198424	-3.3057	-0.02073
C	-0.98591	-3.93128	0.808462	C	3.637493	-0.28918	-0.14438
C	4.354401	-0.42804	0.783627	C	-0.9432	-3.00738	-1.02296
O	3.475798	-0.91105	-1.42673	C	-2.12944	-2.1533	-0.50812
C	-3.69039	2.189514	-1.11859	C	-1.69631	-0.79075	-0.00357
O	-3.55793	-2.04982	1.454087	C	-1.99912	0.40735	-0.5153
H	0.206004	-1.53898	-1.44036	C	-1.50248	1.729981	0.025816
H	-2.96482	-2.80947	-0.74308	C	2.940041	0.420257	-1.33673
H	-2.03415	-1.45695	-1.38801	C	2.005709	1.567404	-0.99945
H	1.597343	-3.96886	-0.18773	C	0.692077	1.412263	-1.23771
H	2.150175	-2.99351	-1.53683	C	-0.45064	2.346954	-0.95012
H	-1.88507	-0.84139	1.256637	C	-2.68707	2.701912	0.304315
H	2.103408	-2.05713	1.378937	C	-3.70051	2.081117	1.279846
H	3.473912	-2.79806	0.560042	C	-2.22637	4.067253	0.841563
H	1.768015	0.451489	1.200013	C	1.111137	-1.73458	2.524452
H	2.024326	1.060593	-1.78637	C	-3.20552	-2.08068	-1.60099
H	1.266809	3.247951	-1.15211	O	-2.65647	-2.89852	0.617732
H	-4.0769	0.429212	0.992915	C	2.650496	2.811684	-0.43678
H	-4.31694	-0.37757	-0.53795	O	4.761807	0.476386	0.325447
H	-0.87662	0.312996	-0.24129	H	1.759044	-2.05303	-0.89223
H	-0.31324	1.825863	-2.34591	H	3.505475	-0.76042	1.913409
H	-1.0897	3.123296	-1.46708	H	2.351734	0.447236	1.412218
H	0.288552	2.094234	1.489696	H	0.677566	-4.23791	-0.356
H	1.845973	3.757886	2.477693	H	-0.24089	-3.52718	0.954693
H	2.752347	2.71125	1.371572	H	3.999074	-1.26335	-0.51166
H	2.287433	4.342304	0.866733	H	-0.53791	-2.53021	-1.92435
H	-0.46629	4.432941	2.011195	H	-1.37551	-3.96498	-1.34068
H	-0.21363	4.883639	0.31943	H	-1.07114	-0.83384	0.886106
H	-1.44415	3.677944	0.747719	H	-2.61551	0.477195	-1.41386
H	-0.01874	-4.36115	1.080663	H	-0.99053	1.541051	0.981157
H	-1.48907	-3.59977	1.724453	H	2.385028	-0.33007	-1.90867
H	-1.61152	-4.73576	0.395853	H	3.743321	0.771479	-2.00569
H	4.75323	0.549799	0.486868	H	0.38546	0.46136	-1.66976
H	4.123169	-0.38289	1.853919	H	-0.08723	3.291402	-0.53795
H	5.130219	-1.18547	0.627575	H	-0.96661	2.595662	-1.8919
H	3.926643	-0.08978	-1.68279	H	-3.20219	2.872118	-0.65458
H	-4.38056	2.560386	-0.34847	H	-4.54662	2.758918	1.442994
H	-3.15041	3.046148	-1.52848	H	-4.09449	1.12961	0.909395
H	-4.31686	1.776815	-1.92187	H	-3.23433	1.889853	2.255664
H	-3.81227	-1.54041	2.240564	H	-3.09278	4.685484	1.104555

H	-1.6194	3.946207	1.748701
H	-1.63451	4.630051	0.113388
H	0.26074	-2.4184	2.557404
H	1.873577	-2.11226	3.22002
H	0.779598	-0.76723	2.927103
H	-4.07925	-1.51859	-1.25153
H	-2.83213	-1.59584	-2.50959
H	-3.53136	-3.0927	-1.8602
H	-3.34345	-2.34388	1.022379
H	3.311885	3.268708	-1.18754
H	1.923683	3.568964	-0.13161
H	3.286909	2.569073	0.420996
H	5.320202	0.671633	-0.44407
07_1S4R10S_27			
C	1.706512	-1.54514	1.131674
C	1.284005	-2.22851	0.056199
C	2.806194	-0.49767	1.100643
C	0.220564	-3.29638	-0.02291
C	3.644819	-0.26577	-0.16892
C	-0.9269	-3.01041	-1.02161
C	-2.11902	-2.16774	-0.50214
C	-1.69531	-0.80178	0.000613
C	-2.00745	0.393486	-0.51181
C	-1.51757	1.719674	0.026088
C	2.941293	0.448981	-1.34722
C	1.99124	1.580346	-0.99586
C	0.67977	1.414313	-1.2387
C	-0.47118	2.340093	-0.95324
C	-2.70729	2.685046	0.305485
C	-3.71489	2.059683	1.284218
C	-2.25372	4.053957	0.839621
C	1.135052	-1.7238	2.521595
C	-3.20004	-2.10478	-1.59062
O	-2.63462	-2.9179	0.625833
C	2.619842	2.829328	-0.4252
O	4.781517	0.550278	0.165021
H	1.764775	-2.02688	-0.90008
H	3.516843	-0.75717	1.903383
H	2.375893	0.466051	1.409318
H	0.708573	-4.22362	-0.3592
H	-0.21332	-3.52195	0.954013
H	4.002714	-1.23865	-0.54249
H	-0.5295	-2.52959	-1.92449
H	-1.35067	-3.97248	-1.33717
H	-1.06728	-0.83938	0.888557
H	-2.62639	0.458113	-1.40898
H	-1.00268	1.535275	0.980794
H	2.39646	-0.29944	-1.93022
H	3.751029	0.819795	-1.99176
H	0.382583	0.463091	-1.67675
H	-0.1155	3.289359	-0.54552
H	-0.98989	2.580718	-1.8956
H	-3.22553	2.850655	-0.65251
H	-4.56509	2.732256	1.447765
H	-4.10337	1.104872	0.916459
H	-3.24555	1.873372	2.259512
H	-3.12326	4.666957	1.104543
H	-1.64322	3.937907	1.745053
H	-1.66773	4.619593	0.108955
H	0.286518	-2.40985	2.556631
H	1.901726	-2.10291	3.211987
H	0.803104	-0.75893	2.9298
H	-4.0772	-1.5502	-1.2378
H	-2.83447	-1.61699	-2.5008
H	-3.51801	-3.1197	-1.8483
H	-3.3259	-2.3701	1.032492
H	3.288477	3.289454	-1.16673
H	1.883346	3.580597	-0.12825
H	3.248509	2.592635	0.43993
H	5.243289	0.120543	0.903086
07_1S4R10S_28			
C	-2.19615	1.686106	0.319591
C	-1.35664	1.811072	-0.72319
C	-2.71491	0.31094	0.736092
C	-0.58652	3.010229	-1.20486
C	-3.23117	-0.62243	-0.38082
C	0.939499	2.759398	-1.27136
C	1.62585	2.40817	0.078968
C	1.582937	0.932203	0.468553
C	1.715339	-0.15298	-0.30252
C	1.781316	-1.57195	0.2248
C	-3.11565	-2.10071	-0.00077
C	-1.71891	-2.70652	0.039787
C	-0.63941	-2.07035	-0.44588
C	0.794269	-2.52334	-0.51292
C	3.233184	-2.15343	0.200397
C	3.906095	-2.0865	-1.18035
C	4.121656	-1.48981	1.26231

C	-2.62209	2.820801	1.222394
C	3.093616	2.86563	0.048665
O	1.030506	3.174295	1.147202
C	-1.69301	-4.09111	0.644807
O	-4.62132	-0.38119	-0.64466
H	-1.13346	0.912326	-1.29523
H	-3.53863	0.42094	1.452597
H	-1.90709	-0.19954	1.277237
H	-0.91268	3.27244	-2.22226
H	-0.7739	3.891732	-0.5854
H	-2.67054	-0.45242	-1.30848
H	1.153428	1.969724	-2.0023
H	1.403136	3.673734	-1.66238
H	1.515685	0.793559	1.549617
H	1.805325	-0.04074	-1.3847
H	1.472805	-1.54809	1.27962
H	-3.72896	-2.67426	-0.71161
H	-3.5979	-2.24681	0.97802
H	-0.78256	-1.07698	-0.85915
H	0.920467	-3.53204	-0.10575
H	1.089813	-2.5807	-1.57174
H	3.136882	-3.21574	0.470863
H	4.877614	-2.5939	-1.15394
H	3.30582	-2.56747	-1.96071
H	4.085912	-1.04856	-1.48506
H	5.119615	-1.94429	1.27325
H	4.242951	-0.41856	1.064521
H	3.692748	-1.59814	2.265956
H	-2.19094	3.785879	0.943794
H	-3.7154	2.928434	1.207572
H	-2.35262	2.614944	2.268162
H	3.604549	2.584761	0.975407
H	3.620542	2.403376	-0.79226
H	3.142638	3.955267	-0.05883
H	0.14348	2.805133	1.290334
H	-2.37553	-4.76209	0.104869
H	-0.70081	-4.54772	0.635752
H	-2.04391	-4.06649	1.685686
H	-4.69784	0.544923	-0.92589

07_1S4R10S_29

C	-1.89153	1.800035	0.890343
C	-1.24132	2.235975	-0.20019
C	-2.75984	0.548328	0.89712
C	-0.24409	3.359069	-0.32987
C	-3.28876	-0.00808	-0.43934
C	0.98589	2.983424	-1.18471
C	2.099416	2.148751	-0.50582
C	1.615437	0.830971	0.079396
C	1.862836	-0.40407	-0.37507
C	1.424896	-1.68588	0.297682
C	-3.44259	-1.52967	-0.40816
C	-2.15885	-2.3482	-0.37695
C	-0.94823	-1.81041	-0.60672
C	0.403357	-2.47466	-0.57795
C	2.631381	-2.58693	0.708388
C	3.489401	-3.06334	-0.47552
C	3.504862	-1.90653	1.772526
C	-1.75688	2.426246	2.25885
C	3.257026	1.968963	-1.50067
O	2.55443	2.972874	0.594424
C	-2.40076	-3.81103	-0.08359
O	-4.58646	0.528388	-0.74217
H	-1.41556	1.718415	-1.14292
H	-3.63948	0.712781	1.535904
H	-2.17636	-0.23446	1.403826
H	-0.73039	4.210931	-0.83018
H	0.094275	3.727397	0.642417
H	-2.61263	0.243786	-1.26415
H	0.658689	2.449205	-2.08591
H	1.464718	3.910771	-1.52408
H	1.038693	0.948481	0.996042
H	2.424521	-0.52957	-1.30091
H	0.909008	-1.4183	1.230741
H	-4.03708	-1.82452	-1.28578
H	-4.05852	-1.8015	0.46292
H	-0.88959	-0.74583	-0.81904
H	0.33626	-3.50087	-0.20076
H	0.799358	-2.54856	-1.60233
H	2.188279	-3.48129	1.171737
H	4.260185	-3.76126	-0.12788
H	2.8943	-3.58231	-1.23494
H	4.004112	-2.22718	-0.96391
H	4.295003	-2.58318	2.119338
H	3.985798	-1.00468	1.375813
H	2.910241	-1.60936	2.645113
H	-2.72765	2.809487	2.603234
H	-1.44139	1.67802	2.999645
H	-1.04275	3.252071	2.287569

H	4.084709	1.41747	-1.04021
H	2.943714	1.422672	-2.39715
H	3.627228	2.951113	-1.81046
H	3.207215	2.449682	1.088024
H	-3.1025	-4.23914	-0.81292
H	-1.49007	-4.41334	-0.10817
H	-2.86631	-3.9388	0.903559
H	-4.48968	1.494011	-0.77078

07_1S4R10S_30

C	1.83284	-1.55304	0.975383
C	1.409331	-2.14086	-0.15482
C	2.878383	-0.45462	1.036546
C	0.390045	-3.24223	-0.31695
C	3.697157	-0.07004	-0.20208
C	-0.83623	-2.8868	-1.19255
C	-2.01913	-2.16311	-0.499
C	-1.62395	-0.82127	0.086345
C	-1.98155	0.395836	-0.33925
C	-1.49311	1.689046	0.272945
C	2.952928	0.714294	-1.31625
C	1.975833	1.78388	-0.86436
C	0.664502	1.579978	-1.07411
C	-0.51716	2.42881	-0.69533
C	-2.6459	2.622068	0.751473
C	-3.62036	3.043279	-0.36116
C	-3.41142	1.99496	1.926758
C	1.304661	-1.89971	2.350983
C	-3.19199	-2.06616	-1.48644
O	-2.39841	-3.0352	0.59305
C	2.576025	3.013157	-0.22513
O	4.788839	0.702664	0.329587
H	1.85061	-1.81846	-1.09689
H	3.615279	-0.73605	1.802738
H	2.397951	0.454691	1.426837
H	0.893496	-4.08742	-0.80988
H	0.036274	-3.62226	0.644189
H	4.100478	-0.98662	-0.66259
H	-0.52195	-2.28408	-2.05402
H	-1.24586	-3.82261	-1.59469
H	-0.96955	-0.89909	0.952698
H	-2.62013	0.49423	-1.21732
H	-0.90713	1.43189	1.166719
H	2.423166	-0.00371	-1.95051
H	3.730537	1.160404	-1.95851
H	0.38991	0.646789	-1.56344
H	-0.20911	3.369497	-0.22471
H	-1.0643	2.702898	-1.60973
H	-2.15958	3.536102	1.124541
H	-4.35005	3.763212	0.027844
H	-3.10796	3.518507	-1.20471
H	-4.18388	2.186195	-0.74901
H	-4.18009	2.679885	2.304247
H	-3.90881	1.066164	1.62258
H	-2.73801	1.754196	2.758379
H	0.465858	-2.59816	2.329997
H	2.096528	-2.34015	2.972964
H	0.971293	-0.99258	2.87411
H	-4.05457	-1.57612	-1.02025
H	-2.9242	-1.49738	-2.38364
H	-3.49276	-3.07204	-1.79503
H	-3.09122	-2.57353	1.093261
H	3.188518	3.56195	-0.95565
H	1.82174	3.704029	0.160533
H	3.251415	2.736857	0.591813
H	5.327234	0.99825	-0.42206

07_1S4R10S_31

C	-2.32157	1.670807	0.579266
C	-1.00743	1.897168	0.755223
C	-2.92383	0.358219	1.054539
C	-0.22041	3.153559	0.484134
C	-3.77345	-0.43543	0.041833
C	0.867624	3.084393	-0.61564
C	2.121892	2.214989	-0.33528
C	1.833679	0.736235	-0.5307
C	1.85214	-0.19832	0.422773
C	1.52867	-1.6711	0.278553
C	-3.00125	-0.97786	-1.17631
C	-1.7845	-1.84925	-0.90674
C	-0.56117	-1.3692	-1.18946
C	0.777502	-2.04699	-1.02685
C	2.777194	-2.58309	0.511653
C	3.874671	-2.39831	-0.54676
C	3.356419	-2.41301	1.925289
C	-3.30003	2.673196	0.010529
C	3.244412	2.631246	-1.3088
O	2.526253	2.513251	1.007685
C	-2.05662	-3.23686	-0.37664
O	-4.39828	-1.55078	0.693429

H	-0.42611	1.086364	1.186248
H	-3.58976	0.579901	1.907182
H	-2.13509	-0.29948	1.435553
H	-0.89067	3.976599	0.213295
H	0.277453	3.45563	1.414726
H	-4.56069	0.224944	-0.35539
H	0.423393	2.749944	-1.56152
H	1.218789	4.111638	-0.78109
H	1.59323	0.469189	-1.55875
H	2.086456	0.126496	1.436292
H	0.836843	-1.90862	1.102533
H	-2.69822	-0.11155	-1.77585
H	-3.72824	-1.54189	-1.77891
H	-0.51658	-0.35002	-1.5667
H	0.664051	-3.1373	-1.03787
H	1.402848	-1.80097	-1.89549
H	2.409099	-3.61733	0.434686
H	4.718716	-3.06883	-0.34546
H	3.514125	-2.61851	-1.55745
H	4.255066	-1.36998	-0.54376
H	4.13024	-3.16549	2.118004
H	3.818979	-1.42753	2.056794
H	2.581274	-2.52561	2.693324
H	-2.84759	3.650185	-0.1745
H	-3.72775	2.326632	-0.93998
H	-4.14745	2.820336	0.694955
H	4.146685	2.031748	-1.1352
H	2.942898	2.483892	-2.35219
H	3.493968	3.687803	-1.16453
H	3.34874	2.023625	1.173403
H	-2.73297	-3.77976	-1.05193
H	-1.14721	-3.83238	-0.25922
H	-2.56964	-3.18664	0.589247
H	-4.87232	-1.20512	1.46692

07_1S4R10S_32

C	1.85248	-1.53346	0.971531
C	1.424877	-2.11984	-0.15787
C	2.897558	-0.43202	1.022467
C	0.415441	-3.23071	-0.31615
C	3.702594	-0.0409	-0.22974
C	-0.81605	-2.88941	-1.18962
C	-2.00468	-2.17818	-0.49346
C	-1.62098	-0.83263	0.090606
C	-1.99025	0.380693	-0.33547
C	-1.51154	1.678617	0.273787
C	2.947376	0.746618	-1.3266
C	1.955951	1.796953	-0.85805
C	0.64725	1.584426	-1.0755
C	-0.54194	2.423276	-0.69673
C	-2.6715	2.60349	0.750842
C	-3.64948	3.014932	-0.36235
C	-3.43177	1.972803	1.927629
C	1.337564	-1.88766	2.350063
C	-3.18071	-2.09317	-1.47815
O	-2.37243	-3.05406	0.599599
C	2.540377	3.026784	-0.20516
O	4.803306	0.796635	0.166595
H	1.855945	-1.78952	-1.10183
H	3.634105	-0.72845	1.788166
H	2.426094	0.476233	1.425276
H	0.92698	-4.07103	-0.80912
H	0.067871	-3.61323	0.646278
H	4.101226	-0.95536	-0.69786
H	-0.51017	-2.28386	-2.0521
H	-1.21616	-3.82994	-1.5902
H	-0.9636	-0.90358	0.95537
H	-2.63133	0.472534	-1.21234
H	-0.92322	1.427848	1.167881
H	2.426408	0.031804	-1.97062
H	3.728518	1.211565	-1.94497
H	0.381707	0.653989	-1.57515
H	-0.24161	3.367586	-0.22843
H	-1.09181	2.691093	-1.61131
H	-2.19227	3.522076	1.121914
H	-4.38493	3.729578	0.025587
H	-3.14097	3.492865	-1.2067
H	-4.20616	2.15275	-0.74881
H	-4.20615	2.652149	2.303537
H	-3.92141	1.039126	1.625798
H	-2.75634	1.739676	2.759825
H	2.135634	-2.32752	2.96479
H	1.003642	-0.9842	2.879151
H	0.501892	-2.59001	2.332738
H	-4.04717	-1.61208	-1.00987
H	-2.92087	-1.52153	-2.37582
H	-3.47182	-3.10205	-1.78625
H	-3.06993	-2.59999	1.100244
H	3.1549	3.584621	-0.92629

H	1.776811	3.707638	0.180323
H	3.213234	2.751502	0.614135
H	5.298495	0.323395	0.854672

07_1R4R10S_3

C	2.731647	1.243619	0.584713
C	1.454348	1.657295	0.671479
C	3.118204	-0.10108	1.181554
C	0.856381	2.97158	0.232866
C	3.646214	-1.20169	0.231877
C	-0.29984	2.8818	-0.79338
C	-1.71632	2.494631	-0.29083
C	-1.75029	1.1041	0.316735
C	-2.12146	-0.01033	-0.32103
C	-2.06117	-1.40122	0.26284
C	2.683733	-1.59415	-0.89924
C	1.403503	-2.29397	-0.47679
C	0.21728	-1.78574	-0.85126
C	-1.16958	-2.32513	-0.61945
C	-3.46726	-2.0322	0.508769
C	-4.19334	-1.32802	1.664446
C	-4.36222	-2.07259	-0.74078
C	3.862377	2.059548	0.002252
C	-2.26366	3.525104	0.712967
O	-2.5098	2.546262	-1.4915
C	1.578448	-3.58392	0.291957
O	4.872698	-0.83543	-0.41742
H	0.753476	0.968289	1.137469
H	3.898269	0.060503	1.944918
H	2.257177	-0.52246	1.71459
H	0.504124	3.517456	1.121266
H	1.62966	3.607118	-0.20946
H	3.835068	-2.0874	0.858875
H	-0.41124	3.864526	-1.26994
H	-0.03063	2.174868	-1.58683
H	-1.42093	1.037144	1.353702
H	-2.44255	0.070409	-1.35998
H	-1.57281	-1.33255	1.245475
H	3.254179	-2.26224	-1.562
H	2.445175	-0.69657	-1.48056
H	0.241045	-0.85016	-1.40884
H	-1.65615	-2.44845	-1.59851
H	-1.14605	-3.31949	-0.16014
H	-3.28417	-3.07232	0.818496
H	-5.1596	-1.80463	1.868587
H	-3.60125	-1.36123	2.587168
H	-4.38138	-0.2741	1.427154
H	-5.29291	-2.60971	-0.52344
H	-4.63638	-1.06261	-1.06865
H	-3.87983	-2.57982	-1.58343
H	3.537973	3.03024	-0.37959
H	4.63174	2.244143	0.766569
H	4.358164	1.513123	-0.80634
H	-3.30421	3.289315	0.967814
H	-1.69144	3.532807	1.646983
H	-2.23428	4.529352	0.277023
H	-3.4043	2.253651	-1.2486
H	2.01398	-3.40684	1.284462
H	0.638118	-4.12016	0.439826
H	2.270248	-4.25662	-0.23359
H	5.496526	-0.55812	0.272681

07_1R4R10S_4

C	-2.79535	0.829567	-0.87462
C	-1.6106	1.409956	-0.62602
C	-2.80201	-0.56306	-1.47589
C	-1.326	2.750371	0.009375
C	-3.16244	-1.72454	-0.51349
C	0.068059	3.325551	-0.31541
C	1.299975	2.607009	0.314181
C	1.590776	1.26893	-0.35052
C	1.908834	0.134463	0.280585
C	2.204548	-1.19373	-0.37957
C	-2.54693	-1.65349	0.888151
C	-1.05485	-1.8381	1.101574
C	-0.16755	-2.08568	0.124072
C	1.319473	-2.31302	0.246995
C	3.725457	-1.54003	-0.28229
C	4.063945	-2.92841	-0.85285
C	4.590135	-0.47845	-0.98107
C	-4.1371	1.452844	-0.57863
C	2.530647	3.518079	0.166201
O	1.118632	2.45876	1.730798
C	-0.68607	-1.7197	2.564895
O	-4.57912	-1.77464	-0.26979
H	-0.72788	0.830697	-0.87919
H	-3.5126	-0.61261	-2.31722
H	-1.81621	-0.76807	-1.90523
H	-2.07624	3.489675	-0.29869
H	-1.42576	2.687252	1.104441

H	-2.85028	-2.66306	-0.99973
H	0.205091	3.364944	-1.40464
H	0.092757	4.362513	0.041357
H	1.546656	1.278773	-1.4408
H	1.973387	0.144228	1.370056
H	1.953101	-1.11577	-1.44881
H	-3.06937	-2.41347	1.488251
H	-2.83971	-0.69188	1.332765
H	-0.53536	-2.15107	-0.89917
H	1.618608	-2.44473	1.293215
H	1.553433	-3.25771	-0.2585
H	3.990745	-1.54075	0.786618
H	5.149378	-3.08304	-0.85365
H	3.619937	-3.74446	-0.27498
H	3.717114	-3.02176	-1.89084
H	5.655099	-0.71109	-0.86404
H	4.374171	-0.44793	-2.05767
H	4.414033	0.522477	-0.57754
H	-4.73321	1.54274	-1.49839
H	-4.7178	0.817896	0.100948
H	-4.05115	2.448465	-0.1343
H	3.405694	3.041973	0.618955
H	2.746587	3.712308	-0.88962
H	2.352915	4.47658	0.666521
H	0.441692	1.774957	1.863066
H	-1.24007	-2.45425	3.165614
H	0.378255	-1.863	2.759361
H	-0.96733	-0.731	2.953902
H	-5.01952	-1.80422	-1.13416

07_1R4R10S_5

C	2.764119	1.165577	0.663952
C	1.498178	1.592698	0.822191
C	3.151211	-0.20873	1.188039
C	0.901703	2.931694	0.462177
C	3.617369	-1.27972	0.174129
C	-0.14484	2.924128	-0.6846
C	-1.6168	2.524438	-0.37072
C	-1.69661	1.141373	0.244867
C	-2.18816	0.042311	-0.33294
C	-2.12162	-1.34106	0.270016
C	2.607705	-1.61026	-0.93559
C	1.32244	-2.29376	-0.50021
C	0.139718	-1.76713	-0.86052
C	-1.25384	-2.28424	-0.61761
C	-3.52171	-1.95934	0.567479
C	-4.22486	-1.20589	1.706485
C	-4.44147	-2.05163	-0.66138
C	3.880749	1.988326	0.064834
C	-2.29607	3.551106	0.545573
O	-2.35118	2.594861	-1.60487
C	1.48646	-3.59104	0.258372
O	4.82497	-0.90942	-0.50756
H	0.808772	0.890573	1.28548
H	3.967033	-0.09059	1.921307
H	2.307043	-0.63526	1.743571
H	0.444374	3.370953	1.361007
H	1.694575	3.62696	0.169631
H	3.811845	-2.19377	0.757118
H	-0.19495	3.932138	-1.11598
H	0.219799	2.258879	-1.48023
H	-1.27475	1.061533	1.24591
H	-2.62919	0.120289	-1.3269
H	-1.60456	-1.25862	1.236796
H	3.141173	-2.26938	-1.63724
H	2.374738	-0.6887	-1.48073
H	0.17188	-0.82824	-1.41185
H	-1.75036	-2.40405	-1.59214
H	-1.24215	-3.27693	-0.15401
H	-3.33304	-2.986	0.916707
H	-5.18365	-1.67707	1.953833
H	-3.61177	-1.19483	2.616033
H	-4.42375	-0.16485	1.425891
H	-5.37077	-2.57151	-0.40014
H	-4.71703	-1.05682	-1.03136
H	-3.97919	-2.60284	-1.48768
H	3.554365	2.977863	-0.26387
H	4.687439	2.130058	0.799069
H	4.329911	1.467899	-0.78701
H	-3.3571	3.304494	0.652631
H	-1.84429	3.556745	1.542311
H	-2.21267	4.555865	0.117587
H	-1.8785	2.047834	-2.25371
H	1.927792	-3.42605	1.250434
H	0.540589	-4.11745	0.406064
H	2.168381	-4.26776	-0.27487
H	5.485587	-0.68802	0.168411

07_1R4R10S_6

C	2.731607	1.243716	0.584692

C	1.454296	1.657357	0.671533
C	3.118195	-0.10093	1.181613
C	0.856243	2.971606	0.232925
C	3.646266	-1.20156	0.231996
C	-0.29996	2.881855	-0.79333
C	-1.71648	2.494627	-0.29084
C	-1.75041	1.104113	0.3168
C	-2.12155	-0.01034	-0.32094
C	-2.06117	-1.40124	0.26291
C	2.683856	-1.59399	-0.8992
C	1.403677	-2.29394	-0.47684
C	0.21742	-1.78559	-0.85106
C	-1.16944	-2.32504	-0.61936
C	-3.4672	-2.03237	0.508751
C	-4.19342	-1.32827	1.664388
C	-4.36209	-2.07287	-0.74085
C	3.862315	2.059533	0.002004
C	-2.26392	3.52511	0.712894
O	-2.50986	2.546155	-1.49156
C	1.578681	-3.58407	0.291584
O	4.87282	-0.83531	-0.4172
H	0.7535	0.968363	1.137645
H	3.898249	0.060693	1.94498
H	2.257174	-0.52233	1.714653
H	0.503958	3.517445	1.121344
H	1.62949	3.607194	-0.20938
H	3.835056	-2.08727	0.859019
H	-0.41137	3.864605	-1.26982
H	-0.03076	2.174952	-1.5868
H	-1.42104	1.037224	1.353768
H	-2.44262	0.070394	-1.35989
H	-1.57288	-1.33254	1.245586
H	3.254385	-2.26196	-1.562
H	2.445245	-0.69636	-1.48042
H	0.241143	-0.84987	-1.4084
H	-1.65591	-2.4483	-1.59847
H	-1.14591	-3.31943	-0.16012
H	-3.28401	-3.07247	0.818494
H	-5.15963	-1.80499	1.868496
H	-3.60135	-1.36139	2.587129
H	-4.38157	-0.27436	1.427081
H	-5.29275	-2.61005	-0.52352
H	-4.63631	-1.06293	-1.06878
H	-3.8796	-2.58012	-1.58344
H	3.537987	3.030342	-0.37959
H	4.631956	2.243837	0.766103
H	4.357728	1.513109	-0.80682
H	-3.30446	3.28928	0.967734
H	-1.69171	3.532852	1.646917
H	-2.23457	4.529347	0.276923
H	-3.40445	2.253791	-1.24865
H	2.01452	-3.40725	1.284001
H	0.63834	-4.12024	0.439599
H	2.270279	-4.25674	-0.23428
H	5.496547	-0.5579	0.272949

07_1R4R10S_7

C	2.719593	1.264136	0.569755
C	1.442863	1.674802	0.680105
C	3.124215	-0.06709	1.187418
C	0.832549	2.984159	0.242953
C	3.658177	-1.17583	0.260806
C	-0.32185	2.888878	-0.78483
C	-1.73495	2.487251	-0.28518
C	-1.75614	1.094372	0.317055
C	-2.11908	-0.02055	-0.3244
C	-2.04875	-1.41255	0.255255
C	2.703299	-1.58322	-0.87987
C	1.420143	-2.28592	-0.46924
C	0.235323	-1.78219	-0.85403
C	-1.15063	-2.32716	-0.62977
C	-3.45044	-2.0544	0.498521
C	-4.18222	-1.35847	1.655587
C	-4.34423	-2.09788	-0.75178
C	3.832343	2.078252	-0.04804
C	-2.29261	3.508731	0.722071
O	-2.52859	2.535916	-1.48607
C	1.593353	-3.57224	0.306054
O	4.914608	-0.73747	-0.27432
H	0.754408	0.989621	1.170104
H	3.911067	0.11356	1.935104
H	2.267749	-0.48726	1.727474
H	0.475088	3.524661	1.132478
H	1.600595	3.62758	-0.19689
H	3.838957	-2.05373	0.900849
H	-0.44131	3.872594	-1.25751
H	-0.04582	2.187406	-1.58079
H	-1.42524	1.026236	1.353401
H	-2.44174	0.061102	-1.3628

H	-1.56111	-1.34356	1.238185
H	3.271643	-2.26478	-1.53602
H	2.471355	-0.69271	-1.47446
H	0.259687	-0.84791	-1.4138
H	-1.63311	-2.44589	-1.61138
H	-1.12486	-3.3244	-0.17694
H	-3.26014	-3.094	0.805637
H	-5.14545	-1.84209	1.857534
H	-3.59066	-1.3904	2.578676
H	-4.37703	-0.30514	1.421177
H	-5.2711	-2.64242	-0.53661
H	-4.62557	-1.08904	-1.07707
H	-3.85756	-2.59923	-1.59552
H	3.49506	3.048553	-0.42004
H	4.626816	2.257716	0.689881
H	4.304204	1.531019	-0.86983
H	-3.33081	3.261809	0.975927
H	-1.72058	3.519012	1.656154
H	-2.27303	4.514652	0.289451
H	-3.42065	2.23516	-1.24412
H	2.025267	-3.38997	1.299097
H	0.65297	-4.10841	0.45333
H	2.286883	-4.24799	-0.2137
H	5.249966	-1.4479	-0.84451
07_1R4R10S_8			
C	-2.78546	0.847447	-0.86673
C	-1.59588	1.421914	-0.62775
C	-2.80691	-0.53766	-1.48687
C	-1.3011	2.759356	0.010282
C	-3.17152	-1.7055	-0.54299
C	0.094985	3.327748	-0.31727
C	1.324472	2.603721	0.310347
C	1.603649	1.261585	-0.35066
C	1.913444	0.12672	0.283708
C	2.195217	-1.20642	-0.37216
C	-2.56964	-1.6446	0.87075
C	-1.07781	-1.82545	1.100349
C	-0.18558	-2.07672	0.128362
C	1.299167	-2.31316	0.260313
C	3.71258	-1.56803	-0.27554
C	4.036342	-2.96124	-0.84282
C	4.586463	-0.51694	-0.97879
C	-4.11994	1.477978	-0.55355
C	2.56045	3.506251	0.155205
O	1.146794	2.461029	1.728123
C	-0.71924	-1.70491	2.565785
O	-4.60587	-1.70167	-0.43923
H	-0.71762	0.841945	-0.89435
H	-3.53225	-0.5779	-2.31152
H	-1.82491	-0.7443	-1.92247
H	-1.39888	2.695564	1.105568
H	-2.04759	3.503697	-0.29474
H	-2.85224	-2.63892	-1.03389
H	0.230058	3.365648	-1.4068
H	0.125691	4.364857	0.03868
H	1.557259	1.268095	-1.44082
H	1.980581	0.139683	1.373013
H	1.943389	-1.13029	-1.4414
H	-3.08105	-2.41848	1.469078
H	-2.87678	-0.6914	1.323238
H	-0.54758	-2.14068	-0.89698
H	1.592722	-2.43805	1.308951
H	1.527131	-3.26422	-0.23603
H	3.979439	-1.5683	0.792997
H	5.120273	-3.12596	-0.8466
H	3.58686	-3.77147	-0.26098
H	3.685276	-3.05458	-1.87937
H	5.64932	-0.75943	-0.86269
H	4.369065	-0.48758	-2.05511
H	4.420516	0.48684	-0.57807
H	-4.7228	1.5793	-1.46719
H	-4.70159	0.840572	0.122651
H	-4.02171	2.468291	-0.09986
H	3.434084	3.02618	0.606533
H	2.773961	3.695458	-0.90202
H	2.390576	4.4676	0.652769
H	0.462728	1.785048	1.864039
H	-1.28106	-2.43425	3.165875
H	0.342904	-1.85388	2.76816
H	-0.99694	-0.71305	2.949275
H	-4.85844	-2.42337	0.159057
07_1R4R10S_9			
C	2.746101	1.18935	0.640665
C	1.489228	1.622162	0.849695
C	3.160139	-0.16585	1.196056
C	0.879006	2.953987	0.484121
C	3.629997	-1.25488	0.213087
C	-0.16231	2.937555	-0.66713

C	-1.62958	2.517248	-0.36101	H	-2.252	4.537564	0.140812
C	-1.69446	1.129219	0.244629	H	-1.88097	2.049148	-2.24736
C	-2.1833	0.031252	-0.33734	H	1.932887	-3.40726	1.277772
C	-2.11015	-1.3542	0.259623	H	0.553273	-4.10704	0.428082
C	2.629292	-1.60436	-0.9075	H	2.185928	-4.26101	-0.239
C	1.339513	-2.28864	-0.4852	H	5.177568	-1.52628	-0.95419
C	0.159365	-1.76704	-0.86077	07_1R4R10S_10			
C	-1.23431	-2.28871	-0.62887	C	2.719391	1.264254	0.569826
C	-3.50777	-1.98184	0.549352	C	1.442687	1.675021	0.680187
C	-4.22088	-1.23527	1.686696	C	3.123866	-0.06699	1.18752
C	-4.42138	-2.07751	-0.68385	C	0.832411	2.984356	0.242942
C	3.827826	1.997427	-0.03683	C	3.658076	-1.17567	0.260961
C	-2.32459	3.528552	0.560732	C	-0.32199	2.888977	-0.78484
O	-2.36154	2.58718	-1.5968	C	-1.73508	2.48721	-0.28522
C	1.499463	-3.58039	0.283714	C	-1.75614	1.094345	0.317188
O	4.876164	-0.826	-0.35324	C	-2.11903	-0.02062	-0.32425
H	0.823455	0.935123	1.36779	C	-2.04863	-1.41265	0.255357
H	3.98768	-0.01943	1.906547	C	2.703433	-1.58297	-0.87996
H	2.32596	-0.58744	1.769015	C	1.420387	-2.2859	-0.4695
H	0.414685	3.389925	1.38059	C	0.235501	-1.78205	-0.85391
H	1.666408	3.656312	0.193495	C	-1.15041	-2.32711	-0.62969
H	3.812974	-2.15771	0.81667	C	-3.45027	-2.05465	0.498465
H	-0.2241	3.946794	-1.0943	C	-4.18208	-1.3591	1.655741
H	0.214676	2.280582	-1.46389	C	-4.34411	-2.09784	-0.75183
H	-1.26687	1.045455	1.242811	C	3.832132	2.07825	-0.04812
H	-2.62926	0.11273	-1.32878	C	-2.29287	3.508705	0.721938
H	-1.59702	-1.27294	1.228581	O	-2.52856	2.535658	-1.48618
H	3.161992	-2.27999	-1.59897	C	1.593757	-3.57248	0.305354
H	2.404054	-0.69181	-1.47065	O	4.914619	-0.73726	-0.27382
H	0.194237	-0.8301	-1.41533	H	0.75418	0.989927	1.170221
H	-1.7242	-2.40349	-1.6073	H	3.910537	0.113631	1.935409
H	-1.22235	-3.28444	-0.17188	H	2.267262	-0.4872	1.727323
H	-3.31426	-3.00803	0.897337	H	0.474949	3.524966	1.132405
H	-5.17728	-1.7137	1.929372	H	1.600452	3.627735	-0.19699
H	-3.61159	-1.22137	2.598734	H	3.838703	-2.05363	0.900966
H	-4.42605	-0.19521	1.407066	H	-0.44155	3.872674	-1.25753
H	-5.34831	-2.60421	-0.42792	H	-0.04591	2.187498	-1.58077
H	-4.70214	-1.08382	-1.05288	H	-1.42528	1.02631	1.353544
H	-3.95179	-2.62379	-1.50932	H	-2.44168	0.061088	-1.36264
H	3.482787	2.982258	-0.36119	H	-1.5611	-1.34368	1.238343
H	4.678885	2.142392	0.643032	H	3.271949	-2.26429	-1.5362
H	4.225751	1.46271	-0.90476	H	2.471406	-0.69234	-1.47436
H	-3.38287	3.268116	0.662051	H	0.259795	-0.84759	-1.41338
H	-1.87638	3.532123	1.559061	H	-1.63289	-2.44578	-1.61131

H	-1.12458	-3.32437	-0.17692
H	-3.25991	-3.09434	0.805287
H	-5.14528	-1.84284	1.857567
H	-3.59049	-1.39125	2.57881
H	-4.37695	-0.30572	1.421633
H	-5.27093	-2.6425	-0.53674
H	-4.62554	-1.08893	-1.07681
H	-3.85744	-2.59894	-1.59571
H	3.495057	3.048978	-0.41918
H	4.627232	2.256701	0.689356
H	4.303193	1.531397	-0.87065
H	-3.33101	3.261593	0.975909
H	-1.72077	3.519143	1.655973
H	-2.27353	4.514601	0.289245
H	-3.42099	2.236182	-1.24403
H	2.024604	-3.39037	1.2989
H	0.653608	-4.10937	0.45148
H	2.288254	-4.24751	-0.21403
H	5.250048	-1.44759	-0.84411
07_1R4R10S_11			
C	2.746392	1.189096	0.640593
C	1.489567	1.621985	0.84978
C	3.160429	-0.16609	1.195996
C	0.879358	2.953833	0.484269
C	3.629898	-1.25527	0.213002
C	-0.16193	2.937493	-0.66703
C	-1.62923	2.517372	-0.36089
C	-1.69428	1.129299	0.244631
C	-2.18341	0.031469	-0.33737
C	-2.11036	-1.35406	0.259448
C	2.629006	-1.60459	-0.90744
C	1.339103	-2.28858	-0.48504
C	0.159072	-1.76706	-0.86109
C	-1.23468	-2.28855	-0.62922
C	-3.50801	-1.9816	0.549205
C	-4.22093	-1.23511	1.686727
C	-4.42177	-2.077	-0.68391
C	3.828079	1.997068	-0.0371
C	-2.32408	3.528697	0.56094
O	-2.36123	2.58749	-1.59666
C	1.498812	-3.58003	0.284424
O	4.876088	-0.82666	-0.35351
H	0.82383	0.934986	1.367974
H	3.988187	-0.01971	1.906244
H	2.32636	-0.58752	1.76924
H	0.415	3.389692	1.380755
H	1.666785	3.656172	0.193749
H	3.812791	-2.15811	0.816606
H	-0.22359	3.946732	-1.09422
H	0.214999	2.280468	-1.46377
H	-1.26657	1.045342	1.242743
H	-2.62954	0.113111	-1.32872
H	-1.59716	-1.27297	1.228377
H	3.161495	-2.28038	-1.59893
H	2.403914	-0.69202	-1.47062
H	0.194131	-0.83037	-1.41606
H	-1.72462	-2.40311	-1.60765
H	-1.22283	-3.28435	-0.17238
H	-3.31458	-3.00786	0.897027
H	-5.17737	-1.71344	1.929427
H	-3.61156	-1.22142	2.598714
H	-4.42599	-0.19498	1.407261
H	-5.34871	-2.60365	-0.42795
H	-4.70247	-1.08322	-1.05277
H	-3.95232	-2.6232	-1.50952
H	3.483089	2.981939	-0.36138
H	4.679282	2.141936	0.642606
H	4.225794	1.462318	-0.90511
H	-3.38241	3.268426	0.662166
H	-1.87593	3.532073	1.559298
H	-2.2513	4.537749	0.141148
H	-1.88082	2.049332	-2.24723
H	1.932052	-3.40652	1.278495
H	0.552557	-4.10655	0.428829
H	2.185331	-4.26092	-0.23788
H	5.177115	-1.52689	-0.95471
07_1R4R10S_12			
C	-2.7746	1.27298	-0.07325
C	-1.47872	1.449068	-0.38162
C	-3.17976	0.051917	0.737377
C	-0.81502	2.496559	-1.23644
C	-3.64339	-1.15406	-0.11153
C	0.253851	3.36232	-0.52252
C	1.288426	2.624175	0.374424
C	1.806536	1.358374	-0.29483
C	1.900617	0.160043	0.289018
C	2.377671	-1.11746	-0.36293
C	-2.53138	-1.84776	-0.92694

C	-1.25914	-2.27703	-0.209
C	-0.0652	-1.85891	-0.66856
C	1.313183	-2.23473	-0.1748
C	3.772245	-1.5482	0.189971
C	4.248992	-2.89829	-0.37291
C	4.839877	-0.47592	-0.07946
C	-3.90055	2.184305	-0.49581
C	2.462147	3.568947	0.677988
O	0.707895	2.341081	1.658955
C	-1.41991	-3.24863	0.93701
O	-4.25225	-2.14891	0.722534
H	-0.79804	0.675699	-0.03304
H	-2.35301	-0.27526	1.37749
H	-4.0109	0.322001	1.4064
H	-0.32354	1.973716	-2.07046
H	-1.54914	3.170227	-1.69172
H	-4.38885	-0.8008	-0.84314
H	0.793805	3.925387	-1.29503
H	-0.23659	4.10261	0.121698
H	2.115841	1.477765	-1.33519
H	1.593761	0.073318	1.332696
H	2.493637	-0.93602	-1.44323
H	-2.26528	-1.17919	-1.75249
H	-3.00466	-2.73274	-1.37858
H	-0.06753	-1.19436	-1.53392
H	1.287611	-2.5175	0.88546
H	1.6502	-3.12847	-0.71767
H	3.670053	-1.65548	1.281381
H	5.266858	-3.11458	-0.02753
H	3.614761	-3.73375	-0.06166
H	4.270203	-2.88071	-1.47085
H	5.800827	-0.76893	0.359552
H	4.995279	-0.34414	-1.15883
H	4.55689	0.494848	0.337246
H	-4.67574	1.62755	-1.03967
H	-4.39392	2.621538	0.383429
H	-3.56783	3.004519	-1.13656
H	3.125562	3.107454	1.416246
H	3.041313	3.778433	-0.22727
H	2.092512	4.517385	1.084348
H	-0.14504	1.903048	1.498321
H	-1.88653	-4.17836	0.581993
H	-0.46692	-3.5114	1.402639
H	-2.09273	-2.85246	1.703886
H	-4.96332	-1.71497	1.220931

07_1R4R10S_13

C	-2.75315	1.020101	-0.74868
C	-1.52703	1.455308	-0.41683
C	-2.87096	-0.32683	-1.43981
C	-1.11631	2.720067	0.295384
C	-3.31734	-1.52075	-0.55745
C	0.269883	3.260498	-0.12078
C	1.521233	2.450343	0.336532
C	1.654247	1.131426	-0.40959
C	1.924783	-0.05904	0.135951
C	2.020652	-1.36279	-0.62434
C	-2.66856	-1.61263	0.827406
C	-1.1841	-1.89278	0.983115
C	-0.34811	-2.17298	-0.03045
C	1.132071	-2.46133	0.031285
C	3.490794	-1.85985	-0.80272
C	4.281616	-0.94108	-1.745
C	4.248377	-2.05209	0.521362
C	-4.04082	1.755522	-0.47144
C	2.778746	3.299579	0.085471
O	1.488729	2.248412	1.757359
C	-0.75893	-1.82836	2.43441
O	-4.72719	-1.46262	-0.27877
H	-0.70276	0.793264	-0.66243
H	-3.58597	-0.26274	-2.27596
H	-1.90649	-0.58028	-1.89077
H	-1.85332	3.515454	0.131289
H	-1.11034	2.560848	1.385903
H	-3.10284	-2.44277	-1.12211
H	0.305369	3.373682	-1.21269
H	0.37836	4.266512	0.303098
H	1.510124	1.201243	-1.48909
H	2.080028	-0.11445	1.213726
H	1.630753	-1.19051	-1.63866
H	-3.22084	-2.39139	1.374666
H	-2.89494	-0.67802	1.35969
H	-0.75587	-2.19698	-1.04058
H	1.459735	-2.62206	1.062937
H	1.327274	-3.404	-0.50167
H	3.417767	-2.84501	-1.28794
H	5.290575	-1.33494	-1.91647
H	3.787519	-0.84853	-2.72005
H	4.380834	0.066036	-1.32378

H	5.241055	-2.47732	0.332057
H	4.394587	-1.09719	1.040364
H	3.725824	-2.7306	1.204673
H	-3.88317	2.706501	0.044788
H	-4.57854	1.962682	-1.40808
H	-4.71128	1.137413	0.137552
H	3.668272	2.75931	0.423564
H	2.890738	3.52273	-0.9807
H	2.712119	4.245846	0.633798
H	0.778909	1.611874	1.941843
H	-1.36743	-2.50861	3.046048
H	0.290084	-2.08374	2.594702
H	-0.92462	-0.82035	2.839767
H	-5.1864	-1.39426	-1.1311

07_1R4R10S_14

C	2.796657	1.103157	0.730159
C	1.535414	1.462926	0.435866
C	3.097612	-0.32419	1.166384
C	1.01791	2.830727	0.053487
C	3.607121	-1.2966	0.074344
C	-0.17686	2.827564	-0.9263
C	-1.59509	2.532178	-0.3654
C	-1.68238	1.16153	0.282397
C	-2.13699	0.051706	-0.30844
C	-2.11674	-1.32518	0.312349
C	2.566795	-1.66256	-1.00471
C	1.292471	-2.33967	-0.52948
C	0.101697	-1.81761	-0.87052
C	-1.28803	-2.31123	-0.5662
C	-3.53823	-1.89082	0.61839
C	-4.20043	-1.12309	1.772097
C	-4.47134	-1.93275	-0.60287
C	3.973564	2.053215	0.744916
C	-2.04558	3.617259	0.629236
O	-2.42673	2.597776	-1.53859
C	1.478745	-3.6145	0.259802
O	4.804404	-0.82434	-0.56034
H	0.78079	0.683745	0.500794
H	3.860979	-0.3004	1.955842
H	2.199219	-0.77232	1.607752
H	0.7397	3.388037	0.961457
H	1.819901	3.420984	-0.40502
H	3.924433	-2.21252	0.585664
H	-0.24163	3.815006	-1.40079
H	0.009793	2.10509	-1.73007
H	-1.30707	1.10278	1.304366
H	-2.49744	0.12191	-1.33511
H	-1.59607	-1.24889	1.277686
H	3.088568	-2.32567	-1.71114
H	2.30709	-0.74911	-1.55576
H	0.122202	-0.89781	-1.45427
H	-1.81634	-2.45939	-1.51974
H	-1.2729	-3.28637	-0.067
H	-3.38718	-2.92765	0.955121
H	-5.1782	-1.55393	2.018357
H	-3.58265	-1.15257	2.677886
H	-4.35375	-0.06996	1.507814
H	-5.41827	-2.41873	-0.3399
H	-4.70971	-0.92327	-0.95902
H	-4.03816	-2.49066	-1.44025
H	3.775064	2.990363	0.217254
H	4.24362	2.309743	1.779879
H	4.861378	1.585316	0.306155
H	-3.08337	3.441957	0.938507
H	-1.43056	3.623552	1.535702
H	-1.98711	4.605473	0.160879
H	-3.32831	2.369388	-1.25629
H	1.964277	-3.42197	1.225966
H	0.536429	-4.12866	0.463247
H	2.132811	-4.31188	-0.28162
H	4.543155	-0.11159	-1.16594

07_1R4R10S_15

C	-2.4968	1.64898	-0.10077
C	-1.16387	1.800152	-0.04371
C	-3.16658	0.622653	0.792109
C	-0.28842	2.672139	-0.90628
C	-3.89837	-0.5039	0.045449
C	0.956917	3.268739	-0.22003
C	1.960767	2.287654	0.446724
C	2.115747	1.002852	-0.34988
C	1.838975	-0.22481	0.100923
C	1.976554	-1.51164	-0.68003
C	-3.00777	-1.37811	-0.87085
C	-1.77929	-2.04747	-0.27656
C	-0.56743	-1.80779	-0.80828
C	0.755749	-2.43487	-0.43476
C	3.317418	-2.26131	-0.39401
C	4.513649	-1.52902	-1.01819

C	3.572353	-2.52577	1.09892
C	-3.41161	2.437261	-1.01052
C	3.330466	2.98365	0.581731
O	1.444407	2.031196	1.764164
C	-2.02795	-3.0418	0.834508
O	-4.56806	-1.26551	1.061172
H	-0.62452	1.177635	0.663362
H	-2.42634	0.16926	1.46068
H	-3.92028	1.109534	1.428916
H	0.05528	2.072221	-1.7634
H	-0.85996	3.500811	-1.34168
H	-4.65959	-0.05169	-0.61078
H	1.503218	3.84404	-0.97802
H	0.65849	3.980615	0.559185
H	2.483001	1.132762	-1.36937
H	1.456605	-0.32722	1.116287
H	1.986862	-1.26016	-1.75122
H	-2.70097	-0.75462	-1.71923
H	-3.66647	-2.15689	-1.29307
H	-0.51567	-1.09839	-1.63563
H	0.747619	-2.75438	0.61353
H	0.909323	-3.35115	-1.02728
H	3.231022	-3.23756	-0.89488
H	5.442069	-2.08965	-0.85614
H	4.383699	-1.4009	-2.09982
H	4.641672	-0.53388	-0.57614
H	4.483364	-3.12144	1.229767
H	3.710697	-1.58704	1.648993
H	2.750391	-3.07428	1.57208
H	-3.78718	1.826874	-1.84389
H	-4.29518	2.789214	-0.46101
H	-2.91877	3.3099	-1.44751
H	4.025285	2.353743	1.150848
H	3.780191	3.176563	-0.39865
H	3.217855	3.938653	1.107132
H	2.092711	1.469803	2.221076
H	-2.64537	-3.87502	0.466634
H	-1.10541	-3.47069	1.233415
H	-2.59144	-2.58523	1.654037
H	-5.03612	-1.99254	0.620073

07_1R4R10S_16

C	-2.75051	1.033686	-0.74164
C	-1.51821	1.46699	-0.43045
C	-2.88406	-0.30336	-1.4508
C	-1.09827	2.726697	0.286716
C	-3.32565	-1.50356	-0.58397
C	0.290684	3.261559	-0.12624
C	1.537442	2.446826	0.334689
C	1.665633	1.12691	-0.40993
C	1.927261	-0.06451	0.137598
C	2.017254	-1.3692	-0.62142
C	-2.68289	-1.60192	0.80908
C	-1.19863	-1.88441	0.976309
C	-0.3594	-2.16131	-0.03536
C	1.118287	-2.4603	0.031822
C	3.484373	-1.87714	-0.79339
C	4.28547	-0.96493	-1.73334
C	4.235247	-2.07351	0.533938
C	-4.03033	1.770614	-0.43326
C	2.799321	3.290157	0.085997
O	1.500666	2.245893	1.75566
C	-0.78143	-1.82902	2.430074
O	-4.75049	-1.38602	-0.4307
H	-0.69747	0.80961	-0.69974
H	-3.61779	-0.23129	-2.26558
H	-1.92552	-0.55503	-1.91391
H	-1.83029	3.527113	0.124437
H	-1.09461	2.564998	1.376942
H	-3.10324	-2.42002	-1.15412
H	0.329385	3.374124	-1.21811
H	0.402209	4.267366	0.297374
H	1.525863	1.196674	-1.48997
H	2.078654	-0.12007	1.215918
H	1.632466	-1.19511	-1.63731
H	-3.22503	-2.39131	1.358119
H	-2.91766	-0.67258	1.346586
H	-0.76241	-2.17538	-1.0475
H	1.440982	-2.62309	1.064722
H	1.307557	-3.40479	-0.5001
H	3.406258	-2.86221	-1.27801
H	5.292316	-1.36607	-1.90021
H	3.796044	-0.86998	-2.71049
H	4.390028	0.041928	-1.31278
H	5.225499	-2.50627	0.349102
H	4.386455	-1.11913	1.052463
H	3.705022	-2.74739	1.215948
H	-3.85773	2.72215	0.077414
H	-4.59081	1.974872	-1.3565

H	-4.68834	1.155047	0.191381
H	3.685703	2.746394	0.426793
H	2.914918	3.51176	-0.9801
H	2.735446	4.237282	0.633181
H	0.786566	1.613642	1.938437
H	-1.3944	-2.51063	3.035948
H	0.266059	-2.08825	2.594397
H	-0.94587	-0.82235	2.839335
H	-5.04726	-2.12277	0.127647

07_1R4R10S_17

C	3.42549	0.881704	0.228985
C	2.136863	1.250766	0.342208
C	3.894889	-0.56448	0.291711
C	1.5817	2.655294	0.378554
C	2.846135	-1.67796	0.430125
C	0.464049	2.953259	-0.65688
C	-1.00839	2.820849	-0.19058
C	-1.34967	1.413696	0.268704
C	-2.12109	0.551316	-0.39985
C	-2.456	-0.8586	0.026862
C	2.105108	-1.96928	-0.89236
C	0.678823	-2.44975	-0.70625
C	-0.33083	-1.66919	-1.12682
C	-1.81279	-1.875	-0.96815
C	-3.99628	-1.05452	0.155392
C	-4.38709	-2.49381	0.530872
C	-4.60077	-0.07508	1.174648
C	4.581545	1.84558	0.073256
C	-1.33702	3.828572	0.928077
O	-1.75726	3.177416	-1.36577
C	0.51807	-3.78791	-0.02643
O	3.484309	-2.90562	0.821122
H	1.383362	0.47145	0.439025
H	4.585031	-0.64769	1.147851
H	4.516836	-0.78426	-0.58995
H	1.202601	2.85578	1.391435
H	2.384834	3.38101	0.227222
H	2.118272	-1.39309	1.20373
H	0.572691	3.986906	-1.00789
H	0.591056	2.312019	-1.53659
H	-0.92587	1.119968	1.229926
H	-2.52655	0.858661	-1.36583
H	-2.00871	-1.04066	1.015673
H	2.708323	-2.718	-1.42631
H	2.102556	-1.06128	-1.50371
H	-0.05826	-0.73241	-1.61138
H	-2.30532	-1.75456	-1.94598
H	-2.03185	-2.89184	-0.63388
H	-4.43616	-0.83132	-0.82955
H	-5.46929	-2.56501	0.691885
H	-4.12521	-3.21835	-0.24614
H	-3.89393	-2.80348	1.461943
H	-5.69068	-0.18491	1.219501
H	-4.20572	-0.26813	2.181106
H	-4.37313	0.96566	0.924702
H	5.266242	1.773791	0.930091
H	5.174109	1.592705	-0.81651
H	4.274133	2.889088	-0.01775
H	-2.40648	3.78926	1.169009
H	-0.78766	3.61636	1.851465
H	-1.09219	4.845369	0.602299
H	-2.69828	3.127437	-1.12843
H	1.057943	-3.8085	0.927104
H	-0.52602	-4.0517	0.159435
H	0.963605	-4.58298	-0.6409
H	3.846903	-2.77443	1.712213

07_1R4R10S_18

C	-2.62532	1.480891	0.021636
C	-1.32605	1.557457	-0.31321
C	-3.11636	0.268796	0.796734
C	-0.59269	2.583775	-1.13739
C	-3.73868	-0.83871	-0.07337
C	0.58402	3.302748	-0.42988
C	1.581469	2.425198	0.379168
C	1.941147	1.153579	-0.37595
C	1.921457	-0.07916	0.140304
C	2.209749	-1.35694	-0.6119
C	-2.74259	-1.60443	-0.98001
C	-1.49677	-2.20274	-0.34485
C	-0.28432	-1.88272	-0.83264
C	1.070545	-2.38889	-0.39288
C	3.605352	-1.96991	-0.27475
C	4.741496	-1.1177	-0.85835
C	3.832338	-2.20855	1.22725
C	-3.67484	2.507947	-0.32516
C	2.85321	3.238379	0.66881
O	1.033339	2.129845	1.674866
C	-1.71538	-3.20689	0.763066

O	-4.42389	-1.70456	0.842627
H	-0.71221	0.708385	-0.02021
H	-2.29678	-0.16886	1.377153
H	-3.88935	0.572763	1.514556
H	-0.19037	2.067159	-2.02152
H	-1.27401	3.350822	-1.52143
H	-4.47994	-0.37994	-0.74872
H	1.142361	3.854505	-1.19744
H	0.194601	4.049917	0.27299
H	2.216315	1.297321	-1.42279
H	1.643373	-0.18524	1.188907
H	2.228562	-1.12006	-1.68641
H	-2.44363	-0.92498	-1.78614
H	-3.32193	-2.40946	-1.46418
H	-0.25266	-1.18147	-1.6682
H	1.046636	-2.68806	0.661248
H	1.330878	-3.29741	-0.95954
H	3.635584	-2.94945	-0.77569
H	5.716888	-1.578	-0.66038
H	4.637973	-1.0047	-1.94459
H	4.747665	-0.11423	-0.41668
H	4.790777	-2.71522	1.390774
H	3.861899	-1.26195	1.779794
H	3.050393	-2.83334	1.672958
H	-4.50783	2.050529	-0.87608
H	-4.10883	2.938357	0.587876
H	-3.28505	3.329138	-0.93175
H	3.501218	2.680473	1.352031
H	3.409239	3.437363	-0.25323
H	2.59519	4.196171	1.134967
H	0.133253	1.790081	1.535673
H	-2.28791	-4.06722	0.386167
H	-0.77836	-3.5911	1.173423
H	-2.31161	-2.78154	1.576482
H	-4.78469	-2.44587	0.330211

07_1R4R10S_19

C	-2.77826	0.964253	-0.82532
C	-1.55466	1.519996	-0.80506
C	-2.93312	-0.41175	-1.45021
C	-1.10748	2.834931	-0.22147
C	-3.29472	-1.57368	-0.49732
C	-0.02427	2.703467	0.878137
C	1.441715	2.423928	0.451285
C	1.594194	1.136979	-0.34227
C	1.939283	-0.05235	0.161237
C	2.045546	-1.33181	-0.63514
C	-2.59913	-1.56775	0.87336
C	-1.11012	-1.84133	0.998207
C	-0.31045	-2.16598	-0.03048
C	1.170677	-2.453	0.000489
C	3.518145	-1.80978	-0.83908
C	4.29299	-0.85222	-1.75579
C	4.286774	-2.03869	0.472656
C	-4.0392	1.610352	-0.30333
C	2.027282	3.602292	-0.34799
O	2.130372	2.326817	1.712945
C	-0.63679	-1.71337	2.429704
O	-4.7178	-1.5049	-0.29941
H	-0.75694	0.930826	-1.24923
H	-3.72291	-0.39649	-2.215
H	-2.00289	-0.67073	-1.96479
H	-0.73248	3.487944	-1.024
H	-1.95759	3.367553	0.217571
H	-3.05595	-2.51316	-1.02125
H	0.002573	3.637201	1.455038
H	-0.31498	1.90887	1.574987
H	1.395078	1.220825	-1.41094
H	2.132422	-0.1237	1.231752
H	1.644658	-1.13665	-1.64096
H	-3.11633	-2.31573	1.498976
H	-2.81244	-0.6036	1.355706
H	-0.74916	-2.22298	-1.02602
H	1.512987	-2.64073	1.022886
H	1.360868	-3.37934	-0.56145
H	3.451418	-2.77912	-1.35602
H	5.306929	-1.22554	-1.94288
H	3.793497	-0.73646	-2.72551
H	4.378323	0.143314	-1.30466
H	5.283402	-2.4446	0.262861
H	4.424955	-1.10116	1.024736
H	3.776853	-2.74651	1.135287
H	-3.85417	2.562703	0.200552
H	-4.7381	1.799531	-1.13076
H	-4.5648	0.942912	0.387756
H	3.089114	3.427797	-0.56101
H	1.518479	3.73888	-1.30859
H	1.938342	4.529106	0.228506
H	3.050502	2.087637	1.510873

H	-1.23881	-2.34637	3.096275
H	0.41169	-1.98332	2.568154
H	-0.76264	-0.68091	2.783703
H	-4.96294	-2.22021	0.309583

07_1R4R10S_20

C	2.860685	0.880541	0.662689
C	1.680374	1.479304	0.905263
C	3.08304	-0.54265	1.149805
C	1.261302	2.895101	0.589262
C	3.389526	-1.64062	0.104379
C	0.221255	3.059123	-0.55131
C	-1.28916	2.838537	-0.24483
C	-1.54472	1.456578	0.32348
C	-2.18116	0.452969	-0.28495
C	-2.30198	-0.94305	0.281229
C	2.337521	-1.79913	-1.0039
C	0.978198	-2.32667	-0.5776
C	-0.12659	-1.62544	-0.88286
C	-1.57468	-1.96605	-0.64278
C	-3.78403	-1.32547	0.587279
C	-4.67585	-1.4406	-0.65953
C	-3.88775	-2.59753	1.444525
C	4.041114	1.55009	-0.00166
C	-1.83351	3.911906	0.707296
O	-2.00701	3.042173	-1.47406
C	0.97671	-3.67632	0.103171
O	4.632748	-1.42189	-0.57896
H	0.927869	0.876238	1.409157
H	3.924034	-0.5453	1.864214
H	2.204838	-0.87118	1.718944
H	0.868189	3.362514	1.503713
H	2.137836	3.489105	0.311917
H	3.460111	-2.58745	0.663014
H	0.298886	4.079193	-0.94897
H	0.498266	2.379811	-1.36983
H	-1.13112	1.285597	1.316651
H	-2.61488	0.621578	-1.27131
H	-1.77409	-0.95251	1.245491
H	2.776018	-2.49643	-1.73373
H	2.220358	-0.83741	-1.51593
H	0.026801	-0.66938	-1.38193
H	-2.08915	-1.9823	-1.61529
H	-1.68169	-2.96948	-0.22057
H	-4.17722	-0.49601	1.192962
H	-5.7184	-1.6089	-0.36454
H	-4.65056	-0.53141	-1.27017
H	-4.37849	-2.28282	-1.29584
H	-4.92341	-2.76094	1.765531
H	-3.57482	-3.49108	0.891316
H	-3.2661	-2.52503	2.345314
H	3.839837	2.585234	-0.28747
H	4.909326	1.552098	0.673522
H	4.350457	0.995196	-0.89304
H	-2.91614	3.793659	0.817217
H	-1.37852	3.832519	1.69951
H	-1.63042	4.911526	0.308161
H	-1.61811	2.446671	-2.1359
H	1.443687	-3.63029	1.096125
H	-0.02859	-4.08308	0.234929
H	1.559388	-4.40346	-0.47907
H	5.318157	-1.29249	0.096188

07_1R4R10S_21

C	-2.63275	1.467237	0.024334
C	-1.33432	1.543488	-0.31392
C	-3.1263	0.248904	0.789097
C	-0.60453	2.571233	-1.13964
C	-3.73653	-0.86364	-0.09508
C	0.567196	3.299149	-0.43303
C	1.568483	2.429226	0.379134
C	1.938219	1.160158	-0.37504
C	1.92342	-0.07305	0.14012
C	2.220647	-1.34856	-0.61225
C	-2.73051	-1.62322	-0.98589
C	-1.48091	-2.20372	-0.33898
C	-0.27086	-1.88788	-0.8358
C	1.086682	-2.38682	-0.39609
C	3.618852	-1.95394	-0.27203
C	4.75174	-1.09592	-0.85339
C	3.843965	-2.19104	1.230509
C	-3.68229	2.495814	-0.31832
C	2.833668	3.251244	0.672144
O	1.018237	2.13058	1.67331
C	-1.69118	-3.19297	0.783547
O	-4.40823	-1.83544	0.717629
H	-0.71988	0.69333	-0.02522
H	-2.31393	-0.18818	1.37976
H	-3.9036	0.562768	1.502489
H	-0.19837	2.053363	-2.02119

H	-1.2892	3.333286	-1.52758
H	-4.46986	-0.40449	-0.77844
H	1.12309	3.85175	-1.20175
H	0.172674	4.046148	0.267191
H	2.215919	1.306088	-1.42089
H	1.642654	-0.18168	1.187758
H	2.240693	-1.11076	-1.68651
H	-2.43179	-0.947	-1.79379
H	-3.3061	-2.43679	-1.45249
H	-0.24331	-1.1981	-1.6812
H	1.064167	-2.68794	0.657421
H	1.352639	-3.29278	-0.96426
H	3.655325	-2.93347	-0.77254
H	5.729175	-1.55082	-0.65297
H	4.650093	-0.98401	-1.93991
H	4.751491	-0.0922	-0.41216
H	4.804354	-2.69331	1.396179
H	3.868149	-1.2441	1.782786
H	3.063941	-2.81922	1.674795
H	-4.51447	2.041297	-0.87305
H	-4.11721	2.922717	0.59609
H	-3.292	3.319886	-0.9205
H	3.483959	2.697717	1.356753
H	3.390532	3.454293	-0.24849
H	2.567891	4.207134	1.13786
H	0.122186	1.781753	1.530553
H	-2.2812	-4.04877	0.427131
H	-0.75091	-3.57976	1.184135
H	-2.27122	-2.75309	1.600926
H	-5.0522	-1.36038	1.267072
07_1R4R10S_22			
C	-2.81428	0.73401	-0.9982
C	-1.62798	1.363896	-0.96262
C	-2.84886	-0.70683	-1.47692
C	-1.29472	2.752191	-0.48245
C	-3.13557	-1.78172	-0.40304
C	-0.30548	2.784208	0.709861
C	1.206469	2.581975	0.424346
C	1.512413	1.268455	-0.27773
C	1.946368	0.152554	0.315078
C	2.231935	-1.16036	-0.3761
C	-2.47444	-1.56628	0.968677
C	-0.97708	-1.74452	1.154068
C	-0.12437	-2.06498	0.16773
C	1.365174	-2.28909	0.261
C	3.754248	-1.50437	-0.32918
C	4.082127	-2.86699	-0.9639
C	4.598229	-0.41199	-1.00576
C	-4.1404	1.343939	-0.61284
C	1.780626	3.75294	-0.3947
O	1.788942	2.604067	1.741315
C	-0.56007	-1.52602	2.591922
O	-4.56397	-1.80599	-0.23446
H	-0.76986	0.783785	-1.29132
H	-3.62341	-0.8375	-2.24589
H	-1.89207	-0.94381	-1.95184
H	-0.88166	3.343168	-1.31377
H	-2.20358	3.277652	-0.17127
H	-2.81002	-2.75153	-0.81243
H	-0.39117	3.757422	1.21052
H	-0.60059	2.024191	1.442579
H	1.363441	1.27147	-1.35791
H	2.096204	0.162479	1.395937
H	1.946987	-1.06707	-1.43568
H	-2.96691	-2.26116	1.670273
H	-2.75125	-0.56448	1.325745
H	-0.52377	-2.18945	-0.83783
H	1.684742	-2.42366	1.300959
H	1.593182	-3.23006	-0.25354
H	4.045577	-1.5472	0.732264
H	5.16727	-3.02111	-0.99291
H	3.649347	-3.70711	-0.41253
H	3.714388	-2.91605	-1.99762
H	5.666947	-0.64141	-0.92117
H	4.357705	-0.34131	-2.07511
H	4.424424	0.572398	-0.56178
H	-4.04548	2.357633	-0.21478
H	-4.80611	1.387545	-1.48674
H	-4.65498	0.722382	0.127908
H	2.866565	3.644251	-0.50555
H	1.351211	3.797218	-1.4016
H	1.57833	4.702044	0.112862
H	2.739854	2.436861	1.629249
H	-1.12596	-2.1834	3.266577
H	0.502878	-1.70089	2.766795
H	-0.78156	-0.49584	2.903228
H	-4.7665	-2.46814	0.446129
07_1R4R10S_23			

C	3.003195	0.614052	0.864634
C	2.486173	1.721584	0.308018
C	3.793632	-0.35861	0.008631
C	1.547684	2.697306	0.971609
C	2.92275	-1.4032	-0.74172
C	0.495819	3.308907	0.023639
C	-0.47598	2.347347	-0.71326
C	-1.19462	1.411646	0.249869
C	-1.62511	0.190085	-0.08363
C	-2.31991	-0.79435	0.827988
C	2.188821	-2.39744	0.213438
C	0.721592	-2.64486	-0.08814
C	-0.20607	-2.18581	0.767975
C	-1.70636	-2.21263	0.647322
C	-3.87138	-0.81691	0.653565
C	-4.51589	0.446451	1.24197
C	-4.32598	-1.01976	-0.80085
C	2.778396	0.273448	2.327445
C	-1.50579	3.200148	-1.47768
O	0.211856	1.604714	-1.73033
C	0.429776	-3.42401	-1.34705
O	1.961136	-0.7477	-1.58462
H	2.730773	1.940602	-0.73111
H	4.358801	0.195592	-0.75345
H	4.53438	-0.90384	0.60907
H	1.045135	2.218272	1.819993
H	2.119179	3.537948	1.398336
H	3.597839	-1.97933	-1.38959
H	-0.10342	4.023299	0.60479
H	1.006913	3.892379	-0.75348
H	-1.36894	1.79296	1.257289
H	-1.43314	-0.1535	-1.10003
H	-2.12888	-0.487	1.86695
H	2.274151	-2.04085	1.242078
H	2.738599	-3.34852	0.173574
H	0.154	-1.66393	1.656607
H	-2.0117	-2.62268	-0.32096
H	-2.1428	-2.86945	1.415557
H	-4.23099	-1.67663	1.239553
H	-5.60825	0.405911	1.153982
H	-4.26882	0.563502	2.30425
H	-4.16968	1.344255	0.716433
H	-5.41781	-1.107	-0.85014
H	-4.0334	-0.16932	-1.42823

H	-3.90368	-1.92669	-1.24761
H	3.502876	-0.46898	2.678174
H	2.880126	1.157814	2.966193
H	1.777005	-0.13783	2.513262
H	-2.15974	2.54877	-2.06512
H	-2.12445	3.784797	-0.78872
H	-0.99595	3.891796	-2.15901
H	0.706893	0.874641	-1.30804
H	0.91609	-4.40927	-1.30643
H	-0.63806	-3.58976	-1.50992
H	0.832691	-2.90197	-2.22194
H	2.455879	-0.29264	-2.28549
07_1R4R10S_24			
C	2.860593	0.880805	0.662442
C	1.680323	1.479622	0.905105
C	3.083057	-0.54225	1.149899
C	1.261218	2.895352	0.588879
C	3.389631	-1.64047	0.104758
C	0.22093	3.059216	-0.55148
C	-1.28943	2.83855	-0.24471
C	-1.54482	1.456559	0.323611
C	-2.18127	0.452935	-0.28478
C	-2.30199	-0.94312	0.281323
C	2.337777	-1.7991	-1.00365
C	0.97843	-2.32673	-0.57755
C	-0.12632	-1.6253	-0.88249
C	-1.57442	-1.96597	-0.64265
C	-3.78403	-1.32577	0.587114
C	-4.67559	-1.44122	-0.65985
C	-3.8877	-2.59774	1.444508
C	4.040876	1.550198	-0.00232
C	-1.83362	3.911882	0.707549
O	-2.00752	3.042169	-1.47379
C	0.97689	-3.67665	0.102695
O	4.632997	-1.42205	-0.57844
H	0.927931	0.876689	1.409322
H	3.924025	-0.54466	1.864345
H	2.204863	-0.87071	1.719083
H	0.868332	3.362989	1.503318
H	2.137719	3.489271	0.311232
H	3.460047	-2.58718	0.6636
H	0.298418	4.079271	-0.94921
H	0.49782	2.379875	-1.37003
H	-1.13112	1.285602	1.316744

H	-2.61508	0.621573	-1.27109
H	-1.77424	-0.95255	1.245671
H	2.776412	-2.49637	-1.73343
H	2.220615	-0.8374	-1.51572
H	0.027148	-0.66904	-1.38114
H	-2.08872	-1.98211	-1.61526
H	-1.68147	-2.96946	-0.2206
H	-4.1775	-0.49631	1.192616
H	-5.71817	-1.60961	-0.36503
H	-4.6503	-0.53213	-1.27065
H	-4.378	-2.2835	-1.29597
H	-4.92341	-2.76132	1.765278
H	-3.57445	-3.4913	0.891497
H	-3.26629	-2.52499	2.345441
H	3.839456	2.585188	-0.28858
H	4.909171	1.552601	0.672754
H	4.35016	0.994912	-0.89348
H	-2.91624	3.793641	0.817632
H	-1.37848	3.832438	1.69969
H	-1.63058	4.911518	0.308426
H	-1.61844	2.447042	-2.13585
H	1.444568	-3.63117	1.095342
H	-0.02847	-4.08308	0.234984
H	1.558912	-4.40377	-0.48023
H	5.318191	-1.29184	0.096772
07_1R4R10S_25			
C	-2.67744	1.172394	-0.66043
C	-1.41445	1.488002	-0.3307
C	-2.90975	-0.10038	-1.45719
C	-0.88773	2.636566	0.489708
C	-3.42874	-1.32149	-0.66314
C	0.447764	3.217666	-0.0262
C	1.736391	2.408095	0.276467
C	1.773607	1.066921	-0.4389
C	1.850308	-0.13882	0.133422
C	1.909611	-1.4534	-0.61476
C	-2.82839	-1.51934	0.738224
C	-1.36518	-1.88309	0.927596
C	-0.51129	-2.13786	-0.07733
C	0.949133	-2.50604	0.009981
C	3.359636	-2.02482	-0.7353
C	4.22536	-1.17496	-1.67613
C	4.065353	-2.21728	0.61696
C	-3.90304	1.96946	-0.28658

C	2.956967	3.238881	-0.17417
O	1.774197	2.251867	1.702196
C	-0.98875	-1.93076	2.392282
O	-4.85059	-1.14363	-0.53639
H	-0.64566	0.797149	-0.6619
H	-1.97325	-0.38698	-1.94482
H	-3.64165	0.072683	-2.25845
H	-1.61985	3.451473	0.536148
H	-0.73062	2.309879	1.527357
H	-3.23625	-2.2187	-1.27356
H	0.389731	3.379715	-1.11027
H	0.592846	4.204361	0.431506
H	1.750446	1.137101	-1.52808
H	1.849907	-0.20105	1.221848
H	1.568435	-1.2705	-1.64462
H	-3.42356	-2.30546	1.234511
H	-3.03024	-0.60597	1.314502
H	-0.88591	-2.07443	-1.09847
H	1.245844	-2.69405	1.04642
H	1.105134	-3.45281	-0.52913
H	3.252943	-3.01817	-1.1969
H	5.216763	-1.62566	-1.80504
H	3.766509	-1.08489	-2.6684
H	4.366137	-0.16253	-1.28007
H	5.042668	-2.69257	0.472259
H	4.238698	-1.256	1.115906
H	3.489737	-2.8517	1.299905
H	-4.44878	2.283684	-1.18774
H	-4.60291	1.355104	0.292417
H	-3.66622	2.865036	0.294791
H	3.888459	2.713748	0.070303
H	2.946174	3.41217	-1.25613
H	2.960313	4.20913	0.334181
H	2.608014	1.803245	1.918932
H	-1.65643	-2.60859	2.942072
H	0.0372	-2.25891	2.569106
H	-1.10622	-0.9396	2.851199
H	-5.19363	-1.89029	-0.01911
07_1R4R10S_26			
C	3.337779	1.163083	0.128124
C	2.023225	1.414906	0.26438
C	3.945778	-0.22817	0.234642
C	1.340005	2.763043	0.266524
C	3.014262	-1.43386	0.428559

C	0.140155	2.903769	-0.70803
C	-1.28468	2.643986	-0.15347
C	-1.46077	1.223479	0.353498
C	-2.1519	0.262891	-0.26886
C	-2.28678	-1.16373	0.206602
C	2.290944	-1.84437	-0.8718
C	0.931207	-2.47564	-0.64276
C	-0.16639	-1.80567	-1.03138
C	-1.61605	-2.1395	-0.80925
C	-3.75657	-1.57753	0.522355
C	-4.288	-0.83185	1.75575
C	-4.72363	-1.4163	-0.66199
C	4.393044	2.224194	-0.09648
C	-1.65103	3.646611	0.957318
O	-2.12703	2.891918	-1.29327
C	0.930005	-3.81959	0.043762
O	3.775094	-2.57695	0.854115
H	1.349637	0.573191	0.413185
H	4.651315	-0.21244	1.082167
H	4.57528	-0.41806	-0.64852
H	1.00508	2.985804	1.290382
H	2.060642	3.549444	0.02734
H	2.269176	-1.19477	1.201097
H	0.122064	3.928457	-1.09938
H	0.282437	2.244275	-1.57179
H	-0.97558	1.001053	1.305047
H	-2.61174	0.499724	-1.22863
H	-1.72557	-1.25935	1.146995
H	2.966649	-2.53319	-1.39941
H	2.173785	-0.95804	-1.50354
H	-0.00551	-0.85012	-1.5296
H	-2.14948	-2.07825	-1.76863
H	-1.74689	-3.16418	-0.44373
H	-3.72074	-2.64827	0.774221
H	-5.29955	-1.16914	2.011458
H	-3.64679	-0.99926	2.629761
H	-4.32921	0.249183	1.576545
H	-5.71382	-1.80619	-0.39816
H	-4.85017	-0.36209	-0.93634
H	-4.38433	-1.95654	-1.55245
H	5.101671	2.250943	0.743183
H	4.985063	1.992459	-0.99229
H	3.98518	3.229387	-0.21763
H	-2.69778	3.511403	1.256103
H	-1.03714	3.514218	1.854526
H	-1.52364	4.672553	0.595386
H	-3.04349	2.74524	-1.00484
H	1.473914	-3.77573	0.9944
H	-0.07645	-4.1999	0.235881
H	1.459675	-4.56088	-0.57112
H	4.128163	-2.37914	1.736576

07_1R4R10S_27

C	-3.43142	0.741697	-0.01943
C	-2.16208	1.153647	0.149345
C	-3.79784	-0.67256	-0.4435
C	-1.68263	2.523186	0.562467
C	-2.67003	-1.70097	-0.60952
C	-0.6383	3.168692	-0.38339
C	0.861493	2.851629	-0.12232
C	1.218877	1.399558	-0.39395
C	2.0022	0.637767	0.376669
C	2.425241	-0.78442	0.087149
C	-2.06349	-2.15464	0.737991
C	-0.58977	-2.49887	0.647524
C	0.314387	-1.68027	1.21218
C	1.816195	-1.76202	1.139383
C	3.979567	-0.88792	0.01807
C	4.475835	-2.32404	-0.22088
C	4.554821	0.040656	-1.06398
C	-4.65044	1.614229	0.181565
C	1.717112	3.751563	-1.03171
O	1.21663	3.237583	1.21427
C	-0.2576	-3.7463	-0.13502
O	-3.17463	-2.88319	-1.2528
H	-1.36165	0.435361	-0.01006
H	-4.5332	-1.08248	0.266365
H	-4.33861	-0.60695	-1.4024
H	-2.52698	3.212646	0.649153
H	-1.25787	2.471733	1.577664
H	-1.87829	-1.2655	-1.23647
H	-0.88265	2.917171	-1.42374
H	-0.73231	4.257843	-0.29529
H	0.832215	0.996164	-1.33103
H	2.399958	1.065491	1.299481
H	2.023603	-1.07417	-0.89549
H	-2.65336	-3.02009	1.071681
H	-2.20668	-1.36051	1.476795
H	-0.07046	-0.8122	1.74705

H	2.244265	-1.52194	2.124373
H	2.134316	-2.78041	0.903223
H	4.37145	-0.55425	0.99208
H	5.564586	-2.33265	-0.34977
H	4.241723	-2.99621	0.610184
H	4.032773	-2.74649	-1.13271
H	5.649907	-0.01021	-1.07503
H	4.198543	-0.25516	-2.05988
H	4.264455	1.083293	-0.90442
H	-5.28416	1.606304	-0.71598
H	-5.27178	1.226851	1.000991
H	-4.41102	2.654419	0.410918
H	2.779494	3.58046	-0.83365
H	1.520746	3.538072	-2.08753
H	1.490004	4.806645	-0.84208
H	0.813228	2.592225	1.817214
H	-0.71868	-3.71289	-1.12901
H	0.816776	-3.90503	-0.25591
H	-0.67788	-4.63194	0.362299
H	-3.44134	-2.63452	-2.15268

07_1R4R10S_28

C	3.358856	1.10948	0.204055
C	2.048241	1.36653	0.362816
C	3.94692	-0.29393	0.233823
C	1.367223	2.713986	0.42162
C	2.99907	-1.49245	0.397108
C	0.255696	2.928586	-0.64396
C	-1.22173	2.66519	-0.23389
C	-1.41616	1.25283	0.285525
C	-2.22459	0.329121	-0.24316
C	-2.32477	-1.10165	0.230392
C	2.242237	-1.83984	-0.90334
C	0.872768	-2.4512	-0.67432
C	-0.21753	-1.75236	-1.03271
C	-1.67108	-2.06845	-0.80775
C	-3.77422	-1.53796	0.597379
C	-4.29267	-0.76034	1.816817
C	-4.77634	-1.44611	-0.56529
C	4.422385	2.169295	0.018679
C	-1.70341	3.683512	0.809842
O	-2.03943	2.914971	-1.38903
C	0.855654	-3.81424	-0.02655
O	3.749617	-2.66364	0.759054
H	1.370828	0.522188	0.471948
H	4.552434	-0.45353	-0.67188
H	4.672029	-0.32804	1.063853
H	0.937193	2.848749	1.425078
H	2.102565	3.515763	0.315611
H	2.274391	-1.27312	1.194781
H	0.288067	3.967443	-0.99435
H	0.477646	2.300838	-1.51824
H	-0.81929	0.994095	1.160898
H	-2.82684	0.59956	-1.11039
H	-1.72905	-1.19186	1.149855
H	2.893235	-2.52381	-1.46701
H	2.134043	-0.92995	-1.50241
H	-0.04463	-0.78625	-1.5056
H	-2.20957	-1.98596	-1.76274
H	-1.81264	-3.09738	-0.4582
H	-3.705	-2.59769	0.886505
H	-5.2815	-1.1238	2.121157
H	-3.61678	-0.86684	2.674147
H	-4.38172	0.309749	1.594826
H	-5.74897	-1.84845	-0.25819
H	-4.93822	-0.40748	-0.8772
H	-4.44828	-2.01443	-1.44245
H	5.148127	2.140975	0.843444
H	4.993362	1.982325	-0.90098
H	4.023121	3.183783	-0.03809
H	-2.77181	3.53961	0.998602
H	-1.17092	3.566741	1.758651
H	-1.54609	4.70416	0.444051
H	-1.7232	2.325894	-2.09434
H	1.420894	-3.80968	0.912493
H	-0.15474	-4.17767	0.176834
H	1.354986	-4.54877	-0.67408
H	4.125393	-2.50783	1.640603

07_1R4R10R_3

C	2.486319	1.669859	0.422086
C	1.198497	1.957385	0.679736
C	3.098811	0.401604	0.992923
C	0.409506	3.179873	0.28206
C	3.834071	-0.51956	-0.00143
C	-0.74074	2.943558	-0.72794
C	-2.06347	2.314311	-0.21692
C	-1.85812	0.921981	0.352531
C	-2.04818	-0.22191	-0.31204
C	-1.76974	-1.59995	0.238454

C	2.942208	-1.17153	-1.0743	H	2.791248	-3.93204	-0.56648
C	1.772194	-2.03344	-0.62389	H	5.082754	-1.1461	1.368487
C	0.52009	-1.64973	-0.93002	07_1R4R10R_4			
C	-0.78075	-2.36945	-0.68543	C	2.578689	1.393456	0.47745
C	-3.06283	-2.4328	0.504947	C	1.44502	1.965216	0.920599
C	-3.8411	-1.87563	1.705967	C	2.900163	-0.03267	0.879312
C	-3.98462	-2.56288	-0.71841	C	0.949882	3.367641	0.657152
C	3.430239	2.563662	-0.34978	C	3.184541	-0.99354	-0.29203
C	-2.75447	3.213206	0.82417	C	-0.57856	3.539518	0.742457
O	-2.87639	2.265903	-1.40473	C	-1.42967	2.674815	-0.23242
C	2.122874	-3.33138	0.066233	C	-1.65583	1.267585	0.308318
O	4.511901	-1.56701	0.705709	C	-1.78428	0.165656	-0.43745
H	0.646455	1.221946	1.260917	C	-2.08446	-1.22412	0.079344
H	2.334576	-0.18507	1.514632	C	2.793837	-2.4436	0.028951
H	3.845627	0.695815	1.750965	C	1.320865	-2.82248	-0.00511
H	1.07416	3.927502	-0.16229	C	0.349414	-1.9686	-0.37306
H	0.001114	3.651189	1.187943	C	-1.11857	-2.26176	-0.5577
H	4.583688	0.07726	-0.5448	C	-3.57973	-1.59988	-0.18687
H	-1.01724	3.912129	-1.16471	C	-3.91722	-3.04676	0.216361
H	-0.37831	2.316453	-1.55081	C	-4.54221	-0.63933	0.529487
H	-1.512	0.884304	1.38549	C	3.606951	2.101884	-0.37123
H	-2.38715	-0.16772	-1.34718	C	-2.79226	3.355911	-0.44507
H	-1.26952	-1.47912	1.209916	O	-0.82336	2.636657	-1.53438
H	3.61115	-1.78195	-1.69937	C	1.091857	-4.27135	0.364324
H	2.566818	-0.36368	-1.71325	O	4.595424	-0.90981	-0.55303
H	0.408687	-0.69405	-1.44236	H	0.809242	1.366309	1.572575
H	-1.26847	-2.53181	-1.65873	H	2.073284	-0.43094	1.475964
H	-0.61478	-3.36322	-0.25628	H	3.797466	-0.05867	1.516105
H	-2.72284	-3.44463	0.772653	H	1.391756	4.060834	1.389721
H	-4.72511	-2.48856	1.919039	H	1.287978	3.722927	-0.32295
H	-3.21977	-1.85695	2.609658	H	2.637459	-0.66217	-1.18671
H	-4.18086	-0.85082	1.514208	H	-0.918	3.343418	1.76873
H	-4.82424	-3.23078	-0.49269	H	-0.80015	4.594745	0.540737
H	-4.40577	-1.59159	-1.00484	H	-1.77207	1.195165	1.390593
H	-3.46386	-2.97316	-1.5905	H	-1.68938	0.260048	-1.52149
H	4.360111	2.722648	0.21345	H	-1.93152	-1.23465	1.169162
H	3.72144	2.111763	-1.30734	H	3.217118	-2.7062	1.01089
H	3.003135	3.544433	-0.57094	H	3.318398	-3.09884	-0.68634
H	-3.73464	2.79941	1.091143	H	0.628446	-0.94459	-0.6113
H	-2.17105	3.294792	1.747739	H	-1.33497	-2.28951	-1.63955
H	-2.90372	4.218147	0.415412	H	-1.35888	-3.25602	-0.17807
H	-3.69993	1.810583	-1.16147	H	-3.75003	-1.50389	-1.27075
H	2.674733	-3.1425	0.992392	H	-4.99304	-3.22767	0.107695
H	1.244738	-3.93886	0.298501	H	-3.40013	-3.7905	-0.39699

H	-3.65644	-3.23291	1.266757
H	-5.58262	-0.88729	0.28871
H	-4.42788	-0.71534	1.619308
H	-4.36713	0.402298	0.247627
H	3.27173	3.086466	-0.70679
H	4.535471	2.241531	0.200059
H	3.883427	1.507249	-1.24865
H	-3.40844	2.762866	-1.12762
H	-3.32701	3.459788	0.505212
H	-2.65059	4.354532	-0.87369
H	-0.02084	2.094668	-1.44586
H	1.59536	-4.93915	-0.34893
H	1.525086	-4.48987	1.349896
H	0.037307	-4.55249	0.392939
H	4.772396	-1.41251	-1.36457
07_1R4R10R_5			
C	2.39171	1.592071	0.648284
C	1.426146	1.913948	-0.22864
C	2.789431	0.127697	0.825544
C	0.776939	3.246554	-0.48847
C	3.189673	-0.62546	-0.4609
C	-0.6239	3.409672	0.151397
C	-1.73927	2.427701	-0.30627
C	-1.56944	1.035175	0.287092
C	-1.84831	-0.11736	-0.33336
C	-1.76611	-1.4907	0.295633
C	3.067207	-2.14347	-0.3089
C	1.661772	-2.72229	-0.20017
C	0.566568	-2.04537	-0.58609
C	-0.87882	-2.46307	-0.53793
C	-3.17757	-2.11024	0.557724
C	-3.93713	-1.33755	1.645514
C	-4.0412	-2.2519	-0.70639
C	3.085213	2.571351	1.562965
C	-3.10065	2.989027	0.142653
O	-1.81624	2.385521	-1.74049
C	1.644945	-4.12954	0.350692
O	4.557578	-0.36169	-0.80399
H	0.996628	1.095677	-0.80289
H	1.952605	-0.40415	1.295616
H	3.637445	0.043979	1.516573
H	1.401872	4.069182	-0.12423
H	0.678685	3.399987	-1.57259
H	2.555591	-0.29604	-1.29399

H	-0.52991	3.342833	1.243184
H	-0.97194	4.42541	-0.07568
H	-1.24163	1.015274	1.327235
H	-2.18482	-0.07947	-1.37048
H	-1.28904	-1.38499	1.280396
H	3.645656	-2.44794	0.576202
H	3.582437	-2.60749	-1.16318
H	0.70422	-1.04303	-0.98272
H	-1.27133	-2.50216	-1.56548
H	-0.99136	-3.47203	-0.12754
H	-2.99099	-3.12331	0.945001
H	-4.8929	-1.82498	1.872424
H	-3.35827	-1.28534	2.575855
H	-4.1505	-0.3107	1.32725
H	-4.97633	-2.77312	-0.46982
H	-4.30842	-1.27263	-1.12101
H	-3.53602	-2.82412	-1.49231
H	2.771644	3.606617	1.405857
H	2.893265	2.319754	2.615532
H	4.17489	2.521518	1.430567
H	-3.90495	2.322943	-0.1834
H	-3.14377	3.080074	1.233033
H	-3.26377	3.980023	-0.29561
H	-1.04078	1.895589	-2.05873
H	2.305408	-4.78068	-0.23869
H	2.030994	-4.14875	1.379266
H	0.650864	-4.58187	0.355418
H	4.630527	0.59583	-0.94752
07_1R4R10R_6			
C	2.521936	1.263436	0.777575
C	1.730568	1.769258	-0.18302
C	2.724704	-0.24527	0.892616
C	1.282195	3.191807	-0.38868
C	3.063871	-0.99502	-0.41156
C	-0.11734	3.51106	0.19006
C	-1.3231	2.70568	-0.37066
C	-1.3963	1.290014	0.190956
C	-1.83908	0.214903	-0.47078
C	-2.03815	-1.16916	0.109216
C	2.723181	-2.48381	-0.33171
C	1.248038	-2.86276	-0.31947
C	0.269744	-1.99758	-0.6395
C	-1.21769	-2.22952	-0.68487
C	-3.55973	-1.52779	0.14476

C	-3.83113	-2.93734	0.69996
C	-4.36429	-0.50147	0.959321
C	3.180489	2.083167	1.859981
C	-2.61944	3.447385	0.003872
O	-1.30573	2.703578	-1.80786
C	1.020558	-4.30921	0.056356
O	4.468249	-0.91191	-0.69457
H	1.296002	1.063051	-0.88795
H	1.814344	-0.68114	1.324596
H	3.534959	-0.46456	1.599527
H	1.990368	3.898772	0.057294
H	1.267355	3.416522	-1.46384
H	2.516249	-0.55272	-1.25328
H	-0.09389	3.384952	1.280437
H	-0.31603	4.573361	-0.00165
H	-1.12581	1.195825	1.243212
H	-2.12623	0.331766	-1.51846
H	-1.67474	-1.16901	1.148017
H	3.205581	-2.90303	0.564374
H	3.211583	-2.98411	-1.18134
H	0.552545	-0.98268	-0.90321
H	-1.5595	-2.19967	-1.73295
H	-1.46048	-3.22643	-0.31358
H	-3.92644	-1.49759	-0.8934
H	-4.91011	-3.10081	0.804657
H	-3.44597	-3.73175	0.054015
H	-3.38091	-3.06149	1.693906
H	-5.42999	-0.75874	0.956044
H	-4.02992	-0.48696	2.005484
H	-4.25872	0.512168	0.56328
H	2.992587	3.155622	1.76507
H	2.826842	1.768546	2.851946
H	4.267889	1.925402	1.858333
H	-3.48696	2.909904	-0.39041
H	-2.72273	3.525981	1.091255
H	-2.6095	4.45864	-0.41808
H	-0.59858	2.099988	-2.0877
H	1.58931	-4.9734	-0.60912
H	1.38438	-4.5074	1.074042
H	-0.02766	-4.6114	0.008327
H	4.679926	0.032378	-0.77592

07_1R4R10R_7

C	2.652916	1.250851	0.393651
C	1.575918	1.898778	0.871818
C	2.903765	-0.1869	0.806956
C	1.16402	3.329027	0.60807
C	3.094734	-1.18179	-0.35567
C	-0.33493	3.623561	0.808793
C	-1.32703	2.835376	-0.0944
C	-1.59261	1.437499	0.4468
C	-1.77465	0.340176	-0.29405
C	-2.09979	-1.03087	0.258922
C	2.620105	-2.5999	-0.00374
C	1.124776	-2.87944	0.01239
C	0.206224	-1.96764	-0.34984
C	-1.28766	-2.1358	-0.46859
C	-3.6424	-1.29064	0.239596
C	-4.249	-1.3362	-1.1719
C	-4.03419	-2.54261	1.040988
C	3.68475	1.882989	-0.50942
C	-2.6571	3.603681	-0.17079
O	-0.84488	2.784243	-1.44635
C	0.803907	-4.29723	0.429122
O	4.500691	-1.19456	-0.65366
H	0.930052	1.350127	1.556959
H	2.075089	-0.52615	1.436843
H	3.818066	-0.25588	1.415798
H	1.714178	4.002539	1.283423
H	1.449109	3.637398	-0.40479
H	2.547739	-0.82814	-1.24168
H	-0.60876	3.452926	1.859031
H	-0.48581	4.693578	0.619957
H	-1.67639	1.367608	1.532738
H	-1.70474	0.429875	-1.37897
H	-1.80328	-1.04396	1.317588
H	3.05492	-2.87784	0.968909
H	3.07954	-3.29622	-0.72506
H	0.552679	-0.97322	-0.62355
H	-1.55165	-2.10635	-1.53741
H	-1.60302	-3.11738	-0.10565
H	-4.0839	-0.42622	0.755736
H	-5.34009	-1.42781	-1.11345
H	-4.02365	-0.42864	-1.74293
H	-3.88033	-2.19579	-1.74435
H	-5.12405	-2.6036	1.145489
H	-3.70402	-3.46576	0.550164
H	-3.60175	-2.52461	2.048826
H	4.649362	1.957745	0.011838

H	3.87188	1.269584	-1.39799
H	3.402154	2.887876	-0.83314
H	-3.3699	3.061023	-0.79902
H	-3.0938	3.723845	0.826448
H	-2.49245	4.598676	-0.59948
H	-0.07504	2.190439	-1.442
H	1.260811	-5.01922	-0.26229
H	1.224508	-4.51179	1.421117
H	-0.26705	-4.50653	0.467402
H	4.622575	-1.7233	-1.45874
07_1R4R10R_8			
C	2.358667	1.69296	0.581384
C	1.308202	1.924018	-0.22371
C	2.810783	0.251747	0.809378
C	0.600516	3.21446	-0.52777
C	3.244359	-0.51679	-0.45778
C	-0.77457	3.358057	0.168087
C	-1.89169	2.360323	-0.22873
C	-1.68147	0.962779	0.34143
C	-1.77879	-0.18982	-0.33023
C	-1.69247	-1.57108	0.284089
C	3.178209	-2.03429	-0.26845
C	1.79363	-2.65942	-0.14511
C	0.67837	-2.03849	-0.56637
C	-0.74986	-2.51231	-0.52176
C	-3.09869	-2.23076	0.473457
C	-3.91191	-1.52296	1.566279
C	-3.91693	-2.33506	-0.82404
C	3.114836	2.750729	1.347292
C	-3.23439	2.894981	0.318525
O	-1.93476	2.355152	-1.66232
C	1.822515	-4.04354	0.461245
O	4.601836	-0.20961	-0.80801
H	0.84731	1.058535	-0.69242
H	1.991807	-0.30118	1.28482
H	3.657226	0.218563	1.506635
H	1.206605	4.07875	-0.23282
H	0.442308	3.296637	-1.6104
H	2.597441	-0.22817	-1.29624
H	-0.63954	3.300466	1.255637
H	-1.1521	4.365168	-0.05108
H	-1.50706	0.936216	1.418393
H	-1.94021	-0.15468	-1.40883
H	-1.26379	-1.46836	1.291199

H	3.766177	-2.29533	0.624226
H	3.710104	-2.5011	-1.11095
H	0.784485	-1.04903	-1.00311
H	-1.12737	-2.58266	-1.55332
H	-0.8251	-3.51921	-0.09835
H	-2.90336	-3.25545	0.823923
H	-4.8659	-2.03689	1.734939
H	-3.36837	-1.50375	2.518852
H	-4.13359	-0.48643	1.287379
H	-4.84801	-2.88485	-0.64286
H	-4.19058	-1.3432	-1.20367
H	-3.37388	-2.8606	-1.61726
H	2.775675	3.766805	1.128984
H	3.017973	2.590954	2.430594
H	4.190181	2.696855	1.126819
H	-4.05672	2.227863	0.032496
H	-3.22345	2.957942	1.412383
H	-3.43472	3.893255	-0.08576
H	-2.73606	1.875126	-1.92735
H	2.512597	-4.6928	-0.09542
H	2.19824	-4.0075	1.493249
H	0.84563	-4.53173	0.475245
H	4.633562	0.74224	-0.99669
07_1R4R10R_9			
C	2.610907	1.483796	0.432716
C	1.368776	1.893714	0.743824
C	3.106442	0.141282	0.944468
C	0.701582	3.205316	0.410666
C	3.714493	-0.81654	-0.09974
C	-0.46683	3.133733	-0.60394
C	-1.84731	2.625601	-0.11334
C	-1.78019	1.206475	0.41961
C	-2.09818	0.10425	-0.26492
C	-1.96727	-1.30312	0.267789
C	2.725423	-1.35002	-1.15278
C	1.4837	-2.09048	-0.67828
C	0.271036	-1.57893	-0.95473
C	-1.09191	-2.16503	-0.68747
C	-3.36118	-1.93866	0.57426
C	-4.2493	-2.13025	-0.6658
C	-3.23785	-3.25199	1.363545
C	3.616363	2.300151	-0.34722
C	-2.4504	3.56113	0.95001
O	-2.65672	2.688174	-1.30262

C	1.714541	-3.42037	0.000844
O	4.313056	-1.9442	0.554575
H	0.763611	1.199756	1.32333
H	2.300795	-0.38224	1.471258
H	3.899633	0.3326	1.688064
H	1.43691	3.9046	0.000377
H	0.343148	3.668816	1.341327
H	4.498738	-0.27957	-0.65633
H	-0.63916	4.142682	-1.0011
H	-0.17215	2.504918	-1.45193
H	-1.42773	1.108428	1.446652
H	-2.44385	0.212937	-1.29341
H	-1.43794	-1.24534	1.22977
H	3.306381	-2.01649	-1.808
H	2.418812	-0.4959	-1.76769
H	0.246437	-0.61231	-1.45796
H	-1.61752	-2.25475	-1.65017
H	-1.01396	-3.1791	-0.28557
H	-3.86901	-1.21545	1.229136
H	-5.24383	-2.48486	-0.37003
H	-4.38399	-1.1956	-1.22147
H	-3.83063	-2.8731	-1.35527
H	-4.22477	-3.59218	1.699358
H	-2.80611	-4.05643	0.756431
H	-2.60669	-3.12621	2.251806
H	4.577002	2.342835	0.184397
H	3.824524	1.85247	-1.32814
H	3.287038	3.326701	-0.52349
H	-3.46436	3.234203	1.211144
H	-1.86017	3.568076	1.872865
H	-2.50601	4.584267	0.563508
H	-3.52724	2.325181	-1.06753
H	2.3237	-3.29424	0.901203
H	0.7841	-3.92434	0.273676
H	2.2831	-4.09307	-0.65669
H	4.953429	-1.59949	1.1977

07_1R4R10R_10

C	3.27304	0.824162	0.613435
C	1.965194	1.127207	0.562332
C	3.840329	-0.43663	-0.02098
C	1.244585	2.28997	1.190589
C	2.845732	-1.46646	-0.57854
C	0.460082	3.205268	0.22413
C	-0.64817	2.570983	-0.65582
C	-1.48856	1.575591	0.127732
C	-1.779	0.327274	-0.2507
C	-2.59781	-0.67243	0.536592
C	2.054577	-2.20254	0.529342
C	0.622464	-2.52316	0.145919
C	-0.39201	-1.94151	0.809258
C	-1.87192	-2.05179	0.546324
C	-4.05394	-0.79947	-0.00925
C	-4.85741	-1.89656	0.710256
C	-4.81583	0.532108	0.075365
C	4.32789	1.672065	1.287003
C	-1.55294	3.697087	-1.19897
O	0.023428	1.946884	-1.76302
C	0.462801	-3.49267	-1.00085
O	3.549434	-2.47692	-1.32205
H	1.299565	0.446611	0.042725
H	4.485581	-0.94895	0.709839
H	4.518648	-0.1404	-0.83896
H	0.532546	1.88672	1.926493
H	1.931776	2.926315	1.758578
H	2.143435	-0.95708	-1.2536
H	-0.00897	3.990863	0.830447
H	1.154852	3.706841	-0.46035
H	-1.88484	1.953245	1.072304
H	-1.37257	-0.04344	-1.19248
H	-2.66832	-0.314	1.575218
H	2.057986	-1.59348	1.438129
H	2.61567	-3.12183	0.751024
H	-0.13026	-1.28143	1.637903
H	-2.05786	-2.55341	-0.41143
H	-2.33662	-2.68196	1.31682
H	-3.97799	-1.07932	-1.07202
H	-5.89085	-1.91399	0.344372
H	-4.43786	-2.89511	0.555718
H	-4.89334	-1.70917	1.791802
H	-5.81655	0.43277	-0.36154
H	-4.94115	0.843286	1.121287
H	-4.2929	1.335814	-0.4508
H	4.789389	1.1312	2.125319
H	5.141273	1.905163	0.58571
H	3.941012	2.618309	1.672062
H	-2.27737	3.290706	-1.91553
H	-2.11689	4.182158	-0.39468
H	-0.94811	4.45639	-1.70764

H	-0.66166	1.658312	-2.38808
H	1.074138	-3.19031	-1.85896
H	0.827119	-4.48774	-0.7079
H	-0.57274	-3.60275	-1.33184
H	3.95634	-2.04111	-2.08843
07_1R4R10R_11			
C	-3.2783	0.750502	-0.59042
C	-1.96926	1.043257	-0.69624
C	-3.79139	-0.5695	-0.04124
C	-1.32278	2.280553	-1.27174
C	-2.77259	-1.56626	0.516888
C	-0.57549	3.194573	-0.26912
C	0.488695	2.536245	0.651427
C	1.381493	1.58527	-0.13242
C	1.784164	0.380593	0.282568
C	2.646194	-0.57987	-0.5061
C	-1.9343	-2.28442	-0.5819
C	-0.49735	-2.54387	-0.17134
C	0.512476	-1.95058	-0.83098
C	1.987549	-1.99261	-0.52605
C	4.104679	-0.64017	0.04469
C	4.966824	-1.6873	-0.68109
C	4.795949	0.730331	-0.02315
C	-4.39312	1.68072	-1.00951
C	1.342532	3.636105	1.303275
O	-0.14945	1.871179	1.755034
C	-0.31905	-3.4559	1.021301
O	-3.54424	-2.50161	1.285466
H	-1.2528	0.283097	-0.39249
H	-4.38534	-1.07262	-0.8206
H	-4.50426	-0.35595	0.768059
H	-0.60377	1.952306	-2.03569
H	-2.05671	2.900342	-1.79672
H	-2.09156	-1.02711	1.193747
H	-0.08679	3.986412	-0.85234
H	-1.29879	3.689577	0.391031
H	1.70818	1.952856	-1.10736
H	1.45615	0.038367	1.264847
H	2.70237	-0.21656	-1.54398
H	-1.94659	-1.6909	-1.50175
H	-2.4575	-3.22587	-0.80519
H	0.248467	-1.33268	-1.69099
H	2.172411	-2.47964	0.438937
H	2.499251	-2.60191	-1.28324

H	4.038638	-0.93303	1.104419
H	5.997477	-1.6562	-0.30838
H	4.598542	-2.70797	-0.53978
H	5.000286	-1.48696	-1.76047
H	5.801849	0.676699	0.409794
H	4.90121	1.062486	-1.0649
H	4.233597	1.497555	0.51639
H	-4.03524	2.625844	-1.42277
H	-5.03324	1.202205	-1.76332
H	-5.04273	1.9131	-0.15477
H	2.006301	3.19207	2.051797
H	1.955685	4.150641	0.556208
H	0.70091	4.374518	1.797854
H	-0.83631	1.293133	1.383395
H	-0.74253	-3.01552	1.936181
H	-0.83829	-4.41023	0.855915
H	0.726976	-3.68521	1.234909
H	-2.92792	-3.1354	1.684155
07_1R4R10R_12			
C	2.314218	1.769716	0.386668
C	1.100198	2.143318	0.827719
C	2.895503	0.442588	0.834071
C	0.356822	3.422528	0.516392
C	3.34806	-0.49003	-0.30744
C	-1.16997	3.350972	0.708526
C	-1.93818	2.322472	-0.17054
C	-1.85007	0.913509	0.400705
C	-1.78661	-0.21196	-0.31856
C	-1.74558	-1.61203	0.251584
C	3.225754	-1.97629	0.060696
C	1.841945	-2.60841	0.066075
C	0.735843	-1.95211	-0.32458
C	-0.66971	-2.48001	-0.45671
C	-3.14059	-2.31875	0.219383
C	-4.11804	-1.67976	1.216034
C	-3.76959	-2.38682	-1.18161
C	3.183033	2.619837	-0.50883
C	-3.41657	2.738717	-0.25287
O	-1.46204	2.363352	-1.52512
C	1.866674	-4.05475	0.507024
O	4.72025	-0.1614	-0.58052
H	0.591517	1.466631	1.513683
H	2.157927	-0.07972	1.451522
H	3.784848	0.604329	1.461844

H	0.722486	4.232079	1.166926	C	0.343703	-2.01739	-0.62624
H	0.566919	3.756034	-0.50685	C	-1.13567	-2.2989	-0.6432
H	2.749826	-0.29219	-1.20885	C	-3.50993	-1.61316	0.100016
H	-1.40126	3.148665	1.763313	C	-3.78324	-3.05614	0.561031
H	-1.57396	4.346869	0.488983	C	-4.33823	-0.64542	0.960288
H	-1.88297	0.849238	1.489158	C	3.289544	2.215214	1.699236
H	-1.76894	-0.12832	-1.40632	C	-2.75351	3.394582	0.08778
H	-1.46117	-1.53962	1.311085	O	-1.33726	2.687359	-1.75563
H	3.701756	-2.12887	1.04186	C	1.176062	-4.26099	0.190322
H	3.848919	-2.55003	-0.64559	O	4.498075	-0.7827	-0.74401
H	0.835049	-0.90802	-0.6144	H	1.123423	1.061895	-0.78252
H	-0.91755	-2.529	-1.52871	H	1.864319	-0.61066	1.311488
H	-0.74788	-3.50557	-0.08151	H	3.58254	-0.35005	1.571333
H	-2.96112	-3.35197	0.553369	H	1.838615	3.9379	0.026642
H	-5.06679	-2.22934	1.235733	H	1.08984	3.394488	-1.46901
H	-3.70873	-1.68109	2.23376	H	2.523983	-0.48803	-1.26426
H	-4.33789	-0.64076	0.944969	H	-0.21268	3.362163	1.302176
H	-4.70549	-2.95718	-1.15072	H	-0.47434	4.552354	0.030329
H	-4.00686	-1.38526	-1.55972	H	-1.40918	1.191107	1.316383
H	-3.11184	-2.87446	-1.90952	H	-1.87526	0.210144	-1.54757
H	4.083808	2.940897	0.032802	H	-1.66567	-1.23582	1.169026
H	3.541904	2.054394	-1.37595	H	3.334959	-2.79939	0.55699
H	2.669987	3.517071	-0.8642	H	3.29493	-2.90107	-1.18744
H	-3.97411	2.025171	-0.86709	H	0.594182	-1.01375	-0.95743
H	-3.8681	2.766364	0.744612	H	-1.48637	-2.31875	-1.68897
H	-3.50334	3.735142	-0.70082	H	-1.34166	-3.28936	-0.23432
H	-0.57055	1.975857	-1.51581	H	-3.85567	-1.52168	-0.94182
H	2.307321	-4.14456	1.50939	H	-4.8628	-3.22815	0.643788
H	0.878155	-4.51785	0.533921	H	-3.38823	-3.80549	-0.13108
H	2.498411	-4.65528	-0.16256	H	-3.34303	-3.24265	1.549686
H	4.9852	-0.65172	-1.37548	H	-5.40436	-0.89412	0.901517
07_1R4R10R_13				H	-4.04066	-0.71187	2.015513
C	2.524909	1.345918	0.73084	H	-4.21389	0.393953	0.645069
C	1.628174	1.79801	-0.16159	H	3.08942	3.282285	1.572528
C	2.760646	-0.15645	0.870472	H	3.042524	1.949945	2.737019
C	1.142023	3.201783	-0.39067	H	4.37189	2.057044	1.592953
C	3.10271	-0.90862	-0.43211	H	-3.62827	2.860113	-0.30233
C	-0.25167	3.493121	0.213335	H	-2.85412	3.445161	1.177745
C	-1.45049	2.673307	-0.32557	H	-2.76017	4.415616	-0.30952
C	-1.50944	1.254599	0.231534	H	-2.16868	2.340706	-2.11765
C	-1.77572	0.142578	-0.46148	H	1.765633	-4.94276	-0.43832
C	-1.98938	-1.24172	0.11725	H	1.549495	-4.38726	1.216001
C	2.81412	-2.4073	-0.32961	H	0.138823	-4.60169	0.165354
C	1.352296	-2.83223	-0.27055	H	4.672413	0.165629	-0.85882

07_1R4R10R_14				H	4.974141	-0.2622	-1.33534
C	-2.86179	1.092204	0.071944	H	4.558586	0.623299	0.14007
C	-1.56397	1.343042	-0.16663	H	-3.70222	2.668961	-1.18528
C	-3.22384	-0.06416	0.991027	H	-4.74697	1.249532	-0.99639
C	-0.93514	2.336548	-1.10358	H	-4.57068	2.376939	0.329963
C	-3.58429	-1.40347	0.300672	H	3.235569	3.29592	1.063678
C	0.118207	3.280149	-0.48092	H	2.878635	3.888761	-0.56706
C	1.294502	2.644734	0.307277	H	2.061498	4.613659	0.836781
C	1.8136	1.378201	-0.35544	H	1.538617	2.073908	2.163403
C	1.900256	0.1785	0.227037	H	-1.70688	-2.73876	2.082708
C	2.413204	-1.09446	-0.40643	H	-1.81822	-4.13714	1.022045
C	-2.49018	-1.99449	-0.61378	H	-0.23459	-3.57266	1.571082
C	-1.15478	-2.34601	0.019414	H	-4.61337	-0.90161	-1.28793
C	-0.01043	-1.93319	-0.5525	07_1R4R10R_15			
C	1.40858	-2.25046	-0.14412	C	-2.6207	-1.54754	0.346266
C	3.845314	-1.45049	0.106054	C	-1.30614	-1.81336	0.27396
C	4.348128	-2.8047	-0.42322	C	-3.08686	-0.23483	0.950783
C	4.862027	-0.35372	-0.24661	C	-0.62354	-3.0391	-0.28425
C	-4.0178	1.890131	-0.48621	C	-3.75055	0.750429	-0.0349
C	2.437894	3.675705	0.413127	C	0.692615	-3.42895	0.42602
O	0.7896	2.381132	1.626461	C	1.965204	-2.60812	0.062682
C	-1.21973	-3.24065	1.235916	C	1.848449	-1.16344	0.51393
O	-4.81544	-1.31788	-0.43497	C	2.082525	-0.08178	-0.23478
H	-0.84498	0.708259	0.341521	C	1.918659	1.344155	0.24032
H	-4.08286	0.206656	1.62028	C	-2.80291	1.327519	-1.10452
H	-2.38434	-0.2644	1.668336	C	-1.54829	2.059886	-0.64942
H	-0.4416	1.770145	-1.90874	C	-0.34084	1.563987	-0.97588
H	-1.69003	2.95924	-1.59702	C	1.025159	2.158427	-0.73997
H	-3.80603	-2.12096	1.099466	C	3.303509	2.015966	0.513667
H	0.54377	3.878186	-1.2968	C	4.179096	2.180138	-0.73912
H	-0.36732	3.98348	0.206148	C	3.1634	3.353075	1.259023
H	2.148178	1.500882	-1.38721	C	-3.70891	-2.47952	-0.134
H	1.553382	0.073745	1.25629	C	3.187333	-3.25678	0.732594
H	2.483231	-0.94002	-1.49461	O	2.222615	-2.70066	-1.34612
H	-2.32238	-1.2973	-1.44493	C	-1.76368	3.365963	0.078801
H	-2.92957	-2.9069	-1.04562	O	-4.33741	1.849337	0.675227
H	-0.09625	-1.31608	-1.44818	H	-0.63512	-1.04131	0.63846
H	1.458922	-2.52213	0.918013	H	-2.24951	0.276843	1.438177
H	1.74738	-3.13507	-0.70051	H	-3.83042	-0.45148	1.735616
H	3.788872	-1.51865	1.204043	H	-1.29761	-3.90246	-0.24191
H	5.383692	-2.97487	-0.10588	H	-0.41657	-2.90253	-1.35849
H	3.752521	-3.64727	-0.05941	H	-4.54687	0.219268	-0.58106
H	4.331396	-2.82785	-1.52103	H	0.544382	-3.39167	1.513493
H	5.848088	-0.59416	0.167964	H	0.919658	-4.47227	0.174836

H	1.536196	-1.03387	1.551639
H	2.41174	-0.21833	-1.26559
H	1.396847	1.314216	1.207442
H	-3.41038	2.013124	-1.71424
H	-2.5152	0.497168	-1.75906
H	-0.32741	0.611279	-1.50559
H	1.540637	2.213889	-1.71041
H	0.945941	3.187276	-0.37764
H	3.828951	1.324528	1.188267
H	5.16851	2.563482	-0.46281
H	4.328533	1.228826	-1.26152
H	3.742202	2.89086	-1.4509
H	4.146815	3.71776	1.579044
H	2.719046	4.131219	0.627328
H	2.537598	3.248528	2.153804
H	-3.32937	-3.46955	-0.39998
H	-4.48094	-2.60861	0.636828
H	-4.21962	-2.0794	-1.02052
H	4.096257	-2.71331	0.456863
H	3.08841	-3.23955	1.822925
H	3.288746	-4.29851	0.408677
H	1.522024	-2.20525	-1.80081
H	-2.3637	4.053218	-0.53402
H	-2.33628	3.203902	0.997419
H	-0.82815	3.870074	0.333359
H	-4.93823	1.476364	1.340277

07_1R4R10R_16

C	2.578758	1.393724	0.477212
C	1.444954	1.965348	0.920162
C	2.900295	-0.03236	0.87918
C	0.949643	3.367682	0.65656
C	3.184771	-0.99332	-0.29203
C	-0.57877	3.539459	0.742443
C	-1.43021	2.674775	-0.23208
C	-1.65587	1.267409	0.30844
C	-1.78435	0.165564	-0.43745
C	-2.08425	-1.22431	0.079242
C	2.794165	-2.44336	0.029211
C	1.321204	-2.82231	-0.00477
C	0.349741	-1.96857	-0.37308
C	-1.1182	-2.2618	-0.55786
C	-3.5795	-1.60016	-0.18707
C	-3.91684	-3.04731	0.215296
C	-4.54208	-0.64018	0.529918
C	3.606998	2.102243	-0.37142
C	-2.79305	3.355624	-0.44395
O	-0.82452	2.636989	-1.53439
C	1.092208	-4.27111	0.365011
O	4.595661	-0.90959	-0.55306
H	0.809225	1.366378	1.572115
H	2.073361	-0.43062	1.475753
H	3.797533	-0.05827	1.516079
H	1.391767	4.061122	1.388748
H	1.287294	3.722764	-0.32377
H	2.637691	-0.66214	-1.18676
H	-0.91782	3.343351	1.768849
H	-0.80049	4.594674	0.540798
H	-1.7718	1.194778	1.390736
H	-1.68986	0.260114	-1.52152
H	-1.93128	-1.23492	1.169051
H	3.217528	-2.70572	1.011178
H	3.318759	-3.09869	-0.68596
H	0.628782	-0.94462	-0.61155
H	-1.3345	-2.28945	-1.63973
H	-1.35854	-3.2561	-0.17836
H	-3.74988	-1.50355	-1.27089
H	-4.99268	-3.22821	0.106658
H	-3.3998	-3.79062	-0.39862
H	-3.65593	-3.23412	1.265538
H	-5.58246	-0.888	0.288898
H	-4.42781	-0.71699	1.61969
H	-4.36703	0.401671	0.248836
H	4.535623	2.241642	0.199762
H	3.883295	1.507776	-1.24901
H	3.27182	3.086929	-0.70671
H	-3.40939	2.762602	-1.12638
H	-3.32741	3.459125	0.506599
H	-2.65183	4.354396	-0.87238
H	-0.02213	2.09473	-1.44648
H	1.526144	-4.48959	1.350275
H	0.037629	-4.55205	0.394478
H	1.595041	-4.93908	-0.34855
H	4.772366	-1.41084	-1.36554

07_1R4R10R_17

C	2.317026	1.921838	0.296526
C	0.982172	2.047801	0.364204
C	2.965579	0.676448	0.866228
C	0.111014	3.138221	-0.21366

C	3.538879	-0.27073	-0.2112	H	-4.48407	1.915621	0.342033
C	-1.27545	3.254831	0.449086	H	-3.65259	2.59529	1.749832
C	-2.34138	2.209336	0.031035	H	-3.97265	3.620141	0.331579
C	-2.00493	0.797368	0.483843	H	-3.14568	1.696461	-1.67534
C	-1.81753	-0.26463	-0.30679	H	2.775357	-4.4415	-0.11931
C	-1.57316	-1.67883	0.171728	H	2.456388	-3.94677	1.537619
C	3.413572	-1.75229	0.173409	H	1.108531	-4.3562	0.468637
C	2.046363	-2.41445	0.074445	H	5.25853	-0.40535	-1.1442
C	0.949274	-1.78568	-0.38533	07_1R4R10R_18			
C	-0.42296	-2.36758	-0.61726	C	-2.46474	1.781115	-0.37357
C	-2.86905	-2.55523	0.128394	C	-1.14487	2.020706	-0.41661
C	-3.90075	-2.09545	1.167705	C	-2.98639	0.465171	-0.9122
C	-3.51033	-2.6458	-1.26604	C	-0.38696	3.201717	0.14153
C	3.240135	2.915319	-0.3646	C	-3.48086	-0.49824	0.189154
C	-3.69553	2.610971	0.654911	C	1.009086	3.398768	-0.47832
O	-2.43654	2.299348	-1.39755	C	2.132492	2.453857	0.017812
C	2.082134	-3.86361	0.508047	C	1.930948	1.002844	-0.39425
O	4.921638	0.085161	-0.37706	C	1.843593	-0.0485	0.426423
H	0.441689	1.248369	0.864484	C	1.785746	-1.50228	0.007852
H	2.228818	0.129287	1.462995	C	-3.20897	-1.97124	-0.14834
H	3.800495	0.930669	1.534959	C	-1.78912	-2.50223	-0.00661
H	0.611525	4.111002	-0.12677	C	-0.75987	-1.77017	0.4593
H	-0.04026	2.97491	-1.28957	C	0.641538	-2.24905	0.752372
H	3.01297	-0.10323	-1.16215	C	3.171919	-2.19085	0.254298
H	-1.17178	3.224432	1.541594	C	3.165965	-3.69615	-0.07011
H	-1.69577	4.237413	0.201774	C	4.292626	-1.51274	-0.55064
H	-1.96029	0.668678	1.566947	C	-3.4857	2.710751	0.234105
H	-1.8472	-0.11945	-1.38738	C	3.475529	2.938312	-0.57098
H	-1.26839	-1.63604	1.227071	O	2.164287	2.601851	1.444556
H	3.808068	-1.87968	1.19365	C	-1.6922	-3.96029	-0.40146
H	4.10342	-2.32612	-0.46787	O	-4.89374	-0.2729	0.329065
H	1.03338	-0.73557	-0.65809	H	-0.52608	1.255428	-0.87917
H	-0.64792	-2.29056	-1.69214	H	-2.19321	-0.02842	-1.48295
H	-0.43937	-3.43673	-0.38298	H	-3.83235	0.619006	-1.5973
H	-2.55189	-3.56998	0.412145	H	-0.95901	4.126508	-0.00745
H	-4.76892	-2.76545	1.174503	H	-0.26431	3.097532	1.228275
H	-3.47381	-2.09008	2.178102	H	-2.98661	-0.25082	1.13954
H	-4.26021	-1.08279	0.951932	H	0.947831	3.318917	-1.57156
H	-4.36077	-3.33756	-1.24986	H	1.345347	4.419228	-0.25715
H	-3.88758	-1.67018	-1.59601	H	1.927307	0.836372	-1.47311
H	-2.80622	-3.00749	-2.02334	H	1.843216	0.127311	1.504149
H	2.705209	3.760569	-0.80604	H	1.58501	-1.55353	-1.07293
H	3.965176	3.310311	0.361171	H	-3.57164	-2.16414	-1.16996
H	3.834492	2.434278	-1.15118	H	-3.85367	-2.58932	0.498812

H	-0.9357	-0.72362	0.699561
H	0.825789	-2.13779	1.834049
H	0.719522	-3.31643	0.545264
H	3.406954	-2.07748	1.324388
H	4.183212	-4.09976	-0.00578
H	2.544594	-4.27872	0.616121
H	2.804149	-3.87642	-1.09123
H	5.259146	-1.98273	-0.33451
H	4.110249	-1.61139	-1.62927
H	4.374995	-0.44646	-0.32462
H	-3.03604	3.613368	0.656705
H	-4.22509	3.016995	-0.5195
H	-4.05414	2.205818	1.025009
H	4.302314	2.317518	-0.20467
H	3.476703	2.882769	-1.66533
H	3.661762	3.976157	-0.2745
H	2.914704	2.078352	1.77057
H	-2.32952	-4.58263	0.242496
H	-2.05886	-4.10279	-1.42716
H	-0.67826	-4.36159	-0.35391
H	-5.19141	-0.76791	1.109462

07_1R4R10R_19

C	2.345689	1.751448	0.403229
C	1.124093	2.125918	0.821966
C	2.899289	0.406528	0.831132
C	0.394752	3.41278	0.50739
C	3.333722	-0.5208	-0.33071
C	-1.1325	3.359187	0.701558
C	-1.9152	2.338053	-0.17366
C	-1.84092	0.92866	0.398566
C	-1.789	-0.19868	-0.31879
C	-1.76376	-1.598	0.254606
C	3.204456	-2.00878	0.028112
C	1.814052	-2.62654	0.059416
C	0.711841	-1.96545	-0.33345
C	-0.69922	-2.4814	-0.45183
C	-3.16726	-2.28799	0.224766
C	-4.1354	-1.63735	1.222937
C	-3.79927	-2.34847	-1.17519
C	3.23968	2.61037	-0.45849
C	-3.38943	2.770211	-0.24972
O	-1.44518	2.373147	-1.53026
C	1.82927	-4.06666	0.520079
O	4.669895	-0.23037	-0.76596
H	0.594859	1.44161	1.48406
H	2.147998	-0.11033	1.437793
H	3.782699	0.54426	1.476778
H	0.770576	4.221197	1.15342
H	0.607079	3.739375	-0.51776
H	2.725927	-0.31303	-1.21707
H	-1.36486	3.16249	1.757188
H	-1.52498	4.359149	0.479791
H	-1.87478	0.866208	1.487156
H	-1.77066	-0.11756	-1.40672
H	-1.47781	-1.52617	1.313789
H	3.692853	-2.18017	1.002293
H	3.81006	-2.56894	-0.70008
H	0.818749	-0.92671	-0.63959
H	-0.95319	-2.53766	-1.52194
H	-0.78451	-3.50281	-0.06697
H	-2.99971	-3.3233	0.558522
H	-5.09076	-2.17532	1.24377
H	-3.72477	-1.64395	2.240123
H	-4.34306	-0.5957	0.952404
H	-4.74186	-2.90761	-1.14272
H	-4.02504	-1.34413	-1.55288
H	-3.14869	-2.84391	-1.90424
H	2.743689	3.523218	-0.79795
H	4.136123	2.908134	0.104134
H	3.603656	2.061608	-1.33387
H	-3.95683	2.062439	-0.86154
H	-3.83653	2.802828	0.749602
H	-3.46748	3.767353	-0.69764
H	-0.55911	1.97342	-1.52716
H	2.459333	-4.67908	-0.14006
H	2.266797	-4.14574	1.524853
H	0.838018	-4.52368	0.550501
H	5.259261	-0.3879	-0.0097

07_1R4R10R_20

C	-2.73615	1.334432	-0.44989
C	-1.44204	1.564141	-0.17633
C	-3.14398	0.033834	-1.11819
C	-0.81921	2.765632	0.487746
C	-3.7172	-1.03743	-0.14308
C	0.495073	3.250778	-0.1654
C	1.782977	2.433698	0.140925
C	1.74875	1.044568	-0.47534
C	2.052553	-0.09396	0.15549

C	2.052094	-1.47355	-0.45823	H	-2.06326	-2.28194	2.656577
C	-2.81439	-2.27878	-0.01819	H	-0.42961	-1.62065	2.474616
C	-1.44059	-2.08608	0.597148	H	-5.59647	-0.80839	-0.64438
C	-0.33941	-2.38616	-0.11433	07_1R4R10R_21			
C	1.102801	-2.43486	0.322425	C	2.840252	0.79567	0.650519
C	3.488754	-2.07467	-0.57429	C	1.94724	1.453623	-0.10709
C	4.366353	-1.26642	-1.5399	C	2.706101	-0.71995	0.790827
C	4.194795	-2.25935	0.778935	C	1.775141	2.929996	-0.33595
C	-3.87428	2.269941	-0.12034	C	2.799293	-1.51941	-0.52696
C	2.997497	3.196765	-0.41443	C	0.504788	3.528354	0.317265
O	2.002196	2.376042	1.558432	C	-0.87235	2.994847	-0.1724
C	-1.43089	-1.63101	2.037398	C	-1.18165	1.603197	0.36326
O	-4.97772	-1.5542	-0.59639	C	-1.79591	0.623065	-0.30899
H	-0.74228	0.778871	-0.45085	C	-2.17622	-0.71954	0.277806
H	-2.28772	-0.38953	-1.65517	C	2.171351	-2.91044	-0.40854
H	-3.91573	0.233944	-1.87467	C	0.654364	-2.98294	-0.27275
H	-1.52082	3.606635	0.496423	C	-0.1586	-1.98295	-0.65534
H	-0.62203	2.55086	1.550727	C	-1.66188	-1.90234	-0.5951
H	-3.85285	-0.58519	0.851027	C	-3.71665	-0.78855	0.545059
H	0.361374	3.309083	-1.25378	C	-4.56931	-0.80785	-0.73372
H	0.68927	4.272331	0.183876	C	-4.0927	-1.95999	1.467095
H	1.4695	1.016951	-1.52987	C	3.954726	1.439414	1.437217
H	2.344228	-0.04059	1.204826	C	-1.97173	3.959674	0.30678
H	1.671552	-1.3913	-1.48652	O	-0.94633	3.036888	-1.60649
H	-2.71822	-2.71477	-1.02025	C	0.177128	-4.29985	0.294961
H	-3.37289	-3.0058	0.589727	O	4.166443	-1.72266	-0.91167
H	-0.49216	-2.70238	-1.14864	H	1.188661	0.844178	-0.59386
H	1.20206	-2.25527	1.397995	H	1.740668	-0.94342	1.260536
H	1.456447	-3.46368	0.153326	H	3.48007	-1.11555	1.460012
H	3.353735	-3.07532	-1.01228	H	2.63679	3.49332	0.038126
H	5.339716	-1.75213	-1.67838	H	1.729627	3.13183	-1.41641
H	3.89407	-1.17453	-2.5258	H	2.294651	-0.96059	-1.32583
H	4.547065	-0.25372	-1.1616	H	0.562391	3.395	1.405424
H	5.152319	-2.77515	0.64049	H	0.517162	4.609427	0.128993
H	4.409655	-1.29602	1.256537	H	-0.90333	1.439836	1.405634
H	3.59728	-2.85633	1.477073	H	-2.07909	0.801142	-1.34751
H	-3.55189	3.18021	0.390927	H	-1.68956	-0.80198	1.260088
H	-4.41157	2.564797	-1.0326	H	2.629682	-3.42101	0.451293
H	-4.61283	1.776026	0.527385	H	2.481475	-3.49332	-1.28866
H	3.919119	2.658744	-0.17272	H	0.299944	-1.08682	-1.06606
H	2.928734	3.299305	-1.50231	H	-2.04795	-1.7708	-1.61709
H	3.049009	4.198229	0.02699	H	-2.09178	-2.83632	-0.22358
H	1.309694	1.809598	1.935832	H	-3.95865	0.13688	1.086913
H	-1.84136	-0.61674	2.126374	H	-5.63486	-0.7555	-0.48082

H	-4.34369	0.04011	-1.38974
H	-4.41476	-1.72927	-1.30765
H	-5.15193	-1.90298	1.745049
H	-3.93667	-2.9318	0.983762
H	-3.50279	-1.9465	2.39162
H	4.013985	2.521664	1.295136
H	3.831571	1.248005	2.512396
H	4.926259	1.007003	1.159712
H	-2.95019	3.611657	-0.03724
H	-1.9879	4.018853	1.400136
H	-1.79262	4.964011	-0.09312
H	-0.37469	2.329819	-1.94693
H	0.569775	-5.13851	-0.29674
H	0.55447	-4.44077	1.317254
H	-0.91062	-4.39272	0.322179
H	4.552564	-0.84299	-1.05153

07_1R4R10R_22

C	2.316929	1.921806	0.296601
C	0.98206	2.047721	0.364229
C	2.965486	0.676422	0.866246
C	0.110875	3.138022	-0.21383
C	3.538949	-0.2706	-0.21124
C	-1.27556	3.254792	0.448937
C	-2.34153	2.20929	0.031058
C	-2.00502	0.797325	0.483865
C	-1.81755	-0.26464	-0.30679
C	-1.57297	-1.67884	0.17162
C	3.413653	-1.75219	0.17326
C	2.046414	-2.4143	0.074309
C	0.949419	-1.78555	-0.38573
C	-0.42283	-2.3674	-0.61764
C	-2.86878	-2.55535	0.128455
C	-3.90044	-2.09559	1.167823
C	-3.51021	-2.64607	-1.2659
C	3.24004	2.915358	-0.36442
C	-3.69561	2.610945	0.655071
O	-2.43689	2.299254	-1.39753
C	2.08204	-3.8633	0.50841
O	4.921685	0.085343	-0.37696
H	0.44159	1.248234	0.86444
H	2.228649	0.129155	1.46282
H	3.800307	0.930576	1.535135
H	0.611392	4.110827	-0.12723
H	-0.04046	2.974443	-1.28969
H	3.013131	-0.10306	-1.16224
H	-1.17188	3.224544	1.541453
H	-1.69584	4.237362	0.201508
H	-1.96033	0.668645	1.56697
H	-1.84726	-0.11942	-1.38737
H	-1.26804	-1.63609	1.226917
H	3.808207	-1.87967	1.193473
H	4.10344	-2.326	-0.4681
H	1.03365	-0.7355	-0.6587
H	-0.64791	-2.29012	-1.69247
H	-0.4392	-3.43661	-0.38361
H	-2.55153	-3.57006	0.412262
H	-4.76853	-2.7657	1.174754
H	-3.4734	-2.0901	2.17818
H	-4.26005	-1.08299	0.952007
H	-4.36063	-3.33786	-1.24957
H	-3.88752	-1.6705	-1.59593
H	-2.80616	-3.00781	-2.02324
H	2.705118	3.760605	-0.80588
H	3.964995	3.310351	0.361439
H	3.834519	2.434378	-1.15094
H	-4.48421	1.915618	0.342277
H	-3.65256	2.595276	1.749991
H	-3.97273	3.620124	0.331759
H	-3.14594	1.696225	-1.67521
H	2.776605	-4.44109	-0.11755
H	2.454539	-3.94597	1.53867
H	1.108727	-4.35635	0.46749
H	5.25872	-0.40528	-1.14397

07_1R4R10R_23

C	-2.66076	1.457253	-0.11043
C	-1.32456	1.583687	-0.13031
C	-3.35205	0.452018	-1.01406
C	-0.45933	2.523818	0.669184
C	-3.91446	-0.80844	-0.30511
C	0.701449	3.139792	-0.14612
C	2.037739	2.361437	-0.09725
C	1.914097	0.940431	-0.62046
C	1.999417	-0.17018	0.118715
C	1.967057	-1.58632	-0.40449
C	-2.97652	-2.0286	-0.34346
C	-1.65829	-1.9609	0.404817
C	-0.51514	-2.25445	-0.23869
C	0.875935	-2.43346	0.313427

C	3.365038	-2.2808	-0.31879	C	1.692153	-2.2425	-0.25394
C	4.390638	-1.61073	-1.24324	C	3.687613	-0.78063	-0.36354
C	3.913295	-2.39226	1.112889	C	0.60802	-3.12656	0.3138
C	-3.59031	2.282646	0.74987	C	3.910216	0.744426	-0.27793
C	3.095027	3.113276	-0.9304	C	-0.66141	-3.25289	-0.56775
O	2.429098	2.378873	1.284952	C	-1.90982	-2.47528	-0.09237
C	-1.759	-1.66713	1.882545	C	-1.65024	-0.98839	0.063409
O	-5.10674	-1.27133	-0.96045	C	-2.29887	0.003055	-0.55931
H	-0.77701	0.915838	-0.79266	C	-2.12226	1.496553	-0.35507
H	-2.67974	0.135926	-1.82066	C	2.826753	1.593581	-0.97343
H	-4.20326	0.953447	-1.50026	C	1.657414	2.015346	-0.10504
H	-1.06296	3.335845	1.087596	C	0.430501	1.517597	-0.32283
H	-0.02497	1.997004	1.529957	C	-0.83991	1.856659	0.424419
H	-4.15321	-0.56275	0.740684	C	-3.39142	2.138282	0.301903
H	0.403223	3.263984	-1.19452	C	-3.37826	3.673592	0.197373
H	0.919959	4.143564	0.238627	C	-4.7111	1.611625	-0.28815
H	1.7767	0.857508	-1.7	C	2.463823	-1.11347	1.867608
H	2.114934	-0.06147	1.19754	C	-3.08927	-2.8026	-1.01873
H	1.711135	-1.55064	-1.47362	O	-2.19302	-3.00791	1.226828
H	-2.79532	-2.26852	-1.39842	C	1.969473	3.045966	0.956038
H	-3.56252	-2.86433	0.068842	O	5.139092	1.06734	-0.9507
H	-0.58881	-2.45791	-1.30953	H	1.859362	-2.38238	-1.32449
H	0.907966	-2.23889	1.39038	H	4.609934	-1.24529	0.028268
H	1.145333	-3.49444	0.188264	H	3.637124	-1.04736	-1.42636
H	3.208102	-3.30451	-0.69105	H	1.034994	-4.13595	0.416491
H	5.338375	-2.16229	-1.23663	H	0.318506	-2.83158	1.32609
H	4.030318	-1.57499	-2.27874	H	3.99236	1.04466	0.777285
H	4.598153	-0.58148	-0.92845	H	-0.43925	-2.95024	-1.59838
H	4.846213	-2.96816	1.119955	H	-0.96144	-4.30697	-0.60911
H	4.135847	-1.40498	1.535334	H	-0.87672	-0.75931	0.792912
H	3.209826	-2.89506	1.785776	H	-3.05435	-0.2591	-1.29699
H	-3.06289	2.969292	1.416015	H	-2.03917	1.941964	-1.36119
H	-4.27305	2.875445	0.124212	H	2.466832	1.056102	-1.85892
H	-4.22579	1.643414	1.377693	H	3.340502	2.495497	-1.33499
H	4.068779	2.613673	-0.85643	H	0.324618	0.777953	-1.11474
H	2.817821	3.150277	-1.99003	H	-0.8671	1.343955	1.398442
H	3.203135	4.138988	-0.56199	H	-0.85229	2.924903	0.660095
H	3.255426	1.872203	1.352306	H	-3.37086	1.864256	1.36795
H	-2.09947	-0.63771	2.051303	H	-4.26605	4.096743	0.681746
H	-2.49507	-2.32786	2.361071	H	-2.50242	4.127722	0.670347
H	-0.80844	-1.78866	2.407027	H	-3.39239	3.989679	-0.85407
H	-5.75336	-0.54865	-0.92746	H	-5.56255	2.139195	0.15707
07_1R4R10R_24				H	-4.75326	1.775461	-1.37331
C	2.522473	-1.40156	0.386036	H	-4.85177	0.542443	-0.10318

H	1.701659	-1.70279	2.382291
H	3.430271	-1.32822	2.34506
H	2.248124	-0.05405	2.055965
H	-4.00875	-2.31665	-0.6727
H	-2.9013	-2.48118	-2.04895
H	-3.25821	-3.88375	-1.02092
H	-2.91038	-2.46728	1.596621
H	2.406197	3.947351	0.503549
H	1.086805	3.347153	1.525677
H	2.711032	2.674093	1.675647
H	5.836089	0.510713	-0.56718

07_1R4R10R_25

C	2.415821	1.801263	0.304533
C	1.10643	2.062313	0.443879
C	2.970044	0.49805	0.842516
C	0.330337	3.243865	-0.09285
C	3.440055	-0.4776	-0.25939
C	-1.05221	3.444588	0.556271
C	-2.18801	2.497407	0.092211
C	-1.97259	1.055544	0.521303
C	-1.84509	-0.00294	-0.28452
C	-1.74741	-1.43815	0.189062
C	3.202	-1.94987	0.108402
C	1.783927	-2.49779	0.029713
C	0.734722	-1.77809	-0.40715
C	-0.68678	-2.24013	-0.61475
C	-3.15743	-2.1185	0.175229
C	-3.74304	-2.30008	-1.23396
C	-3.17356	-3.44693	0.94855
C	3.39364	2.70609	-0.40401
C	-3.52061	2.989063	0.697135
O	-2.2443	2.623846	-1.33573
C	1.707762	-3.94784	0.45459
O	4.843948	-0.23487	-0.45075
H	0.514043	1.323387	0.97802
H	2.202892	0.005509	1.448542
H	3.837898	0.672138	1.495077
H	0.904824	4.168927	0.047107
H	0.185763	3.143985	-1.17708
H	2.911064	-0.25263	-1.19671
H	-0.96708	3.371725	1.648431
H	-1.39128	4.46452	0.336509
H	-1.97152	0.902202	1.602592
H	-1.83064	0.158823	-1.36301
H	-1.42753	-1.42364	1.240836
H	3.605182	-2.12315	1.11834
H	3.830705	-2.56802	-0.55425
H	0.899983	-0.73626	-0.67454
H	-0.92429	-2.14331	-1.68543
H	-0.78747	-3.30251	-0.37957
H	-3.81604	-1.42406	0.716109
H	-4.76764	-2.6861	-1.17445
H	-3.77688	-1.35411	-1.78611
H	-3.15937	-3.0145	-1.82671
H	-4.20018	-3.81948	1.047091
H	-2.5935	-4.22714	0.441564
H	-2.76312	-3.32621	1.95853
H	2.915512	3.593577	-0.82733
H	4.181039	3.03907	0.287223
H	3.909931	2.173494	-1.21214
H	-4.35106	2.355725	0.361885
H	-3.49903	2.957889	1.79234
H	-3.71778	4.019176	0.381591
H	-2.99047	2.081956	-1.64106
H	2.34483	-4.57562	-0.18423
H	2.085745	-4.06791	1.479187
H	0.697073	-4.35951	0.423606
H	5.12303	-0.74137	-1.23064

07_1R4R10R_26

C	2.691938	1.070063	0.635914
C	1.784106	1.62244	-0.18625
C	2.744032	-0.44981	0.783534
C	1.461152	3.073609	-0.41209
C	2.942764	-1.24761	-0.52259
C	0.159581	3.55087	0.276234
C	-1.17185	2.89499	-0.16833
C	-1.35778	1.487581	0.382855
C	-1.74041	0.408706	-0.30774
C	-2.052	-0.94424	0.300856
C	2.482926	-2.70199	-0.39181
C	0.98503	-2.95021	-0.26076
C	0.067776	-2.02967	-0.60293
C	-1.43489	-2.10379	-0.53204
C	-3.59053	-1.10767	0.531746
C	-4.41393	-1.13501	-0.76568
C	-3.92326	-2.32086	1.415127
C	3.647647	1.843121	1.511832
C	-2.34035	3.762002	0.351914

O	-1.17193	2.914834	-1.60255	C	-1.2797	2.853403	-0.10113
C	0.656344	-4.33139	0.256633	C	-1.57118	1.46138	0.441988
O	4.329452	-1.2931	-0.89051	C	-1.77069	0.365018	-0.29583
H	1.138964	0.945686	-0.74048	C	-2.11942	-0.99831	0.262359
H	1.813651	-0.78682	1.256849	C	2.574404	-2.64284	-0.04165
H	3.559137	-0.74042	1.458056	C	1.074663	-2.89837	0.001224
H	2.271945	3.720061	-0.05704	C	0.166418	-1.97623	-0.36002
H	1.361905	3.258634	-1.48879	C	-1.33091	-2.12139	-0.4635
H	2.385656	-0.76511	-1.33601	C	-3.6667	-1.22918	0.24936
H	0.253465	3.422773	1.362134	C	-4.27972	-1.26511	-1.15962
H	0.06658	4.629164	0.092314	C	-4.07897	-2.47242	1.053997
H	-1.22947	1.406299	1.464047	C	3.760926	1.836236	-0.44184
H	-1.85621	0.490379	-1.38981	C	-2.59518	3.646545	-0.17658
H	-1.59024	-0.97468	1.29824	O	-0.80059	2.792641	-1.45352
H	2.993676	-3.1469	0.475435	C	0.737751	-4.30727	0.434292
H	2.86401	-3.25343	-1.26445	O	4.422278	-1.26762	-0.84057
H	0.426029	-1.07547	-0.97972	H	0.928268	1.315006	1.518204
H	-1.84048	-2.0493	-1.55409	H	2.049356	-0.56988	1.421217
H	-1.76578	-3.06133	-0.12123	H	3.795493	-0.34585	1.432427
H	-3.89201	-0.21018	1.09077	H	1.784314	3.966874	1.272849
H	-5.48616	-1.1433	-0.53663	H	1.510524	3.602589	-0.41411
H	-4.21547	-0.25784	-1.39168	H	2.511273	-0.85643	-1.25032
H	-4.20123	-2.0298	-1.36282	H	-0.54881	3.45952	1.851339
H	-4.98598	-2.31686	1.685622	H	-0.40355	4.696289	0.610815
H	-3.7226	-3.27011	0.904519	H	-1.65782	1.39565	1.528021
H	-3.3412	-2.31007	2.344806	H	-1.69844	0.449746	-1.38095
H	3.57509	2.925853	1.380094	H	-1.81952	-1.0133	1.320103
H	3.466091	1.622705	2.573337	H	3.015249	-2.9478	0.922552
H	4.686598	1.545486	1.312044	H	3.014429	-3.32191	-0.78726
H	-3.30125	3.335021	0.039746	H	0.525247	-0.99032	-0.6483
H	-2.34155	3.816677	1.446351	H	-1.6034	-2.09622	-1.53023
H	-2.26074	4.778984	-0.04742	H	-1.65856	-3.09496	-0.08985
H	-2.06183	2.660233	-1.89592	H	-4.08968	-0.35583	0.765989
H	1.133418	-5.09954	-0.36786	H	-5.37217	-1.33495	-1.09654
H	1.051301	-4.47115	1.272487	H	-4.03867	-0.36325	-1.73323
H	-0.41529	-4.54121	0.279802	H	-3.93065	-2.13281	-1.73209
H	4.60997	-0.37515	-1.03671	H	-5.16922	-2.51138	1.164794
07_1R4R10R_27				H	-3.77038	-3.40231	0.561952
C	2.689195	1.206696	0.41521	H	-3.64055	-2.4625	2.059387
C	1.605683	1.863908	0.864952	H	3.511111	2.856265	-0.7446
C	2.894295	-0.24429	0.804715	H	4.711883	1.875535	0.108561
C	1.22096	3.301993	0.599972	H	3.959387	1.2421	-1.34059
C	3.063548	-1.22631	-0.38075	H	-3.31897	3.116341	-0.80284
C	-0.27225	3.62393	0.800824	H	-3.02801	3.776802	0.821095

H	-2.41219	4.637363	-0.6074
H	-0.04251	2.183935	-1.45161
H	1.184731	-5.04159	-0.25041
H	1.157479	-4.51641	1.427947
H	-0.33561	-4.50319	0.47655
H	4.967621	-1.57768	-0.09858

07_1R4R10R_28

C	2.505665	-1.41188	0.369976
C	1.662144	-2.24532	-0.26255
C	3.666202	-0.80129	-0.39355
C	0.585672	-3.13269	0.314699
C	3.924092	0.711182	-0.28236
C	-0.69021	-3.26078	-0.55627
C	-1.92896	-2.46555	-0.0859
C	-1.65341	-0.98065	0.061122
C	-2.29554	0.014434	-0.5625
C	-2.10767	1.506647	-0.36021
C	2.849954	1.603407	-0.95245
C	1.674622	2.004768	-0.08235
C	0.44539	1.524067	-0.32374
C	-0.82624	1.856809	0.424763
C	-3.37361	2.160561	0.291217
C	-3.35062	3.694939	0.175194
C	-4.69665	1.638256	-0.29505
C	2.471625	-1.13659	1.854715
C	-3.11292	-2.78584	-1.00892
O	-2.21671	-2.98611	1.237237
C	1.985096	2.999645	1.012993
O	5.192762	0.8919	-0.93327
H	1.812047	-2.37762	-1.33665
H	3.582748	-1.04632	-1.45971
H	4.593184	-1.27837	-0.0392
H	1.015318	-4.14149	0.412388
H	0.304084	-2.83935	1.329866
H	4.023319	0.988839	0.777658
H	-0.47108	-2.97618	-1.59274
H	-1.00022	-4.31255	-0.57962
H	-0.87579	-0.75626	0.787553
H	-3.05493	-0.24325	-1.29771
H	-2.01677	1.950345	-1.36644
H	2.49955	1.106008	-1.86488
H	3.360925	2.523932	-1.27759
H	0.337865	0.809637	-1.13842
H	-0.85592	1.335991	1.394165
H	-0.83689	2.923058	0.670029
H	-3.35514	1.894277	1.359258
H	-4.23235	4.127917	0.661922
H	-2.46857	4.14664	0.639
H	-3.36931	4.003216	-0.8785
H	-5.54449	2.175088	0.145966
H	-4.73769	1.79392	-1.38144
H	-4.84459	0.571549	-0.1017
H	1.717415	-1.72997	2.376609
H	3.445883	-1.35608	2.313432
H	2.259875	-0.07875	2.056544
H	-4.02744	-2.29017	-0.66348
H	-2.92352	-2.47031	-2.04072
H	-3.29178	-3.86542	-1.00653
H	-2.92839	-2.43585	1.603816
H	2.419459	3.91741	0.591458
H	1.102068	3.280373	1.592253
H	2.72677	2.60494	1.72034
H	5.418099	1.833727	-0.87171

07_1R4R10R_29

C	2.464841	1.780973	0.373896
C	1.14495	2.020568	0.416792
C	2.986334	0.464855	0.912254
C	0.387168	3.201613	-0.14144
C	3.480732	-0.49824	-0.18943
C	-1.00893	3.398828	0.478198
C	-2.13232	2.453913	-0.01796
C	-1.93085	1.002926	0.394155
C	-1.84341	-0.04847	-0.42645
C	-1.78584	-1.50221	-0.0077
C	3.209018	-1.97134	0.147692
C	1.789094	-2.50234	0.006566
C	0.759731	-1.77044	-0.4593
C	-0.64174	-2.24938	-0.75192
C	-3.17214	-2.19047	-0.25426
C	-3.16651	-3.69586	0.069765
C	-4.29269	-1.51231	0.550847
C	3.485862	2.710657	-0.23358
C	-3.4754	2.9384	0.570736
O	-2.16407	2.601862	-1.44468
C	1.692335	-3.96025	0.401988
O	4.893537	-0.27273	-0.32954
H	0.526082	1.255218	0.879142
H	2.193029	-0.02884	1.482719

H	3.832252	0.618339	1.597486	H	-2.54542	-4.27839	-0.61675
H	0.959279	4.126373	0.007508	H	-2.80456	-3.87651	1.090773
H	0.264644	3.097341	-1.2282	H	-5.25932	-1.98204	0.334632
H	2.986274	-0.25056	-1.13965	H	-4.11032	-1.61123	1.629458
H	-0.94784	3.319157	1.571464	H	-4.37485	-0.44596	0.325064
H	-1.34511	4.419273	0.256825	H	3.036296	3.613593	-0.6556
H	-1.92739	0.836445	1.473017	H	4.225526	3.016341	0.519982
H	-1.84282	0.127225	-1.5042	H	4.053954	2.205959	-1.02489
H	-1.58527	-1.55333	1.073116	H	-4.30216	2.317589	0.204409
H	3.572284	-2.1646	1.169031	H	-3.47664	2.882937	1.665092
H	3.853289	-2.58923	-0.50009	H	-3.66163	3.976225	0.274172
H	0.93549	-0.72394	-0.69985	H	-2.91396	2.077682	-1.77079
H	-0.82607	-2.13872	-1.83365	H	2.330403	-4.58267	-0.24116
H	-0.71976	-3.31665	-0.5442	H	2.058195	-4.10214	1.428063
H	-3.40717	-2.07682	-1.32432	H	0.678582	-4.36194	0.353784
H	-4.18388	-4.09918	0.005505	H	5.191402	-0.76865	-1.10928

Table S6. DP4+ results obtained using experimental data of compound 7 *versus* isomers 7a-7d

Functional		Solvent?		Basis Set		Type of Data	
mPW1PW91		PCM		6-311G(d,p)		Shielding Tensors	
		DP4+	0.02%	94.40%	5.57%	0.00%	-
Nuclei	sp2?	xperimenta	Isomer 1	Isomer 2	Isomer 3	Isomer 4	Isomer 5
C		51.96	131.79198	132.085563	133.034999	122.489875	
C	x	122.1	49.3210835	49.544084	51.6863656	45.5630418	
C	x	141.04	39.1342075	39.1418062	38.0373854	33.9795216	
C		74.2	111.267796	110.304144	109.729583	101.166329	
C		44.21	140.136888	139.976138	140.964259	130.921017	
C		23.66	162.305787	160.711794	161.250456	147.916103	
C	x	127.31	49.4880538	50.5581502	51.7661471	48.9011985	
C	x	130.05	47.9550564	47.731788	47.2842666	41.5763432	
C		46.88	139.662912	136.785495	139.824217	126.669345	
C		68.82	114.002245	113.342709	113.894237	103.664556	
C		38.96	139.34176	139.249682	139.590288	127.46339	
C	x	132.94	47.4902038	46.5905988	46.7588256	42.8204473	
C	x	128.47	53.6425279	52.3861372	53.5314434	50.954407	
C		23.68	152.565522	152.876705	151.922136	139.943149	
C		29.13	150.388884	149.631942	150.204653	136.649331	
C		21.15	167.398118	166.135774	165.632923	152.919533	
C		21.85	166.196385	165.596201	169.468369	153.323665	
C		29.17	158.788359	157.198445	157.879622	141.713484	
C		15.32	169.150835	169.819074	169.120236	155.246101	
C		15.09	170.552496	167.48634	169.8699828	154.4454298	
